# An ecological-evolutionary perspective on the genomic diversity and habitat preferences of the Acidobacteriota

**DOI:** 10.1099/mgen.0.001344

**Published:** 2025-01-29

**Authors:** Ella McReynolds, Mostafa S. Elshahed, Noha H. Youssef

**Affiliations:** 1 Department of Microbiology and Molecular Genetics, Oklahoma State University, Stillwater, OK, USA

**Keywords:** *Acidobacteria*, comparative genomics, metagenomics, niche colonization, soil

## Abstract

Members of the phylum *Acidobacteriota* inhabit a wide range of ecosystems including soils. We analysed the global patterns of distribution and habitat preferences of various *Acidobacteriota* lineages across major ecosystems (soil, engineered, host-associated, marine, non-marine saline and alkaline and terrestrial non-soil ecosystems) in 248 559 publicly available metagenomic datasets. Classes *Terriglobia*, *Vicinamibacteria*, *Blastocatellia* and *Thermoanaerobaculia* were highly ubiquitous and showed a clear preference to soil over non-soil habitats, while classes *Aminicenantia* and *Holophagae* showed preferences to non-soil habitats. However, while specific preferences were observed, most *Acidobacteriota* lineages were habitat generalists rather than specialists, with genomic and/or metagenomic fragments recovered from soil and non-soil habitats at various levels of taxonomic resolution. Comparative analysis of 1930 genomes strongly indicates that phylogenetic affiliation plays a more important role than the habitat from which the genome was recovered in shaping the genomic characteristics and metabolic capacities of the *Acidobacteriota*. The observed lack of strong habitat specialization and habitat-transition-driven lineage evolution in the *Acidobacteriota* suggest ready cross-colonization between soil and non-soil habitats. We posit that such capacity is key to the successful establishment of *Acidobacteriota* as a major component in soil microbiomes post-ecosystem disturbance events or during pedogenesis.

**Impact Statement**

The phylum *Acidobacteriota* is widespread in various ecosystems on Earth. However, our understanding of the preference and distribution patterns of various acidobacterial lineages in various habitats is currently incomplete. In this study, we examined the distribution patterns of various lineages in the phylum *Acidobacteriota* across major ecosystems using 248 559 publicly available metagenomic datasets in 17 617 projects comprising 1.3 Pbp of sequencing data. We identify clear soil preferences for multiple lineages, many of which are yet uncultured and poorly characterized. Further, we demonstrate that most *Acidobacteriota* classes, orders, families and genera are habitat generalists rather than specialists. We also use comparative analysis of 1930 genomes to provide new insights into the genomic features, predicted physiological optima and metabolic repertoires of members of the *Acidobacteriota* and disentangle the role played by phylogeny versus habitat in shaping *Acidobacteriota* genomic and predicted metabolic features.

## Data Summary

No new data were generated as part of this study, and all sequence data analysed are publicly available. Sequences from the 248 559 metagenomes examined were assessed through the Sandpiper interface (https://sandpiper.qut.edu.au/). Sequences for 2028 *Acidobacteriota* genomes were downloaded from GenBank.

## Introduction

The phylum *Acidobacteriota* [1] (previously, the *Acidobacteria*) represents one of the most prevalent phyla encountered in soils, as evident in 16S rRNA gene-based surveys, isolation efforts and metagenomic studies [2,15]. Additionally, members of the phylum *Acidobacteriota* are also encountered in a wide range of habitats other than soil, e.g. hydrothermal vents [16], anoxic freshwater mud [17], hydrocarbon-contaminated aquifer [18], marine chiton [19], alkaline hot springs [20], termite nests [21] and marine sediments [22]. Several classification schemes have been proposed for the *Acidobacteriota* [2,23, 24]. The current Genome Taxonomy Database (GTDB, release 220) [25] classifies the phylum *Acidobacteriota* into 15 classes, 54 orders, 110 families and 581 genera, many of which do not have a pure culture representative (for example, only 7 of the 15 classes, 15 of the 54 orders, 17 of the 110 families and 38 of the 581 genera in GTDB have a pure culture representative or a *Candidatus* status).

Based on isolation and culture-independent efforts, preference of specific *Acidobacteriota* lineages to a certain habitat has been observed. For example, *Acidobacteriota* groups 1, 3, 4 and 6 (Barns [2] classification scheme) are the most prevalent members of the *Acidobacteriota* in diversity surveys [9] with several representative isolates described from soil [6,7, 26,29], while representatives of groups 8, 10 and 23 have been isolated from non-soil habitats (e.g. hydrothermal vent, chiton, anoxic sediments and hot springs) [16,18,30]. However, a detailed global meta-analysis to elucidate patterns of distribution and habitat preference for cultured and yet-uncultured lineages at various levels of phylogenetic resolution is currently lacking. The current availability of hundreds of thousands of metagenomic datasets, as well as a genome-based reference database and taxonomic outlines that encompass cultured as well as uncultured taxa [25], allows for assessing global ecological distribution patterns of target *Acidobacteriota* lineages at various levels of phylogenetic resolution using metagenomic read mapping approaches. Such approaches are superior to assessments that rely on documenting the habitat origin of isolates, 16S rRNA sequences or genomes deposited in databases, where issues regarding differential amenability of lineages to culturing, sequence deposition procedures, preferential amplification of lineages and genome assembly problems could impact the accuracy of the obtained outcome. Read mapping-based assessments would also go beyond cursory characterization of habitat preferences by providing quantitative metrics for ubiquity (occurrence of a lineage in a specific ecosystem), preference (comparison of ubiquity levels of a specific lineage across ecosystems) and relative abundance (percentage of reads belonging to a specific lineage within a metagenomic dataset). Further, such assessment could also be used to identify whether a specific lineage is a habitat specialist (i.e. restricted to a single habitat) or generalist (i.e. encountered in a wide range of habitats) at various taxonomic resolutions.

Progress in isolation, genome sequencing and – most importantly – generation of single amplified genomes (SAGs) and metagenome-assembled genomes (MAGs) from environmental samples has greatly increased the number of publicly available *Acidobacteriota* genomes. Currently, the GTDB release 220 contains 3175 *Acidobacteriota* genomes from a wide array of habitats. As such, a phylum-wide comparative genomic analysis to elucidate and expand on key genomic features, metabolic capacities and physiological preferences within various members of the *Acidobacteriota* is feasible. Such detailed analysis could also be used to disentangle the relative importance of phylogeny (lineage to which a genome belongs) versus habitat (origin from which the isolate, MAG or SAG was recovered) in shaping genomic features and metabolic capacities in the *Acidobacteriota*.

Here, we present a detailed analysis of the global distribution patterns of the *Acidobacteriota* based on fragment recruitment from 248 559 metagenomic studies. We combine this analysis with a detailed comparative genomic analysis based on thousands of *Acidobacteriota* genomes available in GenBank to identify key differences across lineages and habitats. Based on our results, we propose the occurrence of ready cross-colonization of *Acidobacteriota* between soil and non-soil habitats through a continuous niche-selection process. We posit that such capacity is key to the rapid and successful establishment of *Acidobacteriota* as a major component of the soil microbiome during pedogenesis or during recolonization post-drastic disturbance events. The implications of such findings on our understanding of how the evolution of soil as a distinct habitat on Earth impacted the evolutionary trajectory of the *Acidobacteriota* are further discussed.

## Methods

### Taxonomic framework for the *Acidobacteriota*

Several classification schemes have been proposed for the phylum *Acidobacteriota*. Barns *et al*. [2] classified the phylum into 26 subgroups based on amplicons identified in subsurface sediments. Dedysh and Yilmaz built on that scheme and assigned the 26 subgroups to 15 class-level units [23]. The GTDB [25] incorporates genomes from isolates as well as SAGs and MAGs into a global genome-based taxonomy, while using validly described names for isolates. We used GTDB release 214 for the current analysis. This specific release classified the phylum *Acidobacteriota* into 14 classes, 52 orders, 102 families and 486 genera (note that the most current GTDB release 220 has more genomes assigned to the *Acidobacteriota* and an updated number of taxa). Here, given the need for genome-based analysis, and the fact that the GTDB incorporates genomes from both cultured and uncultured lineages, we used the GTDB as our classification framework. Table S1 (available in the online version of this article) and [31] provide a comprehensive view of the phylum classification based on the three schemes discussed above [3,23, 25], as well as the silva classification database [24].

### Ecological distribution of the *Acidobacteriota*

We used Sandpiper, an interface that utilizes a recently developed tool (SingleM) for accurate mapping of metagenomic reads to genomes, to determine the occurrence and relative abundance of the phylum *Acidobacteriota*, its classes, orders and families [32] in metagenomes. The SingleM pipeline used to generate the Sandpiper interface was applied to public NCBI metagenomic datasets that were available at the time of analysis (July 2023) and that were assigned as either ‘metagenomic’ or as ‘derived from metagenomic organisms’. At the time of analysis, 248 559 metagenomic datasets derived from 17 617 projects comprising 1.3 Pbp of sequencing data and originating from all habitats (soil, engineered, freshwater, host associated, marine, non-marine saline and alkaline and terrestrial non-soil) were used. SingleM analyses reads covering only conserved regions of 59 single-copy marker genes and performs the search against proteins rather than nt database, hence allowing the identification of lineages without a complete or draft genome (many of the MAGs analysed here fit this category) and making it superior to other marker-based taxonomic profilers that depend on nt searches (e.g. MetaPhlAn) [32]. The taxonomic classification of SingleM and the Sandpiper version utilized for the current analysis is also dependent on GTDB taxonomy release 214, unlike other profilers (e.g. Kaiju, MAP2b and Kraken), and so could easily be adapted to the analysis here. For each taxon searched in Sandpiper, the output includes the number of datasets where the taxon was identified, along with individual accession numbers and ecological classification of each dataset, as well as the relative abundance of the taxon in each dataset. We believe that this approach is superior to other approaches for metagenomic profiling, since shotgun sequencing directly from an environment provides a less-biassed view of the available community that is not prone to experimental issues (e.g. primer bias) usually encountered with other culture-independent studies. Also, searching metagenomic reads obtained with shotgun sequencing to identify taxa without assembling the reads into contigs and binning the contigs into genomes (MAGs) would provide access to the fraction of the community that would otherwise not be binned into MAGs due to variability in coverage.

We used the output from Sandpiper searches to assess the distribution and prevalence patterns of the phylum *Acidobacteriota*, as well as its classes, orders and families recognized in the GTDB taxonomy (release 214) across six major habitats, as defined by Ivanova *et al.* [33]: soil (*n*=10 250 datasets), engineered (*n*=16 320 datasets), host-associated (*n*=165 152 datasets), marine (*n*=13 880 datasets), non-marine saline and alkaline ecosystems (*n*=1306 datasets) and terrestrial non-soil ecosystems (*n*=5039 datasets). For each taxon (the phylum, as well as each class, order and family), three criteria were assessed: (1) ubiquity (assessed as the percentage of the total datasets for each habitat classification where a specific lineage was identified by read mapping), (2) preference of a specific lineage for soil (assessed as the ratio of soil/non-soil datasets where a specific lineage was identified by read mapping) and (3) relative abundance of a specific lineage (assessed as the percentage of reads in a specific dataset that mapped to a target lineage). We considered ‘presence’ to be any non-zero coverage or relative abundance value from the Sandpiper output. Due to the overrepresentation of host-associated (mostly human and murine) datasets in that database, and the well-established scarcity of *Acidobacteriota* in this habitat, all comparative analyses between soil and non-soil habitats, e.g. preference values, were conducted after the exclusion of the host-associated datasets.

### Habitat generalization versus specialization estimation

Habitat specialization was assessed by identifying the range of habitats where a specific lineage (class, order and family) was encountered. The analysis was conducted for all taxa (classes, orders and families) encountered in the GTDB (release 214). However, it is important to note that taxa rarely encountered could erroneously be classified as specialists. Therefore, we also re-analysed such patterns using an empirical cutoff that excludes taxa encountered in less than 250 studies.

### Comparative genomic analysis

We assessed various genomic features (genome size, GC content, coding density, average gene length and number of protein-coding genes), phage infection/immunity features (number of viral contigs and number of Clustered Regularly Interspaced Short Palindromic Repeats (CRISPR) occurrences in a genome), potential extracellular products arsenal [CAZymes, peptidases and biosynthetic gene clusters (BGCs)], predicted physiological optima (temperature, pH and oxygen preferences) and predicted life history strategy (ruderal, competitor and scarcity) within *Acidobacteriota* genomes. We compared these characteristics across various lineages, as well as across habitats in a lineage-agnostic manner (i.e. comparing all genomes recovered from soil to those recovered from non-soil habitats). These genomes are derived from metagenomes representing all of Earth’s major biomes (soil, engineered, host-associated, marine, non-marine saline and alkaline and terrestrial non-soil ecosystems). In addition, we also assessed within-lineage habitat effects, i.e. whether genomic features differ between the same lineage genomes recovered from soil versus non-soil habitats. Out of the 2028 *Acidobacteriota* genomes available in the GTDB (release 214), we focused our comparative genomic analysis on 1930 genomes belonging to classes with at least 100 genomes and for which genomes were recovered from at least 5 of the 7 habitats (Table 1, Fig. S1). These classes are *Aminicenantia*, *Blastocatellia*, *Holophagae*, *Terriglobia*, *Thermoanaerobaculia* and *Vicinamibacteria*.

**Table 1.** Genomes compared in this study. Total number of genomes compared belonging to each of the six *Acidobacteriota* classes is shown. Number of genomes originating from soil, engineered, freshwater, host-associated, marine, non-marine saline and alkaline and terrestrial non-soil is also shown for each class

Class	Total no. of genomes	No. of genomes from soil	No. of genomes from non-soil environments					
			Engineered	Freshwater	Host associated	Marine	Non-marine saline and alkaline	Terrestrial non-soil
*Blastocatellia*	215	64	103	35	8	1	0	4
*Terriglobia*	1006	537	179	197	50	16	0	27
*Thermoanaerobaculia*	153	25	49	22	37	17	0	3
*Vicinamibacteria*	231	44	35	59	47	46	0	0
*Aminicenantia*	119	7	27	49	0	35	1	0
*Holophagae*	206	16	30	151	4	5	0	0

General genomic features (including genome size, GC content, coding density and number of genes) were retrieved from the GTDB metadata file (available at https://data.gtdb.ecogenomic.org/releases/release214/214.1/). The average gene length was calculated using the getFastaStats.pl script (available at https://github.com/bioinformagical/scripts-cluster/blob/master/getFastaStats.pl). CRISPRs were predicted using CCTyper [34]. Viral contigs were predicted using VirSorter2 [35]. For secretory capacities, CAZymes were predicted using dbCAN3 [36], while BGCs were predicted using antiSMASH [37]. Proteases were identified via comparisons to the MEROPS database [38].

We used recently developed machine learning (ML)-based bioinformatic approaches to predict physiological preferences from genomic data. To predict optimal growth temperatures (OGTs), we used the Tome suite [39]. pH preferences were assessed using a newly devised ML approach [40]. To predict oxygen preference from genomic data, we used the recently developed aerobicity ML predictor [41].

To predict life history strategy (scarcity, ruderal or competitor), we used the recently developed ML approach that measures the trade-off between genomic investment in regulatory flexibility and resource acquisition to predict the ecological strategy [42]. Genomic investment in regulatory flexibility was calculated as the number of transcription factors relative to the total gene number [42]. Transcription factors were predicted using two approaches: (1) examining the output of BlastKOALA [43] for the presence of transcription factor KEGG Orthologies (KOs) (a list is available at https://www.genome.jp/brite/ko03000) and (2) predicting additional transcription factors using DeepTFactor [44]. Genomic investment in resource acquisition was calculated as the number of genes encoding secreted enzymes (CAZymes, proteases and lipases/hydrolases) plus the number of predicted BGCs divided by the total number of membrane transporters [42]. CAZymes and proteases were predicted as detailed above. Lipases/hydrolases were identified through comparison to the ESTHER database [45]. All three groups of enzymes were then subjected to SignalP [46] analysis to identify those with a secretion signal. Finally, membrane transporters were predicted by examining the output of BlastKOALA [43] for the presence of transporters KOs (a list is available at https://www.genome.jp/brite/ko02000). Both genomic investments in regulatory flexibility (the number of transcription factors divided by the total number of genes) and in resource acquisition (the number of genes encoding secreted CAZymes, proteases and lipases/hydrolases plus the number of BGCs divided by the total number of membrane transporters) were then used to predict the life history strategy (one of scarcity, ruderal or competitor) of all compared *Acidobacteriota* genomes via k-means clustering using the R package flexclust [47] and the k-centroid cluster analysis. The training dataset was comprised of the 27 ^13^C-labelled MAGs from Barnett *et al.* [42]. Metabolic potential encoded in *Acidobacteriota* genomes was predicted using METABOLIC [48].

### Statistical analysis

Histograms and Q-Q plots of residuals as well as Shapiro–Wilk tests for normality showed that the general genomic features, phage infection/immunity features, potential extracellular products arsenal, predicted physiological optima and predicted life history strategy were slightly departed from normal distribution. However, due to the sufficiently large sample size (1930 genomes), we opted to use the parametric ANOVA to test for the effect of phylogeny (class), habitat (the environmental source from which the genomes were obtained) and the interaction between the two on these features. Specifically, type III ANOVA (run through the ANOVA command in the R package ‘car’ [49]) was used to accommodate the interaction term. Factors with F-test *P*-value <1×10^−5^ were considered significant. The percentage contribution of phylogeny (class) and habitat (the environmental sources from which the genomes were obtained) was calculated based on the F-test sum of squares. For all significant comparisons, the TukeyHSD command in R was used for multiple comparisons of means.

To test for the effect of phylogeny and habitat on metabolic features predicted in the genomes, we first converted the dichotomous output (present/absent) from METABOLIC into 1/0 numerical output (if a pathway was identified as present in a genome, a value of 1 was used, while a value of 0 was used if the pathway was absent). Since these features are not normally distributed, the effect of phylogeny, habitat and their interaction on the metabolic potential (either the pattern of occurrence of KEGG modules or TIGRfam/Pfam/custom HMM functions as predicted by METABOLIC) was tested using the logistic regression model (using glm in R). The percentage contribution of the two variables to the overall model was calculated using the difference between McFadden pseudo *R*^2^ (using pR2 in the package pscl in R [50]) calculated for the full model and for the model when the variable was removed.

## Results

### Global patterns of *Acidobacteriota* distribution across biomes

**Fig. 1.**
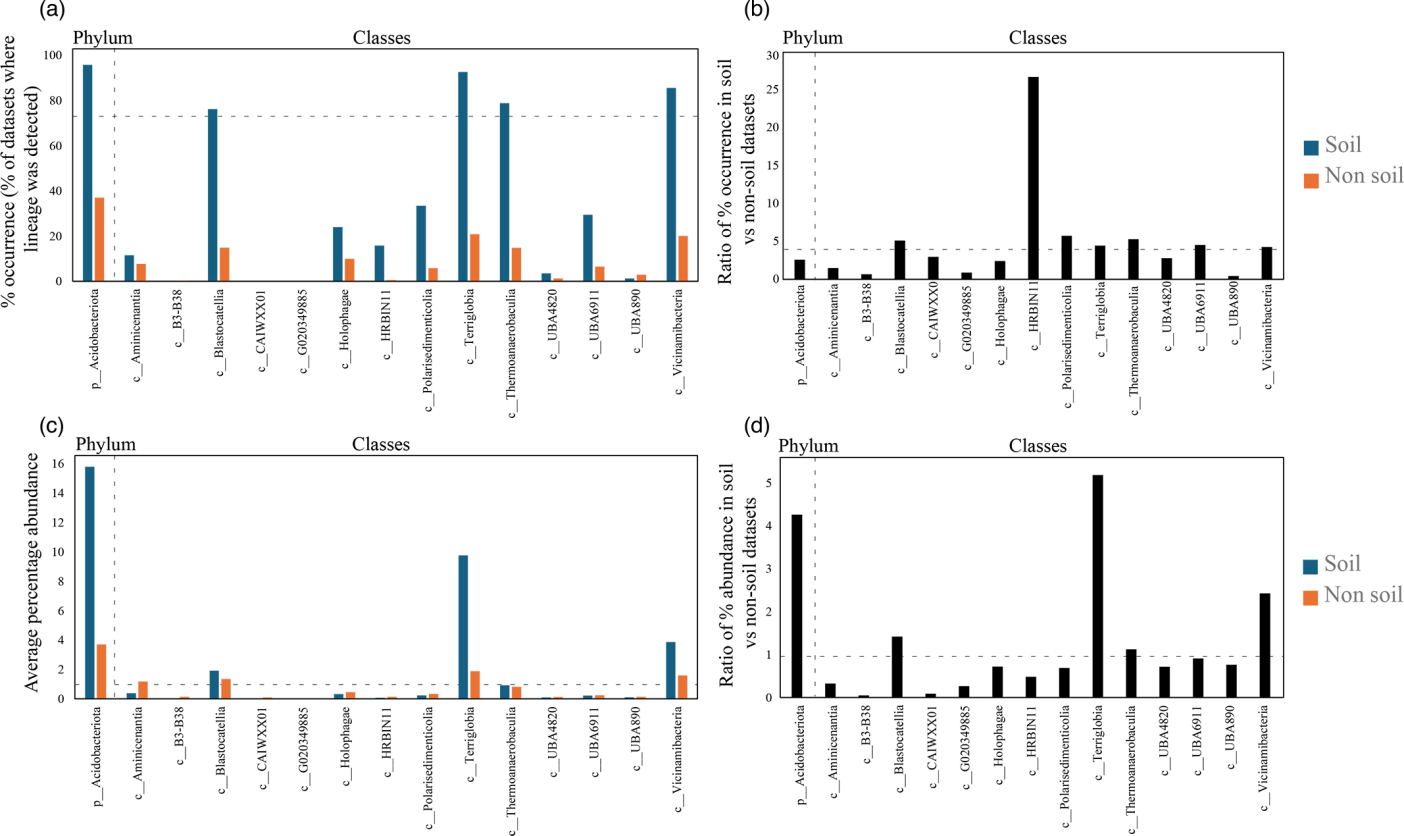
Ecological distribution of the phylum *Acidobacteriota* and its 14 classes in 248 559 metagenomic datasets available through the web interface Sandpiper [32]. (a) Percentage occurrence of members of the phylum *Acidobacteriota* and its 14 classes in datasets originating from soil (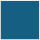) and non-soil (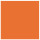) ecosystems. Classes are shown on the X-axis. The dotted line represents 75% occurrence, the cutoff used to define a lineage as ubiquitous in an ecosystem. (b) The ratio of percentage occurrence of the phylum and each of its 14 classes in soil versus non-soil ecosystems. The dotted line represents a ratio of 4, the cutoff used to define a lineage as soil preferring in an ecosystem. (c) Average percentage abundance of members of the phylum *Acidobacteriota* and its 14 classes in datasets originating from soil (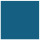) and non-soil (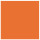) ecosystems. Classes are shown on the X-axis. The dotted line represents 1% occurrence, the cutoff used to define a lineage as abundant in an ecosystem. (d) The ratio of percentage abundance of the phylum and each of its 14 classes in soil versus non-soil ecosystems. The dotted line represents a ratio of 1, the cutoff used to define a lineage as relatively more abundant in an ecosystem.

At the phylum level, *Acidobacteriota* showed the highest level of ubiquity in soil ecosystems, with fragments mapped to 95.7% of soil-derived datasets (9814 out of 10 250 soil datasets) (Table S2, Fig. 1a). In comparison, *Acidobacteriota*-associated metagenomic fragments were mapped to only 37.6% of non-soil-derived datasets (18 027 out of 47 953 datasets from engineered, freshwater, marine, non-marine saline and alkaline and terrestrial non-soil habitats) (Table S2, Fig. 1a). Similarly, *Acidobacteriota* exhibited higher mean relative abundance in soil-derived datasets, where it was encountered on average in 15.79% of reads, compared to only 3.71% of reads in non-soil-derived datasets (Table S2, Fig. 1c).

**Fig. 2.**
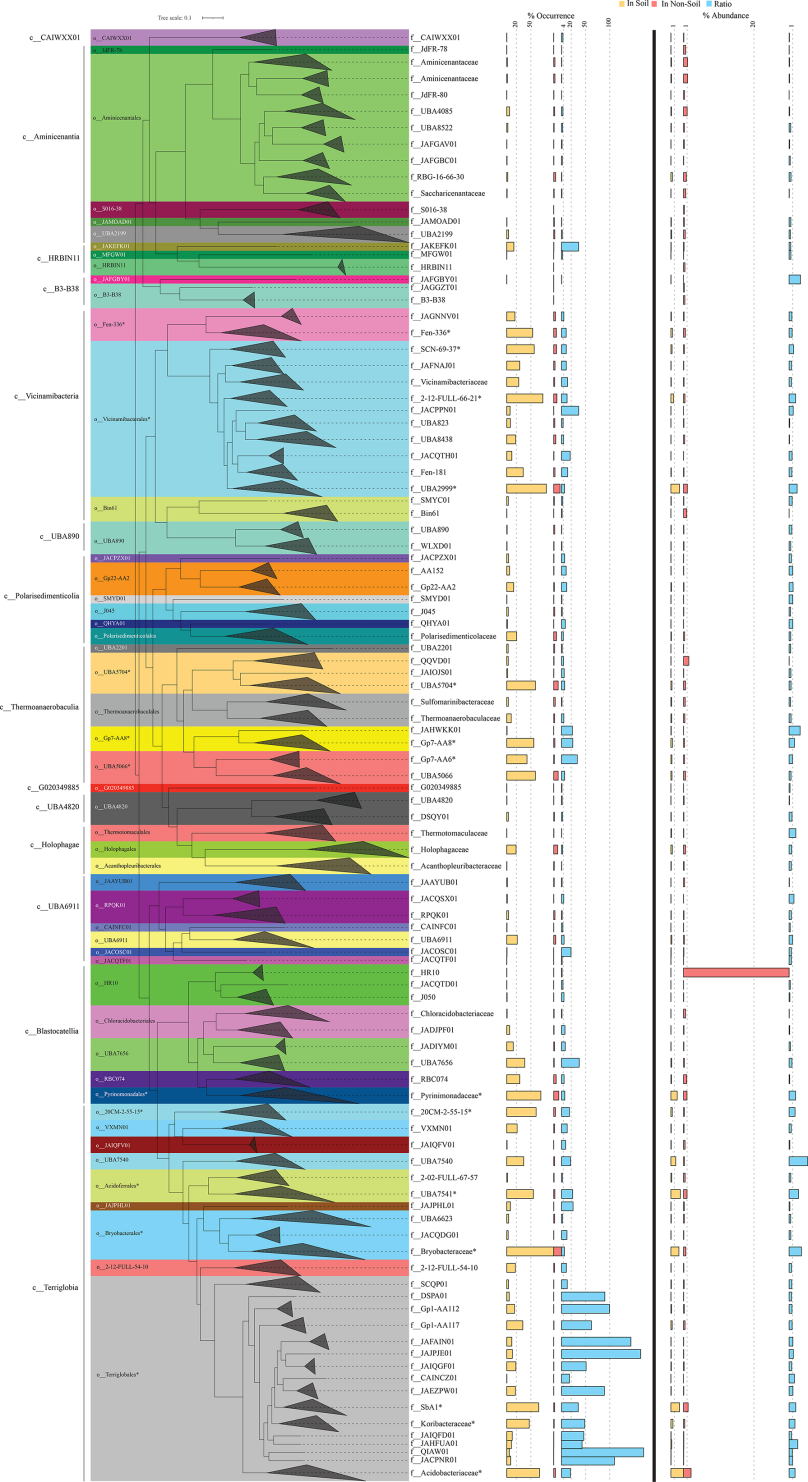
Phylogenomic tree constructed using the GTDB concatenated alignment of 120 single-copy marker gene. The tree was constructed in FastTree [66] and is wedged at the family level. Family names are shown on the right. Wedges are colour coded by order (shown to the left of the tree). Class names are shown to the left. Families and orders with higher soil ubiquity and preference values are highlighted by an asterisk (*). The values corresponding to the percentage occurrence of each family in soil (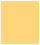) and non-soil (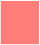) ecosystems and the ratio (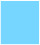) of percentage occurrence in soil versus non-soil ecosystems are shown as horizontal bars to the direct right of the tree. Families with >25% occurrence in soil datasets were considered ubiquitous in soil, while families with a ratio of percentage occurrence in soil versus non-soil ecosystems >4 were considered soil preferring. For ease of visualization, dashed vertical lines are shown for the 20 and 50% occurrence in soil datasets and for the soil/non-soil percentage occurrence ratios of 4, 20, 50 and 100. Horizontal bars to the right of the thick vertical line represent the average percentage abundance of each family in soil (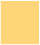) and non-soil (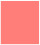) ecosystems, as well as the ratio (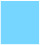) of percentage abundance in soil versus non-soil ecosystems. Families with a ratio of percentage abundance in soil versus non-soil ecosystems >1 were considered more abundant in soil. For ease of visualization, dashed vertical lines are shown for the 1% abundance in soil datasets, 1 and 20% abundance in non-soil datasets and for the soil/non-soil percentage abundance ratio of 1.

Four out of the 14 classes of *Acidobacteriota* were highly ubiquitous in soil (cutoff used for ubiquity in soil occurred in >75% of soil datasets). These were classes *Terriglobia* (occurred in 92.61% of soil datasets), *Vicinamibacteria* (occurred in 85.61% of soil datasets), *Thermoanaerobaculia* (occurred in 78.84% of soil datasets) and *Blastocatellia* (occurred in 76.2% of soil datasets) (Figs1  2, Table S2). In addition, these four classes also showed a strong preference to soil (cutoff used for preference to soil was a ratio of occurrence in the soil to non-soil datasets >4) (Fig. 1b, Table S2). This pattern was mostly driven by higher soil ubiquity and preference values for 14 families belonging to 10 orders within these 4 classes (highlighted in Fig. 2). Interestingly, while many of these lineages have representative isolates and are currently recognized as prevalent members of the microbial communities in soil, e.g. families *Acidobacteriaceae*, *Koribacteraceae*, *Bryobacteraceae*, *Pyrinomonadaceae* and *Vicinamibacteraceae,* corresponding to subgroups 1, 3, 4 and 6 (Barns classification system), others represent lineages mostly identified through metagenomic studies and have, so far, no cultured representatives or recognition in prior amplicon-based [2] and amplicon- and isolate-based [23] taxonomic schemes. For example, within the *Terriglobia*, in addition to families *Acidobacteriaceae* and *Koribacteraceae* (subgroup 1) and *Bryobacteraceae* (subgroup 3), members of the yet-uncultured families 20 CM-2-55-15, UBA7541 and SbA1 also showed high levels of ubiquity and preference to soil ecosystems (Fig. 2). Within the class *Thermoanaerobaculia*, named based on the earliest isolates from high-temperature settings [30], multiple uncultured families showed high levels of ubiquity and preference to the soil, e.g. families Gp7-AA8, Gp7-AA6 and UBA5704. Similarly, within the class *Vicinamibacteria*, in addition to the family *Vicinamibacteraceae* (subgroup 6) [7], the uncultured families Fen-336, 2–12-FULL-66–21, SCN-69–37 and UBA2999 showed high levels of ubiquity and preference to the soil. Finally, within the *Blastocatellia*, in addition to the ubiquitous soil family *Pyrinomonadaceae* (subgroup 4) [6], the uncultured family UBA7656 also showed high ubiquity and preference to soil.

*Acidobacteriota* classes that were less ubiquitous in soil included *Polarisedimenticolia* (encountered in 33.47% of soil studies), UBA6911 (encountered in 29.48% of soil studies), *Holophagae* (encountered in 24% of soil studies), HRBIN11 (encountered in 15.78% of soil studies) and *Aminicenantia* (encountered in 11.5% of soil studies) (Figs1  2, Table S2). Within these five classes, UBA6911, *Polarisedimenticolia* and HRBIN11 showed preference to soil (soil/non-soil occurrence ratio of >4, Fig. 1b, Table S2), while classes *Holophagae* and *Aminicenantia* showed less soil preferences (ratio of occurrence in soil to non-soil datasets <4). Finally, the remaining classes (B3-B38, CAIWXX01, UBA4820, UBA890 and G020349885) were extremely rare (less than 4%) in all habitats examined.

In addition to ubiquity and preference, we also assessed relative abundance values (proportion of reads mapped to a specific lineage within a metagenomic dataset) of various classes, orders and families in the *Acidobacteriota*, an indirect measure of their niche-colonization capacities and relative contribution to ecosystem functions within a specific habitat (Fig. 1c). Classes identified as most ubiquitous in soil also showed the highest relative abundance in soil, with relative abundance values of 9.76, 3.87, 1.9 and 0.92 % for *Terriglobia*, *Vicinamibacteria*, *Blastocatellia* and *Thermoanaerobaculia*, respectively (Fig. 1c). Out of these four classes, *Terriglobia* had the highest ratio of relative abundance between soil and non-soil datasets (5.2), followed by *Vicinamibacteria*, *Blastocatellia* and *Thermoanaerobaculia* with soil/non-soil relative abundance ratios of 2.43, 1.42 and 1.12, respectively (Fig. 1d). On the other hand, classes with lower ubiquity in soil (*Polarisedimenticolia*, HRBIN11, *Holophagae*, *Aminicenantia* and UBA6911) showed lower relative abundance in soil compared to non-soil habitats with ratios consistently <1 (Fig. 1d, Table S2).

Therefore, based on the above analysis of soil ubiquity, preference and relative abundance, we infer that members of the four classes *Terriglobia*, *Vicinamibacteria*, *Blastocatellia* and *Thermoanaerobaculia* show a high level of ubiquity, preference and relative abundance in soil over other habitats. We refer to these lineages henceforth as ‘soil-preferring lineages (SPLs)’. On the other hand, members of the two classes *Holophagae* and *Aminicenantia* show lower soil ubiquity, lower preference to soil habitats and lower relative abundance in soil versus non-soil datasets. We refer to these two lineages henceforth as ‘non-soil preferring lineages (NSPLs)’. The three moderately soil-ubiquitous *Acidobacteriota* classes UBA6911, *Polarisedimenticolia* and HRBIN11 showed moderate relative abundance values that were lower in soil, compared to non-soil habitats (values of soil/non-soil relative abundance ratio of <1). Finally, it is interesting to note that the number of genera in SPLs (50, 52, 70 and 209 genera in *Thermoanaerobaculia*, *Blastocatellia*, *Vicinamibacteria* and *Terriglobia*, respectively, average 95.25±76.36) is higher than in NSPLs (17 and 34 in *Holophagae* and *Aminicenantia*, respectively, average 25.5±12.02), as well as in the moderately ubiquitous *Acidobacteriota* classes (*Polarisedimenticolia*, UBA6911 and HRBIN11, with 19, 19 and 3 genera, respectively, average 13.67±9.24). Such fine-scale taxonomic diversity and underpinning genomic diversity and gene-content variabilities can theoretically enable these classes to persist and adapt in various environments.

### Habitat generalization versus specialization in the *Acidobacteriota*

We examined patterns of habitat generalization versus specialization in the *Acidobacteriota* at various levels of taxonomic resolution. We found 78.6, 61.5 and 52.9% of classes, orders and families, respectively, within the *Acidobacteriota* to be habitat generalists (i.e. with reads belonging to these lineages identified in each of the seven habitat classifications) (Table S3). The exclusion of rare taxa from our datasets (empirically defined as those encountered in less than 250 studies) results in an even higher percentage of generalist taxa (91.7, 77.5 and 66.7% of classes, orders and families, respectively). Our analysis indicates that preference to a specific type of habitat does not necessarily correspond to specialization, and lineages identified as SPLs or NSPLs could still be habitat generalists and exhibit wide global multi-habitat distribution patterns. For example, families *Acidobacteriaceae*, *Bryobacteraceae*, *Pyrinomonadaceae* and *Vicinamibacteraceae,* while encountered in 24.76–97.32% of soil datasets, were also present in 2.03–19.75% of engineered, 4–31% of freshwater, 0.61–4.93% of marine, 0.53–10.8% of non-marine saline and alkaline and 1.47–3.65% of terrestrial non-soil datasets. On the other hand, NSPL families *Aminicenantaceae* and *Holophagaceae,* while enriched in non-soil datasets, were also present in 1.23 and 19.26%, respectively, of soil datasets.

To confirm the results obtained from the global metagenomic dataset utilized, we examined the habitat generalization and specialization patterns in the collection of 2028 acidobacterial genomes recognized in the GTDB (release 214) [25]. Our results (Fig. S2, Table S3) confirm habitat generalization of the *Acidobacteriota* at the class, order and family levels, with 85.7, 65.4 and 54.91% of classes, orders and families, respectively, within the *Acidobacteriota* with genome representatives obtained from two or more habitat classifications (Table S3). At the genus level, 24.69% of genera had genome representatives obtained from more than one habitat, while 75.3% had genome representatives obtained from only one habitat. Further, when removing lineages defined by less than 5 genomes (404 genera, 48 families, 15 orders and 3 classes), 91.67, 84.49, 87.04 and 62.2% of classes, orders, families and genera, respectively, were not specialized to one type of habitat (Table S3).

### Comparative genomic features across lineages and habitats

**Fig. 3.**
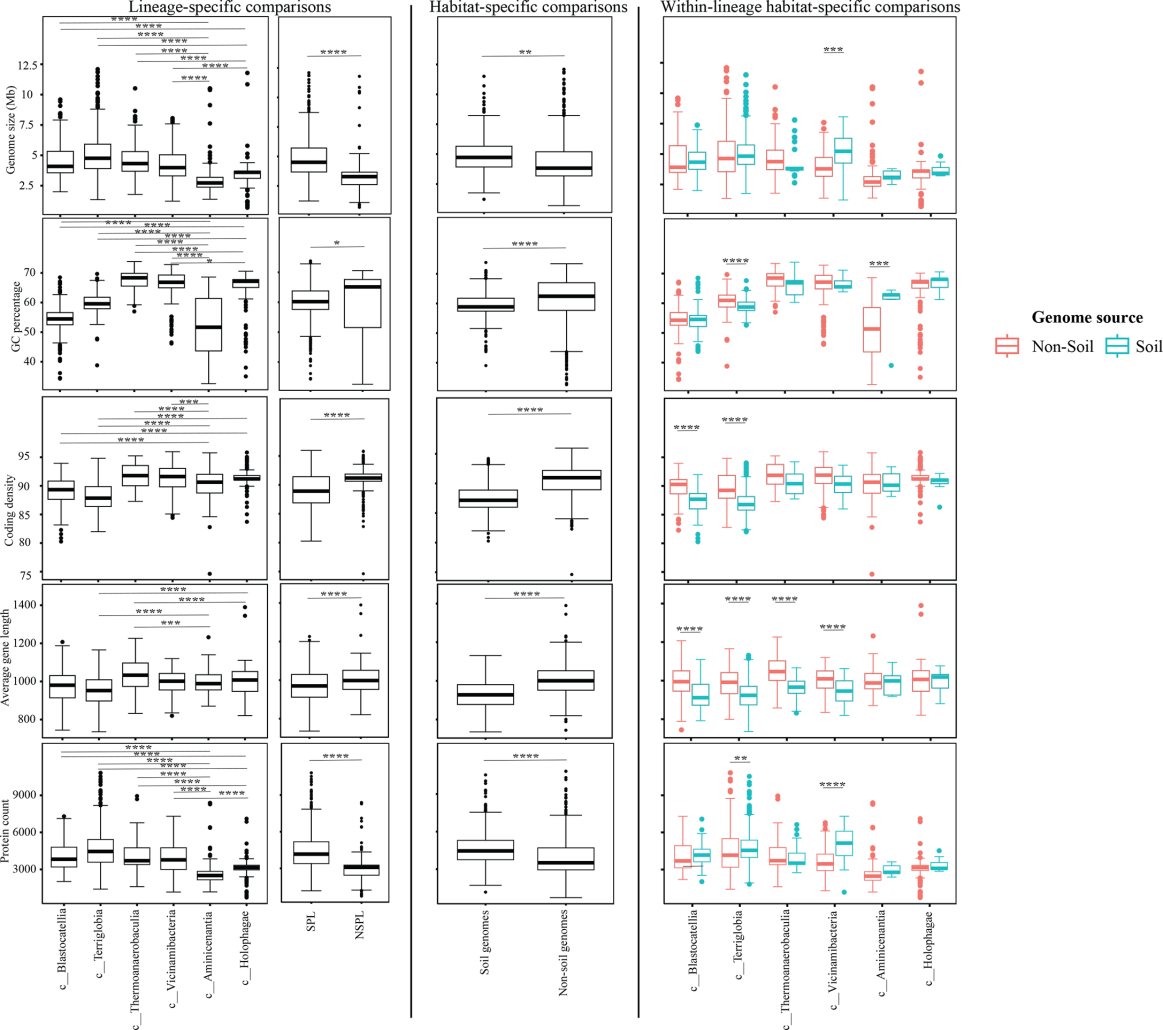
Box plots for the distribution of several general genomic features in the 1930 genomes of the 6 *Acidobacteriota* classes compared in this study. Table S4 shows the actual data used to construct these plots. Features shown are genome size (top row), genome percentage GC (second row from top), genome coding density (third row from top), average gene length (second to last row) and protein count (bottom row). Results for two-tailed ANOVA followed by Tukey for pairwise comparisons are shown on top of the box plots only for significant comparisons. *, 0.01<*P*<0.05; **, *P*<0.01; ***, *P*<0.001; ****, *P*<0.0001; *****, *P*<0.00001. The first two columns show results for lineage-specific comparisons at the *Acidobacteriota* class level, as well as when combining *Acidobacteriota* SPLs (*Terriglobia*, *Vicinamibacteria*, *Thermoanaerobaculia* and *Blastocatellia*) versus NSPLs (*Holophagae* and *Aminicenantia*). The third column shows results for habitat-specific comparisons (genomes originating from soil versus non-soil environments regardless of phylogeny). Results of within-lineage habitat-specific comparisons are shown in the fourth column, where genomes from soil origin are shown in cyan, while genomes from non-soil origin are shown in red. The exact *P*-values for all comparisons are shown in Table S5.

**Fig. 4.**
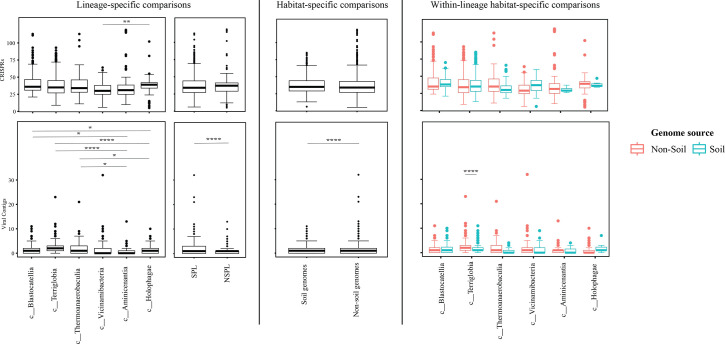
Box plots for the distribution of phage infection/immunity features in the 1930 genomes of the 6 *Acidobacteriota* classes compared in this study. Table S4 shows the actual data used to construct these plots. Features shown are the number of CRISPRs (top row) and number of viral contigs (bottom row). Results for two-tailed ANOVA followed by Tukey for pairwise comparisons are shown on top of the box plots only for significant comparisons. *, 0.01<*P*<0.05; **, *P*<0.01; ***, *P*<0.001; ****, *P*<0.0001; *****, *P*<0.00001. The first two columns show results for lineage-specific comparisons at the *Acidobacteriota* class level, as well as when combining *Acidobacteriota* SPLs (*Terriglobia*, *Vicinamibacteria*, *Thermoanaerobaculia* and *Blastocatellia*) versus NSPLs (*Holophagae* and *Aminicenantia*). The third column shows results for habitat-specific comparisons (genomes originating from soil versus non-soil environments regardless of phylogeny). Results of within-lineage habitat-specific comparisons are shown in the fourth column, where genomes from soil origin are shown in cyan, while genomes from non-soil origin are shown in red. The exact *P*-values for all comparisons are shown in Table S5.

**Fig. 5.**
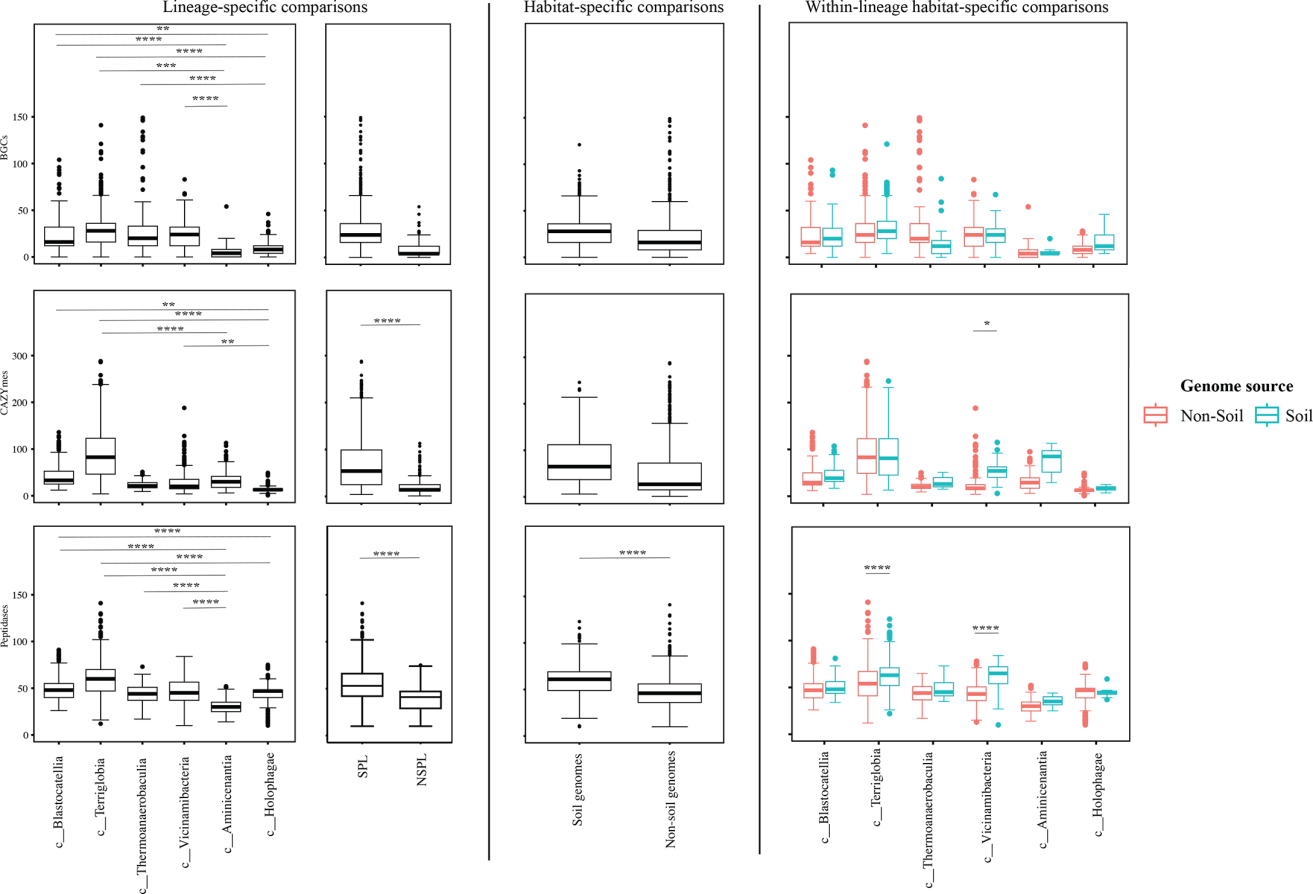
Box plots for the distribution of potential extracellular products arsenal in the 1930 genomes of the 6 *Acidobacteriota* classes compared in this study. Features shown are the number of BGCs (top row), number of CAZymes (middle row) and number of proteases (bottom row). Results for two-tailed ANOVA followed by Tukey for pairwise comparisons are shown on top of the box plots only for significant comparisons. *, 0.01<*P*<0.05; **, *P*<0.01; ***, *P*<0.001; ****, *P*<0.0001; *****, *P*<0.00001. The first two columns show results for lineage-specific comparisons at the *Acidobacteriota* class level, as well as when combining *Acidobacteriota* SPLs (Terriglobia, *Vicinamibacteria*, *Thermoanaerobaculia* and *Blastocatellia*) versus NSPLs (*Holophagae* and *Aminicenantia*). The third column shows results for habitat-specific comparisons (genomes originating from soil versus non-soil environments regardless of phylogeny). Results of within-lineage habitat-specific comparisons are shown in the fourth column, where genomes from soil origin are shown in cyan, while genomes from non-soil origin are shown in red. The exact *P*-values for all comparisons are shown in Table S5.

**Fig. 6.**
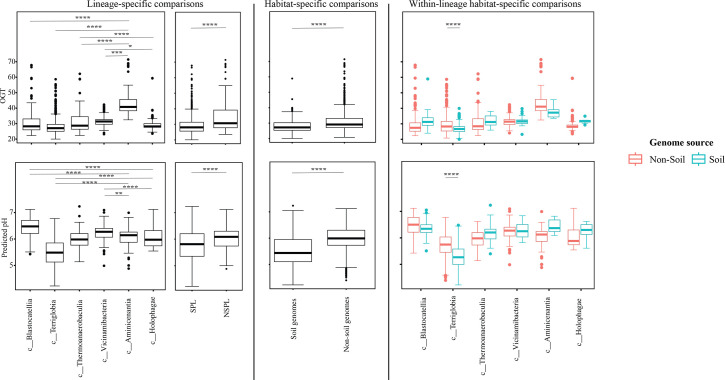
Box plots for the distribution of predicted physiological optima in the 1930 genomes of the 6 *Acidobacteriota* classes compared in this study. Table S4 shows the actual data used to construct these plots. Features shown are predicted OGT (top row) and predicted optimal pH (bottom row). Results for two-tailed ANOVA followed by Tukey for pairwise comparisons are shown on top of the box plots only for significant comparisons. *, 0.01<*P*<0.05; **, *P*<0.01; ***, *P*<0.001; ****, *P*<0.0001; *****, *P*<0.00001. The first two columns show results for lineage-specific comparisons at the *Acidobacteriota* class level, as well as when combining *Acidobacteriota* SPLs (*Terriglobia*, *Vicinamibacteria*, *Thermoanaerobaculia* and *Blastocatellia*) versus NSPLs (*Holophagae* and *Aminicenantia*). The third column shows results for habitat-specific comparisons (genomes originating from soil versus non-soil environments regardless of phylogeny). Results of within-lineage habitat-specific comparisons are shown in the fourth column, where genomes from soil origin are shown in cyan, while genomes from non-soil origin are shown in red. The exact *P*-values for all comparisons are shown in Table S5.

**Fig. 7.**
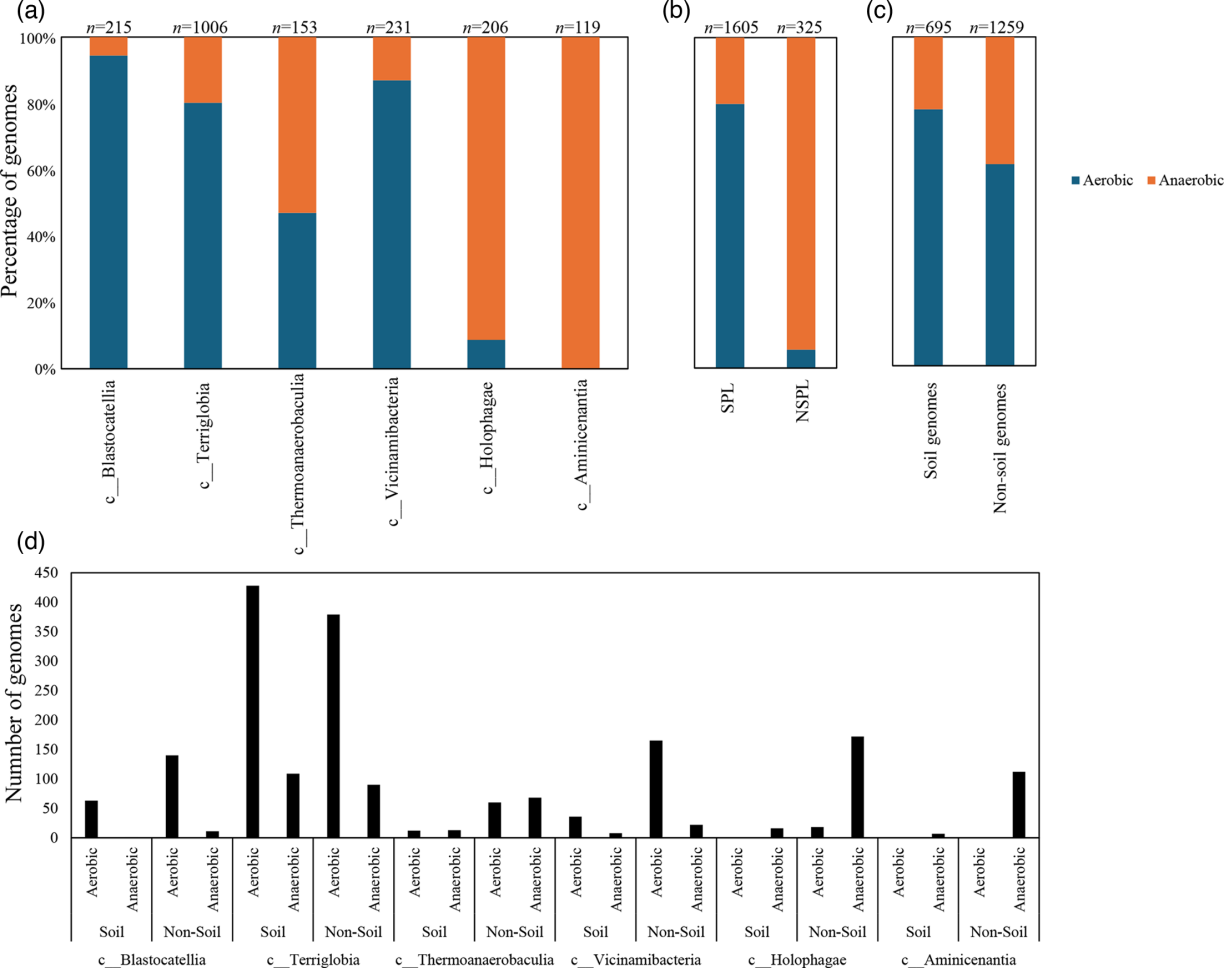
Bar plots for the distribution of the predicted aerobic (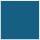) versus anaerobic (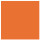) lifestyle in the 1930 genomes of the 6 *Acidobacteriota* classes compared in this study. Table S4 shows the actual data used to construct these plots. Results are shown by class (a), soil preference (SPLs versus NSPLs) (b), habitat from which the genome originated (c) and within lineage broken down by habitat (d). Number of genomes in each category is shown on top of the bar.

**Fig. 8.**
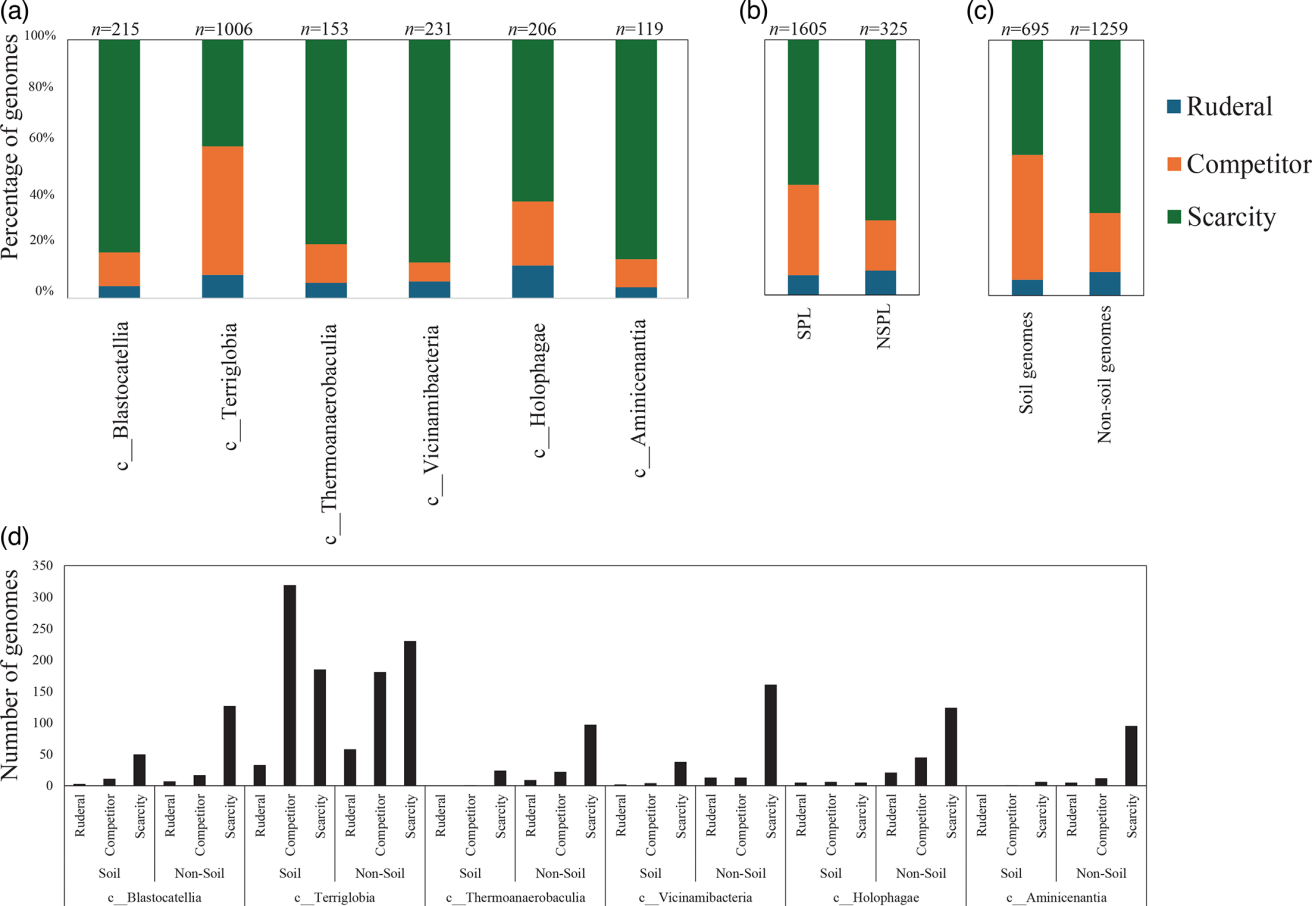
Bar plots for the distribution of the predicted life history strategy [ruderal (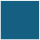), competitor (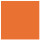) or scarcity (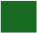)] in the 1930 genomes of the 6 *Acidobacteriota* classes compared in this study. Table S4 shows the actual data used to construct these plots. Results are shown by class (a), soil preference (SPLs versus NSPLs) (b), habitat from which the genome originated (c) and within lineage broken down by habitat (d). Number of genomes in each category is shown on top of the bar.

Table S4 shows the list of the 1930 *Acidobacteriota* genomes used in this study along with their genomic criteria compared (*n*=14). Comparative genomic analysis demonstrated a significant role played by phylogenetic affiliation in shaping all 14 examined criteria in *Acidobacteriota* genomes (F-test *P*-value <1.95×10^−7^, percentage lineage contribution 2.01–41.18%, Figs3, 4, 5, 6, 7  8, Table S5). Interestingly, a trend in some of these criteria was observed where SPLs were more similar to each other, compared to NSPLs. Specifically, SPLs possessed larger genomes (F-test *P*-value = 2×10^−16^, phylogeny percentage contribution=10.16%), higher GC content (F-test *P*-value = 2×10^−16^, phylogeny percentage contribution = 41.18%), lower gene density (F-test *P*-value = 2×10^−16^, phylogeny percentage contribution=10.21%), shorter average gene length (F-test *P*-value = 2×10^−16^, phylogeny percentage contribution=4.14%) and a higher number of protein-coding genes (F-test *P*-value = 2×10^−16^, phylogeny percentage contribution=11.06%) (Fig. 3), as well as a significantly larger number of viral contigs (F-test *P*-value = 2×10^−16^, phylogeny percentage contribution=5.21%) (Fig. 4) and a significantly expanded repertoire of CAZymes (F-test *P*-value = 2×10^−16^, phylogeny percentage contribution=28.63%), peptidases (F-test *P*-value = 2×10^−16^, phylogeny percentage contribution = 12.98%) and BGCs (F-test *P*-value = 7.4×10^−13^, phylogeny percentage contribution = 3.35%) (Fig. 5) and a higher proportion of genes with predicted aerobic preference (F-test *P*-value = 2×10^−16^, phylogeny percentage contribution=36.09%) (Fig. 7). For the remaining criteria, while phylogeny played a significant role in shaping the *Acidobacteriota* genome examined, the SPL/NSPL dichotomy across classes was not observed. For example, while phylogeny had a significant effect on predicted OGT (F-test *P*-value = 2×10^−16^, phylogeny percentage contribution=26.04%), and in general SPLs were predicted to have lower optimal OGT (Fig. 6), this pattern was mainly due to the higher predicted OGT for the *Aminicenantia* genomes, while *Holophagae* genomes were predicted to have lower OGT than genomes from SPLs (Fig. 6). Similarly, while phylogeny had a significant effect on predicted optimal growth pH (F-test *P*-value = 2×10^−16^, phylogeny percentage contribution=21.13%), this pattern was mainly due to the lower predicted pH for *Terriglobia* genomes, while *Blastocatellia* and *Vicinamibacteria* genomes were predicted to have higher pH than genomes from NSPLs. Finally, for life history strategies (ruderal, competitor and scarcity), phylogeny played a significant role in shaping the predicted life history strategy (F-test *P*-value = 1.95×10^−7^, phylogeny percentage contribution=2.01%), and in general, SPLs were predicted to have more competitor strategy. However, this pattern was mainly due to the higher number of predicted competitors for *Terriglobia* genomes, while other SPLs showed no significant difference in terms of the distribution of predicted life history strategies from the two NSPLs (Fig. 8).

On the other hand, habitat-specific, but lineage-agnostic, comparisons (i.e. comparing genomes originating from soil to those originating from non-soil sources, regardless of their phylogenetic affiliation) identified habitat-specific strong significant differences in only 1/14 criteria (F-test *P*-value <10^−5^), with genomes from soil predicted to have lower GC percentages. Weak but significant differences (F-test *P*-value <10^−5^< *P*<0.05) were observed between soil-originating versus non-soil-originating genomes for the number of predicted BGCs and CAZymes and the predicted OGT and pH (Table S5).

While the habitat from which the genome was obtained played a role in shaping genomes in 1/14 criteria, a much higher contribution was from the lineage (41.18% for lineage contribution, as opposed to only 0.63% for the habitat) (Table S5). To further examine differences on a finer scale, we compared within-lineage genomes originating from soil versus non-soil habitats. The SPLs *Terriglobia*, *Vicinamibacteria*, *Blastocatellia* and *Thermoanaerobaculia* showed more within-lineage significant differences between genomes originating from soil versus non-soil habitats with respectively 9, 4, 2 and 1 genomic features identified as within-lineage habitat specific. These included lower coding density, shorter genes, lower GC percentage, higher number of protein-coding genes, more predicted peptidases, less predicted viral contigs, lower predicted OGT, lower predicted pH and more predicted competitor lifestyle in *Terriglobia* soil genomes compared to non-soil genomes, larger genomes, shorter genes, more predicted peptidases and CAZymes in *Vicinamibacteria* soil genomes compared to non-soil genomes, shorter genes and lower coding density in *Blastocatellia* soil genomes compared to non-soil genomes and shorter genes in *Thermoanaerobaculia* soil genomes compared to non-soil genomes (Table S5). On the other hand, NSPLs showed less within-lineage significant differences between genomes originating for Kruskal–Wallis soil versus non-soil habitats with only one genomic feature (GC percentage) identified as within-lineage habitat specific for class *Aminicenantia* (with soil *Aminicenantia* genomes having higher GC percentage than non-soil genomes) (Table S5).

### Comparative genomic analysis identifies more lineage-specific than habitat-specific metabolic differences

We identify 74 features where SPLs differ significantly from NSPLs (Table S6). These features were mainly distributed among catabolic and anabolic pathways. On the catabolic front, genomes from SPLs encoded significantly higher O_2_ respiration genes, as well as dissimilatory sulphate reduction genes (Table S6). On the other hand, genomes from NSPLs encoded significantly higher nitrate reduction to ammonium and dissimilatory nitrate reduction genes. Fermentation pathways were also differentially abundant in genomes from SPLs versus NSPLs. Hydrogenases belonging to fermentative hydrogenogenic classes, as well as hydrogenotrophic classes were significantly encoded in NSPL genomes.

Further, SPL genomes were enriched in some central metabolic pathways that are usually associated with the higher O_2_ tension conditions in soil (Table S6) and in the degradation of several sugars/sugar acids, nt and aas. Of interest is the pathway of hydroxyproline degradation (an aa rich in plant cell wall proteins and glycoproteins and so is assumed to be abundant in soil constituting a rich source of C and N for soil bacteria [51]) (Table S6).

On the anabolic side, SPL genomes encoded a higher biosynthetic capacity, where biosynthesis of seven aas and eight cofactors were significantly enriched in genomes from the four SPLs (Kruskal–Wallis statistic *P*-value <1e−5). On the other hand, the biosynthesis of only one aa and two cofactors was enriched in genomes from the two NSPLs.

On the other hand, habitat-specific comparisons identified only 26 features where genomes from soil differ significantly from genomes from non-soil sources (Table S6). The majority of these features (22 out of 26) were identified as significantly affected by phylogeny, with only 4 features (fatty acid beta-oxidation pathway genes, N_2_ fixation genes, high-affinity O_2_ respiration using cytochrome oxidase cbb3 and molybdenum cofactor biosynthesis) identified as habitat specific (but not lineage specific). Statistical analysis of features where both phylogeny and habitat were significant (*n*=22) showed lineage to be more important (lineage percentage contribution: 0.87–25.48%; average, 10.41%; median, 6.96%), with habitat contributing <46.42% (average, 6.45%; median, 5.91%). Of the 22 features where both phylogeny and habitat were significant, 18 followed the same trend (where the feature was higher/lower in SPLs as well as genomes sourced from soil regardless of their lineage), with only four features with a divergent trend (Table S6).

## Discussion

Here, we examined *Acidobacteriota* global distribution patterns using a metagenomic read mapping approach and identified salient differences in genomic characteristics and metabolic capacities across lineages and habitats in the *Acidobacteriota*. Four classes (*Blastocatellia*, *Terriglobia*, *Thermoanaerobaculia* and *Vicinamibacteria*) were shown to have a clear preference to soil over non-soil habitats, based on their high soil ubiquity (encountered in >75% of soil datasets), soil preference (ratio of occurrence in datasets from soil versus non-soil datasets >4) and higher relative abundance in soil datasets compared to non-soil datasets (Fig. 2, Table S2). These four classes encompass many *Acidobacteriota* taxa previously isolated from soil or shown to occur in high abundance in soil in amplicon-based surveys. More importantly, we identify multiple additional poorly characterized yet-uncultured lineages that are ubiquitous and show high preference and high relative abundance in soil datasets (Fig. 2, Table S2). These include the uncultured families UBA7541 (subgroup 2) and SbA1 in *Terriglobia* and UBA2999 in *Vicinamibacteria*. Most of these families are defined based on MAGs recovered from metagenomic studies and are not currently recognized in amplicon- and isolation-based classification. The recognition of the prevalence of such lineages should spur efforts towards more detailed omics-based characterization and assessment of their importance and potential role in elemental cycling and contribution to ecosystem functioning in soil, as well as stimulate endeavours towards obtaining them in pure cultures.

Organisms encountered in multiple niches are referred to as generalists, while those restricted to a single habitat are specialists. Studies usually utilize occurrence patterns as an empirical measurement of a generalist versus specialist pattern either within a target habitat [52], through a habitat transition gradient, or on a global scale [53]. Assignment of microbial taxa as generalists or specialists could be affected by the level of phylogenetic resolution employed, number of datasets examined, sequencing depth of datasets and detection threshold employed. Prior research provided some insights on such patterns in the *Acidobacteriota*. For example, a study examining a large collection of datasets from farmland soils suggested that *Acidobacteriota* harboured a larger fraction of generalists than specialists at the species operational taxonomic unit level [52]. On the other hand, studies assessing *Acidobacteriota* generalist–specialist patterns on a global scale are quite sparse. A recent analysis, defining specialists and generalists using the level of community similarity between datasets where a specific lineage is encountered (with high community similarity indicating a specialist pattern and low community similarity indicating a generalist pattern) as a substitute for occurrence patterns, concluded that *Acidobacteriota* encompassed a high proportion of specialized genera [53].

Our analysis of *Acidobacteriota* distribution patterns strongly suggests that a habitat-generalist rather than a habitat-specialist pattern is more common in the *Acidobacteriota* (Table S3). Using two datasets (publicly available metagenomes via Sandpiper and publicly available genomes in GTDB), multiple criteria (with and without exclusion of rare taxa, with and without exclusion of taxa with less than five genomes) and taxonomic thresholds (class, order and family), we estimate that 73.3–91.7% of classes, 61.5–86.49% of orders and 52.9–87.04=% of families are habitat generalists, with documented ability to inhabit multiple environments. Our results are conducted at the order and family levels and hence do not rule out the possibility of genus-, species- or strain-level specialization. For example, a class could collectively be considered a generalist, but individual genera within the class could exhibit a finer pattern of habitat specialization.

We attribute the prevalence of habitat-generalist over habitat-specialist pattern in the *Acidobacteriota* to two possible reasons: the nature of its preferred habitat(s) and the genomic features and metabolic capacities of its members. Broadly, habitats that are extreme, restricted and drastically different from their surroundings favour specialists, e.g. chemolithotrophic hyperthermophiles in hydrothermal vents [54], strict halophilic archaea in hypersaline ecosystems [55] and anaerobic gut fungi in the herbivorous alimentary tracts [56]. On the other hand, generalists thrive across more temperate, less restricted and more complex habitats. Broadly, the habitats where *Acidobacteriota* appears to thrive, e.g. soil and freshwater, are temperate, with fluctuating temperature, pH and salinity, as well as a complex variable influx of substrates. Such conditions allow for generalists, organisms usually exhibiting a wider arsenal of substrate utilization capacities and response to environmental fluctuations, to thrive as integral components of a complex habitat.

Regarding metabolic abilities, habitat-specialists’ genomes are usually streamlined and either mediate a specific function or encode exceptional capacity to survive and adapt to a unique ecosystem [57]. Habitat-generalists’ genomes, on the other hand, are more metabolically versatile to enable adaptation, e.g. *Pseudomonas* [58], *Burkholderia* [59] thriving under many substrates and *Shewanella* thriving under many electron acceptors [60]. Within the *Acidobacteriota*, genomic analysis identified moderate to large genomes and suggested a general prevalence of heterotrophy and lack of auxotrophies. Our ML-based analysis suggested the prevalence of moderate predicted OGT and pH, as well as variable predicted oxygen preferences, all of which would explain the prevalence of habitat-generalist patterns in the phylum.

Prior genomic analysis of members of the *Acidobacteriota* has been conducted on pure culture isolates [14,61,63] or assembled MAGs [4,22, 31, 64, 65]. These efforts have yielded valuable insights into the salient genomic features of various members of the phylum. Our analysis aimed for a broader and more comprehensive view of *Acidobacteriota* genomes via a global comparative analysis approach. In addition to expanding on known capacities within the phylum, we aimed to identify variations in genomic features, physiological optima and metabolic capacities within members of the *Acidobacteriota* and to determine whether the observed differences are habitat specific (e.g. present in all genomes of soil *Acidobacteriota* regardless of the lineage they belong to), lineage specific (e.g. present in all genomes of a lineage regardless of the environmental source) or a combination of both. Our results suggest that lineage is more important than habitat in describing the observed differences. Within general genomic features and physiological optima prediction, lineage significantly affected all 14 criteria compared (F-test *P*-value <2.6×10^−10^), while habitat only significantly affected 1 criterion, and the interaction between the two only significantly affected 5 criteria (coding density, GC percentage, number of predicted peptidases, predicted OGT and predicted optimal growth pH) (Table S5, Figs3, 5  6). In all these comparisons, lineage explained 2.01–41.18% of the variability, while habitat only explained 0.63%. Detailed analysis of metabolic capacities identified 74 functions that were significantly different between SPLs and NSPLs, while habitat only significantly affected 26 functions (22 of which were also significantly affected by lineage). In all these comparisons, lineage explained 0.87–42.47% of the variability, while habitat contribution never exceeded 16.42%. Indeed, many of the metabolic features previously shown to underpin the success of *Acidobacteriota* in soil were found to be lineage specific (significantly encountered more in SPL genomes), rather than habitat specific (present in all soil *Acidobacteriota* regardless of their phylogeny). For example, genes encoding CAZymes and BGCs were significantly higher in SPL genomes, but these numbers did not significantly vary in genomes derived from soil versus genomes derived from non-soil environments (*P*-value >6×10^−3^). However, while clearly lineage plays a more important role, some habitat-specific features are of note. Soil as a habitat appears to select for genomes with lower GC percentage and for organisms with lower predicted OGT and lower predicted pH.

Finally, the observed prevalence of a generalist pattern and higher importance of lineage compared to habitat in shaping *Acidobacteriota* genomes strongly suggest a ready cross-colonization of *Acidobacteriota* across major habitats. Under this scenario, while the differential preference of certain *Acidobacteriota* lineages to specific habitat exists, the presence of a ready reservoir, albeit in minor quantities, in other environments would allow ready cross-colonization when needed, e.g. as a mechanism for repopulation post-disturbances (e.g. fire and extensive pollution), or during the process of pedogenesis (soil formation) (Table S2). Nevertheless, our results also advocate for a role played by the environment, specifically the soil studied here, in shaping *Acidobacteriota* genomes post-acquisition. Particularly, a lower GC percentage and lower predicted OGT and pH would allow organisms to survive better in the soil ecosystem. Genomes from the soil also encoded pathways beneficial to surviving in such ecosystem, e.g. N_2_ fixation and purine degradation to urea (Table S6).

In conclusion, our results identify clear soil preferences for four classes (*Terriglobia*, *Vicinamibacteria*, *Blastocatellia* and *Thermoanaerobaculia*) of *Acidobacteriota*. We also demonstrate that such preferences are driven not only by taxa previously recognized as prominent soil dwellers in prior isolation and amplicon efforts but also by multiple yet-uncultured orders and families in these four classes that are ubiquitous and abundant yet poorly characterized. Further, our analysis indicates that despite the observed preference patterns, most *Acidobacteriota* classes, orders and families are habitat generalists rather than specialists. Also, our global comparative genomic analysis provides new insights into the genomic features, predicted physiological optima and metabolic repertoires of members of the *Acidobacteriota* and disentangles the role played by phylogeny versus habitat in shaping *Acidobacteriota* genomic and predicted metabolic features.

## supplementary material

10.1099/mgen.0.001344 Table S1.

## Abbreviations

BGC: biosynthetic gene cluster
GTDB: Genome Taxonomy Database
KOs: KEGG Orthologies
MAG: metagenome-assembled genome
ML: machine learning
NSPL: non-soil preferring lineage
OGT: optimal growth temperature
SAG: single amplified genome
SPL: soil-preferring lineage
