# Supplementary material for: An ecological-evolutionary perspective on the genomic diversity and habitat preferences of the Acidobacteriota
Source: Microb Genom. 2025 Jan 29;11(1):001344. doi: 10.1099/mgen.0.001344 (PMC11778308; doi:10.1099/mgen.0.001344)
Supplement: Table S1. [file mgen-11-01344-s001.pdf]

Supplementary material for

An ecological-evolutionary perspective on the genomic diversity and habitat preferences of the Acidobacteriota

Ella McReynolds, Mostafa S. Elshahed, and Noha H. Youssef*

Department of Microbiology and Molecular Genetics, Oklahoma State University, Stillwater, OK, USA

Supplementary tables.

Table S1. Acidobacteriota classification based on GTDB, and the corresponding taxonomy in Silva, as well as the subgroup classification by Barns et al. [1] and Dedysh and Yilmaz [2].

Table S2. Ecological distribution of Acidobacteriota in the 248,559 metagenomic studies in Sandpiper. Results are shown at the phylum level, all 14 classes, as well as their constituting orders and families. For each taxonomy level, results are shown for ubiquity (measured as the percentage occurrence in datasets from engineered, freshwater, host-associated, marine, non-marine saline and alkaline, terrestrial non-soil, as well as soil datasets). Percentage occurrence in all non-soil datasets is also shown. The ratio between percentage occurrence in soil versus non-soil datasets is calculated as a measure for soil-preference. In addition to occurrence, values of percentage abundance in each of the 7 habitat classifications, as well as percentage abundance in all non-soil datasets are also shown for each of the taxonomy levels. Finally, the ratio between percentage abundance in soil versus non-soil datasets is also shown.

Table S3. Numbers and percentages of habitat-generalist taxa in the Acidobacteriota at different taxonomic levels and using several delineation criteria.

Table S4. Genomes compared in this study, their Acidobacteriota class, lineage preference to soil (Soil-preferring lineage, SPL vs non-soil preferring lineage, NSPL), habitat from which the genome was recovered, and various genomic features compared.

Table S5. ANOVA results for the effect of Acidobacteriota class, the habitat from which the genome originated, and the interaction between the two on various genomic features. Results of Tukey post-hoc pairwise comparisons are shown for the significant factors.

Table S6. Logistic regression results for the effect of Acidobacteria lineage (SPL versus NSPL) and the habitat from which the genome originated on various metabolic functions annotated in the genomes.

Table S1. Acidobacteriota classification based on GTDB, and the corresponding taxonomy in Silva, as well as the subgroup classification by Barns et al. [1] and Dedysh and Yilmaz [2].

GTDB classification of Phylum Acidobacteriota				Silva classification of the top hit (>94%)	Barns Classification	Dedysh and Yilmaz classification
Class	Order	Family	Genus			
c__Aminicenantia	o__Aminicenantales	f__Aminicenantaceae	g__Aminicenans	Bacteria;Acidobacteriota;Aminicenantia;Aminicenantales;Aminicenantes bacterium JGI OTU-2	NA	NA
			g__G020344415	Bacteria;Acidobacteriota;Aminicenantia;Aminicenantales;uncultured bacterium		
			g__G020348385	No 16S rRNA gene		
			g__JADHWO01	No 16S rRNA gene		
			g__JAFGMO01	Bacteria;Acidobacteriota;Aminicenantia;Aminicenantales;uncultured bacterium		
			g__JAGLWV01	No 16S rRNA gene		
			g__JAGXKI01	No/low similarity in Silva		
			g__JAHIPL01	Bacteria;Acidobacteriota;Aminicenantia;Aminicenantales;uncultured Aminicenantes bacterium		
			g__Kmv29	Bacteria;Acidobacteriota;Aminicenantia;Aminicenantales;uncultured bacterium		
			g__SOIA01	No 16S rRNA gene		
			g__SOIV01	Bacteria;Acidobacteriota;Aminicenantia;Aminicenantales;uncultured bacterium		
			g__WJMT01	No/low similarity in Silva		
			g__WTAW01	Bacteria;Acidobacteriota;Aminicenantia;Aminicenantales;uncultured bacterium		
			g__WVZL01	Bacteria;Acidobacteriota;Aminicenantia;Aminicenantales;uncultured bacterium		
		f__JAFGAV01	g__JAFGAV01	No/low similarity in Silva		
		f__JAFGBC01	g__JAFGBC01	No/low similarity in Silva		
		f__JdFR-80	g__JdFR-80	Bacteria;Acidobacteriota;Aminicenantia;Aminicenantales;uncultured bacterium		
		f__RBG-16-66-30	g__JALHVH01	Bacteria;Acidobacteriota;Aminicenantia;Aminicenantales;uncultured bacterium		
			g__RBG-16-66-30	Bacteria;Acidobacteriota;Aminicenantia;Aminicenantales;metagenome		
		f__Saccharicenantaceae	g__Saccharicenans	Bacteria;Acidobacteriota;Aminicenantia;Aminicenantales;uncultured bacterium		
		f__UBA4085	g__FEN-413	Bacteria;Acidobacteriota;Aminicenantia;Aminicenantales;uncultured bacterium		
			g__JAAZPG01	Bacteria;Acidobacteriota;Aminicenantia;Aminicenantales;metagenome		
			g__JALHTW01	No 16S rRNA gene		
			g__UBA4085	No/low similarity in Silva		
		f__UBA8522	g__UBA8522	Bacteria;Acidobacteriota;Aminicenantia;Aminicenantales;uncultured bacterium		
	o__JAMOAD01	f__JAMOAD01	g__JAMOAD01	No 16S rRNA gene		
	o__JdFR-78	f__JdFR-78	g__JdFR-78	No/low similarity in Silva		
	o__S016-38	f__S016-38	g__S016-38	Bacteria;Acidobacteriota;Aminicenantia;Aminicenantales;uncultured Aminicenantes bacterium		
	o__UBA2199	f__UBA2199	g__JAGLQA01	No 16S rRNA gene		
			g__JAHELQ01	No 16S rRNA gene		
			g__JAKAGC01	No/low similarity in Silva		
			g__UBA2199	Bacteria;Acidobacteriota;Aminicenantia;Aminicenantales;uncultured bacterium		
			g__UBA647	Bacteria;Acidobacteriota;Aminicenantia;Aminicenantales;uncultured bacterium		
			g__UBA876	Bacteria;Acidobacteriota;Aminicenantia;Aminicenantales;uncultured bacterium		
c__B3-B38	o__B3-B38	f__B3-B38	g__Guanabacterium	Bacteria;Acidobacteriota;ODP1230B23.02;uncultured bacterium	NA	NA
		f__JAGGZT01	g__JAGGZT01	No/low similarity in Silva		
	o__JAFGBY01	f__JAFGBY01	g__JAFGBY01	No 16S rRNA gene		
c__Blastocatellia	o__Chloracidobacteriales	f__Chloracidobacteriaceae	g__Chloracidobacterium	Bacteria;Acidobacteriota;Blastocatellia;Chloracidobacteriales;Chloracidobacteriaceae;Chloracidobacterium;Chloracidobacterium thermophilum	Subgroup 4	Class 2
			g__JAAFHI01	No 16S rRNA gene		
			g__JACQXX01	No/low similarity in Silva		
			g__JAIBAN01	Bacteria;Acidobacteriota;Blastocatellia;11-24;uncultured bacterium		
		f__JADJPF01	g__JADJPF01	Bacteria;Acidobacteriota;Blastocatellia;11-24;uncultured bacterium		
			g__JAEUQL01	No 16S rRNA gene		
			g__JANWYG01	No 16S rRNA gene		
	o__HR10	f__HR10	g__HR10	Bacteria;Acidobacteriota;Blastocatellia;11-24;uncultured Acidobacteria bacterium		
		f__J050	g__J050	No 16S rRNA gene		
			g__JANWZE01	No/low similarity in Silva		
		f__JACQTD01	g__JACQTD01	No/low similarity in Silva		
	o__Pyrinomonadales	f__Pyrinomonadaceae	g__3-1-20CM-3-53-8	Bacteria;Acidobacteriota;Blastocatellia;Pyrinomonadales;Pyrinomonadaceae;RB41;uncultured bacterium		
			g__Aridibacter	Bacteria;Acidobacteriota;Blastocatellia;Blastocatellales;Blastocatellaceae;Blastocatella;uncultured Acidobacteria bacterium		
			g__GCA-002050365	Bacteria;Acidobacteriota;Blastocatellia;Blastocatellales;Blastocatellaceae;Blastocatella;Acidobacteria bacterium 28-9		
			g__J014	Bacteria;Acidobacteriota;Blastocatellia;Chloracidobacteriales;Chloracidobacteriaceae;Chloracidobacterium;uncultured Acidobacteria bacterium		
			g__JACCRA01	No 16S rRNA gene		
			g__JACCTF01	No 16S rRNA gene		
			g__JACCYN01	No 16S rRNA gene		
			g__JACDEG01	No 16S rRNA gene		
			g__JACMLC01	Bacteria;Acidobacteriota;Blastocatellia;Pyrinomonadales;Pyrinomonadaceae;RB41;uncultured bacterium		
			g__JACVRN01	No 16S rRNA gene		
			g__JAFDVJ01	Bacteria;Acidobacteriota;Blastocatellia;Blastocatellales;Blastocatellaceae;Aridibacter;uncultured bacterium		
			g__JAIBCC01	Bacteria;Acidobacteriota;Blastocatellia;Blastocatellales;Blastocatellaceae;Blastocatella;uncultured bacterium		
			g__JAIVGV01	Bacteria;Acidobacteriota;Blastocatellia;Pyrinomonadales;Pyrinomonadaceae;RB41;uncultured bacterium		
			g__JAIVJG01	Bacteria;Acidobacteriota;Blastocatellia;Pyrinomonadales;Pyrinomonadaceae;RB41;uncultured bacterium		
			g__JAIVJM01	No/low similarity in Silva		
			g__JAIVJZ01	Bacteria;Acidobacteriota;Blastocatellia;Pyrinomonadales;Pyrinomonadaceae;RB41;uncultured Acidobacteria bacterium		
			g__JAJQRF01	Bacteria;Acidobacteriota;Blastocatellia;Blastocatellales;Blastocatellaceae;Blastocatella;uncultured bacterium		
			g__JANDLT01	Bacteria;Acidobacteriota;Blastocatellia;Pyrinomonadales;Pyrinomonadaceae;RB41;uncultured bacterium		
			g__OLB17	No/low similarity in Silva		
			g__PSRF01	Bacteria;Acidobacteriota;Blastocatellia;Pyrinomonadales;Pyrinomonadaceae;RB41;uncultivated soil bacterium clone S023		
			g__Pyrinomonas	Bacteria;Acidobacteriota;Blastocatellia;Pyrinomonadales;Pyrinomonadaceae;Pyrinomonas;uncultured bacterium		
			g__QHXM01	Bacteria;Acidobacteriota;Blastocatellia;Pyrinomonadales;Pyrinomonadaceae;RB41;uncultured Acidobacteria bacterium		
			g__QHXN01	Bacteria;Acidobacteriota;Blastocatellia;Pyrinomonadales;Pyrinomonadaceae;RB41;Arenimicrobium luteum		
			g__QHXQ01	No 16S rRNA gene		
			g__SXVE01	No 16S rRNA gene		
			g__UBA11740	Bacteria;Acidobacteriota;Acidobacteriae;Acidobacteriales;uncultured;uncultured bacterium		
	o__RBC074	f__RBC074	g__CAIOZW01	No 16S rRNA gene		
			g__JACPYM01	Bacteria;Acidobacteriota;Blastocatellia;11-24;uncultured bacterium		
			g__JADJLO01	Bacteria;Acidobacteriota;Blastocatellia;11-24;metagenome		
			g__JADJXM01	Bacteria;Acidobacteriota;Blastocatellia;11-24;uncultured bacterium		
			g__JAFDVL01	No 16S rRNA gene		
			g__JAHBSF01	Bacteria;Acidobacteriota;Blastocatellia;11-24;uncultured Acidobacteria bacterium		
			g__JAJVID01	Bacteria;Acidobacteriota;Blastocatellia;11-24;uncultured Acidobacteria bacterium		
			g__JAKEHZ01	No 16S rRNA gene		
			g__RBC074	Bacteria;Acidobacteriota;Blastocatellia;11-24;uncultured Acidobacteria bacterium		
	o__UBA7656	f__JADIYM01	g__JADIYM01	Bacteria;Acidobacteriota;Blastocatellia;DS-100;uncultured bacterium		
		f__UBA7656	g__JACQDB01	Bacteria;Acidobacteriota;Blastocatellia;11-24;uncultured bacterium		
			g__JADGNW01	No 16S rRNA gene		
			g__JAKEHD01	Bacteria;Acidobacteriota;Blastocatellia;11-24;uncultured Acidobacteria bacterium		
			g__QHVH01	Bacteria;Acidobacteriota;Blastocatellia;11-24;uncultured bacterium		
			g__UBA7656	Bacteria;Acidobacteriota;Blastocatellia;11-24;uncultured bacterium		
c__CAIWXX01	o__CAIWXX01	f__CAIWXX01	g__CAIWXX01	No/low similarity in Silva	NA	NA
		f__CAIWXX01	g__JACPPM01	No 16S rRNA gene		
c__Holophagae	o__Acanthopleuribacterales	f__Acanthopleuribacteraceae	g__Acanthopleuribacter	Bacteria;Acidobacteriota;Holophagae;Acanthopleuribacterales;Acanthopleuribacteraceae;Acanthopleuribacter;Acanthopleuribacter pedis	Subgroup 8	Class 7
			g__GCA-2747255	Bacteria;Acidobacteriota;Holophagae;Acanthopleuribacterales;Acanthopleuribacteraceae;Acanthopleuribacter;uncultured bacterium		
			g__JACJWC01	Bacteria;Acidobacteriota;Holophagae;Acanthopleuribacterales;Acanthopleuribacteraceae;Acanthopleuribacter;uncultured Acidobacteria bacterium		
			g__JAGQSC01	No 16S rRNA gene		
			g__Sulfidibacter	No/low similarity in Silva		
	o__Holophagales	f__Holophagaceae	g__Geothrix	Bacteria;Acidobacteriota;Holophagae;Holophagales;Holophagaceae;Geothrix;Geothrix fermentans DSM 14018		
			g__Holophaga	Bacteria;Acidobacteriota;Holophagae;Holophagales;Holophagaceae;Holophaga;Holophaga foetida		
			g__JACQZU01	No 16S rRNA gene		
			g__JADJVL01	Bacteria;Acidobacteriota;Holophagae;Holophagales;Holophagaceae;Geothrix;uncultured bacterium		
			g__JAFDVN01	No 16S rRNA gene		
			g__JAJYBX01	No 16S rRNA gene		
			g__JAJZQW01	No 16S rRNA gene		
			g__JAKAMQ01	No 16S rRNA gene		
			g__UBA12189	Bacteria;Acidobacteriota;Holophagae;Holophagales;Holophagaceae;marine group;marine metagenome		
			g__WRHW01	Bacteria;Acidobacteriota;Holophagae;Holophagales;Holophagaceae;uncultured;uncultured bacterium		
	o__Thermotomaculales	f__Thermotomaculaceae	g__Thermotomaculum	Bacteria;Acidobacteriota;Holophagae;Acanthopleuribacterales;Acanthopleuribacteraceae;Thermotomaculum;Thermotomaculum hydrothermale		
			g__UM-FILTER-49-7	Bacteria;Acidobacteriota;Holophagae;Acanthopleuribacterales;Acanthopleuribacteraceae;Thermotomaculum;uncultured Acidobacteria bacterium		
c__Polarisedimenticolia	o__Gp22-AA2	f__AA152	g__AA152	Bacteria;Acidobacteriota;Subgroup 22;uncultured bacterium	Subgroup 22	
		f__Gp22-AA2	g__Gp22-AA3	Bacteria;Acidobacteriota;Subgroup 22;uncultured Acidobacteriales bacterium		
			g__JACQNT01	No 16S rRNA gene		
			g__JAGYID01	Bacteria;Acidobacteriota;Subgroup 22;uncultured bacterium		
			g__QHXZ01	Bacteria;Acidobacteriota;Subgroup 22;uncultured bacterium		
	o__J045	f__J045	g__G020350085	No 16S rRNA gene		
			g__J045	No/low similarity in Silva		
			g__J065	No/low similarity in Silva		
			g__JAGOJY01	No/low similarity in Silva		
			g__JAJFTU01	Bacteria;Acidobacteriota;Subgroup 22;uncultured bacterium		
			g__JANTFM01	No 16S rRNA gene		
	o__JACPZX01	f__JACPZX01	g__JACPZX01	No/low similarity in Silva		
	o__Polarisedimenticolales	f__Polarisedimenticolaceae	g__JAAELQ01	No/low similarity in Silva		
			g__JAIQFR01	Bacteria;Acidobacteriota;Subgroup 22;uncultured bacterium		
			g__Mor1	Bacteria;Acidobacteriota;Subgroup 22;Acidobacteria bacterium Mor1		
			g__Polarisedimenticola	Bacteria;Acidobacteriota;Subgroup 22;uncultured bacterium		
			g__WVWW01	No 16S rRNA gene		
	o__QHYA01	f__QHYA01	g__QHYA01	Bacteria;Acidobacteriota;Subgroup 22;uncultured bacterium		
	o__SMYD01	f__SMYD01	g__SMYD01	Bacteria;Acidobacteriota;Subgroup 22;uncultured deep-sea bacterium		
c__HRBIN11	o__HRBIN11	f__HRBIN11	g__HRBIN11	Bacteria;Acidobacteriota;GBS-L1-B05;uncultured bacterium	NA	NA
	o__JAKEFK01	f__JAKEFK01	g__JAKEFK01	No 16S rRNA gene	NA	NA
	o__MFGW01	f__MFGW01	g__MFGW01	Bacteria;Acidobacteriota;Candidatus Fischerbacteria bacterium RBG_13_37_8	Subgroup 25	Class 6-1
c__Terriglobia	o__2-12-FULL-54-10	f__2-12-FULL-54-10	g__2-02-FULL-61-28	No 16S rRNA gene	Subgroup 15	Class 1-1
			g__2-12-FULL-54-10	Bacteria;Acidobacteriota;Acidobacteriae;Subgroup 15;		

		g__JACPPJ01	No 16S rRNA gene	
		g__JACQGE01	No 16S rRNA gene	
		g__JADFGZ01	No 16S rRNA gene	
		g__SHUM01	No/low similarity in Silva	
o__20CM-2-55-15	f__20CM-2-55-15	g__20CM-2-55-15	Bacteria;Acidobacteriota;Subgroup 5;uncultured bacterium	Subgroup 5
		g__CAMBFR01	No 16S rRNA gene	
		g__JAHFTY01	No 16S rRNA gene	
		g__JAJEDW01	Bacteria;Acidobacteriota;Subgroup 5;uncultured bacterium	
o__Acidoferrales	f__2-02-FULL-67-57	g__2-02-FULL-67-57	No 16S rRNA gene	NA
		g__JACPRL01	No 16S rRNA gene	NA
		g__JACQTG01	No 16S rRNA gene	NA
		g__JACZYA01	Bacteria;Acidobacteriota;Acidobacteriae;Subgroup 2;uncultured bacterium	NA
	f__UBA7541	g__Acidoferrum	Bacteria;Acidobacteriota;Acidobacteriae;Subgroup 13;uncultured bacterium	Subgroup 2
		g__DSGC01	No/low similarity in Silva	
		g__JAAFGT01	Bacteria;Acidobacteriota;Acidobacteriae;Subgroup 2;uncultured bacterium	
		g__JACQDE01	Bacteria;Acidobacteriota;Acidobacteriae;Subgroup 2;uncultured bacterium	
		g__JAIQGC01	Bacteria;Acidobacteriota;Acidobacteriae;Subgroup 2;uncultured Acidobacteria bacterium	
		g__JAKASX01	No/low similarity in Silva	
		g__Palsa-147	Bacteria;Acidobacteriota;Acidobacteriae;Subgroup 2;uncultured bacterium	
		g__Palsa-189	Bacteria;Acidobacteriota;Acidobacteriae;Subgroup 2;uncultured bacterium	
		g__PALSA-278	Bacteria;Acidobacteriota;Acidobacteriae;Subgroup 2;uncultured bacterium	
		g__Palsa-295	Bacteria;Acidobacteriota;Acidobacteriae;Subgroup 2;uncultured bacterium	
		g__Palsa-360	Bacteria;Acidobacteriota;Acidobacteriae;Subgroup 2;uncultured bacterium	
		g__QHYP01	Bacteria;Acidobacteriota;Acidobacteriae;Subgroup 2;uncultured bacterium	
		g__QHYU01	Bacteria;Acidobacteriota;Acidobacteriae;Subgroup 2;uncultured bacterium	
o__Bryobacterales	f__Bryobacteraceae	g__Bog-105	Bacteria;Acidobacteriota;Acidobacteriae;Bryobacterales;Bryobacteraceae;Bryobacter;uncultured Acidobacteria bacterium	Subgroup 3
		g__Bog-113	No/low similarity in Silva	
		g__Bog-159	No/low similarity in Silva	
		g__BOG-224	Bacteria;Acidobacteriota;Acidobacteriae;Solibacterales;Solibacteraceae;Candidatus Solibacter;uncultured Acidobacteria bacterium	
		g__BOG-234	Bacteria;Acidobacteriota;Acidobacteriae;Solibacterales;Solibacteraceae;Candidatus Solibacter;uncultured Acidobacteria bacterium	
		g__Bog-375	Bacteria;Acidobacteriota;Acidobacteriae;Acidobacteriales;uncultured;uncultured bacterium	
		g__Bryobacter	Bacteria;Acidobacteriota;Acidobacteriae;Bryobacterales;Bryobacteraceae;Bryobacter;metagenome	
		g__CAADGY01	No 16S rRNA gene	
		g__CADEFT01	Bacteria;Acidobacteriota;Acidobacteriae;Bryobacterales;Bryobacteraceae;Bryobacter;metagenome	
		g__CAIRSA01	Bacteria;Acidobacteriota;Acidobacteriae;Bryobacterales;Bryobacteraceae;Bryobacter;uncultured bacterium	
		g__CAIVVT01	Bacteria;Proteobacteria;Gammaproteobacteria;Steroidobacterales;Steroidobacteraceae;uncultured;uncultured bacterium	
		g__CAJCIT01	Bacteria;Acidobacteriota;Acidobacteriae;Bryobacterales;Bryobacteraceae;Bryobacter;uncultured Acidobacteria bacterium	
		g__DOZZ01	Bacteria;Acidobacteriota;Acidobacteriae;Solibacterales;Solibacteraceae;Candidatus Solibacter;uncultured bacterium	
		g__DSOI01	No 16S rRNA gene	
		g__DTHT01	No 16S rRNA gene	
		g__DTKY01	No 16S rRNA gene	
		g__F-183	Bacteria;Acidobacteriota;Acidobacteriae;Bryobacterales;Bryobacteraceae;Bryobacter;uncultured bacterium	
		g__Fen-178	Bacteria;Acidobacteriota;Acidobacteriae;Solibacterales;Solibacteraceae;Candidatus Solibacter;uncultured bacterium	
		g__Fen-330	Bacteria;Acidobacteriota;Acidobacteriae;AKIW659;uncultured bacterium	
		g__Ga0077553	Bacteria;Acidobacteriota;Acidobacteriae;Paludibaculum;metagenome	
		g__JACADU01	No/low similarity in Silva	
		g__JACMLA01	No 16S rRNA gene	
		g__JACOTZ01	No 16S rRNA gene	
		g__JACPNO01	No 16S rRNA gene	
		g__JACQZT01	No 16S rRNA gene	
		g__JACTMJ01	Bacteria;Acidobacteriota;Acidobacteriae;Bryobacterales;Bryobacteraceae;Bryobacter;uncultured Acidobacteriaceae bacterium	
		g__JADGHK01	No 16S rRNA gene	
		g__JADGNR01	Bacteria;Acidobacteriota;Acidobacteriae;Solibacterales;Solibacteraceae;Candidatus Solibacter;uncultured bacterium	
		g__JADJWL01	Bacteria;Acidobacteriota;Acidobacteriae;Solibacterales;Solibacteraceae;Candidatus Solibacter;metagenome	
		g__JADLDI01	Bacteria;Acidobacteriota;Acidobacteriae;Bryobacterales;Bryobacteraceae;Bryobacter;uncultured bacterium	
		g__JADZAF01	Bacteria;Acidobacteriota;Acidobacteriae;Solibacterales;Solibacteraceae;Candidatus Solibacter;uncultured Acidobacteria bacterium	
		g__JADZCI01	Bacteria;Acidobacteriota;Acidobacteriae;Paludibaculum;uncultured Acidobacteria bacterium	
		g__JADZGD01	No 16S rRNA gene	
		g__JAEHDI01	No 16S rRNA gene	
		g__JAFLBB01	Bacteria;Acidobacteriota;Acidobacteriae;Bryobacterales;Bryobacteraceae;Bryobacter;metagenome	
		g__JAIEPW01	Bacteria;Acidobacteriota;Acidobacteriae;Bryobacterales;Bryobacteraceae;Bryobacter;uncultured bacterium	
		g__JAIMAR01	Bacteria;Chloroflexi;OLB14;uncultured bacterium	
		g__JAIQFL01	Bacteria;Acidobacteriota;Acidobacteriae;Solibacterales;Solibacteraceae;Candidatus Solibacter;uncultured bacterium	
		g__JAIQFS01	Bacteria;Acidobacteriota;Acidobacteriae;Solibacterales;Solibacteraceae;Candidatus Solibacter;uncultured bacterium	
		g__JAJFUV01	Bacteria;Acidobacteriota;Acidobacteriae;Bryobacterales;Bryobacteraceae;Bryobacter;metagenome	
		g__JAJPJX01	No 16S rRNA gene	
		g__JAJYKC01	Bacteria;Acidobacteriota;Acidobacteriae;Bryobacterales;Bryobacteraceae;Bryobacter;uncultured Acidobacteria bacterium	
		g__JAJYLS01	No 16S rRNA gene	
		g__JALHNI01	Bacteria;Acidobacteriota;Acidobacteriae;Bryobacterales;Bryobacteraceae;Bryobacter;metagenome	
		g__JAMCRM01	No 16S rRNA gene	
		g__JANWVO01	Bacteria;Acidobacteriota;Acidobacteriae;Paludibaculum;uncultured Acidobacteria bacterium	
		g__JANWXD01	No/low similarity in Silva	
		g__KBS-96	Bacteria;Acidobacteriota;Acidobacteriae;Bryobacterales;Bryobacteraceae;Bryobacter;Acidobacteriaceae bacterium KBS 96	
		g__OMOG01	No 16S rRNA gene	
		g__PALSA-129	Bacteria;Acidobacteriota;Acidobacteriae;Solibacterales;Solibacteraceae;Candidatus Solibacter;uncultured bacterium	
		g__Palsa-187	Bacteria;Acidobacteriota;Acidobacteriae;Bryobacterales;Bryobacteraceae;Bryobacter;metagenome	
		g__PALSA-243	No 16S rRNA gene	
		g__Palsa-89	Bacteria;Acidobacteriota;Acidobacteriae;Bryobacterales;Bryobacteraceae;Bryobacter;uncultured Acidobacteria bacterium	
		g__Paludibaculum	Bacteria;Acidobacteriota;Acidobacteriae;Paludibaculum;Paludibaculum fermentans	
		g__PNKE01	Bacteria;Acidobacteriota;Acidobacteriae;Paludibaculum;uncultured Acidobacteria bacterium	
		g__PSRJ01	No 16S rRNA gene	
		g__QHXW01	Bacteria;Acidobacteriota;Acidobacteriae;Solibacterales;Solibacteraceae;Candidatus Solibacter;uncultured Acidobacteria bacterium	
		g__Solibacter	Bacteria;Acidobacteriota;Acidobacteriae;Solibacterales;Solibacteraceae;Candidatus Solibacter;Candidatus Solibacter usitatus Ellin6076	
		g__Sulfopaludibacter	No 16S rRNA gene	
		g__SYKU01	No 16S rRNA gene	
		g__SYLY01	Bacteria;Acidobacteriota;Acidobacteriae;Bryobacterales;Bryobacteraceae;Bryobacter;metagenome	
		g__TMP-7	No 16S rRNA gene	
		g__VFZE01	Bacteria;Acidobacteriota;Acidobacteriae;Bryobacterales;Bryobacteraceae;Bryobacter;metagenome	
		g__VFZS01	No 16S rRNA gene	
		g__VFZY01	Bacteria;Acidobacteriota;Acidobacteriae;Bryobacterales;Bryobacteraceae;Bryobacter;metagenome	
	f__JACQDG01	g__JACQDG01	No 16S rRNA gene	NA
		g__JACQDI01	No 16S rRNA gene	
	f__UBA6623	g__RI-231	Bacteria;Acidobacteriota;Acidobacteriae;PAUC26f;uncultured organism	NA
		g__UBA6623	Bacteria;Acidobacteriota;Acidobacteriae;PAUC26f;uncultured bacterium AD370-D1	
		g__VXMF01	Bacteria;Acidobacteriota;Acidobacteriae;PAUC26f;uncultured bacterium	
o__JAIQFV01	f__JAIQFV01	g__JAIQFV01	No/low similarity in Silva	NA
o__JAJPHL01	f__JAJPHL01	g__JAJPHL01	Bacteria;Acidobacteriota;Acidobacteriae;Subgroup 12;uncultured bacterium	Subgroup 12
o__Terriglobales	f__Acidobacteriaceae	g__Acidipila	Bacteria;Acidobacteriota;Acidobacteriae;Acidobacteriales;Acidobacteriaceae (Subgroup 1);Acidipila-Silvibacterium;Acidipila rosea	Subgroup 1
		g__Acidisarcina	Bacteria;Acidobacteriota;Acidobacteriae;Acidobacteriales;Acidobacteriaceae (Subgroup 1);Acidipila-Silvibacterium;Acidobacteriaceae bacterium SBC82	
		g__Acidobacterium	Bacteria;Acidobacteriota;Acidobacteriae;Acidobacteriales;Acidobacteriaceae (Subgroup 1);Acidobacterium;Acidobacterium capsulatum ATCC 51196	
		g__Alloacidobacterium	Bacteria;Acidobacteriota;Acidobacteriae;Acidobacteriales;Acidobacteriaceae (Subgroup 1);Acidipila-Silvibacterium;uncultured Acidobacteria bacterium	
		g__Bryocella	Bacteria;Acidobacteriota;Acidobacteriae;Acidobacteriales;Acidobacteriaceae (Subgroup 1);Bryocella;Bryocella elongata	
		g__CAHJWL01	No 16S rRNA gene	
		g__CAIMNN01	No 16S rRNA gene	
		g__CAIQPK01	Bacteria;Acidobacteriota;Acidobacteriae;Acidobacteriales;Acidobacteriaceae (Subgroup 1);Granulicella;uncultured actinobacterium	
		g__CAIVDV01	Bacteria;Acidobacteriota;Acidobacteriae;Acidobacteriales;Acidobacteriaceae (Subgroup 1);Acidicapsa;Acidicapsa borealis	
		g__CAIZGA01	No 16S rRNA gene	
		g__DZDJ01	Bacteria;Acidobacteriota;Acidobacteriae;Acidobacteriales;Acidobacteriaceae (Subgroup 1);Acidipila-Silvibacterium;uncultured prokaryote	
		g__EB88	Bacteria;Acidobacteriota;Acidobacteriae;Acidobacteriales;Acidobacteriaceae (Subgroup 1);Acidipila-Silvibacterium;uncultured bacterium	
		g__Edaphobacter	Bacteria;Acidobacteriota;Acidobacteriae;Acidobacteriales;Acidobacteriaceae (Subgroup 1);Edaphobacter;Acidobacteriaceae bacterium TAA166	
		g__Granulicella_A	Bacteria;Acidobacteriota;Acidobacteriae;Acidobacteriales;Acidobacteriaceae (Subgroup 1);Granulicella;Granulicella mallensis MP5ACTX8	
		g__Granulicella_B	Bacteria;Acidobacteriota;Acidobacteriae;Acidobacteriales;Acidobacteriaceae (Subgroup 1);Granulicella;Granulicella arctica	
		g__Granulicella_C	Bacteria;Acidobacteriota;Acidobacteriae;Acidobacteriales;Acidobacteriaceae (Subgroup 1);Granulicella;Granulicella rosea	
		g__Granulicella_D	Bacteria;Acidobacteriota;Acidobacteriae;Acidobacteriales;Acidobacteriaceae (Subgroup 1);Granulicella;Granulicella aggregans	
		g__JAJXYY01	Bacteria;Acidobacteriota;Acidobacteriae;Acidobacteriales;Acidobacteriaceae (Subgroup 1);Granulicella;uncultured bacterium	
		g__JAJYVU01	No 16S rRNA gene	
		g__JAJZCX01	Bacteria;Acidobacteriota;Acidobacteriae;Acidobacteriales;Acidobacteriaceae (Subgroup 1);uncultured;uncultured bacterium	
		g__JAJZFE01	Bacteria;Acidobacteriota;Acidobacteriae;Acidobacteriales;Acidobacteriaceae (Subgroup 1);Granulicella;Granulicella sp. 5B5	
		g__JAJZHR01	No 16S rRNA gene	
		g__JAJZIF01	No 16S rRNA gene	
		g__JAJZIJ01	Bacteria;Acidobacteriota;Acidobacteriae;Acidobacteriales;Acidobacteriaceae (Subgroup 1);uncultured;uncultured Acidobacterium sp.	
		g__JAJZJJ01	No 16S rRNA gene	
		g__JAKAAZ01	Bacteria;Acidobacteriota;Acidobacteriae;Acidobacteriales;Acidobacteriaceae (Subgroup 1);Granulicella;uncultured bacterium	
		g__JAKATU01	Bacteria;Acidobacteriota;Acidobacteriae;Acidobacteriales;Acidobacteriaceae (Subgroup 1);Acidobacterium;uncultured Acidobacterium sp.	
		g__JAKAUB01	No 16S rRNA gene	
		g__JAKAYS01	No 16S rRNA gene	
		g__KBS-83	Bacteria;Acidobacteriota;Acidobacteriae;Acidobacteriales;Acidobacteriaceae (Subgroup 1);Acidipila-Silvibacterium;metagenome	
		g__Palsa-288	Bacteria;Acidobacteriota;Acidobacteriae;Acidobacteriales;Acidobacteriaceae (Subgroup 1);Edaphobacter;Edaphobacter sp.	
		g__Palsa-343	Bacteria;Acidobacteriota;Acidobacteriae;Acidobacteriales;Acidobacteriaceae (Subgroup 1);Edaphobacter;uncultured Acidobacteria bacterium	
		g__PALSA-350	No 16S rRNA gene	
		g__Paracidobacterium	Bacteria;Acidobacteriota;Acidobacteriae;Acidobacteriales;Acidobacteriaceae (Subgroup 1);Acidipila-Silvibacterium;Acidipila sp.	
		g__Pseudacidobacterium	Bacteria;Acidobacteriota;Acidobacteriae;Acidobacteriales;Acidobacteriaceae (Subgroup 1);uncultured;uncultured bacterium	
		g__Silvibacterium	Bacteria;Acidobacteriota;Acidobacteriae;Acidobacteriales;Acidobacteriaceae (Subgroup 1);Acidipila-Silvibacterium;Acidipila dinghuensis	
		g__Terracidiphilus	Bacteria;Acidobacteriota;Acidobacteriae;Acidobacteriales;Acidobacteriaceae (Subgroup 1);Acidipila-Silvibacterium;Acidobacteria bacterium CU2	
		g__Terriglobus	Bacteria;Acidobacteriota;Acidobacteriae;Acidobacteriales;Acidobacteriaceae (Subgroup 1);Terriglobus;Terriglobus roseus	

			g__Terriglobus_A	Bacteria;Acidobacteriota;Acidobacteriae;Acidobacteriales;Acidobacteriaceae (Subgroup 1);Terriglobus;Terriglobus albidus		
			g__Tous-C9LFEB	Bacteria;Acidobacteriota;Acidobacteriae;Acidobacteriales;Acidobacteriaceae (Subgroup 1);Terriglobus;Terriglobus roseus		
			g__UBA5172	Bacteria;Acidobacteriota;Acidobacteriae;Acidobacteriales;Acidobacteriaceae (Subgroup 1);Granulicella;Granulicella mallensis MP5ACTX8		
		f__CAINCZ01	g__CAINCZ01	No 16S rRNA gene		
		f__DSPA01	g__DSPA01	Bacteria;Acidobacteriota;Acidobacteriae;Acidobacteriales;uncultured;uncultured Acidobacteria bacterium		
		f__Gp1-AA112	g__Gp1-AA112	Bacteria;Acidobacteriota;Acidobacteriae;Acidobacteriales;Acidobacteriaceae (Subgroup 1);uncultured;uncultured bacterium		
		f__Gp1-AA117	g__Angelobacter	Bacteria;Acidobacteriota;Acidobacteriae;Acidobacteriales;uncultured;uncultured bacterium		
		f__JACPNR01	g__JACPNR01	Bacteria;Acidobacteriota;Acidobacteriae;Acidobacteriales;uncultured;uncultured bacterium		
		f__JAEZPW01	g__JAEZPW01	Bacteria;Acidobacteriota;Acidobacteriae;Acidobacteriales;Koribacteraceae;Candidatus Koribacter;uncultured Acidobacteria bacterium		
		f__JAFAIN01	g__JAFAIN01	Bacteria;Acidobacteriota;Acidobacteriae;Acidobacteriales;uncultured;uncultured bacterium		
		f__JAHFUA01	g__JAHFUA01	Bacteria;Acidobacteriota;Acidobacteriae;Acidobacteriales;uncultured;uncultured Acidobacteria bacterium		
		f__JAIQFD01	g__JAIQFD01	Bacteria;Acidobacteriota;Acidobacteriae;Acidobacteriales;uncultured;uncultured bacterium		
		f__JAIQGF01	g__JAIQGF01	Bacteria;Acidobacteriota;Acidobacteriae;Acidobacteriales;uncultured;uncultured Acidobacteria bacterium		
		f__JAJPJE01	g__JAJPJE01	Bacteria;Acidobacteriota;Acidobacteriae;Acidobacteriales;uncultured;uncultured bacterium		
		f__Koribacteraceae	g__Bog-257	Bacteria;Acidobacteriota;Acidobacteriae;Acidobacteriales;Koribacteraceae;Candidatus Koribacter;uncultured Acidobacteria bacterium		
			g__JAJXZT01	Bacteria;Acidobacteriota;Acidobacteriae;Acidobacteriales;Koribacteraceae;Candidatus Koribacter;uncultured bacterium		
			g__Koribacter	Bacteria;Acidobacteriota;Acidobacteriae;Acidobacteriales;Koribacteraceae;Candidatus Koribacter;Candidatus Koribacter versatilis Ellin345		
			g__TOLSYN	Bacteria;Acidobacteriota;Acidobacteriae;Acidobacteriales;Acidobacteriaceae (Subgroup 1);Occallatibacter;uncultured bacterium		
		f__QIAW01	g__QIAW01	No 16S rRNA gene		
		f__SCQP01	g__JAJPKR01	Bacteria;Acidobacteriota;Acidobacteriae;Acidobacteriales;Acidobacteriaceae (Subgroup 1);uncultured;uncultured bacterium		
			g__JAKASR01	Bacteria;Acidobacteriota;Acidobacteriae;Acidobacteriales;Acidobacteriaceae (Subgroup 1);uncultured;uncultured bacterium		
			g__JAKAUF01	Bacteria;Acidobacteriota;Acidobacteriae;Acidobacteriales;Acidobacteriaceae (Subgroup 1);uncultured;uncultured bacterium		
			g__JAKAZV01	No 16S rRNA gene		
			g__SCQP01	No/low similarity in Silva		
		f__SbA1	g__Bog-209	Bacteria;Acidobacteriota;Acidobacteriae;Acidobacteriales;Acidobacteriaceae (Subgroup 1);Acidipila-Silvibacterium;uncultured Acidobacteria bacterium		
			g__Gp1-AA122	Bacteria;Acidobacteriota;Acidobacteriae;Acidobacteriales;uncultured;uncultured bacterium		
			g__Gp1-AA124	Bacteria;Acidobacteriota;Acidobacteriae;Acidobacteriales;uncultured;uncultured bacterium		
			g__Gp1-AA133	Bacteria;Acidobacteriota;Acidobacteriae;Acidobacteriales;uncultured;uncultured Acidobacteria bacterium		
			g__Gp1-AA142	No/low similarity in Silva		
			g__Gp1-AA145	Bacteria;Acidobacteriota;Acidobacteriae;Acidobacteriales;uncultured;uncultured Acidobacteria bacterium		
			g__JABCYV01	No 16S rRNA gene		
			g__JABCZH01	No 16S rRNA gene		
			g__JABDBE01	Bacteria;Acidobacteriota;Acidobacteriae;Acidobacteriales;uncultured;uncultured bacterium		
			g__JAFAGX01	No 16S rRNA gene		
			g__JAFAWN01	No 16S rRNA gene		
			g__JAIQET01	Bacteria;Acidobacteriota;Acidobacteriae;Acidobacteriales;uncultured;uncultured Acidobacteria bacterium		
			g__JAIQEU01	Bacteria;Acidobacteriota;Acidobacteriae;Acidobacteriales;uncultured;uncultured soil bacterium		
			g__JAIQEY01	Bacteria;Acidobacteriota;Acidobacteriae;Acidobacteriales;uncultured;uncultured bacterium DA038		
			g__JAIQFF01	No 16S rRNA gene		
			g__JAIQFW01	No 16S rRNA gene		
			g__JAJPHW01	No 16S rRNA gene		
			g__JAMXLI01	Bacteria;Acidobacteriota;Acidobacteriae;Acidobacteriales;uncultured;uncultured Acidobacteria bacterium		
			g__KAN-5	No 16S rRNA gene		
			g__PALSA-188	No 16S rRNA gene		
			g__PALSA-270	Bacteria;Acidobacteriota;Acidobacteriae;Acidobacteriales;uncultured;uncultured bacterium		
			g__QIAA01	Bacteria;Acidobacteriota;Acidobacteriae;Acidobacteriales;uncultured;uncultured bacterium		
			g__QIAB01	No 16S rRNA gene		
			g__QIAJ01	Bacteria;Acidobacteriota;Acidobacteriae;Acidobacteriales;uncultured;uncultured Acidobacteria bacterium		
			g__QIAS01	No 16S rRNA gene		
			g__Sulfotelmatobacter	Bacteria;Acidobacteriota;Acidobacteriae;Acidobacteriales;uncultured;uncultured Acidobacterium UA3		
			g__40CM-3-55-5	Bacteria;Acidobacteriota;Acidobacteriae;Acidobacteriales;uncultured;	Subgroup 14	
	o__UBA7540	f__UBA7540	g__Bog-366	Bacteria;Acidobacteriota;Acidobacteriae;Subgroup 13;uncultured bacterium	Subgroup 13	
			g__CADDZC01	Bacteria;Acidobacteriota;Acidobacteriae;Subgroup 13;uncultured bacterium		
			g__CADDZE01	No 16S rRNA gene		
			g__Gp13-AA74	Bacteria;Acidobacteriota;Acidobacteriae;Subgroup 13;uncultured bacterium		
			g__Gp13-AA77	Bacteria;Acidobacteriota;Acidobacteriae;Subgroup 13;uncultured bacterium		
			g__JACQNR01	No 16S rRNA gene		
			g__JAHEKS01	Bacteria;Acidobacteriota;Acidobacteriae;Subgroup 13;uncultured bacterium		
			g__JAIQGA01	Bacteria;Acidobacteriota;Acidobacteriae;Subgroup 13;uncultured bacterium		
			g__JAIQGB01	Bacteria;Acidobacteriota;Acidobacteriae;Subgroup 13;uncultured bacterium		
			g__JAKASG01	No 16S rRNA gene		
			g__JAKCCV01	Bacteria;Acidobacteriota;Acidobacteriae;Subgroup 13;uncultured bacterium		
			g__JALHUF01	Bacteria;Acidobacteriota;Acidobacteriae;Subgroup 13;uncultured bacterium		
			g__JAMCXS01	No 16S rRNA gene		
			g__QHZM01	Bacteria;Acidobacteriota;Acidobacteriae;Subgroup 13;uncultured bacterium		
			g__RH2-MAG17b	Bacteria;Acidobacteriota;Acidobacteriae;Subgroup 13;uncultured Acidobacteria bacterium		
			g__SCRD01	Bacteria;Acidobacteriota;Acidobacteriae;Subgroup 13;uncultured bacterium		
			g__UBA7540	Bacteria;Acidobacteriota;Acidobacteriae;Subgroup 13;uncultured bacterium		
	o__VXMN01	f__VXMN01	g__JAKUNG01	Bacteria;Acidobacteriota;Subgroup 11;uncultured bacterium KM3-18-F8	Subgroup 11, 24	
			g__QHZQ01	Bacteria;Acidobacteriota;Subgroup 11;uncultured soil bacterium		
			g__VFZK01	Bacteria;Acidobacteriota;Subgroup 11;uncultured bacterium		
			g__VXMN01	Bacteria;Acidobacteriota;Subgroup 11;uncultured Acidobacteria bacterium		
c__Thermoanaerobaculia	o__Gp7-AA8	f__Gp7-AA8	g__Gp7-AA10	Bacteria;Acidobacteriota;Holophagae;Subgroup 7;uncultured bacterium	Subgroup 7	Class 1-10
			g__JACPDH01	No 16S rRNA gene		
			g__JADGNX01	No 16S rRNA gene		
			g__JADGNZ01	Bacteria;Acidobacteriota;Holophagae;Subgroup 7;uncultured Acidobacteria bacterium		
			g__JAENWF01	Bacteria;Acidobacteriota;Holophagae;Subgroup 7;uncultured bacterium		
			g__JAFAVZ01	No 16S rRNA gene		
			g__JAJPHC01	Bacteria;Acidobacteriota;Holophagae;Subgroup 7;uncultured proteobacterium		
			g__JAJXWV01	Bacteria;Acidobacteriota;Holophagae;Subgroup 7;uncultured bacterium		
			g__QHVS01	No 16S rRNA gene		
			g__QHVT01	No 16S rRNA gene		
		f__JAHWKK01	g__JAHWKK01	No/low similarity in Silva		
	o__UBA5066	f__UBA5066	g__CAIMWE01	Bacteria;Acidobacteriota;Holophagae;Subgroup 7;uncultured bacterium		
			g__Fen-183	Bacteria;Acidobacteriota;Holophagae;Subgroup 7;uncultured bacterium		
			g__JAEUQS01	No 16S rRNA gene		
			g__JAJVIN01	No/low similarity in Silva		
			g__RHKY01	Bacteria;Acidobacteriota;Holophagae;Subgroup 7;uncultured bacterium SJA-36		
			g__UBA5066	Bacteria;Acidobacteriota;Holophagae;Subgroup 7;uncultured Acidobacteria bacterium		
		f__Gp7-AA6	g__Gp7-AA6	Bacteria;Acidobacteriota;Holophagae;Subgroup 7;uncultured bacterium		
	o__UBA2201	f__UBA2201	g__UBA2201	No/low similarity in Silva		
	o__Thermoanaerobaculales	f__Sulfomarinibacteraceae	g__FEB-10	No/low similarity in Silva	Subgroup 23	Class 4-2
			g__JAADFK01	Bacteria;Acidobacteriota;Thermoanaerobaculia;Thermoanaerobaculales;Thermoanaerobaculaceae;Subgroup 23;uncultured bacterium		
			g__JAADFN01	Bacteria;Acidobacteriota;Thermoanaerobaculia;Thermoanaerobaculales;Thermoanaerobaculaceae;Subgroup 23;uncultured Acidobacteria bacterium		
			g__JAAESQ01	No/low similarity in Silva		
			g__JAHECW01	Bacteria;Acidobacteriota;Thermoanaerobaculia;Thermoanaerobaculales;Thermoanaerobaculaceae;Subgroup 23;uncultured Acidobacteria bacterium		
			g__JAIOSJ01	Bacteria;Acidobacteriota;Thermoanaerobaculia;Thermoanaerobaculales;Thermoanaerobaculaceae;Subgroup 23;uncultured bacterium		
			g__JAJDNH01	No 16S rRNA gene		
			g__JANTFG01	Bacteria;Acidobacteriota;Thermoanaerobaculia;Thermoanaerobaculales;Thermoanaerobaculaceae;Subgroup 23;uncultured bacterium		
			g__Sulfomarinibacter	No 16S rRNA gene		
		f__Thermoanaerobaculaceae	g__JAAYVZ01	Bacteria;Acidobacteriota;Thermoanaerobaculia;Thermoanaerobaculales;Thermoanaerobaculaceae;Thermoanaerobaculum;metagenome		
			g__JACADV01	Bacteria;Acidobacteriota;Thermoanaerobaculia;Thermoanaerobaculales;Thermoanaerobaculaceae;Thermoanaerobaculum;uncultured prokaryote		
			g__JACQZW01	Bacteria;Acidobacteriota;Thermoanaerobaculia;Thermoanaerobaculales;Thermoanaerobaculaceae;Thermoanaerobaculum;uncultured Acidobacteria bacterium		
			g__RBG-13-68-16	Bacteria;Acidobacteriota;Thermoanaerobaculia;Thermoanaerobaculales;Thermoanaerobaculaceae;Thermoanaerobaculum;Acidobacteria bacterium RBG_13_68_16		
			g__Thermoanaerobaculum	No/low similarity in Silva		
	o__UBA5704	f__JAIOJS01	g__JAIOJS01	No/low similarity in Silva	Subgroup 10	Class 4-1
		f__QQVD01	g__QQVD01	Bacteria;Acidobacteriota;Thermoanaerobaculia;Thermoanaerobaculales;Thermoanaerobaculaceae;Subgroup 10;uncultured bacterium gp10		
			g__WTGL01	Bacteria;Acidobacteriota;Thermoanaerobaculia;Thermoanaerobaculales;Thermoanaerobaculaceae;Subgroup 10;uncultured Acidobacteria bacterium		
		f__UBA5704	g__CAJQNK01	No 16S rRNA gene		
			g__CALZIK01	Bacteria;Acidobacteriota;Thermoanaerobaculia;Thermoanaerobaculales;Thermoanaerobaculaceae;Subgroup 10;uncultured bacterium		
			g__CALZJC01	No 16S rRNA gene		
			g__CALZKB01	Bacteria;Acidobacteriota;Thermoanaerobaculia;Thermoanaerobaculales;Thermoanaerobaculaceae;Subgroup 10;uncultured bacterium		
			g__J023	No 16S rRNA gene		
			g__JAAELT01	No 16S rRNA gene		
			g__JAAYLR01	Bacteria;Acidobacteriota;Thermoanaerobaculia;Thermoanaerobaculales;Thermoanaerobaculaceae;Subgroup 10;Acidobacteria bacterium ADurb.Bin051		
			g__JACTMI01	Bacteria;Acidobacteriota;Thermoanaerobaculia;Thermoanaerobaculales;Thermoanaerobaculaceae;Subgroup 10;bacterium enrichment culture clone Anammox_2		
			g__JAGPDF01	Bacteria;Acidobacteriota;Thermoanaerobaculia;Thermoanaerobaculales;Thermoanaerobaculaceae;Subgroup 10;metagenome		
			g__JAHEKO01	No 16S rRNA gene		
			g__JAHZIX01	No/low similarity in Silva		
			g__M0029	No 16S rRNA gene		
			g__SZUA-115	No/low similarity in Silva		
			g__UBA5704	Bacteria;Acidobacteriota;Thermoanaerobaculia;Thermoanaerobaculales;Thermoanaerobaculaceae;Subgroup 10;uncultured bacterium		
c__UBA4820	o__UBA4820	f__DSQY01	g__DTPS01	Bacteria;Acidobacteriota;Thermoanaerobaculia;Thermoanaerobaculales;Thermoanaerobaculaceae;TPD-58;uncultured bacterium	NA	Candidate class 1-11
			g__JAEMPZ01	No/low similarity in Silva		
			g__JAKAJA01	Bacteria;Acidobacteriota;Thermoanaerobaculia;Thermoanaerobaculales;Thermoanaerobaculaceae;TPD-58;uncultured bacterium		
		f__UBA4820	g__UBA4820	Bacteria;Acidobacteriota;Thermoanaerobaculia;Thermoanaerobaculales;Thermoanaerobaculaceae;TPD-58;uncultured bacterium		
c__UBA6911	o__CAINFC01	f__CAINFC01	g__CAINFC01	Bacteria;Acidobacteriota;uncultured bacterium	NA	NA
	o__JAAYUB01	f__JAAYUB01	g__JAAYUB01	Bacteria;Acidobacteriota;c5LKS83;metagenome	NA	Candidate Class 1-3
			g__JAFGRT01	No/low similarity in Silva	NA	NA
			g__JAGNHB01	No/low similarity in Silva	NA	NA
	o__JACOSC01	f__JACOSC01	g__JACOSC01	Bacteria;Acidobacteriota;FFCH5909;uncultured bacterium	NA	NA
	o__JACQTF01	f__JACQTF01	g__JACQTF01	No 16S rRNA gene	NA	NA
	o__RPQK01	f__JACQSX01	g__JACQSX01	Bacteria;Acidobacteriota;Subgroup 20;uncultured bacterium	Subgroup 20	Class 1-4
		f__RPQK01	g__G020350445	No/low similarity in Silva	Subgroup 21	Class 6-2
			g__JACQSY01	No 16S rRNA gene		

			g__JADFOI01	Bacteria;Acidobacteriota;Subgroup 21;uncultured bacterium		
			g__JAJDTT01	No 16S rRNA gene		
			g__RPQK01	No 16S rRNA gene		
	o__UBA6911	f__UBA6911	g__Gp18-AA60	Bacteria;Acidobacteriota;Subgroup 18;uncultured bacterium	Subgroup 18	Class 1-2
			g__JAAYAM01	Bacteria;Acidobacteriota;Subgroup 18;uncultured Acidobacteria bacterium		
			g__JAAYCX01	No/low similarity in Silva		
			g__JACADW01	No/low similarity in Silva		
			g__JAFNAG01	Bacteria;Acidobacteriota;Subgroup 18;uncultured bacterium		
			g__JAHFUH01	No 16S rRNA gene		
			g__UBA6911	Bacteria;Acidobacteriota;Subgroup 18;uncultured Acidobacteria bacterium		
**No genomes available in Gtdb**				Bacteria;Acidobacteriota;Subgroup 19;	Subgroup 19	
c__UBA890	o__UBA890	f__UBA890	g__UBA890	Bacteria;Acidobacteriota;Subgroup 26;uncultured bacterium	Subgroup 26	Class 4-3
		f__WLXD01	g__JACZXM01	No 16S rRNA gene		
			g__WLXD01	Bacteria;Acidobacteriota;Subgroup 26;uncultured bacterium		
c__G020349885	o__G020349885	f__G020349885	g__G020349885	Bacteria;Acidobacteriota;Subgroup 26;uncultured bacterium		
c__Vicinamibacteria	o__Bin61	f__Bin61	g__Bin61	Bacteria;Acidobacteriota;Vicinamibacteria;Subgroup 9;uncultured bacterium	Subgroup 9	Class 1-5
		f__SMYC01	g__SMYC01	Bacteria;Acidobacteriota;Vicinamibacteria;Subgroup 9;uncultured bacterium		
	o__Fen-336	f__Fen-336	g__AA29	No 16S rRNA gene	Subgroup 17	
			g__AA32	No 16S rRNA gene		
			g__CADEEV01	Bacteria;Acidobacteriota;Vicinamibacteria;Subgroup 17;uncultured Acidobacteria bacterium		
			g__FEN-336	No 16S rRNA gene		
			g__JACQGI01	No 16S rRNA gene		
			g__JAFGSN01	Bacteria;Acidobacteriota;Vicinamibacteria;Subgroup 17;uncultured Acidobacteria bacterium		
			g__JAJDNS01	No 16S rRNA gene		
			g__WYBL01	Bacteria;Acidobacteriota;Vicinamibacteria;Subgroup 17;uncultured bacterium		
		f__JAGNNV01	g__JAGNNV01	Bacteria;Acidobacteriota;Vicinamibacteria;Subgroup 17;metagenome		
	o__Vicinamibacterales	f__2-12-FULL-66-21	g__2-12-FULL-66-21	No 16S rRNA gene	Subgroup 6	
			g__Gp6-AA38	No 16S rRNA gene		
			g__JACCRT01	No 16S rRNA gene		
			g__JACCXG01	No 16S rRNA gene		
			g__JACDCA01	Bacteria;Acidobacteriota;Vicinamibacteria;Vicinamibacterales;uncultured;uncultured Acidobacteria bacterium		
			g__JACPDI01	Bacteria;Acidobacteriota;Vicinamibacteria;Vicinamibacterales;Vicinamibacteraceae;uncultured bacterium		
			g__JAHECP01	Bacteria;Acidobacteriota;Vicinamibacteria;Vicinamibacterales;uncultured;uncultured Acidobacteria bacterium		
			g__JAIVJE01	Bacteria;Acidobacteriota;Vicinamibacteria;Vicinamibacterales;uncultured;uncultured Acidobacteria bacterium		
			g__QHWT01	Bacteria;Acidobacteriota;Vicinamibacteria;Vicinamibacterales;uncultured;uncultured bacterium		
		f__Fen-181	g__Fen-181	Bacteria;Acidobacteriota;Vicinamibacteria;Vicinamibacterales;uncultured;uncultured Acidobacteria bacterium		
			g__FEN-299	Bacteria;Acidobacteriota;Vicinamibacteria;Vicinamibacterales;uncultured;uncultured bacterium		
		f__JACPPN01	g__JACPPN01	No 16S rRNA gene		
		f__JACQTH01	g__JACQTH01	No 16S rRNA gene		
		f__JAFNAJ01	g__JAEUQQ01	No 16S rRNA gene		
			g__JAFNAJ01	No 16S rRNA gene		
			g__JAHDVP01	No 16S rRNA gene		
			g__JAHDVQ01	No 16S rRNA gene		
		f__SCN-69-37	g__JADYYY01	Bacteria;Acidobacteriota;Vicinamibacteria;Vicinamibacterales;uncultured;uncultured bacterium		
			g__SCN-69-37	Bacteria;Acidobacteriota;Vicinamibacteria;Vicinamibacterales;uncultured;Acidobacteria bacterium SCN 69-37		
			g__SCUN01	Bacteria;Acidobacteriota;Vicinamibacteria;Vicinamibacterales;uncultured;uncultured Acidobacteriaceae bacterium		
			g__SYFT01	Bacteria;Acidobacteriota;Vicinamibacteria;Vicinamibacterales;uncultured;metagenome		
		f__UBA2999	g__12-FULL-67-14b	Bacteria;Acidobacteriota;Vicinamibacteria;Vicinamibacterales;Vicinamibacteraceae;uncultured bacterium		
			g__2-02-FULL-65-29	No 16S rRNA gene		
			g__2-12-FULL-65-11	No 16S rRNA gene		
			g__CADEFD01	Bacteria;Acidobacteriota;Vicinamibacteria;Vicinamibacterales;Vicinamibacteraceae;uncultured bacterium		
			g__Gp6-AA40	Bacteria;Acidobacteriota;Vicinamibacteria;Vicinamibacterales;Vicinamibacteraceae;uncultured Acidobacteria bacterium		
			g__Gp6-AA45	Bacteria;Acidobacteriota;Vicinamibacteria;Vicinamibacterales;uncultured;uncultured Acidobacteriales bacterium		
			g__JABFRY01	Bacteria;Acidobacteriota;Vicinamibacteria;Vicinamibacterales;Vicinamibacteraceae;uncultured bacterium		
			g__JACCSI01	No 16S rRNA gene		
			g__JADGOC01	Bacteria;Acidobacteriota;Vicinamibacteria;Vicinamibacterales;uncultured;uncultured Acidobacteria bacterium		
			g__JADZED01	Bacteria;Acidobacteriota;Vicinamibacteria;Vicinamibacterales;uncultured;uncultured Acidobacteria bacterium		
			g__JAENWD01	No 16S rRNA gene		
			g__JAHFUI01	Bacteria;Acidobacteriota;Vicinamibacteria;Vicinamibacterales;uncultured;uncultured bacterium		
			g__JAHFUJ01	Bacteria;Acidobacteriota;Vicinamibacteria;Vicinamibacterales;uncultured;uncultured bacterium		
			g__JAIVJN01	No 16S rRNA gene		
			g__JAKFYA01	No 16S rRNA gene		
			g__JAMQPK01	No 16S rRNA gene		
			g__UBA2999	Bacteria;Acidobacteriota;Vicinamibacteria;Vicinamibacterales;uncultured;uncultured bacterium		
			g__VFZH01	Bacteria;Acidobacteriota;Vicinamibacteria;Vicinamibacterales;Vicinamibacteraceae;uncultured Acidobacteria bacterium		
			g__VFZN01	Bacteria;Acidobacteriota;Vicinamibacteria;Vicinamibacterales;Vicinamibacteraceae;uncultured bacterium mle1-25		
			g__WHSN01	Bacteria;Acidobacteriota;Vicinamibacteria;Vicinamibacterales;uncultured;metagenome		
		f__UBA823	g__UBA11600	Bacteria;Acidobacteriota;Vicinamibacteria;Vicinamibacterales;uncultured;uncultured bacterium		
			g__UBA2161	Bacteria;Acidobacteriota;Vicinamibacteria;Vicinamibacterales;uncultured;uncultured Acidimicrobiales bacterium		
			g__UBA8106	Bacteria;Marinimicrobia (SAR406 clade);uncultured Candidatus Marinimicrobia bacterium		
			g__UBA823	Bacteria;Acidobacteriota;Vicinamibacteria;Vicinamibacterales;uncultured;uncultured Acidimicrobiales bacterium		
		f__UBA8438	g__DTWD01	No 16S rRNA gene		
			g__JAAXGX01	Bacteria;Acidobacteriota;Vicinamibacteria;Vicinamibacterales;uncultured;uncultured bacterium		
			g__JAGWAJ01	No 16S rRNA gene		
			g__JAJEEA01	Bacteria;Acidobacteriota;Vicinamibacteria;Vicinamibacterales;uncultured;uncultured bacterium		
			g__NP936	Bacteria;Acidobacteriota;Vicinamibacteria;Vicinamibacterales;uncultured;uncultured Acidobacteria bacterium		
			g__UBA2990	Bacteria;Acidobacteriota;Vicinamibacteria;Vicinamibacterales;uncultured;uncultured bacterium KM3-173-A5		
			g__UBA2994	Bacteria;Actinobacteriota;Actinobacteria;Micrococcales;Microbacteriaceae;Microbacterium;marine metagenome		
			g__UBA8438	No/low similarity in Silva		
			g__UBA9620	No 16S rRNA gene		
			g__VXRJ01	Bacteria;Acidobacteriota;Vicinamibacteria;Vicinamibacterales;uncultured;uncultured Acidobacteria bacterium		
			g__WTFV01	Bacteria;Acidobacteriota;Vicinamibacteria;Vicinamibacterales;uncultured;uncultured Acidobacteria bacterium		
		f__Vicinamibacteraceae	g__JAEZDP01	No 16S rRNA gene		
			g__Luteitalea	Bacteria;Acidobacteriota;Vicinamibacteria;Vicinamibacterales;Vicinamibacteraceae;Luteitalea;bacterium enrichment culture clone DSR_54		
			g__WHTT01	Bacteria;Acidobacteriota;Vicinamibacteria;Vicinamibacterales;Vicinamibacteraceae;Luteitalea;uncultured bacterium		
**No genomes available in Gtdb**				Bacteria;Acidobacteriota;AT-s3-28;	NA	Candidate Class 1-6
**No genomes available in Gtdb**				Bacteria;Acidobacteriota;d142;	NA	NA

Table S2. Ecological distribution of Acidobacteriota in the 248,559 metagenomic studies in Sandpiper. Results are shown at the phylum level, all 14 classes, as well as their constituting orders and families. For each taxonomy level, results are shown for ubiquity (measured as the percentage occurrence in datasets from engineered, freshwater, host-associated, marine, non-marine saline and alkaline, terrestrial non-soil, as well as soil datasets). Percentage occurrence in all non-soil datasets is also shown. The ratio between percentage occurrence in soil versus non-soil datasets is calculated as a measure for soil-preference. In addition to occurrence, values of percentage abundance in each of the 7 habitat classifications, as well as percentage abundance in all non-soil datasets are also shown for each of the taxonomy levels. Finally, the ratio between percentage abundance in soil versus non-soil datasets is also shown.

GTDB phylum	Ubiquity (Percentage occurrence of total datasets								Preference ratio (soil/ Non-soil)	Percentage abundance								Abundance ratio (Soil/ Non-soil)
	Engineered	Freshwater	Host	Marine	Non-marine Saline and Alkaline	Terrestrial non-soil	Soil	Non soil		Engineered	Freshwater	Host	Marine	Non-marine Saline and Alkaline	Terrestrial non-soil	Soil	Non soil	
p__Acidobacteriota	36.97	47.50	3.44	39.99	38.21	10.42	95.75	37.03	2.59	2.09	4.00	6.13	0.88	1.40	4.76	15.79	3.71	0.23

GTDB class	Ubiquity (Percentage occurrence of total datasets								Preference ratio (soil/ Non-soil)	Percentage abundance								Abundance ratio (Soil/ Non-soil)
	Engineered	Freshwater	Host	Marine	Non-marine Saline and Alkaline	Terrestrial non-soil	Soil	Non soil		Engineered	Freshwater	Host	Marine	Non-marine Saline and Alkaline	Terrestrial non-soil	Soil	Non soil	
c__Aminicenantia	8.78	10.48	0.11	6.20	11.79	0.95	11.50	7.70	1.49	0.55	2.52	1.57	0.82	0.46	0.27	0.38	1.18	0.33
c__B3-B38	0.02	0.14	0.00	0.97	0.77	0.00	0.22	0.34	0.66	0.03	0.01		0.20	0.01		0.01	0.15	0.05
c__Blastocatellia	19.72	23.21	1.57	7.12	10.26	2.90	76.20	14.88	5.12	0.92	1.80	2.28	0.06	0.05	2.64	1.91	1.35	1.42
c__CAIWXX01	0.05	0.22	0.00	0.00	0.00	0.00	0.20	0.07	2.98	0.02	0.02	0.97				0.01	0.10	0.09
c__Holophagae	12.95	18.27	0.30	3.42	6.13	0.69	24.00	9.98	2.40	0.42	0.69	0.40	0.08	0.08	0.29	0.33	0.45	0.72
c__HRBIN11	0.07	2.02	0.21	0.17	0.08	0.32	15.78	0.59	26.73	0.61	0.09	0.28	0.03		0.03	0.07	0.15	0.48
c__Polarisedimenticolia	3.15	9.84	0.35	7.16	7.96	1.05	33.47	5.81	5.76	0.11	0.40	0.23	0.42	0.27	0.44	0.23	0.33	0.69
c__Terriglobia	21.30	35.04	1.74	15.58	14.09	3.47	92.61	20.84	4.44	0.67	1.35	3.93	0.18	0.38	1.86	9.76	1.88	5.18
c__Thermoanaerobaculia	18.01	19.11	1.34	12.05	13.78	2.94	78.84	14.85	5.31	0.50	0.71	1.70	0.57	0.66	0.89	0.92	0.82	1.12

GTDB order	Ubiquity (Percentage occurrence of total datasets								Preference ratio (soil/ Non-soil)	Percentage abundance								Abundance ratio (Soil/ Non-soil)
	Engineered	Freshwater	Host	Marine	Non-marine Saline and Alkaline	Terrestrial non-soil	Soil	Non soil		Engineered	Freshwater	Host	Marine	Non-marine Saline and Alkaline	Terrestrial non-soil	Soil	Non soil	
o__Aminicenantales	4.34	8.15	0.06	4.36	9.26	0.69	9.85	5.00	1.97	0.67	2.88	2.38	0.99	0.53	0.20	0.36	1.37	0.26
o__JAMOAD01	3.50	0.34	0.01	0.16	0.00	0.02	0.45	1.32	0.34	0.24	0.21	0.08	0.16		0.00	0.11	0.23	0.49
o__JdFR-78	0.00	0.11	0.00	0.21	0.00	0.00	0.01	0.09	0.11		0.03		0.82			0.02	0.59	0.03
o__S016-38	0.00	0.00	0.00	0.45	0.00	0.00	0.00	0.13	0.00				2.82				0.25	0.00
o__UBA2199	1.63	3.88	0.03	2.18	4.36	0.06	3.68	2.23	1.65	0.55	0.67	0.39	0.19	0.08	0.20	0.18	0.48	0.37
o__B3-B38	0.00	0.04	0.00	0.71	0.31	0.00	0.06	0.22	0.26		0.01		0.26	0.01			0.24	0.00
o__JAFGBY01	0.02	0.08	0.00	0.25	0.38	0.00	0.02	0.11	0.18	0.04	0.01		0.01	0.02		0.05	0.01	3.21
o__Chloracidobacteriales	3.03	2.12	0.14	0.47	0.15	0.28	12.04	1.71	7.06	0.23	1.07	0.06	0.01	0.01	0.04	0.02	0.32	0.08
o__HR10	0.06	1.14	0.04	0.64	0.00	0.20	2.04	0.50	4.11	13.08	20.07	0.01	0.02		0.04	0.01	7.07	0.00
o__Pyrinomonadales	16.86	13.81	1.13	3.60	5.44	2.68	70.68	10.50	6.73	0.68	0.89	1.29	0.08	0.07	2.68	1.77	0.97	1.83
o__RBC074	7.93	8.72	0.62	0.55	0.15	0.95	26.92	5.04	5.34	0.48	0.21	3.05	0.02	0.02	0.27	0.15	0.91	0.17
o__UBA7656	4.86	4.19	0.47	0.31	0.00	1.17	42.47	2.86	14.83	0.17	0.35	0.23	0.01		0.14	0.21	0.21	0.98
o__CAIWXX01	0.05	0.22	0.00	0.00	0.00	0.00	0.20	0.07	2.98	0.02	0.02	0.97				0.01	0.10	0.09
o__Acanthopleuribacterales	0.94	0.37	0.02	2.05	3.29	0.04	0.34	1.09	0.31	0.32	0.11	0.12	0.09	0.07	0.03	0.10	0.17	0.61
o__Holophagales	11.64	15.75	0.22	0.94	0.23	0.48	19.26	8.04	2.40	0.45	0.79	0.53	0.12	0.02	0.34	0.40	0.55	0.73
o__Thermotomaculales	0.04	0.44	0.00	1.00	1.91	0.02	0.10	0.46	0.21	0.55	0.03	0.06	0.08	0.09	0.04	0.16	0.08	1.90
o__HRBIN11	0.04	0.11	0.00	0.05	0.00	0.00	0.00	0.05	0.00	1.20	0.25		0.10				0.41	0.00
o__JAKEFK01	0.02	1.57	0.20	0.03	0.00	0.28	5.07	0.42	12.10	0.05	0.09	0.28	0.00		0.03	0.07	0.16	0.46
o__MFGW01	0.03	0.26	0.00	0.00	0.00	0.00	0.15	0.07	2.01	0.02	0.04					0.02	0.04	0.51
o__Gp22-AA2	0.21	4.48	0.16	1.31	2.07	0.34	17.21	1.61	10.70	0.08	0.19	0.19	0.07	0.04	0.26	0.16	0.15	1.08
o__J045	2.29	3.04	0.03	1.82	2.07	0.14	3.16	2.10	1.51	0.09	0.08	0.07	0.05	0.03	0.06	0.03	0.08	0.41
o__JACPZX01	0.02	1.67	0.04	0.54	0.77	0.10	3.78	0.59	6.40	0.03	0.07	0.03	0.03	0.03	0.11	0.03	0.05	0.63
o__Polarisedimenticolales	0.67	14.89	0.21	5.68	6.36	0.69	20.42	5.66	3.61	0.11	0.34	0.14	0.41	0.27	0.43	0.14	0.31	0.45
o__QHYA01	0.01	0.62	0.02	0.09	0.08	0.04	1.40	0.18	7.74	0.01	0.04	0.03	0.00	0.01	0.22	0.03	0.03	1.04
o__SMYD01	0.00	0.23	0.01	1.07	1.30	0.00	0.31	0.40	0.78		0.03	0.01	0.04	0.03		0.04	0.04	1.02
o__2-12-FULL-54-10	0.40	5.36	0.18	0.94	1.68	0.50	17.86	1.79	10.01	0.05	0.13	0.05	0.06	0.05	0.22	0.07	0.10	0.70
o__20CM-2-55-15	1.08	8.13	0.54	3.85	5.51	1.09	60.86	3.68	16.52	0.17	0.21	0.37	0.05	0.12	0.37	0.38	0.23	1.69
o__Acidoferrales	1.19	7.15	0.52	1.59	3.52	0.75	56.70	2.74	20.66	0.47	0.81	1.45	0.16	0.08	1.51	2.61	0.97	2.68
o__Bryobacterales	17.10	28.59	1.24	8.39	10.03	2.88	88.48	15.63	5.66	0.39	0.70	1.20	0.12	0.23	0.71	2.29	0.74	3.10
o__JAIQFV01	0.06	0.25	0.00	0.02	0.00	0.02	0.73	0.09	8.35	0.08	0.75		0.00		0.00	0.09	0.43	0.21
o__JAJPHL01	0.05	1.12	0.01	0.01	0.00	0.24	7.42	0.31	23.89	0.10	0.33	0.03	0.01		0.11	0.05	0.20	0.23
o__Terriglobales	3.36	11.39	1.01	2.38	5.82	1.49	79.77	4.86	16.42	1.82	1.25	3.96	0.04	0.10	1.39	5.73	2.70	2.13
o__UBA7540	0.70	4.72	0.22	1.23	4.82	0.28	35.10	1.88	18.66	0.19	0.20	0.15	0.12	0.28	0.62	1.37	0.26	5.28
o__VXMN01	0.27	3.76	0.29	2.77	0.23	0.46	21.53	1.84	11.69	0.07	0.19	0.24	0.06	0.01	0.26	0.09	0.16	0.60
o__Gp7-AA8	0.77	6.92	0.59	1.17	3.37	1.83	56.54	2.53	22.35	0.14	0.25	0.40	0.03	0.05	0.43	0.44	0.28	1.56
o__Thermoanaerobaculales	1.90	6.87	0.10	5.35	9.19	0.75	13.04	4.16	3.14	0.43	0.70	0.10	0.41	0.58	1.40	0.20	0.51	0.40
o__UBA2201	0.32	2.21	0.01	4.07	3.83	0.65	2.92	1.99	1.47	0.07	0.05	0.05	0.01	0.00	0.01	0.02	0.05	0.43
o__UBA5066	10.26	8.89	0.61	0.84	0.69	1.05	52.76	5.98	8.82	0.24	0.30	0.47	0.05	0.02	0.17	0.38	0.30	1.26
o__UBA5704	10.80	9.77	0.96	7.80	9.04	1.53	56.33	8.66	6.50	0.46	0.33	1.65	0.51	0.36	0.32	0.31	0.66	0.47

GTDB family	Ubiquity (Percentage occurrence of total datasets								Preference ratio (soil/ Non-soil)	Percentage abundance								Abundance ratio (Soil/ Non-soil)
	Engineered	Freshwater	Host	Marine	Non-marine Saline and Alkaline	Terrestrial non-soil	Soil	Non soil		Engineered	Freshwater	Host	Marine	Non-marine Saline and Alkaline	Terrestrial non-soil	Soil	Non soil	
f__Aminicenantaceae	0.29	4.69	0.02	4.56	0.00	1.19	1.23	2.66	0.46	0.28	1.35	3.05	0.87		1.58	0.15	1.14	0.14
f__JAFGAV01	0.21	0.61	0.01	0.09	0.00	0.06	0.28	0.25	1.14	0.12	0.10	1.04	0.00		0.07	0.03	0.21	0.15
f__JAFGBC01	0.21	1.23	0.01	0.17	0.00	0.00	0.67	0.41	1.64	0.08	0.05	0.11	0.00			0.02	0.05	0.31
f__JdFR-80	0.00	0.44	0.00	1.25	0.54	0.00	0.03	0.48	0.06		0.21	0.31	0.35	0.02		0.00	0.29	0.01
f__RBG-16-66-30	4.94	7.85	0.03	1.22	0.84	0.24	2.12	3.95	0.54	0.31	1.40	0.77	0.18	0.08	0.40	0.44	0.81	0.54
f__Saccharicenantaceae	1.82	0.66	0.02	0.58	0.00	0.16	0.79	0.96	0.82	0.83	0.62	0.08	0.64		0.04	0.03	0.60	0.04
f__UBA4085	1.50	5.56	0.02	0.40	0.00	0.14	5.79	1.96	2.95	0.61	2.54	0.89	0.08		0.04	0.15	1.04	0.15
f__UBA8522	0.04	3.24	0.01	0.41	1.15	0.00	2.31	0.93	2.47	0.07	0.30	0.28	0.03	0.07		0.11	0.25	0.46
f__JAMOAD01	3.50	0.34	0.01	0.16	0.00	0.02	0.45	1.32	0.34	0.24	0.21	0.08	0.16		0.00	0.11	0.23	0.49
f__JdFR-78	0.00	0.11	0.00	0.21	0.00	0.00	0.01	0.09	0.11		0.03		0.82			0.02	0.59	0.03
f__S016-38	0.00	0.00	0.00	0.45	0.00	0.00	0.00	0.13	0.00				0.25				0.25	0.00
f__UBA2199	1.63	3.88	0.03	2.18	4.36	0.06	3.68	2.23	1.65	0.55	0.67	0.39	0.19	0.08	0.20	0.18	0.48	0.37
f__B3-B38	0.00	0.03	0.00	0.39	0.38	0.00	0.00	0.13	0.00		0.00		0.43	0.02			0.38	0.00
f__JAGGZT01	0.00	0.00	0.00	0.33	0.15	0.00	0.00	0.10	0.00				0.23	0.00			0.22	0.00
f__JAFGBY01	0.02	0.08	0.00	0.25	0.38	0.00	0.02	0.11	0.18	0.04	0.01		0.01	0.02		0.05	0.01	3.21
f__Chloracidobacteriaceae	1.86	0.89	0.02	0.14	0.00	0.04	0.85	0.89	0.95	0.16	2.32	0.24	0.00		0.04	0.02	0.56	0.04
f__JADJPF01	1.57	1.05	0.09	0.10	0.00	0.16	5.94	0.83	7.16	0.27	0.09	0.03	0.03		0.04	0.02	0.14	0.17
f__HR10	0.04	0.49	0.00	0.06	0.00	0.06	0.19	0.16	1.19	16.81	46.48		0.00		0.01	0.01	29.97	0.00
f__J050	0.01	0.39	0.00	0.22	0.00	0.06	0.84	0.16	5.09	0.00	0.09	0.01	0.03		0.01	0.01	0.06	0.22
f__JACQTD01	0.00	0.09	0.00	0.00	0.00	0.08	0.09	0.03	3.01		0.01				0.04	0.01	0.02	0.33
f__Pyrinomonadaceae	16.86	13.81	1.13	3.60	5.44	2.68	70.68	10.50	6.73	0.68	0.89	1.29	0.08	0.07	2.68	1.77	0.97	1.83
f__RBC074	7.93	8.72	0.62	0.55	0.15	0.95	26.92	5.04	5.34	0.48	0.21	3.05	0.02	0.02	0.27	0.15	0.91	0.17
f__JADIYM01	4.41	0.76	0.19	0.05	0.00	0.58	13.46	1.76	7.67	0.17	0.04	0.05	0.00		0.06	0.04	0.11	0.38
f__UBA7656	0.40	3.20	0.40	0.12	0.00	0.81	37.25	1.02	36.60	0.12	0.43	0.21	0.00		0.13	0.20	0.25	0.81
f__CAIWXX01	0.05	0.22	0.00	0.00	0.00	0.00	0.20	0.07	2.98	0.02	0.02	0.97				0.01	0.10	0.09
f__Acanthopleuribacteraceae	0.94	0.37	0.02	2.05	3.29	0.04	0.34	1.09	0.31	0.32	0.11	0.12	0.09	0.07	0.03	0.10	0.17	0.61
f__Holophagaceae	11.64	15.75	0.22	0.94	0.23	0.48	19.26	8.04	2.40	0.45	0.79	0.53	0.12	0.02	0.34	0.40	0.55	0.73
f__Thermotomaculaceae	0.04	0.44	0.00	1.00	1.91	0.02	0.10	0.46	0.21	0.55	0.03	0.06	0.08	0.09	0.04	0.16	0.08	1.90
f__HRBIN11	0.04	0.11	0.00	0.05	0.00	0.00	0.00	0.05	0.00	1.20	0.25		0.10				0.41	0.00
f__JAKEFK01	0.02	1.57	0.20	0.03	0.00	0.28	14.83	0.42	35.38	0.05	0.09	0.28	0.00		0.03	0.07	0.16	0.46
f__MFGW01	0.03	0.26	0.00	0.00	0.00	0.00	0.15	0.07	2.01	0.02	0.04					0.02	0.04	0.51
f__AA152	0.03	1.93	0.06	0.40	0.54	0.18	5.93	0.62	9.61	0.08	0.06	0.12	0.02	0.03	0.04	0.06	0.06	1.02
f__Gp22-AA2	0.15	3.83	0.14	1.05	1.68	0.28	14.33	1.34	10.67	0.08	0.17	0.14	0.07	0.04	0.27	0.15	0.14	1.12
f__J045	2.29	3.04	0.03	1.82	2.07	0.14	3.16	2.10	1.51	0.09	0.08	0.07	0.05	0.03	0.06	0.03	0.08	0.41
f__JACPZX01	0.02	1.67	0.04	0.54	0.77	0.10	3.78	0.59	6.40	0.03	0.07	0.03	0.03	0.03	0.11	0.03	0.05	0.63
f__Polarisedimenticolaceae	0.67	14.89	0.21	5.68	6.36	0.69	20.42	5.66	3.61	0.11	0.34	0.14	0.41	0.27	0.43	0.14	0.31	0.45
f__QHYA01	0.01	0.62	0.02	0.09	0.08	0.04	1.40	0.18	7.74	0.01	0.04	0.03	0.00	0.01	0.22	0.03	0.03	1.04
f__SMYD01	0.00	0.23	0.01	1.07	1.30	0.00	0.31	0.40	0.78		0.03	0.01	0.04	0.03		0.04	0.04	1.02
f__2-12-FULL-54-10	0.40	5.36	0.18	0.94	1.68	0.50	17.86	1.79	10.01	0.05	0.13	0.05	0.06	0.05	0.22	0.07	0.10	0.70
f__20CM-2-55-15	1.08	8.13	0.54	3.85	5.51	1.09	60.86	3.68	16.52	0.17	0.21	0.37	0.05	0.12	0.37	0.38	0.23	1.69
f__2-02-FULL-67-57	0.10	2.00	0.02	0.97	0.15	0.79	1.72	0.88	1.96	0.03	0.54	0.12	0.32	0.01	0.79	0.21	0.45	0.47
f__UBA7541	1.17	6.68	0.50	0.94	2.76	0.56	55.20	2.39	23.06	0.48	0.70	1.47	0.04	0.09	1.99	2.67	0.99	2.70
f__Bryobacteraceae	19.75	31.00	1.20	4.93	10.80	3.65	97.33	16.20	6.01	0.40	0.69	1.16	0.11	0.17	0.77	2.27	0.65	3.50
f__JACQDG01	0.02	0.92	0.01	0.03	0.08	0.24	2.93	0.26	11.23	0.10	0.08	0.04	0.01	0.02	0.23	0.04	0.09	0.48
f__UBA6623	0.74	1.06	0.19	4.17	4.29	0.36	3.93	1.87	2.11	0.21	0.12	0.74	0.08	0.15	0.03	0.11	0.24	0.45
f__JAIQFV01	0.06	0.25	0.00	0.02	0.00	0.02	0.73	0.09	8.35	0.08	0.75		0.00		0.00	0.09	0.43	0.21
f__JAJPHL01	0.05	1.12	0.01	0.01	0.00	0.24	7.42	0.31	23.89	0.10	0.33	0.03	0.01		0.11	0.05	0.20	0.23
f__Acidobacteriaceae	2.56	9.03	0.84	1.19	4.06	1.96	67.87	3.68	18.45	1.89	1.36	3.31	0.02	0.44	1.46	3.59	2.13	1.69
f__CAINCZ01	0.00	0.07	0.00	0.00	0.00	0.00	0.27	0.02	16.37		0.01					0.02	0.01	1.50
f__DSPA01	0.04	0.12	0.07	0.00	0.00	0.12	5.05	0.06	89.75	0.05	0.06	0.03			0.03	0.03	0.04	0.69
f__Gp1-AA112	0.10	0.44	0.15	0.00	0.00	0.22	16.18	0.16	99.44	0.20	0.03	0.21			0.14	0.17	0.21	0.81
f__Gp1-AA117	0.32	1.55	0.30	0.06	0.15	0.34	33.27	0.54	61.83	0.19	0.23	0.39	0.00	0.04	0.24	0.35	0.42	0.85
f__JACPNR01	0.03	0.19	0.08	0.00	0.00	0.16	7.99	0.07	109.47	0.03	0.03	0.04			0.02	0.04	0.04	0.95
f__JAEZPW01	0.13	0.56	0.14	0.01	0.00	0.18	17.96	0.20	88.79	0.05	0.03	0.05	0.00		0.02	0.09	0.08	1.07
f__JAFAIN01	0.06	0.15	0.10	0.00	0.00	0.18	10.49	0.07	143.69	0.05	0.02	0.04			0.03	0.05	0.05	1.05
f__JAHFUA01	0.06	0.77	0.06	0.00	0.00	0.20	9.59	0.23	42.58	0.04	0.07	0.03			0.02	0.22	0.09	2.44
f__JAIQFD01	0.10	0.79	0.10	0.00	0.00	0.24	11.35	0.25	46.11	0.07	0.10	0.04			0.04	0.06	0.09	0.67
f__JAIQGF01	0.15	1.25	0.14	0.00	0.00	0.20	18.93	0.37	50.99	0.10	0.26	0.06			0.04	0.12	0.17	0.74
f__JAJPJE01	0.07	0.13	0.08	0.00	0.00	0.14	11.63	0.07	164.02	0.06	0.02	0.05			0.02	0.08	0.07	1.20
f__Koribacteraceae	0.74	2.59	0.38	0.19	0.00	0.46	46.68	0.97	48.04	0.34	0.33	0.30	0.01		0.08	0.57	0.34	1.66
f__QIAW01	0.01	0.07	0.06	0.00	0.00	0.10	5.32	0.03	169.98	0.09	0.01	0.06			0.02	0.05	0.06	0.92
f__SbA1	1.16	5.01	0.56	0.73	1.30	0.69	66.10	1.91	34.64	0.65	0.83	1.31	0.04	0.03	0.73	2.44	1.30	1.88
f__SCQP01	0.55	0.24	0.05	0.17	0.15	0.04	3.55	0.30	11.83	0.33	0.10	0.13	0.00	0.02	0.01	0.13	0.17	0.76
f__UBA7540	0.70	4.72	0.22	1.23	4.82	0.28	35.10	1.88	18.66	0.19	0.20	0.15	0.12	0.28	0.62	1.37	0.26	5.28
f__VXMN01	0.27	3.76	0.29	2.77	0.23	0.46	21.53	1.84	11.69	0.07	0.19	0.24	0.06	0.01	0.26	0.09	0.16	0.60
f__Gp7-AA8	0.75	6.60	0.58	1.04	2.99	1.85	56.08	2.40	23.34	0.15	0.25	0.40	0.03	0.04	0.41	0.44	0.29	1.53
f__JAHWKK01	0.00	0.09	0.01	0.00	0.00	0.10	0.70	0.03	22.46		0.01	0.13			0.01	0.09	0.03	3.12
f__Sulfomarinibacteraceae	0.53	4.45	0.03	5.32	10.41	0.73	3.24	3.14	1.03	0.23	0.41	0.19	0.48	0.59	1.45	0.19	0.46	0.41
f__Thermoanaerobaculaceae	1.54	4.36	0.06	1.04	2.45	0.04	9.27	1.93	4.79	0.47	0.73	0.06	0.04	0.04	0.01	0.21	0.37	0.55
f__UBA2201	0.32	2.21	0.01	4.07	3.83	0.65	2.92	1.99	1.47	0.07	0.05	0.05	0.01	0.00	0.01	0.02	0.05	0.43
f__Gp7-AA6	0.61	4.01	0.52	0.19	0.08	0.67	42.05	1.29	32.57	0.20	0.18	0.48	0.05	0.01	0.15	0.28	0.30	0.94
f__UBA5066	9.72	6.78	0.17	0.37	0.15	0.52	25.84	5.09	5.08	0.24	0.27	0.11	0.03	0.05	0.11	0.25	0.24	1.07
f__JAIOJS01	0.12	0.69	0.07	0.33	1.00	0.16	1.90	0.34	5.53	0.19	0.04	0.07	0.01	0.05	0.03	0.03	0.06	0.51
f__QQVD01	0.10	0.25	0.19	1.92	2.91	0.18	3.12	0.75	4.17	1.19	0.03	3.59	0.06	0.03	0.04	0.04	1.51	0.02
f__UBA5704	11.26	11.41	0.84	7.73	10.26	1.92	59.35	9.27	6.40	0.44	0.31	0.97	0.53	0.38	0.35	0.30	0.54	0.55

c__UBA4820	1.04	2.62	0.01	0.65	3.06	0.08	3.51	1.26	2.80	0.24	0.09	0.17	0.08	0.35	0.04	0.09	0.13	0.72	o__UBA4820	1.04	2.62	0.01	0.65	3.06	0.08	3.51	1.26	2.80	0.24	0.09	0.17	0.08	0.35	0.04	0.09	0.13	0.72	f__DSQY01	0.70	4.06	0.01	0.70	3.98	0.02	3.29	1.52	2.17	0.19	0.07	0.15	0.07	0.28	0.03	0.10	0.12	0.82
																																						f__UBA4820	0.55	0.39	0.00	0.03	0.00	0.02	0.41	0.29	1.41	0.24	0.10		0.04		0.03	0.02	0.16	0.12
c__UBA6911	4.25	9.16	0.38	8.53	9.42	1.27	29.48	6.48	4.55	0.25	0.25	0.20	0.15	0.19	0.30	0.22	0.24	0.91	o__CAINFC01	0.09	0.70	0.00	0.01	0.00	0.00	0.56	0.20	2.78	0.07	0.04	0.15	0.00	0.00	0.00	0.01	0.04	0.31	f__CAINFC01	0.09	0.70	0.00	0.01	0.00	0.00	0.56	0.20	2.78	0.07	0.04	0.15	0.00			0.01	0.04	0.31
																			o__JAAYUB01	1.45	1.26	0.02	0.70	0.54	0.00	1.22	1.01	1.21	0.51	0.27	0.13	0.02	0.01	0.00	0.02	0.30	0.08	f__JAAYUB01	1.45	1.26	0.02	0.70	0.54	0.00	1.22	1.01	1.21	0.51	0.27	0.13	0.02	0.01		0.02	0.30	0.08
																			o__JACOSC01	0.00	0.11	0.01	0.01	0.00	0.02	0.61	0.03	19.65	0.00	0.20	0.03	0.00	0.00	0.03	0.07	0.11	0.62	f__JACOSC01	0.00	0.11	0.01	0.01	0.00	0.02	0.61	0.03	19.65		0.20	0.03	0.00		0.03	0.07	0.11	0.62
																			o__JACQTF01	0.00	0.11	0.00	0.02	0.00	0.00	0.04	0.03	1.25	0.00	0.03	0.00	0.00	0.00	0.00	0.02	0.03	0.63	f__JACQTF01	0.00	0.11	0.00	0.02	0.00	0.00	0.04	0.03	1.25		0.03		0.00			0.02	0.03	0.63
																			o__RPQK01	0.45	2.66	0.11	7.18	7.27	0.34	6.42	3.10	2.07	0.05	0.07	0.21	0.12	0.09	0.05	0.07	0.12	0.54	f__JACQSX01	0.02	0.75	0.01	0.18	0.15	0.12	1.17	0.25	4.60	0.02	0.04	0.01	0.00	0.00	0.02	0.04	0.03	1.38
																																						f__RPQK01	0.40	1.48	0.06	6.83	7.04	0.16	3.75	2.67	1.40	0.05	0.10	0.34	0.13	0.09	0.08	0.09	0.14	0.63
																			o__UBA6911	2.19	11.68	0.23	1.23	5.28	0.73	21.78	4.10	5.31	0.19	0.24	0.19	0.20	0.17	0.46	0.25	0.26	0.96	f__UBA6911	2.19	11.68	0.23	1.23	5.28	0.73	21.78	4.10	5.31	0.19	0.24	0.19	0.20	0.17	0.46	0.25	0.26	0.96
c__UBA890	0.07	0.82	0.05	8.87	4.82	0.04	1.26	2.92	0.43	0.16	0.07	1.11	0.10	0.07	0.05	0.11	0.14	0.76	o__UBA890	0.07	0.82	0.05	8.87	4.82	0.04	1.26	2.92	0.43	0.16	0.07	1.11	0.10	0.07	0.05	0.11	0.14	0.76	f__UBA890	0.02	0.36	0.02	6.83	3.14	0.02	0.86	2.16	0.40	0.04	0.06	0.99	0.09	0.07	0.04	0.09	0.12	0.77
																																						f__WLXD01	0.04	0.19	0.01	2.12	2.76	0.00	0.60	0.75	0.81	0.15	0.04	2.06	0.05	0.03		0.06	0.14	0.46
c__G020349885	0.00	0.22	0.00	0.11	0.00	0.04	0.08	0.09	0.89		0.02		0.01		0.05	0.01	0.02	0.26	o__G020349885	0.00	0.22	0.00	0.11	0.00	0.04	0.08	0.09	0.89	0.00	0.02	0.00	0.01	0.00	0.05	0.01	0.02	0.26	f__G020349885	0.00	0.22	0.00	0.11	0.00	0.04	0.08	0.09	0.89		0.02		0.01		0.05	0.01	0.02	0.26
c__Vicinamibacteria	19.47	31.21	2.28	18.62	12.10	3.08	85.61	20.09	4.26	0.65	1.18	2.82	0.51	0.43	2.40	3.87	1.59	2.43	o__Bin61	0.15	1.98	0.19	4.01	4.36	0.18	4.21	1.82	2.32	0.41	0.07	0.67	0.10	0.10	0.12	0.08	0.21	0.38	f__Bin61	0.07	0.04	0.09	0.40	0.08	0.02	0.20	0.15	1.28	0.77	0.01	1.29	0.01	0.00	0.00	0.01	0.91	0.01
																																						f__SMYC01	0.04	1.37	0.08	3.17	0.31	0.12	3.28	1.28	2.56	0.09	0.08	0.15	0.11	0.10	0.16	0.09	0.11	0.86
																			o__Fen-336	8.90	11.61	0.84	3.21	6.89	1.71	54.64	7.09	7.71	0.30	0.47	0.81	0.17	0.26	0.29	0.45	0.52	0.87	f__Fen-336	5.55	10.05	0.81	3.05	7.89	1.63	53.65	5.55	9.67	0.26	0.47	0.81	0.17	0.26	0.28	0.43	0.51	0.85
																																						f__JAGNNV01	6.23	5.65	0.08	0.20	0.61	0.24	16.96	3.56	4.76	0.18	0.10	0.06	0.07	0.01	0.09	0.12	0.16	0.78
																			o__Vicinamibacterales	18.13	29.49	2.12	16.19	9.80	2.74	84.83	18.42	4.60	0.53	1.04	2.47	0.48	0.25	2.49	3.56	1.41	2.53	f__2-12-FULL-66-21	6.85	12.92	0.99	2.51	6.58	2.30	74.82	6.55	11.42	0.27	0.29	0.68	0.08	0.07	0.60	0.74	0.41	1.83
																																						f__Fen-181	1.33	8.08	0.33	0.55	0.38	1.07	34.15	2.66	12.85	0.08	0.17	0.13	0.02	0.02	0.04	0.11	0.14	0.78
																																						f__JACPPN01	0.01	0.67	0.11	0.04	0.08	0.16	6.89	0.19	35.52	0.02	0.02	0.02	0.00	0.00	0.02	0.02	0.02	1.17
																																						f__JACQTH01	0.15	2.02	0.12	0.11	0.23	0.16	10.52	0.59	17.95	0.02	0.04	0.02	0.00	0.01	0.02	0.02	0.03	0.84
																																						f__JAFNAJ01	3.75	4.82	0.36	0.61	1.07	0.93	26.85	2.73	9.85	0.10	0.07	0.07	0.01	0.03	0.05	0.06	0.09	0.68
																																						f__SCN-69-37	6.84	11.81	0.67	1.26	4.29	1.49	56.92	5.78	9.85	0.16	0.21	0.29	0.08	0.10	0.34	0.32	0.26	1.24
																																						f__UBA2999	13.68	24.46	1.66	7.84	6.74	2.40	82.17	13.18	6.24	0.45	0.86	1.82	0.12	0.16	1.77	2.45	1.08	2.26
																																						f__UBA823	0.03	0.82	0.11	7.07	0.31	0.22	7.50	2.29	3.28	0.01	0.02	0.05	0.17	0.02	0.01	0.02	0.13	0.17
																																						f__UBA8438	0.18	3.11	0.32	11.47	1.91	0.56	18.43	4.23	4.36	0.58	0.03	0.72	0.35	0.04	0.03	0.04	0.32	0.13
																																						f__Vicinamibacteraceae	2.03	4.00	0.47	0.61	0.54	1.47	24.76	1.99	12.45	0.09	0.06	0.33	0.01	0.05	0.09	0.10	0.15	0.66

Table S3. Numbers and percenatges of habitat-generalist taxa in the Acidobacteriota at different taxonomic levels and using several delineation criteria.

		Phylum	Class	Order	Family	Genus
Results using occurrence in metagenomic datasets						
Using all taxa	Total number of taxa	1	14	52	102	
	Number of generalist taxa[a]	1	11	32	54	
	Percentage of total	100	78.6	61.5	52.9	
Using only taxa identified in [illegible]	Total number of taxa	1	12	40	78	
	Number of generalist taxa[a]	1	11	31	52	
	Percentage of total	100	91.7	77.5	66.7	
Results using distribution of genomes in GTDB						
Using all taxa	Total number of taxa	1	14	52	102	486
	Number of generalist taxa[b]	1	12	34	56	120
	Percentage of total	100	85.71	65.38	54.90	24.69
Using taxa with at least 5 genomes	Total number of taxa	1	12	37	54	82
	Number of generalist taxa[b]	1	11	32	47	51
	Percentage of total	100	91.67	86.49	87.04	62.20

a: reads identified in the 7 different habitats

b: Genomes obtained from more than one habitat

Table S4. Genomes compared in this study, their Acidobacteriota class, lineage preference to soil (SPL vs NSPL), habitat from which the genome was recovered, and various genomic features compared.

NCBI accession	GTDB taxonomy			Lineage	Habitat	General genomic features					Phage infection/immunity		Extracellular product arsenal			Predicted physiological optima			Life history
number	Class	order	Family	preference to soil		Coding density	Percentage GC	Genome size (bp)	Protein count	Average gene length	CRISPRs	Viral contigs	BGCs	CAZYmes	Peptidases	Predicted OGT	Predicted pH	Predicted O2 preference	strategy
GCA_000402595	c__Aminicenantia	o__Aminicenantales	f__Aminicenantaceae	NSPL	Freshwater	87.96	39.25	2970774	2734	955.38	45	1	0	32	28	44.17	6.24	Anaerobic	Ruderal
GCA_000402635	c__Aminicenantia	o__Aminicenantales	f__Aminicenantaceae	NSPL	Soil	88.16	39.04	2494861	2391	917.48	33	0	4	29	25	45.54	6.24	Anaerobic	Competitor
GCA_000493965	c__Aminicenantia	o__Aminicenantales	f__Aminicenantaceae	NSPL	Freshwater	88.00	39.27	2913626	2671	959.80	44	1	0	32	25	44.25	6.24	Anaerobic	Scarcity
GCA_000493985	c__Aminicenantia	o__Aminicenantales	f__Aminicenantaceae	NSPL	Freshwater	88.27	39.08	2405692	2279	930.52	31	0	4	28	25	45.36	6.24	Anaerobic	Competitor
GCA_001773855	c__Aminicenantia	o__Aminicenantales	f__RBG_16_66_30	NSPL	Freshwater	82.79	63.73	1792761	1712	961.23	20	3	4	32	25	40.74	6.34	Anaerobic	Scarcity
GCA_001773875	c__Aminicenantia	o__Aminicenantales	f__RBG_16_66_30	NSPL	Freshwater	85.81	63.95	2388421	2209	997.70	22	0	0	34	28	39.35	6.23	Anaerobic	Scarcity
GCA_001775715	c__Aminicenantia	o__Aminicenantales	f__RBG_16_66_30	NSPL	Freshwater	92.01	65.98	2075472	1896	1018.40	21	1	4	36	22	41.14	5.90	Anaerobic	Scarcity
GCA_001775755	c__Aminicenantia	o__Aminicenantales	f__RBG_16_66_30	NSPL	Freshwater	91.38	64.05	2453444	2185	1047.04	29	2	0	42	22	40.46	6.25	Anaerobic	Scarcity
GCA_002010665	c__Aminicenantia	o__JdFR_78	f__JdFR_78	NSPL	Marine	93.93	32.60	2530072	2388	997.34	49	2	4	18	28	66.74	6.13	Anaerobic	Ruderal
GCA_002010715	c__Aminicenantia	o__JdFR_78	f__JdFR_78	NSPL	Marine	94.25	32.64	2046026	1935	998.30	42	0	0	14	28	67.57	6.16	Anaerobic	Scarcity
GCA_002010725	c__Aminicenantia	o__Aminicenantales	f__JdFR_80	NSPL	Marine	89.39	44.60	2914703	2545	1023.51	36	3	8	24	29	50.79	5.64	Anaerobic	Scarcity
GCA_002070465	c__Aminicenantia	o__Aminicenantales	f__UBA4085	NSPL	Engineered	91.32	62.88	3054023	2862	975.07	24	2	0	31	28	39.18	6.34	Anaerobic	Scarcity
GCA_002294665	c__Aminicenantia	o__UBA2199	f__UBA2199	NSPL	Freshwater	91.13	56.77	1927288	1788	982.14	21	1	16	6	20	40.79	5.29	Anaerobic	Scarcity
GCA_002299555	c__Aminicenantia	o__UBA2199	f__UBA2199	NSPL	Freshwater	92.24	37.77	2656053	2445	1012.63	32	0	4	14	24	32.45	6.28	Anaerobic	Scarcity
GCA_002316315	c__Aminicenantia	o__Aminicenantales	f__Saccharicenantaceae	NSPL	Engineered	89.51	45.74	2510304	2116	1061.29	22	0	0	42	31	41.23	5.96	Anaerobic	Scarcity
GCA_002327965	c__Aminicenantia	o__UBA2199	f__UBA2199	NSPL	Engineered	89.59	62.18	2513533	2265	1002.85	24	1	8	13	19	37.2	5.60	Anaerobic	Scarcity
GCA_002328685	c__Aminicenantia	o__Aminicenantales	f__Saccharicenantaceae	NSPL	Engineered	90.67	47.40	2374313	2086	1033.12	24	1	0	47	35	38.46	6.12	Anaerobic	Scarcity
GCA_002335385	c__Aminicenantia	o__UBA2199	f__UBA2199	NSPL	Engineered	90.64	62.76	2953209	2584	1037.86	23	1	14	21	28	35.12	6.03	Anaerobic	Scarcity
GCA_002383325	c__Aminicenantia	o__Aminicenantales	f__UBA4085	NSPL	Engineered	91.47	65.29	3491420	3079	1038.37	28	0	16	95	30	35.59	6.99	Anaerobic	Scarcity
GCA_002436105	c__Aminicenantia	o__UBA2199	f__UBA2199	NSPL	Engineered	90.64	62.56	3116808	2651	1067.55	28	0	12	20	25	35.85	6.03	Anaerobic	Scarcity
GCA_002441005	c__Aminicenantia	o__Aminicenantales	f__Saccharicenantaceae	NSPL	Engineered	90.87	47.19	2456980	2070	1080.59	22	0	0	45	35	39.4	6.10	Anaerobic	Scarcity
GCA_002877915	c__Aminicenantia	o__Aminicenantales	f__Saccharicenantaceae	NSPL	Freshwater	88.35	43.29	2550048	2295	981.77	31	0	0	45	28	49.82	6.21	Anaerobic	Scarcity
GCA_003141555	c__Aminicenantia	o__Aminicenantales	f__UBA4085	NSPL	Soil	92.04	62.83	3792977	3192	1094.77	31	3	4	85	38	37.2	6.54	Anaerobic	Scarcity
GCA_003153725	c__Aminicenantia	o__Aminicenantales	f__UBA4085	NSPL	Soil	89.23	61.25	3737067	3619	930.60	29	0	20	113	42	33.56	6.82	Anaerobic	Scarcity
GCA_003154255	c__Aminicenantia	o__Aminicenantales	f__UBA4085	NSPL	Soil	89.00	61.49	3482832	3424	918.65	28	0	8	107	44	34.67	6.09	Anaerobic	Scarcity
GCA_003426165	c__Aminicenantia	o__Aminicenantales	f__Saccharicenantaceae	NSPL	Freshwater	88.54	54.52	2899266	2304	1115.27	27	1	0	57	32	49.9	6.17	Anaerobic	Scarcity
GCA_003476685	c__Aminicenantia	o__Aminicenantales	f__Saccharicenantaceae	NSPL	Engineered	88.04	53.70	1933857	1824	935.89	25	0	0	31	30	45.69	6.04	Anaerobic	Scarcity
GCA_003485065	c__Aminicenantia	o__Aminicenantales	f__Saccharicenantaceae	NSPL	Engineered	90.79	47.48	1641452	1687	882.97	25	0	0	33	23	38.97	6.12	Anaerobic	Scarcity
GCA_003524335	c__Aminicenantia	o__Aminicenantales	f__UBA8522	NSPL	Freshwater	90.10	58.43	3507216	3610	877.78	35	3	0	55	52	42.3	5.78	Anaerobic	Scarcity
GCA_003648765	c__Aminicenantia	o__Aminicenantales	f__JdFR_80	NSPL	Marine	86.14	43.45	2767995	2401	991.70	28	0	0	24	30	49.73	5.70	Anaerobic	Scarcity
GCA_004377005	c__Aminicenantia	o__Aminicenantales	f__Aminicenantaceae	NSPL	Marine	90.67	42.06	1931870	1765	988.50	23	0	0	11	21	50.04	6.22	Anaerobic	Scarcity
GCA_004378665	c__Aminicenantia	o__Aminicenantales	f__Aminicenantaceae	NSPL	Marine	92.55	47.97	2049380	1907	993.32	28	0	4	22	21	46.01	5.83	Anaerobic	Scarcity
GCA_004525465	c__Aminicenantia	o__UBA2199	f__UBA2199	NSPL	Marine	92.00	54.25	2746428	2770	912.46	25	1	0	17	30	32.62	6.27	Anaerobic	Scarcity
GCA_011042725	c__Aminicenantia	o__Aminicenantales	f__JdFR_80	NSPL	Marine	86.56	42.76	2558371	2359	938.25	29	0	8	24	33	46.5	5.70	Anaerobic	Scarcity
GCA_011048485	c__Aminicenantia	o__Aminicenantales	f__RBG_16_66_30	NSPL	Freshwater	92.68	66.47	2045217	2033	933.45	30	1	4	38	24	40.06	6.53	Anaerobic	Scarcity
GCA_011049425	c__Aminicenantia	o__UBA2199	f__UBA2199	NSPL	Freshwater	90.93	55.61	2623831	2432	982.67	38	1	12	15	25	40.44	5.80	Anaerobic	Scarcity
GCA_011049545	c__Aminicenantia	o__Aminicenantales	f__Aminicenantaceae	NSPL	Marine	87.48	41.53	3235875	2927	967.46	42	3	8	10	31	46.75	6.32	Anaerobic	Scarcity
GCA_011055285	c__Aminicenantia	o__Aminicenantales	f__Saccharicenantaceae	NSPL	Freshwater	88.54	43.49	2516061	2210	1008.52	33	0	0	47	29	49.76	6.21	Anaerobic	Scarcity
GCA_011771225	c__Aminicenantia	o__UBA2199	f__UBA2199	NSPL	Marine	85.53	43.68	10499685	8379	1070.73	117	1	574	39	52	44.1	5.00	Anaerobic	Scarcity
GCA_011771485	c__Aminicenantia	o__UBA2199	f__UBA2199	NSPL	Marine	85.74	43.68	10419537	8312	1074.69	117	1	574	39	51	44.22	5.00	Anaerobic	Competitor
GCA_011771685	c__Aminicenantia	o__UBA2199	f__UBA2199	NSPL	Marine	85.55	43.66	10513167	8389	1070.84	119	1	582	39	51	44.17	5.00	Anaerobic	Competitor
GCA_011771905	c__Aminicenantia	o__UBA2199	f__UBA2199	NSPL	Marine	85.61	43.67	10495492	8377	1071.98	118	1	586	39	51	44.08	5.00	Anaerobic	Competitor
GCA_011772425	c__Aminicenantia	o__UBA2199	f__UBA2199	NSPL	Marine	86.04	43.66	10383395	8274	1085.00	115	1	586	39	51	44.33	4.88	Anaerobic	Scarcity
GCA_011772475	c__Aminicenantia	o__Aminicenantales	f__Aminicenantaceae	NSPL	Marine	87.02	42.26	3142456	2838	965.02	39	2	8	11	25	50.36	6.22	Anaerobic	Scarcity
GCA_011772495	c__Aminicenantia	o__Aminicenantales	f__Aminicenantaceae	NSPL	Marine	89.30	44.30	3276286	2824	1036.78	35	1	12	22	30	47.92	5.73	Anaerobic	Scarcity
GCA_011773465	c__Aminicenantia	o__Aminicenantales	f__Aminicenantaceae	NSPL	Marine	86.35	41.50	2591933	2290	979.20	41	1	8	10	24	52.41	6.30	Anaerobic	Scarcity
GCA_012516875	c__Aminicenantia	o__Aminicenantales	f__RBG_16_66_30	NSPL	Engineered	89.62	63.82	4027175	3715	969.64	38	13	8	81	31	36.67	6.43	Anaerobic	Scarcity
GCA_012797375	c__Aminicenantia	o__Aminicenantales	f__UBA4085	NSPL	Engineered	91.69	62.92	2405437	2449	900.86	20	0	0	19	35	39.54	6.19	Anaerobic	Scarcity
GCA_012797795	c__Aminicenantia	o__Aminicenantales	f__Saccharicenantaceae	NSPL	Engineered	91.30	47.53	2235898	2139	954.18	25	0	0	44	36	38.43	6.10	Anaerobic	Scarcity
GCA_013139435	c__Aminicenantia	o__Aminicenantales	f__Aminicenantaceae	NSPL	Marine	90.43	46.46	2613071	2569	919.84	37	0	0	18	29	41.72	5.81	Anaerobic	Scarcity
GCA_013178315	c__Aminicenantia	o__Aminicenantales	f__Saccharicenantaceae	NSPL	Freshwater	88.76	54.30	2941401	2451	1065.78	30	1	4	56	35	49.18	6.17	Anaerobic	Scarcity
GCA_014359965	c__Aminicenantia	o__Aminicenantales	f__Saccharicenantaceae	NSPL	Engineered	88.66	45.54	2633990	2335	999.11	39	2	0	57	36	54.64	6.17	Anaerobic	Scarcity
GCA_014360005	c__Aminicenantia	o__Aminicenantales	f__Saccharicenantaceae	NSPL	Engineered	88.35	52.26	2906650	2403	1067.94	33	1	0	58	35	46.91	6.21	Anaerobic	Scarcity
GCA_014360015	c__Aminicenantia	o__Aminicenantales	f__Saccharicenantaceae	NSPL	Engineered	88.72	45.05	2722800	2322	1041.90	40	1	4	39	27	54.89	6.04	Anaerobic	Competitor
GCA_014727835	c__Aminicenantia	o__Aminicenantales	f__Aminicenantaceae	NSPL	Non_marine	92.95	44.16	3135038	2777	1049.65	46	0	8	37	32	34.6	6.49	Anaerobic	Scarcity
GCA_014859315	c__Aminicenantia	o__Aminicenantales	f__Aminicenantaceae	NSPL	Marine	90.44	39.20	2061027	1880	990.93	34	0	4	24	25	50.14	6.48	Anaerobic	Competitor
GCA_014894455	c__Aminicenantia	o__Aminicenantales	f__RBG_16_66_30	NSPL	Soil	90.13	62.88	2949287	2636	1024.57	37	4	4	63	30	39.41	6.37	Anaerobic	Scarcity
GCA_015486055	c__Aminicenantia	o__S016_38	f__S016_38	NSPL	Marine	95.16	40.36	1861152	1793	992.58	28	0	4	13	14	63.6	6.20	Anaerobic	Scarcity
GCA_016783445	c__Aminicenantia	o__Aminicenantales	f__Aminicenantaceae	NSPL	Freshwater	88.14	40.53	2894707	2508	1017.91	37	0	0	33	27	45.43	6.20	Anaerobic	Scarcity
GCA_016867585	c__Aminicenantia	o__Aminicenantales	f__UBA4085	NSPL	Freshwater	93.11	64.36	3450504	3268	983.52	36	1	0	51	45	41.86	6.09	Anaerobic	Scarcity
GCA_016867615	c__Aminicenantia	o__Aminicenantales	f__JAFGAV01	NSPL	Freshwater	91.47	64.91	2441500	2568	870.45	32	0	16	21	35	40.76	6.13	Anaerobic	Scarcity
GCA_016867635	c__Aminicenantia	o__Aminicenantales	f__RBG_16_66_30	NSPL	Freshwater	92.52	65.64	2664845	2546	968.87	33	0	8	38	39	39.99	5.58	Anaerobic	Scarcity
GCA_016926835	c__Aminicenantia	o__UBA2199	f__UBA2199	NSPL	Freshwater	92.70	55.57	2049147	1979	960.96	30	0	8	10	20	39.84	6.04	Anaerobic	Scarcity
GCA_016926925	c__Aminicenantia	o__Aminicenantales	f__Aminicenantaceae	NSPL	Freshwater	89.78	48.65	4326490	3858	1007.03	62	2	4	14	49	37.14	5.54	Anaerobic	Scarcity
GCA_016927475	c__Aminicenantia	o__Aminicenantales	f__Aminicenantaceae	NSPL	Freshwater	90.21	45.82	4989209	4094	1100.28	61	1	54	41	37	40.66	5.90	Anaerobic	Competitor
GCA_016931595	c__Aminicenantia	o__Aminicenantales	f__UBA4085	NSPL	Freshwater	93.90	61.59	2931239	2666	1033.61	29	0	8	32	27	35.18	6.52	Anaerobic	Ruderal
GCA_016933355	c__Aminicenantia	o__Aminicenantales	f__JAFGBC01	NSPL	Freshwater	93.96	66.69	2053444	1976	977.07	16	0	4	33	21	40.95	6.54	Anaerobic	Scarcity
GCA_016933515	c__Aminicenantia	o__Aminicenantales	f__JAFGAV01	NSPL	Freshwater	91.79	62.21	2374780	2134	1021.14	29	0	12	17	21	40.71	6.03	Anaerobic	Scarcity
GCA_016933885	c__Aminicenantia	o__Aminicenantales	f__RBG_16_66_30	NSPL	Freshwater	93.09	66.75	2657141	2653	934.18	29	0	4	47	38	39.22	6.18	Anaerobic	Scarcity
GCA_016934475	c__Aminicenantia	o__UBA2199	f__UBA2199	NSPL	Freshwater	91.99	63.72	2570893	2482	954.31	24	0	8	15	29	42.35	5.96	Anaerobic	Scarcity
GCA_016934745	c__Aminicenantia	o__UBA2199	f__UBA2199	NSPL	Freshwater	93.19	51.37	2208932	2080	990.90	25	0	8	12	17	37.22	6.34	Anaerobic	Scarcity
GCA_016934895	c__Aminicenantia	o__Aminicenantales	f__UBA8522	NSPL	Freshwater	91.95	58.91	2127094	2026	965.44	16	1	0	28	28	45.71	6.22	Anaerobic	Scarcity
GCA_017883285	c__Aminicenantia	o__Aminicenantales	f__UBA4085	NSPL	Soil	92.20	63.19	3085923	2772	1026.25	29	0	4	40	33	38.84	6.23	Anaerobic	Scarcity
GCA_017883305	c__Aminicenantia	o__Aminicenantales	f__RBG_16_66_30	NSPL	Soil	93.35	64.45	2932371	2741	999.04	26	0	4	87	35	34.17	6.81	Anaerobic	Scarcity
GCA_017886205	c__Aminicenantia	o__Aminicenantales	f__UBA8522	NSPL	Freshwater	91.14	57.98	3516080	3063	1046.21	36	2	0	73	33	43.61	6.38	Anaerobic	Scarcity
GCA_017992175	c__Aminicenantia	o__Aminicenantales	f__Saccharicenantaceae	NSPL	Engineered	91.74	47.94	1382151	1180	1077.33	10	0	0	24	19	37.35	6.10	Anaerobic	Scarcity
GCA_017999365	c__Aminicenantia	o__Aminicenantales	f__RBG_16_66_30	NSPL	Engineered	93.26	68.60	2817073	2423	1085.72	25	2	4	64	27	40.31	6.34	Anaerobic	Scarcity
GCA_018052465	c__Aminicenantia	o__Aminicenantales	f__Saccharicenantaceae	NSPL	Engineered	89.69	45.52	2358053	1989	1063.25	24	2	0	38	30	41.46	5.96	Anaerobic	Scarcity
GCA_018262835	c__Aminicenantia	o__UBA2199	f__UBA2199	NSPL	Freshwater	91.75	56.89	2728240	2538	987.65	19	0	12	17	19	34.06	5.46	Anaerobic	Scarcity
GCA_018335295	c__Aminicenantia	o__Aminicenantales	f__Aminicenantaceae	NSPL	Freshwater	89.20	43.77	2537978	2301	987.55	42	0	4	15	28	42.68	6.59	Anaerobic	Competitor
GCA_018819045	c__Aminicenantia	o__Aminicenantales	f__Aminicenantaceae	NSPL	Freshwater	92.42	53.79	2273784	2210	950.11	25	0	4	23	25	36.56	5.69	Anaerobic	Scarcity
GCA_018820345	c__Aminicenantia	o__Aminicenantales	f__Aminicenantaceae	NSPL	Freshwater	92.11	53.48	2739089	2678	940.90	38	0	4	29	35	36.6	5.57	Anaerobic	Scarcity
GCA_018821265	c__Aminicenantia	o__Aminicenantales	f__Aminicenantaceae	NSPL	Freshwater	92.26	53.86	2692487	2623	947.24	33	0	4	30	32	35.79	5.57	Anaerobic	Scarcity
GCA_018894615	c__Aminicenantia	o__Aminicenantales	f__Aminicenantaceae	NSPL	Freshwater	91.59	53.28	2953228	2791	970.47	36	1	4	30	33	35.6	5.57	Anaerobic	Scarcity

GCA_018895915	c__Aminicenantia	o__UBA2199	f__UBA2199	NSPL	Freshwater	90.01	53.20	2098670	1998	947.17	19	0	4	13	23	37.06	6.40	Anaerobic	Scarcity
GCA_018896615	c__Aminicenantia	o__Aminicenantales	f__Aminicenantaceae	NSPL	Freshwater	91.95	53.47	2892141	2708	981.85	38	0	4	29	33	35.99	5.99	Anaerobic	Scarcity
GCA_018897215	c__Aminicenantia	o__UBA2199	f__UBA2199	NSPL	Freshwater	89.41	51.29	2107638	1949	966.70	21	0	4	11	19	38.77	5.87	Anaerobic	Scarcity
GCA_018897435	c__Aminicenantia	o__UBA2199	f__UBA2199	NSPL	Freshwater	89.39	51.55	2191297	2022	969.11	22	0	4	13	20	38.65	5.87	Anaerobic	Scarcity
GCA_018897505	c__Aminicenantia	o__Aminicenantales	f__Aminicenantaceae	NSPL	Freshwater	92.10	53.54	2868411	2745	962.72	40	0	4	29	35	36.23	5.99	Anaerobic	Scarcity
GCA_020344415	c__Aminicenantia	o__Aminicenantales	f__Aminicenantaceae	NSPL	Freshwater	84.60	41.90	4431024	3713	1023.15	50	0	8	24	39	47.06	6.29	Anaerobic	Scarcity
GCA_020348385	c__Aminicenantia	o__Aminicenantales	f__Aminicenantaceae	NSPL	Freshwater	90.88	46.30	4351283	3647	1095.31	44	0	4	55	37	35.31	6.25	Anaerobic	Scarcity
GCA_020355945	c__Aminicenantia	o__UBA2199	f__UBA2199	NSPL	Freshwater	74.62	42.84	9131798	6411	1232.02	83	0	675	33	40	42.17	6.20	Anaerobic	Competitor
GCA_020717815	c__Aminicenantia	o__Aminicenantales	f__Saccharicenantaceae	NSPL	Freshwater	88.71	54.63	2852822	2280	1111.13	27	1	0	57	32	49.75	6.17	Anaerobic	Scarcity
GCA_020853335	c__Aminicenantia	o__Aminicenantales	f__UBA4085	NSPL	Engineered	91.58	62.98	2722650	2525	988.91	20	0	0	22	32	38.64	6.19	Anaerobic	Scarcity
GCA_021157535	c__Aminicenantia	o__Aminicenantales	f__JdFR_80	NSPL	Marine	85.64	43.07	3244585	2848	974.78	33	5	12	25	30	51.28	5.70	Anaerobic	Ruderal
GCA_021161165	c__Aminicenantia	o__S016_38	f__S016_38	NSPL	Marine	95.77	39.61	1468488	1527	924.84	26	0	0	10	16	71.45	6.27	Anaerobic	Scarcity
GCA_021162615	c__Aminicenantia	o__Aminicenantales	f__Aminicenantaceae	NSPL	Marine	89.98	42.85	2853261	2566	1000.05	33	1	20	32	30	39.73	5.88	Anaerobic	Competitor
GCA_021817755	c__Aminicenantia	o__UBA2199	f__UBA2199	NSPL	Engineered	90.90	38.15	4744584	4195	1027.45	66	1	348	28	30	37.1	6.36	Anaerobic	Scarcity
GCA_022013915	c__Aminicenantia	o__UBA2199	f__UBA2199	NSPL	Freshwater	89.10	51.66	3459119	3242	949.47	32	1	8	17	28	38.47	6.13	Anaerobic	Scarcity
GCA_022013925	c__Aminicenantia	o__Aminicenantales	f__Aminicenantaceae	NSPL	Freshwater	91.16	52.95	3298910	3184	944.07	41	2	4	34	34	36.09	5.99	Anaerobic	Scarcity
GCA_022709005	c__Aminicenantia	o__Aminicenantales	f__UBA4085	NSPL	Engineered	89.49	63.08	2708546	2501	998.28	22	0	0	23	29	39.18	6.19	Anaerobic	Scarcity
GCA_022711665	c__Aminicenantia	o__Aminicenantales	f__RBG_16_66_30	NSPL	Engineered	92.61	67.35	3253509	2646	1139.92	27	0	8	65	31	39.55	6.43	Anaerobic	Scarcity
GCA_022711715	c__Aminicenantia	o__UBA2199	f__UBA2199	NSPL	Engineered	89.92	66.23	2090247	1929	975.87	17	0	0	17	15	40.42	6.14	Anaerobic	Scarcity
GCA_022865785	c__Aminicenantia	o__UBA2199	f__UBA2199	NSPL	Marine	91.00	53.16	3054272	2971	935.93	26	1	20	16	31	34.22	5.86	Anaerobic	Scarcity
GCA_022866045	c__Aminicenantia	o__Aminicenantales	f__UBA4085	NSPL	Marine	89.15	55.24	3276775	3054	955.72	41	1	4	48	41	43.7	6.34	Anaerobic	Scarcity
GCA_022867105	c__Aminicenantia	o__Aminicenantales	f__RBG_16_66_30	NSPL	Marine	91.99	59.99	2486546	2569	891.23	30	4	4	32	43	43.95	6.22	Anaerobic	Scarcity
GCA_022867325	c__Aminicenantia	o__Aminicenantales	f__JAFGBC01	NSPL	Marine	90.19	61.26	3731918	3561	944.72	31	1	12	77	44	40.99	6.30	Anaerobic	Scarcity
GCA_023133225	c__Aminicenantia	o__Aminicenantales	f__Aminicenantaceae	NSPL	Marine	89.91	42.95	3084722	2747	1008.03	45	2	4	32	33	44.73	5.84	Anaerobic	Scarcity
GCA_023135565	c__Aminicenantia	o__UBA2199	f__UBA2199	NSPL	Marine	87.01	45.53	7622728	6369	1038.00	81	4	215	28	29	41.41	5.24	Anaerobic	Scarcity
GCA_023135995	c__Aminicenantia	o__Aminicenantales	f__Aminicenantaceae	NSPL	Marine	90.58	46.99	2802604	2590	976.59	37	2	0	26	33	40.03	6.49	Anaerobic	Scarcity
GCA_023136725	c__Aminicenantia	o__UBA2199	f__UBA2199	NSPL	Marine	94.46	37.69	2245325	2391	887.75	37	0	12	14	25	34.72	5.63	Anaerobic	Competitor
GCA_023137075	c__Aminicenantia	o__UBA2199	f__UBA2199	NSPL	Marine	91.06	44.14	5237052	4657	1022.37	63	1	236	22	37	42.53	5.59	Anaerobic	Scarcity
GCA_023621955	c__Aminicenantia	o__JAMOAD01	f__JAMOAD01	NSPL	Engineered	93.78	58.33	2344528	2371	929.05	31	0	0	16	27	45.19	6.00	Anaerobic	Scarcity
GCA_024178865	c__Aminicenantia	o__JdFR_78	f__JdFR_78	NSPL	Marine	93.82	32.72	2482978	2417	965.03	46	2	4	18	34	67.29	6.13	Anaerobic	Ruderal
GCA_024179005	c__Aminicenantia	o__JdFR_78	f__JdFR_78	NSPL	Marine	94.67	32.95	1549968	1510	973.19	32	1	4	14	20	68.99	6.05	Anaerobic	Scarcity
GCA_024179025	c__Aminicenantia	o__JdFR_78	f__JdFR_78	NSPL	Marine	94.63	33.05	1411864	1397	957.71	32	1	4	13	22	68.55	6.42	Anaerobic	Competitor
GCA_024644125	c__Aminicenantia	o__UBA2199	f__UBA2199	NSPL	Freshwater	92.77	49.26	5714171	4765	1115.05	46	1	859	29	32	38.16	6.11	Anaerobic	Scarcity
GCA_024653215	c__Aminicenantia	o__Aminicenantales	f__Saccharicenantaceae	NSPL	Engineered	88.97	54.44	2604269	2128	1090.14	26	0	0	50	32	49.66	6.17	Anaerobic	Scarcity
GCA_024653405	c__Aminicenantia	o__Aminicenantales	f__Saccharicenantaceae	NSPL	Engineered	88.18	53.74	2310725	2258	902.25	26	0	0	45	34	45.37	6.17	Anaerobic	Scarcity
GCA_000226295	c__Blastocatellia	o__Chloracidobacteriales	f__Chloracidobacteriaceae	SPL	Freshwater	90.89	61.34	3695372	3051	1102.74	34	0	28	19	53	42.77	6.82	Aerobic	Scarcity
GCA_000820845	c__Blastocatellia	o__Pyrinomonadales	f__Pyrinomonadaceae	SPL	Soil	88.39	59.37	3788560	3189	1050.74	36	1	8	83	44	58.94	6.16	Aerobic	Competitor
GCA_001464455	c__Blastocatellia	o__Pyrinomonadales	f__Pyrinomonadaceae	SPL	Engineered	91.19	53.13	4200469	3782	1014.18	35	3	16	50	44	23.18	6.49	Aerobic	Scarcity
GCA_001464665	c__Blastocatellia	o__Pyrinomonadales	f__Pyrinomonadaceae	SPL	Engineered	90.83	55.14	3964155	3694	976.20	35	2	16	23	37	24.06	6.90	Aerobic	Scarcity
GCA_001482755	c__Blastocatellia	o__Chloracidobacteriales	f__Chloracidobacteriaceae	SPL	Freshwater	91.51	62.42	3600358	2944	1120.94	31	0	32	18	52	41.57	6.82	Aerobic	Scarcity
GCA_001567505	c__Blastocatellia	o__Pyrinomonadales	f__Pyrinomonadaceae	SPL	Engineered	90.56	56.31	2971784	2967	909.23	34	2	4	22	42	30.55	7.00	Aerobic	Scarcity
GCA_001920415	c__Blastocatellia	o__Pyrinomonadales	f__Pyrinomonadaceae	SPL	Soil	85.07	52.89	6102709	5524	941.79	62	10	37	40	55	32.24	6.41	Aerobic	Competitor
GCA_002050355	c__Blastocatellia	o__Pyrinomonadales	f__Pyrinomonadaceae	SPL	Soil	88.49	45.70	3652584	3528	917.00	38	3	12	32	43	30.26	6.67	Aerobic	Scarcity
GCA_002050365	c__Blastocatellia	o__Pyrinomonadales	f__Pyrinomonadaceae	SPL	Soil	87.96	45.41	4149107	4127	886.56	34	0	8	32	48	31.12	6.45	Aerobic	Scarcity
GCA_002050455	c__Blastocatellia	o__Pyrinomonadales	f__Pyrinomonadaceae	SPL	Soil	88.71	44.29	3211011	3223	884.06	37	3	12	22	38	33	7.05	Aerobic	Scarcity
GCA_002254845	c__Blastocatellia	o__Chloracidobacteriales	f__Chloracidobacteriaceae	SPL	Freshwater	90.22	64.18	3411091	3078	1000.03	24	1	28	19	57	39.82	6.93	Aerobic	Scarcity
GCA_002333435	c__Blastocatellia	o__Pyrinomonadales	f__Pyrinomonadaceae	SPL	Engineered	90.52	53.18	3749176	3513	974.06	34	1	12	25	40	22.83	6.83	Aerobic	Scarcity
GCA_002344645	c__Blastocatellia	o__Pyrinomonadales	f__Pyrinomonadaceae	SPL	Engineered	90.78	53.37	3443216	3199	986.60	30	1	12	24	41	23.52	6.86	Aerobic	Scarcity
GCA_002360555	c__Blastocatellia	o__Pyrinomonadales	f__Pyrinomonadaceae	SPL	Engineered	91.58	51.94	3358442	3165	981.19	34	0	16	28	37	22.86	6.50	Aerobic	Scarcity
GCA_002384175	c__Blastocatellia	o__Pyrinomonadales	f__Pyrinomonadaceae	SPL	Soil	85.90	53.19	4904582	4636	910.56	53	2	24	36	49	33.02	6.26	Aerobic	Scarcity
GCA_002404175	c__Blastocatellia	o__Pyrinomonadales	f__Pyrinomonadaceae	SPL	Soil	81.57	54.33	4992344	4693	929.31	44	1	25	73	43	31.29	6.32	Aerobic	Scarcity
GCA_002427845	c__Blastocatellia	o__Pyrinomonadales	f__Pyrinomonadaceae	SPL	Soil	83.16	54.10	5388017	5009	927.60	50	2	28	89	49	29.31	6.38	Aerobic	Scarcity
GCA_002483395	c__Blastocatellia	o__Pyrinomonadales	f__Pyrinomonadaceae	SPL	Engineered	87.31	51.35	4454120	4795	819.57	44	1	11	48	53	23.6	6.58	Aerobic	Scarcity
GCA_002483445	c__Blastocatellia	o__UBA7656	f__UBA7656	SPL	Engineered	89.20	54.55	8149540	6906	1053.74	93	1	36	35	81	30.68	5.85	Aerobic	Ruderal
GCA_002898775	c__Blastocatellia	o__HR10	f__HR10	SPL	Engineered	91.94	63.70	2602175	2410	994.17	25	0	4	22	26	66.51	5.82	Anaerobic	Scarcity
GCA_002923315	c__Blastocatellia	o__HR10	f__HR10	SPL	Engineered	91.78	63.75	3131958	2786	1033.18	32	1	8	24	31	67.83	5.91	Anaerobic	Scarcity
GCA_003135515	c__Blastocatellia	o__Pyrinomonadales	f__Pyrinomonadaceae	SPL	Soil	87.15	55.87	5155147	4492	1001.59	43	3	26	35	46	28.52	6.12	Aerobic	Scarcity
GCA_003168335	c__Blastocatellia	o__Pyrinomonadales	f__Pyrinomonadaceae	SPL	Soil	87.09	55.87	5160709	4471	1006.41	43	2	26	35	46	28.6	6.12	Aerobic	Scarcity
GCA_003175865	c__Blastocatellia	o__Pyrinomonadales	f__Pyrinomonadaceae	SPL	Soil	89.34	50.85	3566438	3126	1016.64	35	1	16	64	35	27.43	6.55	Aerobic	Competitor
GCA_003222245	c__Blastocatellia	o__UBA7656	f__UBA7656	SPL	Soil	87.74	55.49	6999717	5533	1109.67	61	3	160	31	59	26.91	6.16	Aerobic	Scarcity
GCA_003222835	c__Blastocatellia	o__Pyrinomonadales	f__Pyrinomonadaceae	SPL	Soil	86.72	55.07	5483195	5886	814.57	62	2	33	64	66	28.02	6.49	Aerobic	Competitor
GCA_003222845	c__Blastocatellia	o__Pyrinomonadales	f__Pyrinomonadaceae	SPL	Soil	87.63	54.64	6049692	5913	902.09	36	0	36	93	59	27.43	6.26	Aerobic	Scarcity
GCA_003222855	c__Blastocatellia	o__Pyrinomonadales	f__Pyrinomonadaceae	SPL	Soil	87.01	54.81	4114900	4391	821.91	32	0	20	50	57	28.84	6.27	Aerobic	Scarcity
GCA_003222895	c__Blastocatellia	o__Pyrinomonadales	f__Pyrinomonadaceae	SPL	Soil	89.54	54.36	4774423	4383	977.68	47	1	52	47	47	29.32	6.14	Aerobic	Scarcity
GCA_003222935	c__Blastocatellia	o__Pyrinomonadales	f__Pyrinomonadaceae	SPL	Soil	84.32	51.54	5193765	5159	871.34	46	1	16	107	67	25.04	6.15	Aerobic	Scarcity
GCA_003222945	c__Blastocatellia	o__Pyrinomonadales	f__Pyrinomonadaceae	SPL	Soil	88.24	53.46	4744547	4486	933.66	40	2	12	66	62	34.34	6.15	Aerobic	Competitor
GCA_003222975	c__Blastocatellia	o__Pyrinomonadales	f__Pyrinomonadaceae	SPL	Soil	87.50	65.14	4928114	4127	1043.34	45	2	25	86	59	36.54	6.60	Aerobic	Competitor
GCA_003223025	c__Blastocatellia	o__Pyrinomonadales	f__Pyrinomonadaceae	SPL	Soil	88.92	53.40	4340924	3756	1030.15	39	3	16	52	46	23.94	6.27	Aerobic	Scarcity
GCA_003223935	c__Blastocatellia	o__Pyrinomonadales	f__Pyrinomonadaceae	SPL	Soil	88.50	59.56	5269222	4518	1032.31	47	4	57	56	56	36.54	6.21	Aerobic	Competitor
GCA_003223975	c__Blastocatellia	o__Pyrinomonadales	f__Pyrinomonadaceae	SPL	Soil	91.99	52.36	3658515	3663	921.04	28	7	12	52	49	25.57	6.31	Aerobic	Scarcity
GCA_003385575	c__Blastocatellia	o__Pyrinomonadales	f__Pyrinomonadaceae	SPL	Engineered	87.69	53.51	4128806	3514	1031.18	31	0	30	25	40	27.44	6.78	Aerobic	Scarcity
GCA_003385625	c__Blastocatellia	o__Pyrinomonadales	f__Pyrinomonadaceae	SPL	Engineered	87.63	53.51	4128552	3509	1031.94	31	0	30	25	40	27.49	6.78	Aerobic	Scarcity
GCA_003385635	c__Blastocatellia	o__Pyrinomonadales	f__Pyrinomonadaceae	SPL	Engineered	87.65	53.51	4128386	3511	1031.57	31	0	30	25	40	27.48	6.78	Aerobic	Scarcity
GCA_003389245	c__Blastocatellia	o__Pyrinomonadales	f__Pyrinomonadaceae	SPL	Engineered	87.63	53.51	4127862	3511	1031.20	31	0	35	25	40	27.48	6.78	Aerobic	Scarcity
GCA_003445755	c__Blastocatellia	o__Pyrinomonadales	f__Pyrinomonadaceae	SPL	Soil	80.27	54.44	5079152	4972	899.71	47	2	24	76	45	31.88	6.41	Aerobic	Scarcity
GCA_003445815	c__Blastocatellia	o__Pyrinomonadales	f__Pyrinomonadaceae	SPL	Soil	83.18	54.27	4827676	4666	897.32	44	2	36	78	40	31.98	6.41	Aerobic	Scarcity
GCA_003487805	c__Blastocatellia	o__Pyrinomonadales	f__Pyrinomonadaceae	SPL	Soil	83.38	54.49	6016230	5432	980.45	51	3	22	55	59	33.04	6.21	Aerobic	Scarcity
GCA_003488005	c__Blastocatellia	o__Pyrinomonadales	f__Pyrinomonadaceae	SPL	Soil	83.36	54.11	4905516	4358	990.37	44	2	25	37	42	31.94	6.47	Aerobic	Scarcity
GCA_003501905	c__Blastocatellia	o__Pyrinomonadales	f__Pyrinomonadaceae	SPL	Engineered	85.10	53.52	2935450	3071	872.59	26	1	8	23	37	31.31	6.96	Aerobic	Scarcity
GCA_003539435	c__Blastocatellia	o__Pyrinomonadales	f__Pyrinomonadaceae	SPL	Soil	80.87	54.40	4161140	4226	862.83	37	0	28	53	40	35.48	6.24	Aerobic	Scarcity
GCA_003577445	c__Blastocatellia	o__Pyrinomonadales	f__Pyrinomonadaceae	SPL	Engineered	91.83	54.67	2802878	2662	967.27	27	2	8	26	38	27.8	7.08	Aerobic	Scarcity
GCA_003696865	c__Blastocatellia	o__HR10	f__J050	SPL	Freshwater	88.34	58.91	4440908	4075	960.62	44	0	28	36	58	52.93	5.79	Anaerobic	Competitor
GCA_003697135	c__Blastocatellia	o__Pyrinomonadales	f__Pyrinomonadaceae	SPL	Freshwater	91.72	42.89	2794560	2438	1052.16	35	4	12	22	34	47.53	6.76	Aerobic	Competitor
GCA_005790265	c__Blastocatellia	o__Pyrinomonadales	f__Pyrinomonadaceae	SPL	Freshwater	88.92	49.31	3830711	3725	912.43	40	1	12	50	49	29.96	6.42	Aerobic	Scarcity
GCA_013003215	c__Blastocatellia	o__Pyrinomonadales	f__Pyrinomonadaceae	SPL	Soil	89.40	49.48	4225043	3935	959.24	38	0	28	27	43	25.5	6.39	Aerobic	Scarcity
GCA_013003295	c__Blastocatellia	o__Pyrinomonadales	f__Pyrinomonadaceae	SPL	Soil	88.53	47.09	3851259	3801	895.96	40	0	20	33	39	29.05	6.68	Aerobic	Scarcity
GCA_013140875	c__Blastocatellia	o__Pyrinomonadales	f__Pyrinomonadaceae	SPL	Engineered	90.19	52.68	3494272	3703	850.00	26	0	12	58	45	25.18	6.93	Aerobic	Scarcity

GCA_013140935	c__Blastocatellia	o__RBC074	f__RBC074	SPL	Engineered	90.28	55.43	6089498	6078	904.11	72	1	20	63	67	29.31	6.21	Aerobic	Scarcity
GCA_013298335	c__Blastocatellia	o__Chloracidobacteriales	f__Chloracidobacteriaceae	SPL	Freshwater	87.79	60.70	5114314	4973	900.79	58	2	28	28	72	25.49	6.64	Aerobic	Scarcity
GCA_013695195	c__Blastocatellia	o__Pyrinomonadales	f__Pyrinomonadaceae	SPL	Soil	88.44	48.96	2831976	3007	832.85	26	1	12	24	37	26.28	6.62	Aerobic	Scarcity
GCA_013695245	c__Blastocatellia	o__Pyrinomonadales	f__Pyrinomonadaceae	SPL	Soil	89.48	55.02	3202503	3195	893.53	36	0	8	30	52	39.16	6.87	Aerobic	Scarcity
GCA_013695495	c__Blastocatellia	o__Pyrinomonadales	f__Pyrinomonadaceae	SPL	Soil	91.54	54.82	2483437	2641	858.97	25	0	12	17	34	26.19	6.95	Aerobic	Scarcity
GCA_013696385	c__Blastocatellia	o__Pyrinomonadales	f__Pyrinomonadaceae	SPL	Soil	88.83	43.74	2258757	2528	792.06	22	0	8	17	34	34.58	6.38	Aerobic	Scarcity
GCA_013696465	c__Blastocatellia	o__Pyrinomonadales	f__Pyrinomonadaceae	SPL	Soil	88.67	56.57	3286123	3171	917.69	29	0	12	26	41	37.36	6.87	Aerobic	Scarcity
GCA_013697915	c__Blastocatellia	o__Pyrinomonadales	f__Pyrinomonadaceae	SPL	Soil	86.40	54.07	4515099	4240	919.31	38	9	20	34	51	29.81	6.21	Aerobic	Scarcity
GCA_013698055	c__Blastocatellia	o__Pyrinomonadales	f__Pyrinomonadaceae	SPL	Soil	88.07	54.95	3815168	4139	810.84	35	2	8	25	54	32.09	6.15	Aerobic	Competitor
GCA_013811945	c__Blastocatellia	o__Pyrinomonadales	f__Pyrinomonadaceae	SPL	Soil	84.77	54.58	3978449	3900	864.70	29	6	36	22	51	29.78	6.21	Aerobic	Competitor
GCA_013812565	c__Blastocatellia	o__Pyrinomonadales	f__Pyrinomonadaceae	SPL	Soil	86.78	55.21	5337958	5484	842.15	55	1	20	68	50	33.15	5.92	Aerobic	Scarcity
GCA_013812905	c__Blastocatellia	o__Pyrinomonadales	f__Pyrinomonadaceae	SPL	Soil	85.44	54.14	3794124	3603	898.47	27	1	12	38	47	31.01	6.15	Aerobic	Scarcity
GCA_013813755	c__Blastocatellia	o__Pyrinomonadales	f__Pyrinomonadaceae	SPL	Soil	89.21	44.86	1996848	2019	882.13	21	0	0	21	35	30.7	6.92	Aerobic	Scarcity
GCA_013813825	c__Blastocatellia	o__Pyrinomonadales	f__Pyrinomonadaceae	SPL	Soil	86.04	59.62	3955857	4085	829.21	38	0	8	38	63	34.14	6.46	Aerobic	Scarcity
GCA_013816505	c__Blastocatellia	o__Pyrinomonadales	f__Pyrinomonadaceae	SPL	Soil	85.29	58.44	6380153	6212	874.37	52	3	40	75	73	32.64	5.80	Aerobic	Ruderal
GCA_013816965	c__Blastocatellia	o__Pyrinomonadales	f__Pyrinomonadaceae	SPL	Soil	88.58	44.30	3719358	3712	888.27	40	1	12	33	47	33.24	6.39	Aerobic	Scarcity
GCA_013817595	c__Blastocatellia	o__Pyrinomonadales	f__Pyrinomonadaceae	SPL	Soil	84.13	54.70	2918649	2961	826.81	27	2	8	24	45	32.38	6.73	Aerobic	Scarcity
GCA_013820745	c__Blastocatellia	o__Pyrinomonadales	f__Pyrinomonadaceae	SPL	Soil	87.79	45.49	4637274	4606	885.24	35	2	12	39	56	31.11	6.23	Aerobic	Scarcity
GCA_014379075	c__Blastocatellia	o__Pyrinomonadales	f__Pyrinomonadaceae	SPL	Soil	90.05	50.42	3379958	3479	873.79	35	0	8	36	53	24.8	6.64	Aerobic	Scarcity
GCA_014379795	c__Blastocatellia	o__Pyrinomonadales	f__Pyrinomonadaceae	SPL	Soil	88.24	55.51	3744036	3791	869.40	35	0	36	17	53	33.52	5.88	Aerobic	Scarcity
GCA_014380365	c__Blastocatellia	o__Pyrinomonadales	f__Pyrinomonadaceae	SPL	Soil	87.76	55.45	4977406	4192	1043.17	36	1	26	31	38	27.55	6.21	Aerobic	Scarcity
GCA_014534425	c__Blastocatellia	o__Pyrinomonadales	f__Pyrinomonadaceae	SPL	Soil	89.32	56.68	5425231	4894	988.54	51	1	42	43	58	36.36	6.50	Aerobic	Ruderal
GCA_015075585	c__Blastocatellia	o__Pyrinomonadales	f__Pyrinomonadaceae	SPL	Engineered	92.56	53.82	2970818	2690	1023.76	25	1	8	26	37	29.08	6.54	Aerobic	Competitor
GCA_016179065	c__Blastocatellia	o__RBC074	f__RBC074	SPL	Freshwater	88.06	58.23	9076198	7125	1120.97	70	1	28	108	77	28.28	5.80	Aerobic	Scarcity
GCA_016196035	c__Blastocatellia	o__RBC074	f__RBC074	SPL	Freshwater	87.97	58.28	9593663	7300	1156.75	72	4	36	111	76	28.18	5.81	Aerobic	Competitor
GCA_016201275	c__Blastocatellia	o__UBA7656	f__UBA7656	SPL	Freshwater	84.04	52.97	6840417	5541	1037.58	63	3	40	54	50	31.39	5.96	Aerobic	Scarcity
GCA_016208495	c__Blastocatellia	o__Chloracidobacteriales	f__Chloracidobacteriaceae	SPL	Freshwater	85.32	51.08	8375582	6047	1181.63	111	2	74	35	56	24.65	6.60	Aerobic	Ruderal
GCA_016210985	c__Blastocatellia	o__HR10	f__JACQTD01	SPL	Freshwater	88.61	59.93	3849857	3539	962.53	32	2	16	12	34	32.25	6.22	Anaerobic	Scarcity
GCA_016213785	c__Blastocatellia	o__Chloracidobacteriales	f__JADJPF01	SPL	Freshwater	88.65	34.30	6395054	4988	1136.27	86	0	56	29	44	27.66	6.50	Aerobic	Competitor
GCA_016700075	c__Blastocatellia	o__Pyrinomonadales	f__Pyrinomonadaceae	SPL	Engineered	92.55	53.85	3393545	3080	1021.35	34	3	12	19	29	26.11	6.90	Aerobic	Scarcity
GCA_016700175	c__Blastocatellia	o__Pyrinomonadales	f__Pyrinomonadaceae	SPL	Engineered	85.80	57.32	4490879	3751	1028.11	31	2	45	22	44	28.02	6.46	Aerobic	Scarcity
GCA_016700195	c__Blastocatellia	o__RBC074	f__RBC074	SPL	Engineered	89.44	58.59	6333691	4928	1150.92	57	2	36	73	60	33.34	5.76	Aerobic	Scarcity
GCA_016702945	c__Blastocatellia	o__RBC074	f__RBC074	SPL	Engineered	87.32	53.21	5688097	5009	993.78	37	4	28	67	82	28.36	5.77	Aerobic	Scarcity
GCA_016703135	c__Blastocatellia	o__Pyrinomonadales	f__Pyrinomonadaceae	SPL	Engineered	88.99	52.48	3961352	3877	911.54	36	0	12	30	39	23.24	6.50	Aerobic	Scarcity
GCA_016703285	c__Blastocatellia	o__Pyrinomonadales	f__Pyrinomonadaceae	SPL	Engineered	88.72	53.11	4274637	4342	876.54	37	1	8	39	52	23.45	6.47	Aerobic	Scarcity
GCA_016703965	c__Blastocatellia	o__Pyrinomonadales	f__Pyrinomonadaceae	SPL	Engineered	88.96	52.16	5079012	4800	942.61	47	6	12	83	55	23.03	6.37	Aerobic	Scarcity
GCA_016704725	c__Blastocatellia	o__UBA7656	f__JADIYM01	SPL	Engineered	86.51	63.78	5782885	5000	1002.59	44	3	28	30	52	26.42	5.85	Anaerobic	Scarcity
GCA_016705245	c__Blastocatellia	o__UBA7656	f__JADIYM01	SPL	Engineered	87.12	63.90	5570566	4800	1013.91	44	1	24	32	49	26.73	5.77	Aerobic	Scarcity
GCA_016706035	c__Blastocatellia	o__Pyrinomonadales	f__Pyrinomonadaceae	SPL	Engineered	90.18	52.11	3900387	3523	999.79	34	1	14	25	46	23.23	6.48	Aerobic	Scarcity
GCA_016706275	c__Blastocatellia	o__Pyrinomonadales	f__Pyrinomonadaceae	SPL	Engineered	91.78	51.88	3369823	3140	986.23	31	0	12	23	34	22.84	6.50	Aerobic	Scarcity
GCA_016709645	c__Blastocatellia	o__Pyrinomonadales	f__Pyrinomonadaceae	SPL	Engineered	83.46	55.23	5938577	5725	867.95	40	3	16	50	54	24.74	6.78	Aerobic	Scarcity
GCA_016710075	c__Blastocatellia	o__RBC074	f__RBC074	SPL	Engineered	85.73	53.30	5949241	5507	928.81	43	1	20	70	91	28.48	5.79	Aerobic	Scarcity
GCA_016711345	c__Blastocatellia	o__Pyrinomonadales	f__Pyrinomonadaceae	SPL	Engineered	90.66	49.87	4595114	4235	985.25	38	11	20	23	29	22.3	6.50	Aerobic	Scarcity
GCA_016711965	c__Blastocatellia	o__RBC074	f__RBC074	SPL	Engineered	86.46	53.36	5710942	5167	957.47	35	1	24	68	91	28.67	5.82	Aerobic	Scarcity
GCA_016712045	c__Blastocatellia	o__Pyrinomonadales	f__Pyrinomonadaceae	SPL	Engineered	88.84	52.01	3716898	4204	788.86	34	2	12	33	53	22.84	6.50	Aerobic	Scarcity
GCA_016712785	c__Blastocatellia	o__Pyrinomonadales	f__Pyrinomonadaceae	SPL	Engineered	84.23	55.37	5527228	5331	875.79	38	4	16	50	56	24.8	6.78	Aerobic	Scarcity
GCA_016713405	c__Blastocatellia	o__Chloracidobacteriales	f__JADJPF01	SPL	Engineered	87.62	34.50	6273563	5678	969.54	69	1	68	27	54	25.92	5.56	Aerobic	Scarcity
GCA_016713675	c__Blastocatellia	o__Pyrinomonadales	f__Pyrinomonadaceae	SPL	Engineered	89.61	52.32	4128691	3891	952.76	35	2	12	32	44	23.19	6.50	Aerobic	Scarcity
GCA_016714265	c__Blastocatellia	o__Pyrinomonadales	f__Pyrinomonadaceae	SPL	Engineered	89.04	52.09	4087816	3899	934.36	35	5	14	26	53	23.37	6.48	Aerobic	Scarcity
GCA_016715115	c__Blastocatellia	o__Pyrinomonadales	f__Pyrinomonadaceae	SPL	Engineered	86.54	55.23	5677984	5014	981.89	36	4	24	57	48	24.52	6.78	Anaerobic	Scarcity
GCA_016715795	c__Blastocatellia	o__Pyrinomonadales	f__Pyrinomonadaceae	SPL	Engineered	91.92	55.67	3307481	3125	974.92	31	1	12	18	35	27.39	6.63	Aerobic	Scarcity
GCA_016715865	c__Blastocatellia	o__RBC074	f__RBC074	SPL	Engineered	88.18	54.76	5835500	4846	1063.11	52	6	40	76	61	29.86	5.97	Aerobic	Scarcity
GCA_016716305	c__Blastocatellia	o__Pyrinomonadales	f__Pyrinomonadaceae	SPL	Engineered	91.41	53.09	3793765	3453	1005.93	31	1	12	27	43	23.59	6.81	Aerobic	Scarcity
GCA_016716365	c__Blastocatellia	o__Pyrinomonadales	f__Pyrinomonadaceae	SPL	Engineered	88.62	56.43	4391464	4124	945.09	31	2	12	27	60	26.53	6.50	Aerobic	Scarcity
GCA_016716435	c__Blastocatellia	o__Pyrinomonadales	f__Pyrinomonadaceae	SPL	Engineered	89.82	55.54	3805121	3714	921.67	31	1	16	26	36	24.44	6.90	Aerobic	Scarcity
GCA_016717465	c__Blastocatellia	o__RBC074	f__RBC074	SPL	Engineered	87.84	53.33	5355134	4689	1005.80	32	0	16	64	87	28.78	5.71	Aerobic	Scarcity
GCA_016718135	c__Blastocatellia	o__Pyrinomonadales	f__Pyrinomonadaceae	SPL	Engineered	89.54	53.14	3913344	3731	941.27	34	2	12	37	55	23.27	6.38	Aerobic	Scarcity
GCA_016719125	c__Blastocatellia	o__Pyrinomonadales	f__Pyrinomonadaceae	SPL	Engineered	89.78	52.12	3886761	3587	974.18	33	0	14	24	47	23.22	6.48	Aerobic	Scarcity
GCA_016720705	c__Blastocatellia	o__Pyrinomonadales	f__Pyrinomonadaceae	SPL	Engineered	89.69	52.04	4036661	3623	1000.44	33	2	14	26	46	23.22	6.48	Aerobic	Scarcity
GCA_016721095	c__Blastocatellia	o__Pyrinomonadales	f__Pyrinomonadaceae	SPL	Engineered	86.89	52.12	3781336	4435	744.58	33	2	14	27	62	23.4	6.75	Aerobic	Scarcity
GCA_016789085	c__Blastocatellia	o__Chloracidobacteriales	f__JADJPF01	SPL	Engineered	89.90	34.50	5833372	5254	997.70	72	0	78	27	53	26.4	6.24	Aerobic	Competitor
GCA_016789145	c__Blastocatellia	o__Pyrinomonadales	f__Pyrinomonadaceae	SPL	Engineered	90.75	51.82	3614633	3477	942.89	28	0	12	25	44	25.83	6.50	Aerobic	Scarcity
GCA_016789265	c__Blastocatellia	o__Pyrinomonadales	f__Pyrinomonadaceae	SPL	Engineered	91.88	52.01	3883780	3590	994.54	37	1	12	42	48	25.17	6.55	Aerobic	Scarcity
GCA_016789285	c__Blastocatellia	o__Pyrinomonadales	f__Pyrinomonadaceae	SPL	Engineered	91.93	53.36	3307635	3140	968.61	30	0	16	24	40	23.53	6.56	Aerobic	Scarcity
GCA_016789295	c__Blastocatellia	o__Chloracidobacteriales	f__JADJPF01	SPL	Engineered	89.34	41.02	4588508	4390	931.62	74	1	96	25	60	26.44	6.13	Aerobic	Competitor
GCA_016789605	c__Blastocatellia	o__RBC074	f__RBC074	SPL	Engineered	90.68	55.93	8464934	6668	1150.61	82	1	46	107	86	27.37	5.74	Aerobic	Ruderal
GCA_016789645	c__Blastocatellia	o__RBC074	f__RBC074	SPL	Engineered	90.94	53.85	6273080	6015	946.49	66	2	28	55	79	26.74	6.84	Aerobic	Scarcity
GCA_016789725	c__Blastocatellia	o__RBC074	f__RBC074	SPL	Engineered	91.19	52.78	7771044	6716	1055.52	83	1	36	102	89	28.09	6.66	Aerobic	Competitor
GCA_017303955	c__Blastocatellia	o__Chloracidobacteriales	f__JADJPF01	SPL	Engineered	90.49	34.51	6148772	4818	1156.02	63	0	58	29	45	26.62	6.10	Aerobic	Competitor
GCA_017883315	c__Blastocatellia	o__UBA7656	f__UBA7656	SPL	Soil	88.74	56.31	4628963	4506	912.53	46	1	32	41	64	29.37	5.51	Anaerobic	Scarcity
GCA_017983495	c__Blastocatellia	o__Pyrinomonadales	f__Pyrinomonadaceae	SPL	Engineered	90.82	53.27	4016138	3562	1025.46	36	1	12	35	39	22.67	6.71	Aerobic	Scarcity
GCA_017985955	c__Blastocatellia	o__Pyrinomonadales	f__Pyrinomonadaceae	SPL	Engineered	91.07	53.30	3492091	3228	985.86	34	1	4	35	34	22.85	6.72	Aerobic	Scarcity
GCA_017991275	c__Blastocatellia	o__RBC074	f__RBC074	SPL	Engineered	90.35	52.31	5688912	5137	999.13	51	2	36	72	68	25.47	5.93	Aerobic	Scarcity
GCA_017997075	c__Blastocatellia	o__Pyrinomonadales	f__Pyrinomonadaceae	SPL	Engineered	91.23	52.49	3415414	3271	956.43	32	1	12	33	42	23.99	6.50	Aerobic	Scarcity
GCA_017997755	c__Blastocatellia	o__Pyrinomonadales	f__Pyrinomonadaceae	SPL	Engineered	91.93	53.12	3500360	3366	957.28	39	2	12	39	48	23.47	6.38	Aerobic	Scarcity
GCA_018060305	c__Blastocatellia	o__Pyrinomonadales	f__Pyrinomonadaceae	SPL	Engineered	90.58	55.74	2558652	2704	864.79	24	0	4	17	28	27.33	6.66	Aerobic	Scarcity
GCA_018063205	c__Blastocatellia	o__Pyrinomonadales	f__Pyrinomonadaceae	SPL	Engineered	91.10	51.94	3412064	3057	1017.54	28	0	14	22	46	23.42	6.48	Aerobic	Scarcity
GCA_018268995	c__Blastocatellia	o__RBC074	f__RBC074	SPL	Engineered	90.27	51.48	8408168	6862	1107.47	89	3	36	115	84	27.5	6.58	Aerobic	Ruderal
GCA_018269005	c__Blastocatellia	o__Pyrinomonadales	f__Pyrinomonadaceae	SPL	Engineered	90.79	54.46	3553110	3293	980.14	35	2	19	32	39	27.48	6.60	Aerobic	Scarcity
GCA_018269055	c__Blastocatellia	o__RBC074	f__RBC074	SPL	Engineered	90.73	53.47	7909138	6242	1151.82	79	3	52	86	73	26.59	5.78	Aerobic	Scarcity
GCA_018269075	c__Blastocatellia	o__Pyrinomonadales	f__Pyrinomonadaceae	SPL	Engineered	85.23	58.60	7405842	5923	1066.00	57	3	20	112	65	27.73	6.45	Aerobic	Ruderal
GCA_018304665	c__Blastocatellia	o__Chloracidobacteriales	f__Chloracidobacteriaceae	SPL	Freshwater	90.43	61.31	3756354	3243	1049.99	34	0	28	19	53	42.6	6.82	Aerobic	Scarcity
GCA_018304685	c__Blastocatellia	o__Chloracidobacteriales	f__Chloracidobacteriaceae	SPL	Freshwater	91.06	61.48	3635588	3019	1098.20	40	0	32	18	53	43.46	6.82	Aerobic	Scarcity
GCA_018304705	c__Blastocatellia	o__Chloracidobacteriales	f__Chloracidobacteriaceae	SPL	Freshwater	90.74	62.15	3766674	3155	1085.50	32	1	32	18	51	42.18	6.53	Aerobic	Scarcity
GCA_018304725	c__Blastocatellia	o__Chloracidobacteriales	f__Chloracidobacteriaceae	SPL	Freshwater	90.55	62.13	3757470	3200	1065.45	32	2	32	18	51	42.07	6.77	Aerobic	Scarcity
GCA_018304745	c__Blastocatellia	o__Chloracidobacteriales	f__Chloracidobacteriaceae	SPL	Freshwater	90.54	62.14	3769663	3228	1059.66	34	1	32	18	54	42.25	6.77	Aerobic	Scarcity
GCA_018304765	c__Blastocatellia	o__Chloracidobacteriales	f__Chloracidobacteriaceae	SPL	Freshwater	90.87	62.15	3715176	3036	1113.67	32	1	32	18	50	42.47	6.77	Aerobic	Scarcity

GCA_018304785	c__Blastocatellia	o__Chloracidobacteriales	f__Chloracidobacteriaceae	SPL	Freshwater	90.66	62.18	3777155	3127	1096.96	31	1	32	18	52	42.02	6.77	Aerobic	Scarcity
GCA_018304805	c__Blastocatellia	o__Chloracidobacteriales	f__Chloracidobacteriaceae	SPL	Freshwater	91.23	62.68	3662232	2967	1127.80	31	0	32	18	50	41.69	6.82	Aerobic	Scarcity
GCA_018304825	c__Blastocatellia	o__Chloracidobacteriales	f__Chloracidobacteriaceae	SPL	Freshwater	91.18	59.87	3659143	2986	1119.03	33	1	37	19	52	38.63	6.77	Aerobic	Scarcity
GCA_019186825	c__Blastocatellia	o__Pyrinomonadales	f__Pyrinomonadaceae	SPL	Engineered	91.53	54.77	3204297	2937	1000.34	30	3	12	28	38	27.94	7.00	Aerobic	Scarcity
GCA_019239425	c__Blastocatellia	o__Pyrinomonadales	f__Pyrinomonadaceae	SPL	Host	89.61	66.80	7446924	5950	1122.26	73	2	74	136	74	36.25	6.50	Aerobic	Competitor
GCA_019242475	c__Blastocatellia	o__Pyrinomonadales	f__Pyrinomonadaceae	SPL	Host	89.84	67.12	6142585	4951	1115.07	56	1	60	125	67	35.79	6.63	Aerobic	Scarcity
GCA_019247455	c__Blastocatellia	o__Pyrinomonadales	f__Pyrinomonadaceae	SPL	Terrestrial_n	92.60	54.93	3279301	3315	914.90	28	3	12	29	36	27.21	6.47	Aerobic	Scarcity
GCA_019247865	c__Blastocatellia	o__Pyrinomonadales	f__Pyrinomonadaceae	SPL	Host	89.56	61.10	3983875	3902	913.49	34	0	20	25	52	33.98	6.04	Aerobic	Scarcity
GCA_019639115	c__Blastocatellia	o__Pyrinomonadales	f__Pyrinomonadaceae	SPL	Engineered	93.09	55.71	3091289	2792	1031.82	25	2	12	30	35	27.75	6.70	Aerobic	Scarcity
GCA_019639135	c__Blastocatellia	o__Pyrinomonadales	f__Pyrinomonadaceae	SPL	Engineered	90.49	55.52	4088851	3809	972.10	37	3	12	28	31	26.25	6.93	Aerobic	Scarcity
GCA_019639155	c__Blastocatellia	o__Pyrinomonadales	f__Pyrinomonadaceae	SPL	Engineered	92.31	54.00	3219030	2906	1024.06	31	0	12	14	27	26.63	7.12	Aerobic	Scarcity
GCA_019639175	c__Blastocatellia	o__Pyrinomonadales	f__Pyrinomonadaceae	SPL	Engineered	90.16	51.45	3800428	3658	937.75	37	3	8	34	36	27.07	6.25	Aerobic	Scarcity
GCA_019639195	c__Blastocatellia	o__Pyrinomonadales	f__Pyrinomonadaceae	SPL	Engineered	92.15	53.83	3222933	2929	1015.13	31	3	4	29	37	27.44	6.37	Aerobic	Scarcity
GCA_019639215	c__Blastocatellia	o__Pyrinomonadales	f__Pyrinomonadaceae	SPL	Engineered	90.80	48.59	2910571	2777	952.59	24	1	4	28	36	26.57	6.51	Aerobic	Scarcity
GCA_019639235	c__Blastocatellia	o__Pyrinomonadales	f__Pyrinomonadaceae	SPL	Engineered	91.00	56.26	3842181	3609	969.55	35	3	12	26	35	25.48	6.90	Aerobic	Scarcity
GCA_019639255	c__Blastocatellia	o__RBC074	f__RBC074	SPL	Engineered	89.32	58.04	5831261	4528	1149.61	50	1	24	58	54	35.16	6.24	Aerobic	Scarcity
GCA_019686075	c__Blastocatellia	o__Pyrinomonadales	f__Pyrinomonadaceae	SPL	Terrestrial_n	89.33	60.04	3229178	2879	998.74	32	1	8	77	42	59.47	6.16	Aerobic	Competitor
GCA_019694345	c__Blastocatellia	o__Pyrinomonadales	f__Pyrinomonadaceae	SPL	Engineered	92.17	54.79	3511489	3186	1015.93	33	1	12	31	44	25.2	6.76	Aerobic	Scarcity
GCA_019694355	c__Blastocatellia	o__Pyrinomonadales	f__Pyrinomonadaceae	SPL	Engineered	91.05	53.73	3406639	3136	990.24	31	1	19	32	38	27.32	6.60	Aerobic	Scarcity
GCA_019694395	c__Blastocatellia	o__Pyrinomonadales	f__Pyrinomonadaceae	SPL	Engineered	90.48	40.27	6370948	5850	985.82	73	5	44	117	73	29.72	6.38	Aerobic	Competitor
GCA_019695185	c__Blastocatellia	o__Chloracidobacteriales	f__Chloracidobacteriaceae	SPL	Engineered	88.55	53.97	8329599	6211	1187.29	113	5	90	31	66	24.43	6.24	Aerobic	Competitor
GCA_020199775	c__Blastocatellia	o__Pyrinomonadales	f__Pyrinomonadaceae	SPL	Soil	86.86	61.24	5908740	4786	1072.23	44	5	155	52	47	35.02	5.87	Aerobic	Competitor
GCA_020199835	c__Blastocatellia	o__Pyrinomonadales	f__Pyrinomonadaceae	SPL	Soil	91.55	55.83	4871352	4235	1054.78	44	3	88	52	45	30.68	6.26	Aerobic	Competitor
GCA_020199975	c__Blastocatellia	o__Pyrinomonadales	f__Pyrinomonadaceae	SPL	Soil	85.21	54.70	2927977	3116	802.28	30	0	12	38	41	33.05	5.87	Aerobic	Scarcity
GCA_020199995	c__Blastocatellia	o__Pyrinomonadales	f__Pyrinomonadaceae	SPL	Soil	85.81	63.97	5317045	4795	958.28	49	1	93	41	57	33.8	6.24	Aerobic	Scarcity
GCA_020200095	c__Blastocatellia	o__Pyrinomonadales	f__Pyrinomonadaceae	SPL	Soil	87.24	44.65	4320852	4412	853.21	45	4	8	31	45	30.22	6.43	Aerobic	Scarcity
GCA_020200135	c__Blastocatellia	o__Pyrinomonadales	f__Pyrinomonadaceae	SPL	Soil	88.42	68.48	5268388	4608	1012.13	42	1	32	76	52	36.2	6.53	Aerobic	Scarcity
GCA_020200155	c__Blastocatellia	o__Pyrinomonadales	f__Pyrinomonadaceae	SPL	Soil	87.37	61.13	4251322	3874	960.46	36	2	33	37	45	36.15	6.93	Aerobic	Scarcity
GCA_020200175	c__Blastocatellia	o__Pyrinomonadales	f__Pyrinomonadaceae	SPL	Soil	87.46	44.20	3629057	3894	814.50	36	1	12	28	48	30.31	6.47	Aerobic	Scarcity
GCA_020200215	c__Blastocatellia	o__Pyrinomonadales	f__Pyrinomonadaceae	SPL	Soil	86.65	66.47	3643475	3505	904.26	25	0	12	36	51	35.53	6.21	Aerobic	Scarcity
GCA_020200235	c__Blastocatellia	o__Pyrinomonadales	f__Pyrinomonadaceae	SPL	Soil	86.69	66.19	4078885	3626	977.93	27	1	12	36	47	34.96	6.15	Aerobic	Scarcity
GCA_020201365	c__Blastocatellia	o__Pyrinomonadales	f__Pyrinomonadaceae	SPL	Soil	88.53	66.97	4117239	3795	959.46	28	0	16	46	62	36.43	6.47	Aerobic	Scarcity
GCA_020846885	c__Blastocatellia	o__Pyrinomonadales	f__Pyrinomonadaceae	SPL	Engineered	89.67	53.94	3474071	3634	856.34	35	2	8	42	48	26.24	6.54	Aerobic	Scarcity
GCA_020848535	c__Blastocatellia	o__Pyrinomonadales	f__Pyrinomonadaceae	SPL	Engineered	92.78	54.38	2773288	2543	1012.47	24	0	12	22	36	29.56	6.54	Aerobic	Scarcity
GCA_020849315	c__Blastocatellia	o__UBA7656	f__JADIYM01	SPL	Engineered	89.63	63.37	4179968	4013	933.97	43	3	8	19	44	27.79	6.20	Aerobic	Scarcity
GCA_020853255	c__Blastocatellia	o__Pyrinomonadales	f__Pyrinomonadaceae	SPL	Engineered	89.72	53.46	3733934	3924	852.01	33	4	16	43	46	25.78	5.88	Aerobic	Scarcity
GCA_021155085	c__Blastocatellia	o__RBC074	f__RBC074	SPL	Engineered	90.67	56.44	9436427	7091	1207.25	89	3	48	105	85	25.66	5.82	Aerobic	Ruderal
GCA_021155145	c__Blastocatellia	o__Pyrinomonadales	f__Pyrinomonadaceae	SPL	Engineered	91.21	55.57	2678735	2925	833.61	25	0	20	23	36	27.57	6.75	Aerobic	Scarcity
GCA_021228405	c__Blastocatellia	o__Pyrinomonadales	f__Pyrinomonadaceae	SPL	Freshwater	88.56	46.36	5719470	4922	1030.03	71	2	40	80	45	29.21	6.89	Aerobic	Scarcity
GCA_021323895	c__Blastocatellia	o__Pyrinomonadales	f__Pyrinomonadaceae	SPL	Soil	91.28	55.60	4396503	4076	984.88	43	1	12	54	64	29.78	6.49	Aerobic	Scarcity
GCA_021405015	c__Blastocatellia	o__Pyrinomonadales	f__Pyrinomonadaceae	SPL	Engineered	91.78	54.65	3009623	2994	923.83	28	2	8	25	45	28.26	7.00	Aerobic	Scarcity
GCA_021463085	c__Blastocatellia	o__Pyrinomonadales	f__Pyrinomonadaceae	SPL	Engineered	92.29	54.28	3162093	3022	966.61	24	2	20	24	37	29.32	6.54	Aerobic	Scarcity
GCA_021463145	c__Blastocatellia	o__UBA7656	f__JADIYM01	SPL	Engineered	88.71	63.41	5281103	4380	1068.65	47	5	16	22	44	27.28	6.01	Aerobic	Competitor
GCA_021463165	c__Blastocatellia	o__Pyrinomonadales	f__Pyrinomonadaceae	SPL	Engineered	91.83	54.19	3661027	3381	995.24	30	1	12	50	43	27.29	6.26	Aerobic	Scarcity
GCA_022072205	c__Blastocatellia	o__RBC074	f__RBC074	SPL	Engineered	87.91	57.80	6468668	5353	1093.70	60	2	36	81	70	33.2	5.44	Aerobic	Scarcity
GCA_022614735	c__Blastocatellia	o__RBC074	f__RBC074	SPL	Terrestrial_n	88.10	52.22	4376610	3827	1005.35	48	5	20	43	42	37.06	6.03	Aerobic	Scarcity
GCA_022615215	c__Blastocatellia	o__UBA7656	f__UBA7656	SPL	Terrestrial_n	86.62	55.30	7589036	6631	986.26	93	4	60	54	69	33.6	6.24	Aerobic	Competitor
GCA_022841755	c__Blastocatellia	o__Pyrinomonadales	f__Pyrinomonadaceae	SPL	Engineered	91.12	52.94	4708242	4272	1005.62	35	6	16	52	45	23.57	6.49	Aerobic	Scarcity
GCA_022841865	c__Blastocatellia	o__Pyrinomonadales	f__Pyrinomonadaceae	SPL	Engineered	90.87	55.23	3893264	3648	971.21	33	6	16	24	37	24.12	6.90	Aerobic	Scarcity
GCA_022843305	c__Blastocatellia	o__Pyrinomonadales	f__Pyrinomonadaceae	SPL	Engineered	88.73	47.15	5721532	5422	936.93	55	7	104	38	50	29.71	6.69	Aerobic	Scarcity
GCA_023265195	c__Blastocatellia	o__UBA7656	f__UBA7656	SPL	Freshwater	82.29	56.76	7546609	6531	966.40	69	0	208	40	49	28.15	5.97	Anaerobic	Scarcity
GCA_023265235	c__Blastocatellia	o__Chloracidobacteriales	f__JADJPF01	SPL	Freshwater	88.51	36.17	5454763	4928	980.37	78	0	73	28	44	26.79	6.77	Aerobic	Ruderal
GCA_023389035	c__Blastocatellia	o__Pyrinomonadales	f__Pyrinomonadaceae	SPL	Host	90.90	54.52	3710723	3362	1004.57	24	0	16	24	36	25.1	6.66	Aerobic	Scarcity
GCA_023391155	c__Blastocatellia	o__Pyrinomonadales	f__Pyrinomonadaceae	SPL	Host	91.45	56.63	3123171	3087	924.79	27	0	8	15	43	28.04	6.63	Aerobic	Scarcity
GCA_023415725	c__Blastocatellia	o__Pyrinomonadales	f__Pyrinomonadaceae	SPL	Host	90.02	56.91	3807720	3626	943.60	34	0	20	36	52	28.56	6.95	Aerobic	Scarcity
GCA_023417295	c__Blastocatellia	o__Pyrinomonadales	f__Pyrinomonadaceae	SPL	Host	92.19	56.54	2096127	2268	849.10	25	0	4	12	31	29.5	6.90	Aerobic	Scarcity
GCA_023436495	c__Blastocatellia	o__Pyrinomonadales	f__Pyrinomonadaceae	SPL	Host	90.50	54.69	3476376	3260	965.89	33	1	16	25	41	27.3	6.95	Aerobic	Scarcity
GCA_023717565	c__Blastocatellia	o__Pyrinomonadales	f__Pyrinomonadaceae	SPL	Freshwater	88.86	54.87	6905000	6148	994.78	62	1	29	128	68	25.63	6.26	Aerobic	Scarcity
GCA_023717605	c__Blastocatellia	o__Pyrinomonadales	f__Pyrinomonadaceae	SPL	Soil	86.34	54.53	7378535	7073	899.20	70	3	52	98	81	27.56	6.52	Aerobic	Ruderal
GCA_023958255	c__Blastocatellia	o__Pyrinomonadales	f__Pyrinomonadaceae	SPL	Engineered	91.39	54.24	2770820	2705	937.70	24	0	8	25	40	29.02	6.53	Aerobic	Scarcity
GCA_024103495	c__Blastocatellia	o__Pyrinomonadales	f__Pyrinomonadaceae	SPL	Engineered	86.55	57.23	4375970	3809	993.67	34	0	45	18	51	27.63	6.37	Aerobic	Scarcity
GCA_024330265	c__Blastocatellia	o__Pyrinomonadales	f__Pyrinomonadaceae	SPL	Marine	87.71	54.48	4797666	4235	993.51	39	7	16	62	42	34.76	6.16	Aerobic	Scarcity
GCA_024640445	c__Blastocatellia	o__Pyrinomonadales	f__Pyrinomonadaceae	SPL	Freshwater	88.75	54.55	3715511	3429	961.31	30	0	16	35	48	26.03	6.59	Aerobic	Scarcity
GCA_025054715	c__Blastocatellia	o__Pyrinomonadales	f__Pyrinomonadaceae	SPL	Freshwater	93.57	40.67	2454796	2196	1047.19	30	0	8	22	37	48.22	6.28	Aerobic	Scarcity
GCA_025054955	c__Blastocatellia	o__HR10	f__J050	SPL	Freshwater	87.30	55.74	4687160	4007	1019.82	48	0	30	31	52	41.98	5.43	Aerobic	Scarcity
GCA_025055075	c__Blastocatellia	o__HR10	f__HR10	SPL	Freshwater	92.28	61.14	3024198	2600	1074.92	37	0	4	22	36	67.78	5.74	Anaerobic	Competitor
GCA_025057015	c__Blastocatellia	o__Chloracidobacteriales	f__JADJPF01	SPL	Freshwater	93.94	46.93	3331005	2817	1113.12	36	0	36	24	36	42.87	6.79	Aerobic	Competitor
GCA_025057975	c__Blastocatellia	o__Chloracidobacteriales	f__Chloracidobacteriaceae	SPL	Freshwater	89.59	60.79	3522876	3005	1051.66	36	0	37	18	49	46.27	6.40	Aerobic	Scarcity
GCA_025060035	c__Blastocatellia	o__HR10	f__HR10	SPL	Freshwater	91.33	60.87	3239854	2820	1051.72	42	0	4	25	37	67.73	5.74	Anaerobic	Scarcity
GCA_902805935	c__Blastocatellia	o__Pyrinomonadales	f__Pyrinomonadaceae	SPL	Soil	89.59	50.52	3869462	3491	990.87	34	0	16	53	44	29.55	6.37	Aerobic	Scarcity
GCA_902825875	c__Blastocatellia	o__RBC074	f__RBC074	SPL	Engineered	90.28	53.45	7320713	5934	1114.61	74	5	28	72	71	25.27	5.81	Aerobic	Scarcity
GCA_902826385	c__Blastocatellia	o__Pyrinomonadales	f__Pyrinomonadaceae	SPL	Engineered	89.91	52.39	3691301	3877	856.07	33	0	12	50	45	24.66	6.22	Aerobic	Scarcity
GCA_902826505	c__Blastocatellia	o__Pyrinomonadales	f__Pyrinomonadaceae	SPL	Engineered	88.81	48.22	5724394	5839	870.57	55	4	16	101	66	26.83	6.66	Aerobic	Scarcity
GCA_902826795	c__Blastocatellia	o__Pyrinomonadales	f__Pyrinomonadaceae	SPL	Engineered	90.90	53.71	4151551	4037	936.36	43	1	30	57	53	25.23	6.51	Aerobic	Scarcity
GCA_903828635	c__Blastocatellia	o__RBC074	f__RBC074	SPL	Freshwater	90.72	58.56	4315207	3554	1098.47	34	0	48	47	49	31.53	6.15	Anaerobic	Scarcity
GCA_903862855	c__Blastocatellia	o__RBC074	f__RBC074	SPL	Freshwater	89.17	56.74	4597131	3861	1059.91	32	2	12	52	52	33.25	6.10	Anaerobic	Scarcity
GCA_000242615	c__Holophagae	o__Holophagales	f__Holophagaceae	NSPL	Freshwater	89.78	62.95	4127237	3590	1032.87	41	5	4	18	29	28.88	6.75	Anaerobic	Ruderal
GCA_000428885	c__Holophagae	o__Holophagales	f__Holophagaceae	NSPL	Freshwater	91.04	68.89	3289813	2923	1025.25	38	1	12	9	37	31.57	5.97	Anaerobic	Ruderal
GCA_002068835	c__Holophagae	o__Holophagales	f__Holophagaceae	NSPL	Engineered	92.73	67.77	2817428	2897	901.09	27	6	0	14	34	38.51	6.69	Anaerobic	Scarcity
GCA_002297665	c__Holophagae	o__Holophagales	f__Holophagaceae	NSPL	Engineered	89.93	69.06	3940022	3710	966.37	38	2	20	28	44	30.53	6.50	Anaerobic	Competitor
GCA_002298155	c__Holophagae	o__Holophagales	f__Holophagaceae	NSPL	Engineered	89.60	67.83	4106629	3964	932.15	31	1	16	30	46	31.23	6.26	Anaerobic	Scarcity
GCA_002319925	c__Holophagae	o__Holophagales	f__Holophagaceae	NSPL	Soil	86.31	61.22	4242989	4045	955.57	39	3	37	23	47	31.38	5.87	Anaerobic	Ruderal
GCA_002747255	c__Holophagae	o__Acanthopleuribacterales	f__Acanthopleuribacteraceae	NSPL	Host	87.06	46.46	3496648	2870	1060.98	26	1	10	19	20	26.16	6.22	Anaerobic	Scarcity
GCA_002789615	c__Holophagae	o__Thermotomaculales	f__Thermotomaculaceae	NSPL	Freshwater	90.35	49.10	2111409	2077	924.20	24	0	0	6	31	32.96	6.33	Anaerobic	Scarcity
GCA_003158505	c__Holophagae	o__Holophagales	f__Holophagaceae	NSPL	Soil	89.89	63.84	3285741	3240	913.15	35	1	20	12	46	30.23	6.05	Anaerobic	Scarcity
GCA_003160655	c__Holophagae	o__Holophagales	f__Holophagaceae	NSPL	Soil	90.09	63.81	3206208	3285	880.74	36	0	24	12	47	30.29	6.09	Anaerobic	Scarcity
GCA_003165415	c__Holophagae	o__Holophagales	f__Holophagaceae	NSPL	Soil	89.90	66.97	4841571	4520	963.78	47	2	46	16	59	29.22	5.61	Anaerobic	Competitor

GCA_003530835	c__Holophagae	o__Holophagales	f__Holophagaceae	NSPL	Freshwater	94.15	45.89	691035	728	896.05	5	0	0	1	15	25.53	6.47	Aerobic	Scarcity
GCA_003538615	c__Holophagae	o__Holophagales	f__Holophagaceae	NSPL	Freshwater	88.22	68.63	2437560	2554	858.07	25	1	4	8	27	33.61	7.03	Anaerobic	Scarcity
GCA_003650445	c__Holophagae	o__Thermotomaculales	f__Thermotomaculaceae	NSPL	Marine	91.10	49.41	2161331	1980	995.49	27	0	8	8	19	35.44	6.37	Anaerobic	Scarcity
GCA_005788175	c__Holophagae	o__Holophagales	f__Holophagaceae	NSPL	Freshwater	94.67	43.46	893751	855	989.18	7	0	0	2	10	23.51	6.09	Aerobic	Scarcity
GCA_009783055	c__Holophagae	o__Holophagales	f__Holophagaceae	NSPL	Host	90.09	44.95	2709559	2401	1016.57	30	1	8	31	23	25.07	6.47	Anaerobic	Scarcity
GCA_013151025	c__Holophagae	o__Thermotomaculales	f__Thermotomaculaceae	NSPL	Marine	91.33	48.93	2635118	2418	998.66	29	1	8	9	23	31.4	6.37	Anaerobic	Scarcity
GCA_013152385	c__Holophagae	o__Thermotomaculales	f__Thermotomaculaceae	NSPL	Marine	92.60	49.56	2574910	2321	1030.67	38	0	4	12	22	34.91	6.37	Anaerobic	Scarcity
GCA_013334395	c__Holophagae	o__Holophagales	f__Holophagaceae	NSPL	Engineered	92.26	68.17	3155907	3059	954.24	35	1	0	10	47	28.32	5.86	Anaerobic	Scarcity
GCA_013390615	c__Holophagae	o__Holophagales	f__Holophagaceae	NSPL	Engineered	91.40	67.57	3723465	3274	1044.89	38	0	12	22	46	30.56	5.98	Anaerobic	Scarcity
GCA_014189895	c__Holophagae	o__Holophagales	f__Holophagaceae	NSPL	Freshwater	94.59	46.70	1165493	1148	961.16	14	0	0	5	16	24.7	6.72	Aerobic	Competitor
GCA_015487755	c__Holophagae	o__Thermotomaculales	f__Thermotomaculaceae	NSPL	Marine	95.84	38.03	1664784	1692	944.82	31	0	0	6	16	59.25	6.44	Anaerobic	Scarcity
GCA_016178955	c__Holophagae	o__Holophagales	f__Holophagaceae	NSPL	Freshwater	90.84	65.95	4318631	3700	1060.25	46	1	27	49	50	29.47	6.45	Anaerobic	Ruderal
GCA_016192145	c__Holophagae	o__Holophagales	f__Holophagaceae	NSPL	Freshwater	91.56	66.91	3800023	3359	1036.83	43	2	10	12	38	34.19	6.49	Anaerobic	Ruderal
GCA_016195985	c__Holophagae	o__Holophagales	f__Holophagaceae	NSPL	Freshwater	91.00	65.95	4263011	3654	1061.85	47	1	27	47	50	29.58	6.45	Anaerobic	Competitor
GCA_016213735	c__Holophagae	o__Holophagales	f__Holophagaceae	NSPL	Freshwater	91.70	68.42	3591538	3124	1055.36	38	1	12	9	38	33.07	6.11	Anaerobic	Scarcity
GCA_016592575	c__Holophagae	o__Thermotomaculales	f__Thermotomaculaceae	NSPL	Marine	94.27	35.06	2317141	2108	1039.11	37	1	0	11	16	59.43	6.12	Anaerobic	Scarcity
GCA_016705945	c__Holophagae	o__Holophagales	f__Holophagaceae	NSPL	Engineered	89.95	68.00	3301456	3124	952.73	31	2	8	16	40	31.1	5.63	Anaerobic	Scarcity
GCA_016710635	c__Holophagae	o__Holophagales	f__Holophagaceae	NSPL	Engineered	89.04	68.57	3867194	3506	983.90	30	1	26	9	43	31.08	5.98	Aerobic	Scarcity
GCA_016714485	c__Holophagae	o__Holophagales	f__Holophagaceae	NSPL	Engineered	88.47	68.02	3303267	3170	923.64	34	0	8	14	42	30.51	5.79	Anaerobic	Scarcity
GCA_016714685	c__Holophagae	o__Holophagales	f__Holophagaceae	NSPL	Engineered	89.08	67.55	3466241	3201	966.08	38	2	8	17	41	31.02	6.26	Anaerobic	Scarcity
GCA_016715205	c__Holophagae	o__Holophagales	f__Holophagaceae	NSPL	Engineered	90.82	60.44	3757211	3515	973.02	36	2	20	21	46	29.48	6.20	Anaerobic	Scarcity
GCA_016720465	c__Holophagae	o__Holophagales	f__Holophagaceae	NSPL	Engineered	89.76	63.53	3523586	3005	1053.68	28	1	8	12	35	27.44	6.54	Anaerobic	Scarcity
GCA_016721195	c__Holophagae	o__Holophagales	f__Holophagaceae	NSPL	Engineered	89.81	66.98	3749529	3377	998.16	35	0	8	23	44	30.57	5.71	Anaerobic	Competitor
GCA_016722505	c__Holophagae	o__Holophagales	f__Holophagaceae	NSPL	Engineered	90.69	59.20	3629636	3257	1012.78	31	0	8	15	42	27.17	6.53	Anaerobic	Scarcity
GCA_017377855	c__Holophagae	o__Acanthopleuribacterales	f__Acanthopleuribacteraceae	NSPL	Host	85.02	57.32	10848561	6863	1344.77	81	2	292	47	31	31.22	5.77	Aerobic	Competitor
GCA_017498545	c__Holophagae	o__Acanthopleuribacterales	f__Acanthopleuribacteraceae	NSPL	Host	83.72	60.16	11786365	7107	1389.85	102	4	310	43	33	27.95	6.09	Aerobic	Ruderal
GCA_017859955	c__Holophagae	o__Holophagales	f__Holophagaceae	NSPL	Soil	90.52	62.85	3762762	3539	962.67	34	3	12	20	37	29.28	6.62	Anaerobic	Ruderal
GCA_017859995	c__Holophagae	o__Holophagales	f__Holophagaceae	NSPL	Soil	91.92	68.60	3226663	3121	951.23	39	1	8	14	45	32.34	6.62	Anaerobic	Scarcity
GCA_017998635	c__Holophagae	o__Holophagales	f__Holophagaceae	NSPL	Engineered	92.19	67.85	2416140	2555	872.19	32	0	12	16	38	31.77	6.43	Anaerobic	Scarcity
GCA_018269025	c__Holophagae	o__Holophagales	f__Holophagaceae	NSPL	Engineered	93.37	65.67	2306895	2316	931.21	27	0	12	4	29	31.99	5.80	Anaerobic	Competitor
GCA_019135905	c__Holophagae	o__Holophagales	f__Holophagaceae	NSPL	Engineered	93.45	68.16	2670364	2597	961.05	28	3	0	12	26	38.55	6.69	Anaerobic	Scarcity
GCA_019449415	c__Holophagae	o__Holophagales	f__Holophagaceae	NSPL	Engineered	92.79	67.86	2153008	2024	987.92	27	1	4	6	31	31.99	6.69	Anaerobic	Competitor
GCA_020048105	c__Holophagae	o__Holophagales	f__Holophagaceae	NSPL	Freshwater	92.52	58.36	2954443	2788	981.72	31	1	16	18	41	28.76	6.47	Anaerobic	Competitor
GCA_020439745	c__Holophagae	o__Acanthopleuribacterales	f__Acanthopleuribacteraceae	NSPL	Engineered	93.79	52.28	5135038	4345	1111.02	52	0	28	21	36	26.83	5.98	Aerobic	Scarcity
GCA_020439765	c__Holophagae	o__Acanthopleuribacterales	f__Acanthopleuribacteraceae	NSPL	Engineered	91.40	51.50	5785093	5096	1038.91	55	0	24	32	40	27.31	6.55	Aerobic	Ruderal
GCA_020622065	c__Holophagae	o__Holophagales	f__Holophagaceae	NSPL	Engineered	91.42	67.09	3348603	3039	1008.64	30	2	8	9	41	30.95	6.66	Anaerobic	Competitor
GCA_020637335	c__Holophagae	o__Acanthopleuribacterales	f__Acanthopleuribacteraceae	NSPL	Engineered	91.20	51.42	4393739	3625	1107.18	46	1	16	25	26	27.24	6.42	Anaerobic	Competitor
GCA_020848495	c__Holophagae	o__Holophagales	f__Holophagaceae	NSPL	Engineered	93.11	67.93	2339256	2422	899.95	31	2	8	15	33	32.94	5.63	Anaerobic	Scarcity
GCA_021286765	c__Holophagae	o__Holophagales	f__Holophagaceae	NSPL	Engineered	92.96	66.99	3152536	2813	1042.82	33	0	0	14	37	31.34	6.15	Anaerobic	Scarcity
GCA_021287085	c__Holophagae	o__Holophagales	f__Holophagaceae	NSPL	Engineered	93.57	63.28	2426400	2405	942.47	35	0	16	16	33	34.01	6.54	Anaerobic	Scarcity
GCA_021778785	c__Holophagae	o__Holophagales	f__Holophagaceae	NSPL	Engineered	93.03	70.14	2417783	2595	867.37	28	1	8	14	39	34.63	6.15	Anaerobic	Scarcity
GCA_021786615	c__Holophagae	o__Holophagales	f__Holophagaceae	NSPL	Engineered	90.11	61.89	2876990	2736	946.52	26	4	20	9	35	29.37	7.12	Anaerobic	Competitor
GCA_021794095	c__Holophagae	o__Holophagales	f__Holophagaceae	NSPL	Engineered	92.37	67.21	2993436	2689	1029.46	25	2	4	13	36	33.39	6.53	Anaerobic	Scarcity
GCA_021802985	c__Holophagae	o__Holophagales	f__Holophagaceae	NSPL	Engineered	92.02	67.31	2301304	2174	974.97	26	3	4	13	27	33.7	6.66	Anaerobic	Competitor
GCA_021812805	c__Holophagae	o__Holophagales	f__Holophagaceae	NSPL	Engineered	92.12	67.69	2907361	2792	959.97	34	10	4	9	36	33.57	6.11	Anaerobic	Competitor
GCA_022708985	c__Holophagae	o__Holophagales	f__Holophagaceae	NSPL	Engineered	92.33	67.89	2591088	2563	936.29	32	5	0	12	31	38.8	6.44	Anaerobic	Scarcity
GCA_022808725	c__Holophagae	o__Holophagales	f__Holophagaceae	NSPL	Soil	91.30	70.57	4294032	3647	1074.86	39	0	34	24	46	34.94	6.18	Anaerobic	Competitor
GCA_022808755	c__Holophagae	o__Holophagales	f__Holophagaceae	NSPL	Soil	91.03	70.37	4345132	3672	1076.92	35	1	24	25	46	34.92	6.14	Anaerobic	Ruderal
GCA_022808775	c__Holophagae	o__Holophagales	f__Holophagaceae	NSPL	Soil	90.98	68.08	3428306	3045	1025.10	36	2	4	16	45	31.54	6.30	Anaerobic	Competitor
GCA_022808815	c__Holophagae	o__Holophagales	f__Holophagaceae	NSPL	Soil	91.24	68.10	3407788	3042	1023.01	35	1	12	15	44	31.81	6.30	Anaerobic	Competitor
GCA_022808835	c__Holophagae	o__Holophagales	f__Holophagaceae	NSPL	Soil	91.38	68.12	3462833	3066	1032.99	36	1	8	19	43	31.63	6.55	Anaerobic	Ruderal
GCA_022808865	c__Holophagae	o__Holophagales	f__Holophagaceae	NSPL	Soil	92.18	68.15	3230256	2863	1041.02	34	1	12	7	44	32.33	6.38	Anaerobic	Ruderal
GCA_022808885	c__Holophagae	o__Holophagales	f__Holophagaceae	NSPL	Soil	90.73	68.05	3421064	3034	1024.10	39	3	8	17	44	32.04	6.24	Anaerobic	Scarcity
GCA_022808935	c__Holophagae	o__Holophagales	f__Holophagaceae	NSPL	Soil	91.22	68.11	3356763	2972	1031.35	36	2	8	20	41	32.04	6.57	Anaerobic	Competitor
GCA_023257195	c__Holophagae	o__Holophagales	f__Holophagaceae	NSPL	Freshwater	93.24	46.11	1198512	1171	953.40	12	0	0	5	15	23.73	6.47	Aerobic	Scarcity
GCA_023701165	c__Holophagae	o__Holophagales	f__Holophagaceae	NSPL	Freshwater	93.03	69.28	1743209	1849	877.23	15	0	0	7	25	33.46	6.14	Anaerobic	Scarcity
GCA_024609265	c__Holophagae	o__Holophagales	f__Holophagaceae	NSPL	Soil	91.20	66.72	3285156	2997	999.49	40	7	8	10	39	31.94	6.49	Anaerobic	Scarcity
GCA_024609325	c__Holophagae	o__Holophagales	f__Holophagaceae	NSPL	Soil	90.97	65.86	3318792	2978	1014.75	37	1	20	14	42	31.8	6.36	Anaerobic	Competitor
GCA_903819625	c__Holophagae	o__Holophagales	f__Holophagaceae	NSPL	Freshwater	91.97	67.29	3113120	3202	893.53	33	0	12	13	48	28.5	6.40	Anaerobic	Scarcity
GCA_903820395	c__Holophagae	o__Holophagales	f__Holophagaceae	NSPL	Freshwater	91.13	67.74	3624777	3141	1052.21	42	0	4	13	47	27.61	5.74	Anaerobic	Scarcity
GCA_903820535	c__Holophagae	o__Holophagales	f__Holophagaceae	NSPL	Freshwater	91.21	67.79	3598903	3114	1054.94	40	0	4	13	47	27.63	5.74	Anaerobic	Ruderal
GCA_903820555	c__Holophagae	o__Holophagales	f__Holophagaceae	NSPL	Freshwater	94.82	46.78	1212225	1226	938.51	13	0	0	5	15	24.91	6.69	Aerobic	Scarcity
GCA_903821245	c__Holophagae	o__Holophagales	f__Holophagaceae	NSPL	Freshwater	90.82	63.56	3065987	3390	820.64	42	1	8	20	51	29.7	5.89	Anaerobic	Scarcity
GCA_903823075	c__Holophagae	o__Holophagales	f__Holophagaceae	NSPL	Freshwater	94.64	45.90	947791	957	938.17	12	1	0	3	13	24.57	6.69	Aerobic	Competitor
GCA_903823375	c__Holophagae	o__Holophagales	f__Holophagaceae	NSPL	Freshwater	91.12	67.74	3628377	3143	1052.37	42	0	4	13	47	27.66	5.74	Anaerobic	Scarcity
GCA_903823525	c__Holophagae	o__Holophagales	f__Holophagaceae	NSPL	Freshwater	91.86	65.94	3318801	3417	892.04	45	0	12	15	57	28.52	5.88	Anaerobic	Scarcity
GCA_903823985	c__Holophagae	o__Holophagales	f__Holophagaceae	NSPL	Freshwater	91.21	67.11	3619303	3148	1047.99	39	2	4	11	48	27.3	5.79	Anaerobic	Ruderal
GCA_903824115	c__Holophagae	o__Holophagales	f__Holophagaceae	NSPL	Freshwater	91.05	67.61	3682529	3181	1054.28	41	0	4	12	47	27.61	5.74	Anaerobic	Competitor
GCA_903824975	c__Holophagae	o__Holophagales	f__Holophagaceae	NSPL	Freshwater	91.56	67.51	3315142	2909	1043.58	37	2	4	11	46	27.34	6.32	Anaerobic	Competitor
GCA_903826035	c__Holophagae	o__Holophagales	f__Holophagaceae	NSPL	Freshwater	91.17	67.77	3601744	3115	1054.90	40	0	4	12	47	27.66	5.74	Anaerobic	Scarcity
GCA_903826495	c__Holophagae	o__Holophagales	f__Holophagaceae	NSPL	Freshwater	90.97	67.52	3728694	3221	1053.27	42	0	8	13	47	27.61	5.74	Anaerobic	Scarcity
GCA_903827515	c__Holophagae	o__Holophagales	f__Holophagaceae	NSPL	Freshwater	91.10	65.08	4077681	3822	972.32	53	3	12	13	72	28.24	5.88	Anaerobic	Scarcity
GCA_903829455	c__Holophagae	o__Holophagales	f__Holophagaceae	NSPL	Freshwater	94.83	46.86	1177622	1205	927.76	13	1	0	5	19	25.05	6.69	Aerobic	Scarcity
GCA_903831225	c__Holophagae	o__Holophagales	f__Holophagaceae	NSPL	Freshwater	91.84	67.14	3272853	3128	960.75	33	1	4	10	50	27.99	5.64	Anaerobic	Scarcity
GCA_903831365	c__Holophagae	o__Holophagales	f__Holophagaceae	NSPL	Freshwater	92.10	67.53	2852286	2953	889.04	35	0	8	13	46	29.33	5.84	Anaerobic	Scarcity
GCA_903832585	c__Holophagae	o__Holophagales	f__Holophagaceae	NSPL	Freshwater	91.06	67.67	3655181	3165	1051.25	43	0	4	13	47	27.49	5.74	Anaerobic	Ruderal
GCA_903832715	c__Holophagae	o__Holophagales	f__Holophagaceae	NSPL	Freshwater	94.51	47.73	835051	822	960.62	9	1	0	3	12	27.12	6.00	Aerobic	Scarcity
GCA_903833415	c__Holophagae	o__Holophagales	f__Holophagaceae	NSPL	Freshwater	91.01	67.61	3685618	3194	1050.37	42	0	4	13	47	27.74	5.74	Anaerobic	Competitor
GCA_903836145	c__Holophagae	o__Holophagales	f__Holophagaceae	NSPL	Freshwater	91.16	67.74	3624153	3138	1053.60	42	0	4	13	47	27.57	5.74	Anaerobic	Scarcity
GCA_903836495	c__Holophagae	o__Holophagales	f__Holophagaceae	NSPL	Freshwater	91.11	65.61	3750510	3376	1011.69	47	2	12	13	58	28.48	6.25	Anaerobic	Scarcity
GCA_903837115	c__Holophagae	o__Holophagales	f__Holophagaceae	NSPL	Freshwater	91.36	65.34	3685698	3815	881.07	40	1	4	15	74	28.12	6.28	Anaerobic	Scarcity
GCA_903837725	c__Holophagae	o__Holophagales	f__Holophagaceae	NSPL	Freshwater	91.15	65.64	3740243	3369	1011.11	47	2	12	13	59	28.29	6.25	Anaerobic	Scarcity
GCA_903837865	c__Holophagae	o__Holophagales	f__Holophagaceae	NSPL	Freshwater	91.80	67.28	3075072	3138	899.27	35	0	8	10	46	28.72	5.64	Anaerobic	Scarcity
GCA_903839095	c__Holophagae	o__Holophagales	f__Holophagaceae	NSPL	Freshwater	91.14	67.74	3630769	3144	1052.68	42	0	4	13	47	27.59	5.74	Anaerobic	Ruderal
GCA_903839205	c__Holophagae	o__Holophagales	f__Holophagaceae	NSPL	Freshwater	92.04	67.47	3080620	2957	958.43	37	0	12	9	45	30.01	6.30	Anaerobic	Scarcity
GCA_903839295	c__Holophagae	o__Holophagales	f__Holophagaceae	NSPL	Freshwater	91.17	67.77	3609893	3126	1053.35	41	0	4	13	47	27.66	5.74	Anaerobic	Competitor
GCA_903840385	c__Holophagae	o__Holophagales	f__Holophagaceae	NSPL	Freshwater	92.06	67.81	3106673	2837	1008.44	39	0	12	13	43	28.35	5.84	Anaerobic	Scarcity

GCA_903840675	c__Holophagae	o__Holophagales	f__Holophagaceae	NSPL	Freshwater	91.04	67.61	3692293	3193	1052.94	42	0	4	13	47	27.52	5.74	Anaerobic	Scarcity
GCA_903841285	c__Holophagae	o__Holophagales	f__Holophagaceae	NSPL	Freshwater	91.08	67.61	3678994	3182	1053.21	41	0	4	12	47	27.6	5.74	Anaerobic	Scarcity
GCA_903841555	c__Holophagae	o__Holophagales	f__Holophagaceae	NSPL	Freshwater	91.54	65.85	3678806	3376	997.03	46	0	12	13	59	28.31	5.88	Anaerobic	Competitor
GCA_903841945	c__Holophagae	o__Holophagales	f__Holophagaceae	NSPL	Freshwater	91.51	65.78	3715524	3342	1015.20	46	1	12	15	56	28.12	5.88	Anaerobic	Scarcity
GCA_903843615	c__Holophagae	o__Holophagales	f__Holophagaceae	NSPL	Freshwater	91.19	67.77	3600020	3116	1054.35	40	0	4	12	47	27.64	5.74	Anaerobic	Ruderal
GCA_903843705	c__Holophagae	o__Holophagales	f__Holophagaceae	NSPL	Freshwater	91.03	66.73	3748633	3549	961.19	37	2	8	9	55	28.58	5.88	Anaerobic	Scarcity
GCA_903844125	c__Holophagae	o__Holophagales	f__Holophagaceae	NSPL	Freshwater	91.12	67.65	3662101	3166	1054.50	40	0	4	12	47	27.64	5.74	Anaerobic	Competitor
GCA_903845715	c__Holophagae	o__Holophagales	f__Holophagaceae	NSPL	Freshwater	91.11	67.64	3663508	3169	1053.77	40	0	4	12	47	27.62	5.74	Anaerobic	Scarcity
GCA_903846255	c__Holophagae	o__Holophagales	f__Holophagaceae	NSPL	Freshwater	91.15	67.74	3609968	3125	1053.37	41	0	4	12	47	27.68	5.74	Anaerobic	Scarcity
GCA_903846785	c__Holophagae	o__Holophagales	f__Holophagaceae	NSPL	Freshwater	91.15	67.70	3641847	3153	1053.01	40	0	4	12	47	27.63	5.74	Anaerobic	Scarcity
GCA_903849145	c__Holophagae	o__Holophagales	f__Holophagaceae	NSPL	Freshwater	92.01	67.20	3120171	3179	902.49	30	1	4	9	52	28.2	5.64	Anaerobic	Scarcity
GCA_903850415	c__Holophagae	o__Holophagales	f__Holophagaceae	NSPL	Freshwater	91.42	67.43	3592998	3116	1053.18	38	1	4	11	49	27.69	6.09	Anaerobic	Ruderal
GCA_903850645	c__Holophagae	o__Holophagales	f__Holophagaceae	NSPL	Freshwater	91.81	67.15	3529309	3337	971.06	37	3	4	11	50	27.73	5.64	Anaerobic	Scarcity
GCA_903856355	c__Holophagae	o__Holophagales	f__Holophagaceae	NSPL	Freshwater	91.07	65.15	4164592	3729	1015.72	54	6	12	16	57	28.84	6.25	Anaerobic	Scarcity
GCA_903857195	c__Holophagae	o__Holophagales	f__Holophagaceae	NSPL	Freshwater	91.19	67.77	3603921	3120	1054.07	40	0	4	12	47	27.66	5.74	Anaerobic	Competitor
GCA_903857495	c__Holophagae	o__Holophagales	f__Holophagaceae	NSPL	Freshwater	90.87	67.59	3695687	3197	1050.62	42	0	4	13	47	27.76	5.74	Anaerobic	Competitor
GCA_903859585	c__Holophagae	o__Holophagales	f__Holophagaceae	NSPL	Freshwater	91.78	67.10	2562649	2472	951.65	29	1	4	6	39	27.14	5.54	Anaerobic	Scarcity
GCA_903859805	c__Holophagae	o__Holophagales	f__Holophagaceae	NSPL	Freshwater	91.56	65.90	3648152	3344	998.06	44	0	12	13	57	27.88	6.25	Anaerobic	Scarcity
GCA_903860015	c__Holophagae	o__Holophagales	f__Holophagaceae	NSPL	Freshwater	90.97	67.54	3719961	3217	1052.07	43	0	4	13	47	27.64	5.74	Anaerobic	Scarcity
GCA_903860595	c__Holophagae	o__Holophagales	f__Holophagaceae	NSPL	Freshwater	91.17	67.74	3610731	3124	1054.53	41	0	4	12	47	27.74	5.74	Anaerobic	Competitor
GCA_903863195	c__Holophagae	o__Holophagales	f__Holophagaceae	NSPL	Freshwater	91.19	68.05	3599456	3333	984.36	36	0	8	8	52	29.12	5.98	Anaerobic	Scarcity
GCA_903863765	c__Holophagae	o__Holophagales	f__Holophagaceae	NSPL	Freshwater	91.17	67.72	3625758	3136	1054.23	41	0	4	12	47	27.61	5.74	Anaerobic	Ruderal
GCA_903864105	c__Holophagae	o__Holophagales	f__Holophagaceae	NSPL	Freshwater	94.37	46.85	1158957	1162	941.25	15	0	0	5	16	24.65	6.69	Aerobic	Scarcity
GCA_903864235	c__Holophagae	o__Holophagales	f__Holophagaceae	NSPL	Freshwater	91.12	67.64	3662172	3169	1053.16	40	1	4	12	47	27.67	5.74	Anaerobic	Competitor
GCA_903865165	c__Holophagae	o__Holophagales	f__Holophagaceae	NSPL	Freshwater	91.46	64.31	3337808	3237	942.58	35	0	16	15	44	28.52	6.32	Anaerobic	Scarcity
GCA_903865605	c__Holophagae	o__Holophagales	f__Holophagaceae	NSPL	Freshwater	91.20	67.77	3602994	3117	1054.95	40	0	4	12	47	27.63	5.74	Anaerobic	Scarcity
GCA_903868115	c__Holophagae	o__Holophagales	f__Holophagaceae	NSPL	Freshwater	91.74	62.93	3424645	3326	941.88	34	1	4	26	43	30.56	5.64	Anaerobic	Scarcity
GCA_903868765	c__Holophagae	o__Holophagales	f__Holophagaceae	NSPL	Freshwater	91.14	67.73	3629677	3145	1051.64	42	0	4	13	47	27.6	5.74	Anaerobic	Scarcity
GCA_903868785	c__Holophagae	o__Holophagales	f__Holophagaceae	NSPL	Freshwater	91.86	67.37	3214722	3241	911.39	36	1	8	15	51	29.09	5.84	Anaerobic	Scarcity
GCA_903868965	c__Holophagae	o__Holophagales	f__Holophagaceae	NSPL	Freshwater	91.22	67.77	3604001	3120	1054.54	40	0	4	12	47	27.54	5.74	Anaerobic	Scarcity
GCA_903870635	c__Holophagae	o__Holophagales	f__Holophagaceae	NSPL	Freshwater	91.58	65.93	3600759	3310	995.97	44	0	12	12	58	28.15	5.88	Anaerobic	Scarcity
GCA_903870935	c__Holophagae	o__Holophagales	f__Holophagaceae	NSPL	Freshwater	91.05	67.61	3692954	3198	1051.24	42	0	4	13	47	27.7	5.74	Anaerobic	Competitor
GCA_903871515	c__Holophagae	o__Holophagales	f__Holophagaceae	NSPL	Freshwater	91.09	67.64	3664066	3166	1054.35	40	0	4	12	47	27.76	5.74	Anaerobic	Scarcity
GCA_903872635	c__Holophagae	o__Holophagales	f__Holophagaceae	NSPL	Freshwater	91.32	67.84	3556913	3193	1016.94	38	0	4	13	51	27.71	5.57	Anaerobic	Scarcity
GCA_903872645	c__Holophagae	o__Holophagales	f__Holophagaceae	NSPL	Freshwater	91.21	67.77	3604154	3122	1053.42	40	0	4	12	47	27.58	5.74	Anaerobic	Ruderal
GCA_903873655	c__Holophagae	o__Holophagales	f__Holophagaceae	NSPL	Freshwater	91.12	64.01	3473286	3364	940.26	37	2	16	16	43	28.3	6.32	Anaerobic	Scarcity
GCA_903875405	c__Holophagae	o__Holophagales	f__Holophagaceae	NSPL	Freshwater	91.29	65.68	3747097	3410	1001.34	45	2	12	13	56	28.09	6.25	Anaerobic	Competitor
GCA_903876405	c__Holophagae	o__Holophagales	f__Holophagaceae	NSPL	Freshwater	91.10	67.71	3639232	3151	1051.99	43	0	4	13	47	27.65	5.74	Anaerobic	Ruderal
GCA_903876615	c__Holophagae	o__Holophagales	f__Holophagaceae	NSPL	Freshwater	91.62	67.61	2784079	2844	894.54	32	4	4	11	47	26.58	6.65	Anaerobic	Scarcity
GCA_903877655	c__Holophagae	o__Holophagales	f__Holophagaceae	NSPL	Freshwater	91.19	67.77	3600824	3119	1053.56	40	0	4	12	47	27.68	5.74	Anaerobic	Scarcity
GCA_903877725	c__Holophagae	o__Holophagales	f__Holophagaceae	NSPL	Freshwater	91.14	67.73	3629782	3146	1051.36	42	0	4	13	47	27.67	5.74	Anaerobic	Competitor
GCA_903877745	c__Holophagae	o__Holophagales	f__Holophagaceae	NSPL	Freshwater	90.69	64.86	4124394	3869	967.70	49	4	12	15	75	28.13	5.88	Anaerobic	Scarcity
GCA_903878845	c__Holophagae	o__Holophagales	f__Holophagaceae	NSPL	Freshwater	91.17	67.77	3612471	3132	1052.34	41	0	4	13	47	27.66	5.74	Anaerobic	Scarcity
GCA_903879545	c__Holophagae	o__Holophagales	f__Holophagaceae	NSPL	Freshwater	90.98	67.49	3733603	3227	1053.16	42	0	8	13	47	27.54	5.74	Anaerobic	Competitor
GCA_903880025	c__Holophagae	o__Holophagales	f__Holophagaceae	NSPL	Freshwater	90.63	64.92	4173761	3701	1021.40	47	0	16	18	46	28.69	6.06	Anaerobic	Ruderal
GCA_903880425	c__Holophagae	o__Holophagales	f__Holophagaceae	NSPL	Freshwater	91.20	67.77	3602848	3119	1054.20	40	0	4	12	47	27.65	5.74	Anaerobic	Ruderal
GCA_903880675	c__Holophagae	o__Holophagales	f__Holophagaceae	NSPL	Freshwater	91.80	67.10	2490836	2316	987.74	27	1	8	9	34	27.76	5.82	Anaerobic	Competitor
GCA_903883145	c__Holophagae	o__Holophagales	f__Holophagaceae	NSPL	Freshwater	91.81	67.17	3314960	3159	962.97	31	0	4	10	55	27.98	5.64	Anaerobic	Scarcity
GCA_903883395	c__Holophagae	o__Holophagales	f__Holophagaceae	NSPL	Freshwater	91.77	65.94	3557794	3473	939.88	47	0	12	15	64	28.16	5.64	Anaerobic	Scarcity
GCA_903884175	c__Holophagae	o__Holophagales	f__Holophagaceae	NSPL	Freshwater	92.05	67.45	2991231	3059	899.95	37	0	8	13	53	28.48	5.84	Anaerobic	Scarcity
GCA_903884655	c__Holophagae	o__Holophagales	f__Holophagaceae	NSPL	Freshwater	91.05	65.13	3920214	3465	1029.81	41	0	16	19	45	28.92	6.27	Anaerobic	Ruderal
GCA_903886655	c__Holophagae	o__Holophagales	f__Holophagaceae	NSPL	Freshwater	92.03	67.67	2861385	2980	883.36	37	0	8	12	46	29.94	5.84	Anaerobic	Scarcity
GCA_903887115	c__Holophagae	o__Holophagales	f__Holophagaceae	NSPL	Freshwater	91.65	64.46	3209740	3201	919.36	41	1	12	16	46	27.96	6.40	Anaerobic	Scarcity
GCA_903888075	c__Holophagae	o__Holophagales	f__Holophagaceae	NSPL	Freshwater	91.01	67.52	3725449	3220	1053.23	42	0	8	13	47	27.59	5.74	Anaerobic	Scarcity
GCA_903888125	c__Holophagae	o__Holophagales	f__Holophagaceae	NSPL	Freshwater	91.08	67.64	3676765	3177	1054.21	41	0	4	13	47	27.68	5.74	Anaerobic	Ruderal
GCA_903888495	c__Holophagae	o__Holophagales	f__Holophagaceae	NSPL	Freshwater	95.06	46.62	1097615	1144	912.14	15	0	0	5	18	24.75	6.69	Aerobic	Scarcity
GCA_903890595	c__Holophagae	o__Holophagales	f__Holophagaceae	NSPL	Freshwater	91.70	67.14	3193077	3154	928.15	34	0	8	13	47	29.22	6.40	Anaerobic	Scarcity
GCA_903891855	c__Holophagae	o__Holophagales	f__Holophagaceae	NSPL	Freshwater	90.71	66.93	3880625	3579	983.31	40	2	8	11	53	27.66	6.09	Anaerobic	Scarcity
GCA_903892555	c__Holophagae	o__Holophagales	f__Holophagaceae	NSPL	Freshwater	91.62	65.57	3864533	3464	1020.54	48	3	12	15	57	28.18	6.25	Anaerobic	Scarcity
GCA_903893385	c__Holophagae	o__Holophagales	f__Holophagaceae	NSPL	Freshwater	91.46	68.19	3467830	3324	954.36	34	1	8	8	53	28.62	5.64	Anaerobic	Scarcity
GCA_903893415	c__Holophagae	o__Holophagales	f__Holophagaceae	NSPL	Freshwater	90.99	67.62	3687537	3187	1052.09	42	0	4	13	47	27.6	5.74	Anaerobic	Competitor
GCA_903893685	c__Holophagae	o__Holophagales	f__Holophagaceae	NSPL	Freshwater	90.96	66.82	3700676	3212	1047.64	37	2	4	10	47	27.79	6.09	Anaerobic	Competitor
GCA_903893695	c__Holophagae	o__Holophagales	f__Holophagaceae	NSPL	Freshwater	90.82	65.14	4247475	3917	984.79	52	4	16	16	71	28.29	5.88	Anaerobic	Scarcity
GCA_903893705	c__Holophagae	o__Holophagales	f__Holophagaceae	NSPL	Freshwater	91.20	67.77	3606621	3121	1054.71	40	0	4	12	47	27.58	5.74	Anaerobic	Scarcity
GCA_903894055	c__Holophagae	o__Holophagales	f__Holophagaceae	NSPL	Freshwater	91.12	67.64	3664835	3169	1054.91	40	0	4	12	47	27.62	5.74	Anaerobic	Competitor
GCA_903894365	c__Holophagae	o__Holophagales	f__Holophagaceae	NSPL	Freshwater	94.54	46.84	1168885	1159	953.56	12	0	0	5	14	24.88	6.69	Aerobic	Scarcity
GCA_903896485	c__Holophagae	o__Holophagales	f__Holophagaceae	NSPL	Freshwater	91.21	67.77	3603742	3122	1053.62	40	1	4	12	47	27.62	5.74	Anaerobic	Ruderal
GCA_903896565	c__Holophagae	o__Holophagales	f__Holophagaceae	NSPL	Freshwater	91.51	67.22	3312186	2885	1050.13	36	1	4	8	40	27.81	6.09	Anaerobic	Competitor
GCA_903897625	c__Holophagae	o__Holophagales	f__Holophagaceae	NSPL	Freshwater	91.74	67.55	3248075	2959	1007.64	36	0	16	15	46	28.76	5.84	Anaerobic	Scarcity
GCA_903898575	c__Holophagae	o__Holophagales	f__Holophagaceae	NSPL	Freshwater	91.76	65.92	3536620	3410	952.01	44	2	12	13	60	28.36	5.88	Anaerobic	Scarcity
GCA_903899055	c__Holophagae	o__Holophagales	f__Holophagaceae	NSPL	Freshwater	91.73	65.99	3467583	3160	1005.12	43	0	12	12	51	27.91	6.25	Anaerobic	Scarcity
GCA_903900385	c__Holophagae	o__Holophagales	f__Holophagaceae	NSPL	Freshwater	91.08	65.26	4148175	3814	989.77	55	3	12	16	64	28.32	5.88	Anaerobic	Scarcity
GCA_903900395	c__Holophagae	o__Holophagales	f__Holophagaceae	NSPL	Freshwater	91.21	65.73	3746485	3368	1013.96	44	0	12	14	59	28.28	6.25	Anaerobic	Competitor
GCA_903900515	c__Holophagae	o__Holophagales	f__Holophagaceae	NSPL	Freshwater	91.50	67.27	3563308	3085	1056.14	40	1	4	11	48	27.39	5.79	Anaerobic	Competitor
GCA_903901535	c__Holophagae	o__Holophagales	f__Holophagaceae	NSPL	Freshwater	91.20	67.77	3614665	3133	1052.66	41	0	4	13	47	27.57	5.74	Anaerobic	Scarcity
GCA_903901715	c__Holophagae	o__Holophagales	f__Holophagaceae	NSPL	Freshwater	91.38	65.77	3706427	3391	997.91	47	0	12	13	57	28.26	5.88	Anaerobic	Scarcity
GCA_903903165	c__Holophagae	o__Holophagales	f__Holophagaceae	NSPL	Freshwater	91.47	65.89	3537022	3214	1005.73	46	0	12	13	55	27.85	6.28	Anaerobic	Competitor
GCA_903905085	c__Holophagae	o__Holophagales	f__Holophagaceae	NSPL	Freshwater	91.69	64.53	2980092	3124	873.84	32	1	8	15	46	27.73	6.15	Anaerobic	Scarcity
GCA_903906445	c__Holophagae	o__Holophagales	f__Holophagaceae	NSPL	Freshwater	91.41	65.65	3826069	3458	1009.36	47	0	12	14	59	28.1	5.88	Anaerobic	Competitor
GCA_903908045	c__Holophagae	o__Holophagales	f__Holophagaceae	NSPL	Freshwater	91.31	65.20	3705405	3340	1012.74	39	0	12	18	44	29.1	6.27	Anaerobic	Scarcity
GCA_903908895	c__Holophagae	o__Holophagales	f__Holophagaceae	NSPL	Freshwater	91.22	65.86	3641180	3282	1011.38	44	2	12	13	57	28.21	5.88	Anaerobic	Scarcity
GCA_903909095	c__Holophagae	o__Holophagales	f__Holophagaceae	NSPL	Freshwater	91.21	67.77	3602525	3121	1053.59	40	0	4	12	47	27.61	5.74	Anaerobic	Competitor
GCA_903910905	c__Holophagae	o__Holophagales	f__Holophagaceae	NSPL	Freshwater	90.98	67.52	3722123	3216	1053.51	42	0	8	13	47	27.6	5.74	Anaerobic	Competitor
GCA_903912205	c__Holophagae	o__Holophagales	f__Holophagaceae	NSPL	Freshwater	91.05	67.61	3695653	3199	1052.02	42	0	4	13	47	27.55	5.74	Anaerobic	Ruderal
GCA_903912235	c__Holophagae	o__Holophagales	f__Holophagaceae	NSPL	Freshwater	94.70	46.78	1174810	1187	938.14	12	0	0	5	15	24.68	6.69	Aerobic	Scarcity
GCA_903912815	c__Holophagae	o__Holophagales	f__Holophagaceae	NSPL	Freshwater	91.12	67.67	3653204	3167	1051.22	43	0	4	13	48	27.43	5.74	Anaerobic	Competitor
GCA_903913215	c__Holophagae	o__Holophagales	f__Holophagaceae	NSPL	Freshwater	90.61	64.98	3902199	3422	1034.28	46	3	20	15	61	28.01	5.88	Anaerobic	Scarcity

GCA_903913465	c__Holophagae	o__Holophagales	f__Holophagaceae	NSPL	Freshwater	91.57	65.78	3663396	3546	944.99	44	1	12	13	62	28.63	5.88	Anaerobic	Scarcity
GCA_903916335	c__Holophagae	o__Holophagales	f__Holophagaceae	NSPL	Freshwater	91.20	68.02	3525222	3332	964.32	33	1	8	8	47	28.64	5.98	Anaerobic	Scarcity
GCA_903917165	c__Holophagae	o__Holophagales	f__Holophagaceae	NSPL	Freshwater	91.67	67.19	2804134	2612	984.99	31	1	8	8	35	27.65	5.68	Anaerobic	Scarcity
GCA_903917195	c__Holophagae	o__Holophagales	f__Holophagaceae	NSPL	Freshwater	91.38	67.00	3529936	3429	940.55	41	2	16	13	49	28.4	6.40	Anaerobic	Scarcity
GCA_903918185	c__Holophagae	o__Holophagales	f__Holophagaceae	NSPL	Freshwater	92.36	66.26	3034803	2771	1012.41	33	0	12	17	51	29.39	5.88	Anaerobic	Scarcity
GCA_903919165	c__Holophagae	o__Holophagales	f__Holophagaceae	NSPL	Freshwater	90.71	63.76	3179760	3367	854.27	37	6	4	19	53	30.69	5.97	Anaerobic	Scarcity
GCA_903919275	c__Holophagae	o__Holophagales	f__Holophagaceae	NSPL	Freshwater	92.03	67.74	2607991	2721	881.69	39	0	8	8	47	29.02	5.68	Anaerobic	Competitor
GCA_903922015	c__Holophagae	o__Holophagales	f__Holophagaceae	NSPL	Freshwater	91.13	67.71	3618823	3133	1053.36	41	0	4	12	47	27.58	5.74	Anaerobic	Competitor
GCA_903924355	c__Holophagae	o__Holophagales	f__Holophagaceae	NSPL	Freshwater	91.96	64.61	2930721	3089	870.70	38	1	16	16	43	28.49	5.73	Anaerobic	Scarcity
GCA_903925375	c__Holophagae	o__Holophagales	f__Holophagaceae	NSPL	Freshwater	91.10	67.62	3667376	3180	1051.08	41	1	4	12	47	27.81	5.74	Anaerobic	Scarcity
GCA_903925945	c__Holophagae	o__Holophagales	f__Holophagaceae	NSPL	Freshwater	90.97	68.00	3418759	3193	973.07	37	0	4	8	49	29.24	5.98	Anaerobic	Scarcity
GCA_903927445	c__Holophagae	o__Holophagales	f__Holophagaceae	NSPL	Freshwater	91.70	64.46	2912209	3095	862.44	35	1	8	14	54	28.5	6.13	Anaerobic	Scarcity
GCA_903927475	c__Holophagae	o__Holophagales	f__Holophagaceae	NSPL	Freshwater	91.45	68.22	3212168	3238	906.82	36	2	4	8	53	28.61	5.90	Anaerobic	Scarcity
GCA_903927605	c__Holophagae	o__Holophagales	f__Holophagaceae	NSPL	Freshwater	90.38	63.46	3800741	3663	936.66	37	6	8	9	44	30.8	5.98	Anaerobic	Scarcity
GCA_903928735	c__Holophagae	o__Holophagales	f__Holophagaceae	NSPL	Freshwater	91.83	66.02	3359973	3057	1007.30	44	1	12	12	55	28.1	6.37	Anaerobic	Competitor
GCA_903937385	c__Holophagae	o__Holophagales	f__Holophagaceae	NSPL	Freshwater	91.05	66.97	3675806	3181	1051.18	40	2	8	11	48	27.32	5.79	Anaerobic	Competitor
GCA_903939925	c__Holophagae	o__Holophagales	f__Holophagaceae	NSPL	Freshwater	91.16	65.23	3914665	3835	931.37	49	3	8	12	71	28.24	6.00	Anaerobic	Scarcity
GCA_903940725	c__Holophagae	o__Holophagales	f__Holophagaceae	NSPL	Freshwater	91.24	67.03	3641853	3152	1053.76	38	2	8	10	48	27.3	5.79	Anaerobic	Competitor
GCA_903953415	c__Holophagae	o__Holophagales	f__Holophagaceae	NSPL	Freshwater	90.80	65.04	3848983	3918	891.20	48	3	16	14	73	28.67	5.88	Anaerobic	Scarcity
GCA_903956775	c__Holophagae	o__Holophagales	f__Holophagaceae	NSPL	Freshwater	90.69	66.72	3844646	3716	936.61	38	2	12	10	54	28.25	6.19	Anaerobic	Scarcity
GCA_945907205	c__Holophagae	o__Holophagales	f__Holophagaceae	NSPL	Freshwater	94.98	46.74	1153646	1122	977.40	12	0	0	5	16	24.52	6.69	Aerobic	Scarcity
GCA_000014005	c__Terriglobia	o__Terriglobales	f__Koribacteraceae	SPL	Soil	90.15	58.38	5650368	4998	1020.38	41	2	24	85	87	26.88	5.27	Aerobic	Competitor
GCA_000022565	c__Terriglobia	o__Terriglobales	f__Acidobacteriaceae	SPL	Engineered	88.57	60.50	4127356	3370	1085.63	33	2	47	96	52	29.14	5.05	Aerobic	Competitor
GCA_000178955	c__Terriglobia	o__Terriglobales	f__Acidobacteriaceae	SPL	Soil	87.79	57.91	6237577	4906	1117.48	37	2	73	189	87	21.06	5.11	Aerobic	Competitor
GCA_000178975	c__Terriglobia	o__Terriglobales	f__Acidobacteriaceae	SPL	Engineered	88.09	59.98	5503984	4718	1029.02	36	4	24	123	68	20.79	4.91	Aerobic	Competitor
GCA_000179915	c__Terriglobia	o__Terriglobales	f__Acidobacteriaceae	SPL	Soil	90.46	57.34	5095226	4274	1079.81	34	2	50	133	75	21.16	4.79	Aerobic	Competitor
GCA_000265425	c__Terriglobia	o__Terriglobales	f__Acidobacteriaceae	SPL	Soil	89.08	60.28	5227858	4353	1070.99	32	1	28	113	72	23.92	4.32	Aerobic	Competitor
GCA_000381585	c__Terriglobia	o__Terriglobales	f__Acidobacteriaceae	SPL	Soil	87.31	59.15	6250814	5439	1006.81	50	4	42	142	78	24.29	5.08	Aerobic	Competitor
GCA_000381605	c__Terriglobia	o__Terriglobales	f__Acidobacteriaceae	SPL	Soil	87.98	57.55	6009926	5060	1045.94	49	3	40	152	86	21.68	5.38	Aerobic	Competitor
GCA_000381625	c__Terriglobia	o__Bryobacterales	f__Bryobacteraceae	SPL	Soil	89.55	57.17	6692160	5780	1039.90	63	4	28	155	51	27.85	6.18	Aerobic	Competitor
GCA_000421065	c__Terriglobia	o__Terriglobales	f__Acidobacteriaceae	SPL	Freshwater	86.83	58.83	6136933	5276	1011.67	50	4	79	123	79	24.28	5.24	Aerobic	Competitor
GCA_000688455	c__Terriglobia	o__Terriglobales	f__Acidobacteriaceae	SPL	Marine	91.73	56.49	3686523	3169	1068.49	29	2	30	109	47	32.43	4.67	Aerobic	Competitor
GCA_000688615	c__Terriglobia	o__Terriglobales	f__Acidobacteriaceae	SPL	Soil	89.23	56.74	5001037	4170	1071.42	49	1	32	141	65	24.7	5.76	Aerobic	Competitor
GCA_000702445	c__Terriglobia	o__Bryobacterales	f__Bryobacteraceae	SPL	Soil	93.24	57.95	5747993	5068	1060.90	44	5	8	54	61	25.91	5.98	Aerobic	Ruderal
GCA_000745965	c__Terriglobia	o__Terriglobales	f__Acidobacteriaceae	SPL	Soil	83.86	58.74	8177135	7678	895.27	76	0	67	165	90	23.45	4.65	Aerobic	Competitor
GCA_000800015	c__Terriglobia	o__Terriglobales	f__Acidobacteriaceae	SPL	Soil	91.19	56.69	4947033	4223	1069.45	32	4	34	119	70	23.31	4.55	Aerobic	Competitor
GCA_001006305	c__Terriglobia	o__Terriglobales	f__Acidobacteriaceae	SPL	Soil	87.99	58.23	6459122	5346	1064.73	37	3	78	148	95	23.98	4.61	Aerobic	Competitor
GCA_001449115	c__Terriglobia	o__Terriglobales	f__Acidobacteriaceae	SPL	Soil	89.16	57.30	5345702	4440	1074.72	37	1	65	148	79	22.72	4.70	Aerobic	Competitor
GCA_001464575	c__Terriglobia	o__Bryobacterales	f__Bryobacteraceae	SPL	Engineered	94.00	62.70	6454507	6012	1012.13	45	0	50	61	77	25.34	6.04	Aerobic	Scarcity
GCA_001766965	c__Terriglobia	o__Acidoferrales	f__2_02_FULL_67_57	SPL	Freshwater	90.16	67.31	1641117	1714	873.25	12	1	4	5	21	47.96	6.07	Anaerobic	Scarcity
GCA_001766975	c__Terriglobia	o__Acidoferrales	f__2_02_FULL_67_57	SPL	Freshwater	91.26	67.47	1496642	1671	820.80	12	2	12	4	27	48.8	6.36	Anaerobic	Scarcity
GCA_001766985	c__Terriglobia	o__Acidoferrales	f__2_02_FULL_67_57	SPL	Freshwater	90.71	67.33	1346034	1407	874.14	9	1	8	4	12	48.26	6.28	Anaerobic	Scarcity
GCA_001767035	c__Terriglobia	o__2_12_FULL_54_10	f__2_12_FULL_54_10	SPL	Freshwater	85.88	60.88	3864371	3604	923.72	27	2	16	12	31	33.89	5.54	Aerobic	Scarcity
GCA_001767075	c__Terriglobia	o__2_12_FULL_54_10	f__2_12_FULL_54_10	SPL	Freshwater	87.74	53.90	2949164	2800	936.71	31	0	20	13	21	27.95	6.16	Aerobic	Scarcity
GCA_001897435	c__Terriglobia	o__Terriglobales	f__Acidobacteriaceae	SPL	Engineered	88.76	58.90	4923487	4150	1053.85	45	6	24	135	46	24.74	4.98	Aerobic	Competitor
GCA_001914735	c__Terriglobia	o__Acidoferrales	f__UBA7541	SPL	Soil	85.32	58.05	7412957	6580	963.18	58	1	40	97	73	25.68	4.99	Aerobic	Competitor
GCA_001914775	c__Terriglobia	o__Acidoferrales	f__UBA7541	SPL	Soil	87.44	57.48	2818596	2740	912.31	23	0	12	45	44	26.13	5.94	Aerobic	Competitor
GCA_001914785	c__Terriglobia	o__Acidoferrales	f__UBA7541	SPL	Soil	88.70	57.08	4577825	4132	984.00	33	1	46	53	44	26.38	5.37	Aerobic	Competitor
GCA_001914815	c__Terriglobia	o__Terriglobales	f__SbA1	SPL	Soil	88.49	55.37	2774858	2792	885.40	27	0	20	42	51	30.71	5.28	Aerobic	Scarcity
GCA_001917435	c__Terriglobia	o__Acidoferrales	f__UBA7541	SPL	Soil	88.69	57.39	4455545	4057	975.06	37	2	36	71	49	27.22	5.95	Aerobic	Competitor
GCA_001917495	c__Terriglobia	o__Terriglobales	f__SbA1	SPL	Soil	89.54	55.22	1757384	1812	870.01	26	0	16	17	28	31.29	5.83	Aerobic	Scarcity
GCA_001918935	c__Terriglobia	o__Terriglobales	f__SbA1	SPL	Soil	88.18	55.27	2865787	2919	875.04	28	0	36	29	43	29.82	5.78	Aerobic	Scarcity
GCA_001919455	c__Terriglobia	o__20CM_2_55_15	f__20CM_2_55_15	SPL	Soil	91.37	55.80	3181833	3066	952.93	26	0	20	14	34	31.7	5.83	Aerobic	Scarcity
GCA_001919715	c__Terriglobia	o__Acidoferrales	f__UBA7541	SPL	Soil	86.79	57.92	3861114	3457	971.63	33	0	24	56	39	23.93	5.60	Aerobic	Scarcity
GCA_001920875	c__Terriglobia	o__20CM_2_55_15	f__20CM_2_55_15	SPL	Soil	91.61	55.55	3444861	3368	941.38	26	1	12	14	41	31.91	5.61	Aerobic	Scarcity
GCA_002197525	c__Terriglobia	o__UBA7540	f__UBA7540	SPL	Engineered	84.04	58.52	2798462	2432	965.83	15	1	16	16	25	35.55	5.58	Aerobic	Ruderal
GCA_002197545	c__Terriglobia	o__Terriglobales	f__Acidobacteriaceae	SPL	Engineered	88.56	57.64	4885153	5058	853.29	41	4	24	99	67	25.19	4.88	Aerobic	Competitor
GCA_002298225	c__Terriglobia	o__Bryobacterales	f__Bryobacteraceae	SPL	Engineered	92.70	62.40	6737936	5778	1084.00	56	1	24	195	67	30.83	5.85	Aerobic	Competitor
GCA_002307235	c__Terriglobia	o__Terriglobales	f__Acidobacteriaceae	SPL	Soil	86.25	60.99	3309334	2722	1069.42	22	2	4	90	41	23.78	5.14	Anaerobic	Competitor
GCA_002314435	c__Terriglobia	o__Terriglobales	f__Acidobacteriaceae	SPL	Soil	84.50	61.56	3946909	3615	983.21	18	0	8	119	61	31.37	5.05	Aerobic	Competitor
GCA_002403945	c__Terriglobia	o__Terriglobales	f__Acidobacteriaceae	SPL	Soil	83.58	58.28	3121854	2980	930.44	18	4	20	89	32	28.63	6.13	Aerobic	Competitor
GCA_002413385	c__Terriglobia	o__Terriglobales	f__Acidobacteriaceae	SPL	Soil	85.39	58.89	3584921	3671	846.77	18	2	12	115	52	22.89	5.46	Aerobic	Scarcity
GCA_002433055	c__Terriglobia	o__Bryobacterales	f__UBA6623	SPL	Marine	89.66	47.83	1812321	1568	1043.42	12	1	24	21	16	23.53	6.09	Aerobic	Scarcity
GCA_002478115	c__Terriglobia	o__Acidoferrales	f__UBA7541	SPL	Soil	84.81	57.99	5512597	4890	961.84	39	1	56	96	53	28.52	5.21	Aerobic	Competitor
GCA_002478145	c__Terriglobia	o__UBA7540	f__UBA7540	SPL	Soil	82.22	58.09	6337545	5688	928.87	62	2	21	134	57	31.96	5.42	Anaerobic	Scarcity
GCA_002716325	c__Terriglobia	o__Bryobacterales	f__UBA6623	SPL	Marine	90.32	47.60	1999383	1690	1068.89	14	0	16	29	18	24.42	6.09	Aerobic	Scarcity
GCA_003010405	c__Terriglobia	o__Terriglobales	f__Koribacteraceae	SPL	Engineered	87.23	55.46	3640359	3462	918.51	33	3	20	21	38	31.66	5.23	Anaerobic	Ruderal
GCA_003131205	c__Terriglobia	o__Terriglobales	f__Acidobacteriaceae	SPL	Soil	87.26	59.10	6278575	5373	1020.73	47	2	16	137	58	24.77	4.81	Aerobic	Competitor
GCA_003131305	c__Terriglobia	o__Terriglobales	f__SbA1	SPL	Soil	85.83	58.21	5904282	5498	925.03	30	6	75	74	77	28	4.80	Aerobic	Competitor
GCA_003131315	c__Terriglobia	o__Terriglobales	f__SbA1	SPL	Soil	85.90	58.25	6004649	5372	959.20	33	5	77	74	75	28.33	4.80	Aerobic	Competitor
GCA_003131545	c__Terriglobia	o__Terriglobales	f__SbA1	SPL	Soil	84.22	58.62	4608064	4623	845.75	29	1	32	49	67	28.87	4.88	Anaerobic	Competitor
GCA_003131585	c__Terriglobia	o__Terriglobales	f__SbA1	SPL	Soil	86.12	59.24	3934625	3670	925.34	26	3	32	25	44	28.91	5.38	Aerobic	Scarcity
GCA_003131625	c__Terriglobia	o__Terriglobales	f__Acidobacteriaceae	SPL	Soil	87.99	61.85	5394508	4842	981.56	44	3	24	107	73	26.39	5.05	Aerobic	Competitor
GCA_003131635	c__Terriglobia	o__Terriglobales	f__SbA1	SPL	Soil	86.69	57.30	5285449	5117	902.46	29	3	66	59	66	26.14	5.26	Aerobic	Scarcity
GCA_003131705	c__Terriglobia	o__Acidoferrales	f__UBA7541	SPL	Soil	85.74	57.65	3940173	4344	779.87	30	4	36	21	40	25.85	5.76	Aerobic	Scarcity
GCA_003131985	c__Terriglobia	o__Acidoferrales	f__UBA7541	SPL	Soil	87.62	56.30	3948416	3997	877.31	34	1	45	24	58	22.56	5.56	Aerobic	Scarcity
GCA_003132145	c__Terriglobia	o__Terriglobales	f__Acidobacteriaceae	SPL	Soil	86.27	61.79	3165847	2756	992.33	16	0	16	68	37	25.57	5.40	Anaerobic	Competitor
GCA_003132325	c__Terriglobia	o__Terriglobales	f__SbA1	SPL	Soil	83.92	58.58	5317643	5266	850.57	33	4	29	47	68	28.61	4.67	Anaerobic	Competitor
GCA_003132365	c__Terriglobia	o__Terriglobales	f__Acidobacteriaceae	SPL	Soil	84.74	57.71	6726311	6525	872.47	47	2	52	232	69	25.05	5.29	Aerobic	Competitor
GCA_003132375	c__Terriglobia	o__Terriglobales	f__SbA1	SPL	Soil	86.33	56.69	5697891	5502	895.79	41	2	24	90	85	27.02	4.93	Aerobic	Scarcity
GCA_003132405	c__Terriglobia	o__Terriglobales	f__SbA1	SPL	Soil	86.18	56.60	5724666	5312	930.33	46	2	20	89	79	26.89	4.90	Aerobic	Competitor
GCA_003132445	c__Terriglobia	o__Terriglobales	f__SbA1	SPL	Soil	86.29	56.23	5123786	4767	928.03	40	0	20	86	64	28.04	4.85	Aerobic	Scarcity
GCA_003132465	c__Terriglobia	o__Acidoferrales	f__UBA7541	SPL	Soil	87.44	57.55	4252346	4295	867.07	45	1	23	39	64	25.71	5.24	Aerobic	Scarcity
GCA_003132485	c__Terriglobia	o__Acidoferrales	f__UBA7541	SPL	Soil	87.04	59.67	4171751	4363	834.51	34	1	40	32	75	25.05	5.41	Aerobic	Scarcity
GCA_003132545	c__Terriglobia	o__Acidoferrales	f__UBA7541	SPL	Soil	87.27	60.16	3268539	3543	807.27	25	1	32	17	67	25.37	5.50	Aerobic	Scarcity
GCA_003132745	c__Terriglobia	o__Terriglobales	f__Koribacteraceae	SPL	Soil	85.76	58.71	4463622	4148	922.37	33	3	20	23	65	32.36	5.59	Aerobic	Scarcity
GCA_003132825	c__Terriglobia	o__Terriglobales	f__SbA1	SPL	Soil	85.19	59.88	4618113	4634	853.81	38	0	28	98	68	24.75	5.06	Aerobic	Competitor

GCA_003132845	c__Terriglobia	o__Terriglobales	f__SbA1	SPL	Soil	86.78	56.90	4958720	4679	920.94	31	0	36	79	82	27.22	4.99	Aerobic	Competitor
GCA_003132865	c__Terriglobia	o__Bryobacterales	f__Bryobacteraceae	SPL	Soil	85.81	61.61	9717417	10044	831.80	76	5	45	150	105	28.21	5.35	Aerobic	Scarcity
GCA_003132885	c__Terriglobia	o__Acidoferrales	f__UBA7541	SPL	Soil	89.49	56.55	3495868	3172	987.64	38	2	36	19	41	26.83	5.40	Aerobic	Competitor
GCA_003132905	c__Terriglobia	o__Bryobacterales	f__Bryobacteraceae	SPL	Soil	89.38	57.68	6508434	6957	839.55	47	5	28	43	85	25.02	5.35	Aerobic	Scarcity
GCA_003132965	c__Terriglobia	o__Acidoferrales	f__UBA7541	SPL	Soil	87.59	59.86	3906114	3784	906.88	32	1	36	24	60	24.67	5.50	Aerobic	Scarcity
GCA_003132985	c__Terriglobia	o__Terriglobales	f__Koribacteraceae	SPL	Soil	85.84	58.69	4530645	4172	931.87	33	2	16	24	60	32.18	5.59	Anaerobic	Scarcity
GCA_003133145	c__Terriglobia	o__Terriglobales	f__SbA1	SPL	Soil	87.46	58.86	4239900	3976	936.08	22	0	40	42	64	27.37	5.00	Aerobic	Competitor
GCA_003133205	c__Terriglobia	o__Terriglobales	f__Acidobacteriaceae	SPL	Soil	86.91	60.10	6149698	5628	950.88	35	5	44	246	63	22.66	4.73	Aerobic	Competitor
GCA_003133425	c__Terriglobia	o__Terriglobales	f__Acidobacteriaceae	SPL	Soil	84.95	58.13	6466358	5774	951.59	42	5	28	214	69	25.93	4.22	Aerobic	Ruderal
GCA_003133445	c__Terriglobia	o__Terriglobales	f__Koribacteraceae	SPL	Soil	85.73	58.69	4427505	4122	920.50	32	1	16	24	62	31.9	5.76	Aerobic	Competitor
GCA_003133465	c__Terriglobia	o__Bryobacterales	f__Bryobacteraceae	SPL	Soil	89.98	58.36	7510429	7697	881.35	66	2	80	108	109	26.71	4.96	Aerobic	Ruderal
GCA_003133505	c__Terriglobia	o__Acidoferrales	f__UBA7541	SPL	Soil	86.19	56.78	3222601	3606	772.54	23	2	45	16	34	22.56	5.82	Aerobic	Scarcity
GCA_003133525	c__Terriglobia	o__Acidoferrales	f__UBA7541	SPL	Soil	89.53	56.49	3695824	3288	1007.85	40	1	24	22	40	27.02	5.40	Aerobic	Competitor
GCA_003133595	c__Terriglobia	o__Terriglobales	f__SbA1	SPL	Soil	86.56	58.60	4888831	4533	936.46	26	2	77	61	67	26.73	4.97	Aerobic	Scarcity
GCA_003134095	c__Terriglobia	o__Terriglobales	f__SbA1	SPL	Soil	87.47	58.99	3319618	3134	930.57	16	0	24	30	54	26.83	5.43	Aerobic	Competitor
GCA_003134295	c__Terriglobia	o__Terriglobales	f__Acidobacteriaceae	SPL	Soil	85.72	59.82	4581855	4572	862.48	29	0	20	122	60	24.74	5.21	Aerobic	Competitor
GCA_003134355	c__Terriglobia	o__Terriglobales	f__Acidobacteriaceae	SPL	Soil	86.71	61.60	4779977	4494	923.48	30	4	20	151	51	23.7	5.15	Aerobic	Scarcity
GCA_003134435	c__Terriglobia	o__Terriglobales	f__SbA1	SPL	Soil	88.40	58.13	5749602	5112	995.29	44	1	52	97	69	26.84	4.92	Aerobic	Competitor
GCA_003134575	c__Terriglobia	o__Bryobacterales	f__Bryobacteraceae	SPL	Soil	89.81	58.53	6982827	7179	875.99	47	2	28	63	90	25.77	5.75	Aerobic	Competitor
GCA_003134655	c__Terriglobia	o__Terriglobales	f__SbA1	SPL	Soil	86.40	58.05	8045290	7189	967.01	76	8	66	148	79	24.86	5.15	Aerobic	Competitor
GCA_003134815	c__Terriglobia	o__Terriglobales	f__Acidobacteriaceae	SPL	Soil	87.15	59.13	4179761	3634	1003.56	23	1	20	71	42	24.15	5.11	Aerobic	Competitor
GCA_003134855	c__Terriglobia	o__Terriglobales	f__Acidobacteriaceae	SPL	Soil	86.72	59.94	3940365	3334	1025.62	21	1	8	88	38	27.17	5.02	Anaerobic	Competitor
GCA_003134895	c__Terriglobia	o__Terriglobales	f__SbA1	SPL	Soil	86.48	58.05	8018070	7274	955.33	72	6	54	147	81	24.79	5.15	Aerobic	Competitor
GCA_003134995	c__Terriglobia	o__Terriglobales	f__SbA1	SPL	Soil	87.24	56.83	5620309	5078	966.84	32	1	36	62	65	25.64	4.72	Aerobic	Competitor
GCA_003135015	c__Terriglobia	o__Bryobacterales	f__Bryobacteraceae	SPL	Soil	90.31	58.32	8097738	7640	957.39	74	2	70	115	97	26.91	4.65	Aerobic	Competitor
GCA_003135055	c__Terriglobia	o__Bryobacterales	f__Bryobacteraceae	SPL	Soil	84.60	60.94	10710138	9849	922.07	85	2	28	139	94	24.46	5.04	Aerobic	Ruderal
GCA_003135085	c__Terriglobia	o__Acidoferrales	f__UBA7541	SPL	Soil	89.16	56.47	4072028	3667	990.76	42	2	29	28	44	26.58	5.38	Aerobic	Competitor
GCA_003135175	c__Terriglobia	o__Bryobacterales	f__Bryobacteraceae	SPL	Soil	87.75	62.45	5265980	5070	915.40	37	5	8	129	66	29.1	5.22	Anaerobic	Competitor
GCA_003135275	c__Terriglobia	o__Terriglobales	f__SbA1	SPL	Soil	87.29	58.88	4094525	3870	926.24	24	0	36	41	61	27.08	5.00	Aerobic	Scarcity
GCA_003135315	c__Terriglobia	o__Terriglobales	f__Koribacteraceae	SPL	Soil	85.48	59.27	4365399	4488	832.91	27	1	24	21	61	30.69	5.07	Aerobic	Scarcity
GCA_003135475	c__Terriglobia	o__Terriglobales	f__Koribacteraceae	SPL	Soil	85.14	58.65	3894972	3873	859.09	21	5	20	18	55	32.29	4.97	Aerobic	Scarcity
GCA_003135495	c__Terriglobia	o__Terriglobales	f__SbA1	SPL	Soil	85.81	55.03	4758665	5050	811.26	32	5	32	67	71	25.74	4.93	Aerobic	Scarcity
GCA_003135815	c__Terriglobia	o__Terriglobales	f__Koribacteraceae	SPL	Soil	82.83	58.15	4844851	4813	835.26	39	2	20	28	56	31.94	5.76	Aerobic	Competitor
GCA_003135875	c__Terriglobia	o__Terriglobales	f__Acidobacteriaceae	SPL	Soil	86.45	57.46	6035365	5737	907.93	39	5	28	229	55	23.84	4.55	Aerobic	Competitor
GCA_003135955	c__Terriglobia	o__Terriglobales	f__Acidobacteriaceae	SPL	Soil	85.05	60.25	6408911	6069	897.87	57	3	8	177	71	25.6	5.10	Aerobic	Competitor
GCA_003136035	c__Terriglobia	o__Bryobacterales	f__Bryobacteraceae	SPL	Soil	91.91	56.18	6318816	6082	958.12	49	4	32	121	89	22.79	5.56	Aerobic	Scarcity
GCA_003136055	c__Terriglobia	o__Terriglobales	f__SbA1	SPL	Soil	85.03	58.84	4662823	4649	853.62	31	3	28	27	51	26.72	5.36	Aerobic	Scarcity
GCA_003136115	c__Terriglobia	o__Terriglobales	f__SbA1	SPL	Soil	86.74	56.84	5268641	5206	878.91	33	2	40	62	66	24.85	4.68	Aerobic	Competitor
GCA_003136215	c__Terriglobia	o__Bryobacterales	f__Bryobacteraceae	SPL	Soil	91.03	62.85	7095248	6669	970.55	52	6	28	177	88	28.22	5.66	Anaerobic	Scarcity
GCA_003136335	c__Terriglobia	o__Terriglobales	f__SbA1	SPL	Soil	85.38	57.83	5067794	5222	829.75	48	0	32	83	66	26.33	5.81	Aerobic	Competitor
GCA_003136375	c__Terriglobia	o__Terriglobales	f__Acidobacteriaceae	SPL	Soil	86.38	59.62	5547843	4931	972.51	34	0	16	193	64	23.02	4.89	Aerobic	Competitor
GCA_003136575	c__Terriglobia	o__Bryobacterales	f__Bryobacteraceae	SPL	Soil	87.53	60.07	6596786	6345	912.09	59	1	36	59	61	22.78	5.48	Aerobic	Scarcity
GCA_003136675	c__Terriglobia	o__Bryobacterales	f__Bryobacteraceae	SPL	Soil	92.21	59.46	6102160	5810	970.59	52	2	28	102	70	23.48	5.47	Aerobic	Competitor
GCA_003136775	c__Terriglobia	o__Terriglobales	f__Acidobacteriaceae	SPL	Soil	85.70	59.83	4630185	4521	882.06	30	2	20	119	57	24.68	5.06	Aerobic	Competitor
GCA_003136815	c__Terriglobia	o__Terriglobales	f__Acidobacteriaceae	SPL	Soil	85.18	58.06	5760949	5550	881.93	47	2	24	210	68	24.55	4.58	Anaerobic	Scarcity
GCA_003136835	c__Terriglobia	o__Terriglobales	f__SbA1	SPL	Soil	85.04	58.80	4472401	4486	848.70	24	1	37	36	66	29.6	4.94	Anaerobic	Scarcity
GCA_003136855	c__Terriglobia	o__Terriglobales	f__SbA1	SPL	Soil	87.28	56.83	5607601	5095	962.13	33	1	36	62	66	25.7	4.72	Aerobic	Competitor
GCA_003137035	c__Terriglobia	o__Bryobacterales	f__Bryobacteraceae	SPL	Soil	90.53	64.59	5068287	5043	912.40	38	3	36	135	61	34.29	5.53	Anaerobic	Scarcity
GCA_003137155	c__Terriglobia	o__Bryobacterales	f__Bryobacteraceae	SPL	Soil	87.08	62.42	4816775	4965	853.23	32	1	4	116	60	29.24	5.42	Aerobic	Competitor
GCA_003138115	c__Terriglobia	o__Terriglobales	f__Acidobacteriaceae	SPL	Soil	86.42	61.51	4996975	4649	929.58	30	4	20	155	50	23.99	5.22	Aerobic	Scarcity
GCA_003138175	c__Terriglobia	o__Terriglobales	f__Acidobacteriaceae	SPL	Soil	86.93	59.96	2990647	3038	856.97	23	1	20	51	37	27.32	5.40	Anaerobic	Competitor
GCA_003138215	c__Terriglobia	o__Terriglobales	f__Acidobacteriaceae	SPL	Soil	85.32	61.15	5283229	4969	906.89	47	1	12	154	72	25.49	5.31	Aerobic	Competitor
GCA_003138365	c__Terriglobia	o__Terriglobales	f__Acidobacteriaceae	SPL	Soil	86.10	59.80	4780506	4441	927.69	30	1	20	124	55	25.01	5.23	Anaerobic	Competitor
GCA_003138435	c__Terriglobia	o__Terriglobales	f__SbA1	SPL	Soil	86.45	58.85	5505352	4790	994.28	32	3	38	32	56	27.28	5.01	Aerobic	Competitor
GCA_003138455	c__Terriglobia	o__Bryobacterales	f__Bryobacteraceae	SPL	Soil	87.72	62.46	5407871	5100	934.72	35	0	12	128	63	29.41	5.24	Anaerobic	Competitor
GCA_003138475	c__Terriglobia	o__Bryobacterales	f__Bryobacteraceae	SPL	Soil	88.39	62.56	7690263	7346	929.35	50	1	28	189	94	28.22	5.78	Anaerobic	Scarcity
GCA_003138515	c__Terriglobia	o__Terriglobales	f__Acidobacteriaceae	SPL	Soil	86.68	60.16	4234646	3731	983.80	27	0	24	77	49	25.87	5.24	Aerobic	Competitor
GCA_003138555	c__Terriglobia	o__Terriglobales	f__SbA1	SPL	Soil	85.60	58.12	5473310	4870	961.85	48	0	20	96	66	30.95	4.83	Anaerobic	Ruderal
GCA_003138695	c__Terriglobia	o__Terriglobales	f__Acidobacteriaceae	SPL	Soil	82.44	61.82	2594363	2543	844.21	15	1	20	18	26	26.34	5.24	Anaerobic	Competitor
GCA_003138715	c__Terriglobia	o__Terriglobales	f__Acidobacteriaceae	SPL	Soil	87.39	60.28	2762120	2571	941.60	20	2	20	28	60	25.52	5.15	Aerobic	Competitor
GCA_003138735	c__Terriglobia	o__Terriglobales	f__Acidobacteriaceae	SPL	Soil	86.04	61.56	5056452	4695	928.72	24	2	20	154	52	23.11	5.88	Aerobic	Scarcity
GCA_003138795	c__Terriglobia	o__Terriglobales	f__Acidobacteriaceae	SPL	Soil	87.07	60.41	3137262	2985	918.44	22	2	20	30	66	25.3	4.99	Aerobic	Competitor
GCA_003138955	c__Terriglobia	o__Terriglobales	f__SbA1	SPL	Soil	84.97	58.58	4622803	4574	862.81	35	2	66	28	64	30.38	4.51	Anaerobic	Scarcity
GCA_003138975	c__Terriglobia	o__Terriglobales	f__SbA1	SPL	Soil	86.11	57.15	5453551	5267	893.88	43	2	44	72	71	29.04	4.83	Aerobic	Competitor
GCA_003138995	c__Terriglobia	o__Bryobacterales	f__Bryobacteraceae	SPL	Soil	86.68	62.42	4640461	4820	846.99	33	2	8	113	77	28.81	5.23	Anaerobic	Scarcity
GCA_003139095	c__Terriglobia	o__Terriglobales	f__SbA1	SPL	Soil	86.37	58.86	5531701	4826	990.34	33	2	38	33	58	27.1	4.80	Aerobic	Competitor
GCA_003139165	c__Terriglobia	o__Bryobacterales	f__Bryobacteraceae	SPL	Soil	88.90	62.59	7482670	7141	934.39	51	2	32	191	95	28.11	5.44	Aerobic	Scarcity
GCA_003139195	c__Terriglobia	o__Terriglobales	f__Acidobacteriaceae	SPL	Soil	82.36	60.88	3466778	3299	866.68	27	7	20	22	37	27.82	5.02	Anaerobic	Competitor
GCA_003139335	c__Terriglobia	o__Terriglobales	f__Acidobacteriaceae	SPL	Soil	86.44	61.35	2651444	2372	969.14	19	2	18	43	30	27.06	5.32	Anaerobic	Competitor
GCA_003139375	c__Terriglobia	o__Terriglobales	f__SbA1	SPL	Soil	87.04	57.67	3956048	4113	839.63	33	1	28	33	61	25.14	5.13	Aerobic	Scarcity
GCA_003139435	c__Terriglobia	o__Terriglobales	f__SbA1	SPL	Soil	86.92	56.86	4825677	4970	846.37	28	1	36	55	60	24.95	5.30	Aerobic	Scarcity
GCA_003139495	c__Terriglobia	o__Terriglobales	f__SbA1	SPL	Soil	86.48	56.59	5100493	5372	821.73	41	1	28	83	77	26.46	5.01	Aerobic	Scarcity
GCA_003139635	c__Terriglobia	o__Acidoferrales	f__UBA7541	SPL	Soil	89.83	56.56	3646543	3297	995.37	38	2	36	22	43	27.28	5.38	Aerobic	Competitor
GCA_003139735	c__Terriglobia	o__Bryobacterales	f__Bryobacteraceae	SPL	Soil	88.79	63.18	5281686	5518	853.03	33	7	16	178	64	28.7	5.51	Anaerobic	Scarcity
GCA_003139755	c__Terriglobia	o__Terriglobales	f__Acidobacteriaceae	SPL	Soil	83.30	61.99	2827421	2638	894.84	18	2	16	18	33	26.29	5.24	Anaerobic	Competitor
GCA_003139795	c__Terriglobia	o__Terriglobales	f__Acidobacteriaceae	SPL	Soil	85.29	60.50	6342538	5660	955.93	50	2	8	180	63	25.48	5.26	Aerobic	Competitor
GCA_003139815	c__Terriglobia	o__Terriglobales	f__Acidobacteriaceae	SPL	Soil	85.84	59.81	4694399	4420	914.49	31	1	20	125	55	25.26	5.23	Anaerobic	Competitor
GCA_003139875	c__Terriglobia	o__Terriglobales	f__Acidobacteriaceae	SPL	Soil	86.15	59.81	4725122	4431	920.62	31	1	21	121	53	25.42	5.23	Anaerobic	Competitor
GCA_003139975	c__Terriglobia	o__Terriglobales	f__SbA1	SPL	Soil	86.34	58.47	3907728	4188	806.51	26	2	48	31	55	27.41	5.19	Aerobic	Scarcity
GCA_003139995	c__Terriglobia	o__Terriglobales	f__SbA1	SPL	Soil	85.50	58.11	5493220	4986	942.66	47	1	16	100	68	30.8	4.83	Anaerobic	Competitor
GCA_003140035	c__Terriglobia	o__Terriglobales	f__SbA1	SPL	Soil	85.63	58.09	5499470	4947	952.31	47	1	16	101	67	30.85	5.03	Anaerobic	Competitor
GCA_003140075	c__Terriglobia	o__Terriglobales	f__SbA1	SPL	Soil	86.71	57.95	5768439	5464	916.25	43	2	80	59	65	26.34	4.93	Aerobic	Scarcity
GCA_003140155	c__Terriglobia	o__Terriglobales	f__Acidobacteriaceae	SPL	Soil	87.58	62.19	4141534	3973	918.56	31	0	16	82	74	26.75	4.93	Aerobic	Scarcity
GCA_003140235	c__Terriglobia	o__Terriglobales	f__Gp1_AA117	SPL	Soil	88.38	56.59	4904602	4708	922.96	42	2	16	26	65	27.05	5.61	Aerobic	Scarcity
GCA_003140295	c__Terriglobia	o__Acidoferrales	f__UBA7541	SPL	Soil	84.50	58.98	4101052	4140	841.08	21	4	24	16	37	27.88	6.10	Aerobic	Scarcity
GCA_003140355	c__Terriglobia	o__Terriglobales	f__SbA1	SPL	Soil	85.89	59.24	3481599	3297	906.15	24	4	32	21	41	28.86	5.40	Aerobic	Scarcity
GCA_003140575	c__Terriglobia	o__Terriglobales	f__Acidobacteriaceae	SPL	Soil	85.90	59.86	4134954	4165	857.14	29	1	29	111	64	24.41	5.64	Anaerobic	Competitor

GCA_003140635	c__Terriglobia	o__Terriglobales	f__Acidobacteriaceae	SPL	Soil	87.39	62.23	4106068	4025	895.53	29	0	20	76	81	25.89	4.71	Aerobic	Scarcity
GCA_003140655	c__Terriglobia	o__Terriglobales	f__SbA1	SPL	Soil	83.45	57.98	4663284	5393	735.73	29	1	32	52	71	25.35	5.55	Aerobic	Scarcity
GCA_003140695	c__Terriglobia	o__Terriglobales	f__SbA1	SPL	Soil	86.35	57.76	5222111	5012	904.71	40	1	36	60	67	26.88	5.27	Aerobic	Competitor
GCA_003140705	c__Terriglobia	o__Terriglobales	f__Acidobacteriaceae	SPL	Soil	85.45	57.85	5241902	5409	830.13	37	0	40	153	63	23.69	4.46	Aerobic	Scarcity
GCA_003140725	c__Terriglobia	o__Terriglobales	f__SbA1	SPL	Soil	86.17	58.13	7405126	7050	907.20	62	1	52	133	92	24.98	5.47	Aerobic	Competitor
GCA_003140755	c__Terriglobia	o__Terriglobales	f__SbA1	SPL	Soil	86.57	56.62	6047625	5588	938.28	46	2	32	94	82	27	4.90	Aerobic	Competitor
GCA_003140775	c__Terriglobia	o__Terriglobales	f__Acidobacteriaceae	SPL	Soil	86.79	61.66	5430155	4793	982.95	41	1	24	121	76	24.93	5.14	Aerobic	Competitor
GCA_003140785	c__Terriglobia	o__Terriglobales	f__Acidobacteriaceae	SPL	Soil	86.30	57.95	5414947	5367	872.58	37	2	32	120	62	24.17	5.32	Aerobic	Competitor
GCA_003140875	c__Terriglobia	o__Terriglobales	f__Acidobacteriaceae	SPL	Soil	86.68	61.28	3528381	3138	975.82	19	1	22	61	38	27.15	5.17	Anaerobic	Competitor
GCA_003140995	c__Terriglobia	o__Terriglobales	f__SbA1	SPL	Soil	87.30	58.86	4277882	3979	944.75	21	0	40	42	67	27.03	5.00	Aerobic	Competitor
GCA_003141015	c__Terriglobia	o__Bryobacterales	f__Bryobacteraceae	SPL	Soil	87.32	62.68	4288222	4219	895.60	32	0	8	93	62	29.74	5.22	Anaerobic	Scarcity
GCA_003141135	c__Terriglobia	o__Acidoferrales	f__UBA7541	SPL	Soil	87.38	57.62	3743143	4178	784.19	31	2	20	36	58	25.51	5.69	Aerobic	Scarcity
GCA_003141975	c__Terriglobia	o__Bryobacterales	f__Bryobacteraceae	SPL	Soil	85.63	62.17	5093910	5395	824.51	30	1	12	114	63	28.2	4.82	Anaerobic	Scarcity
GCA_003142015	c__Terriglobia	o__Terriglobales	f__Acidobacteriaceae	SPL	Soil	86.90	59.56	3421685	3171	940.74	28	1	16	55	46	26.13	4.97	Anaerobic	Competitor
GCA_003142135	c__Terriglobia	o__Terriglobales	f__Acidobacteriaceae	SPL	Soil	85.37	59.79	4738309	4516	900.77	31	1	28	131	57	25.09	5.24	Anaerobic	Competitor
GCA_003142185	c__Terriglobia	o__Bryobacterales	f__Bryobacteraceae	SPL	Soil	88.41	62.38	5791462	5103	1003.51	37	2	12	137	62	29.41	5.18	Anaerobic	Competitor
GCA_003142215	c__Terriglobia	o__Terriglobales	f__Acidobacteriaceae	SPL	Soil	86.30	61.48	3636854	3431	920.37	20	2	24	89	46	24.47	5.41	Anaerobic	Scarcity
GCA_003142235	c__Terriglobia	o__Terriglobales	f__Acidobacteriaceae	SPL	Soil	85.74	62.63	2806769	2523	956.26	15	1	16	65	36	24.77	5.40	Anaerobic	Competitor
GCA_003142355	c__Terriglobia	o__Bryobacterales	f__Bryobacteraceae	SPL	Soil	89.51	62.99	6556001	6508	904.09	34	6	32	149	75	31.33	5.70	Anaerobic	Competitor
GCA_003142415	c__Terriglobia	o__Acidoferrales	f__UBA7541	SPL	Soil	85.25	58.46	4621151	4131	954.66	31	4	24	66	52	30.32	5.25	Aerobic	Competitor
GCA_003142675	c__Terriglobia	o__Acidoferrales	f__UBA7541	SPL	Soil	85.16	58.43	4481147	4267	895.14	27	3	20	64	52	30.16	5.19	Aerobic	Competitor
GCA_003142885	c__Terriglobia	o__Acidoferrales	f__UBA7541	SPL	Soil	85.19	58.43	4666617	4109	968.44	33	4	28	69	52	30.56	5.25	Aerobic	Competitor
GCA_003142935	c__Terriglobia	o__Terriglobales	f__Acidobacteriaceae	SPL	Soil	85.29	58.75	5195183	4654	952.52	41	1	24	134	51	25.98	5.40	Aerobic	Competitor
GCA_003151155	c__Terriglobia	o__Terriglobales	f__Koribacteraceae	SPL	Soil	87.18	57.06	5709437	5506	905.82	35	3	28	85	77	27.56	5.46	Aerobic	Scarcity
GCA_003151335	c__Terriglobia	o__Terriglobales	f__Koribacteraceae	SPL	Soil	85.00	58.82	4652150	4269	925.84	46	3	12	23	59	32.59	6.18	Aerobic	Scarcity
GCA_003151355	c__Terriglobia	o__Terriglobales	f__SbA1	SPL	Soil	86.33	58.11	6652349	6479	888.06	56	2	50	123	74	24.34	4.91	Aerobic	Competitor
GCA_003151435	c__Terriglobia	o__Terriglobales	f__Acidobacteriaceae	SPL	Soil	85.60	59.10	5960205	5114	997.81	38	5	20	211	62	23.5	4.89	Aerobic	Ruderal
GCA_003151875	c__Terriglobia	o__Terriglobales	f__Koribacteraceae	SPL	Soil	85.76	59.23	3724574	3812	839.85	20	2	12	24	54	32.24	5.70	Aerobic	Scarcity
GCA_003151985	c__Terriglobia	o__Terriglobales	f__Acidobacteriaceae	SPL	Soil	86.26	57.90	6028593	5490	947.69	36	2	16	210	62	23.36	4.50	Anaerobic	Ruderal
GCA_003152195	c__Terriglobia	o__Terriglobales	f__Acidobacteriaceae	SPL	Soil	85.79	58.46	6799527	6581	886.71	42	2	28	148	94	24.69	4.89	Aerobic	Competitor
GCA_003152325	c__Terriglobia	o__Bryobacterales	f__Bryobacteraceae	SPL	Soil	91.14	62.19	5881655	5608	959.78	49	3	24	146	79	26.07	5.45	Aerobic	Competitor
GCA_003152355	c__Terriglobia	o__Acidoferrales	f__UBA7541	SPL	Soil	87.44	57.50	4575937	4357	920.47	45	2	24	47	71	25.04	5.46	Aerobic	Scarcity
GCA_003152365	c__Terriglobia	o__Terriglobales	f__SbA1	SPL	Soil	84.25	58.49	5754238	5700	853.28	42	3	45	55	67	29.38	5.05	Anaerobic	Competitor
GCA_003152415	c__Terriglobia	o__Terriglobales	f__Acidobacteriaceae	SPL	Soil	87.22	62.20	4250281	4073	916.59	30	2	20	85	83	26.89	5.43	Aerobic	Competitor
GCA_003152595	c__Terriglobia	o__Terriglobales	f__Acidobacteriaceae	SPL	Soil	85.97	61.77	5285138	5125	894.01	41	2	20	143	81	28.28	4.95	Aerobic	Competitor
GCA_003152605	c__Terriglobia	o__Terriglobales	f__SbA1	SPL	Soil	85.71	57.65	5073848	5049	876.70	39	3	32	59	69	26.65	5.23	Aerobic	Competitor
GCA_003152695	c__Terriglobia	o__Terriglobales	f__Koribacteraceae	SPL	Soil	85.72	58.67	4504025	4156	928.57	32	3	16	23	65	32.18	5.59	Anaerobic	Scarcity
GCA_003152795	c__Terriglobia	o__Terriglobales	f__SbA1	SPL	Soil	85.93	59.05	4720128	4763	859.91	25	3	36	78	75	26.82	4.94	Anaerobic	Competitor
GCA_003153175	c__Terriglobia	o__Terriglobales	f__SbA1	SPL	Soil	86.45	58.08	6958030	6466	929.80	57	1	70	123	88	24.7	4.98	Aerobic	Competitor
GCA_003153585	c__Terriglobia	o__Acidoferrales	f__UBA7541	SPL	Soil	88.47	56.57	3280613	3401	862.88	33	2	52	20	48	25.52	5.39	Aerobic	Scarcity
GCA_003153615	c__Terriglobia	o__Terriglobales	f__Acidobacteriaceae	SPL	Soil	86.89	60.73	5064768	4907	899.82	42	1	24	92	80	25.2	4.58	Aerobic	Competitor
GCA_003153635	c__Terriglobia	o__Terriglobales	f__SbA1	SPL	Soil	86.43	56.63	6115516	5661	934.68	47	1	32	89	81	26.81	4.90	Aerobic	Competitor
GCA_003153695	c__Terriglobia	o__Terriglobales	f__Acidobacteriaceae	SPL	Soil	86.06	61.25	3376921	3388	861.91	16	3	28	58	39	27.13	5.31	Aerobic	Competitor
GCA_003154055	c__Terriglobia	o__Terriglobales	f__SbA1	SPL	Soil	86.85	59.72	3943854	3933	881.84	31	0	24	61	64	27.59	6.40	Aerobic	Scarcity
GCA_003154775	c__Terriglobia	o__Acidoferrales	f__UBA7541	SPL	Soil	85.24	58.47	4631202	4101	962.78	30	4	28	69	53	30.56	5.25	Aerobic	Competitor
GCA_003154855	c__Terriglobia	o__Bryobacterales	f__Bryobacteraceae	SPL	Soil	88.86	64.77	4547715	4700	868.13	41	2	25	99	51	31.83	6.12	Anaerobic	Scarcity
GCA_003154935	c__Terriglobia	o__Terriglobales	f__SbA1	SPL	Soil	87.58	58.84	4343156	3904	975.70	22	1	32	42	62	27.27	4.68	Aerobic	Competitor
GCA_003155015	c__Terriglobia	o__Terriglobales	f__Acidobacteriaceae	SPL	Soil	85.47	60.00	4426527	4413	860.15	27	3	28	118	60	24.49	4.79	Aerobic	Competitor
GCA_003155155	c__Terriglobia	o__Terriglobales	f__SbA1	SPL	Soil	87.31	58.91	4303643	3939	958.20	23	1	36	42	65	27.62	5.00	Aerobic	Scarcity
GCA_003155215	c__Terriglobia	o__Terriglobales	f__SbA1	SPL	Soil	87.17	58.97	4138350	4021	899.03	22	0	40	39	67	26.62	4.88	Aerobic	Scarcity
GCA_003155295	c__Terriglobia	o__Terriglobales	f__SbA1	SPL	Soil	87.42	58.94	3729378	3465	942.11	15	0	36	27	59	27.06	5.00	Aerobic	Ruderal
GCA_003155955	c__Terriglobia	o__Bryobacterales	f__Bryobacteraceae	SPL	Soil	88.25	62.44	5691524	5160	974.58	38	3	12	141	65	29.48	5.18	Anaerobic	Competitor
GCA_003155975	c__Terriglobia	o__Terriglobales	f__Acidobacteriaceae	SPL	Soil	86.36	62.40	4162915	3979	907.16	22	3	20	129	47	23.63	4.97	Aerobic	Scarcity
GCA_003156155	c__Terriglobia	o__Terriglobales	f__Acidobacteriaceae	SPL	Soil	85.90	59.92	4449273	4524	848.18	30	0	32	120	56	24.42	5.37	Anaerobic	Competitor
GCA_003156235	c__Terriglobia	o__Terriglobales	f__Acidobacteriaceae	SPL	Soil	85.20	58.14	4381105	4358	858.08	38	5	36	74	48	26.02	5.16	Anaerobic	Competitor
GCA_003156475	c__Terriglobia	o__Terriglobales	f__SbA1	SPL	Soil	87.30	58.86	4273586	3923	955.71	24	0	40	42	66	27.2	5.00	Aerobic	Scarcity
GCA_003156495	c__Terriglobia	o__Terriglobales	f__Acidobacteriaceae	SPL	Soil	85.31	59.39	2828576	2726	887.26	14	1	20	68	43	27.11	5.27	Anaerobic	Competitor
GCA_003156535	c__Terriglobia	o__Bryobacterales	f__Bryobacteraceae	SPL	Soil	88.41	62.66	4981074	4582	961.91	32	2	12	129	64	29.35	5.23	Aerobic	Competitor
GCA_003156565	c__Terriglobia	o__Terriglobales	f__Acidobacteriaceae	SPL	Soil	85.84	60.01	3494373	3581	840.30	21	5	12	58	48	27.1	5.64	Anaerobic	Competitor
GCA_003156615	c__Terriglobia	o__Terriglobales	f__SbA1	SPL	Soil	83.60	57.70	7207389	7642	803.24	50	0	56	109	116	25.36	5.21	Aerobic	Competitor
GCA_003156635	c__Terriglobia	o__Acidoferrales	f__UBA7541	SPL	Soil	87.89	58.80	4181007	4025	916.65	35	1	32	44	45	24.24	5.49	Aerobic	Scarcity
GCA_003156775	c__Terriglobia	o__Acidoferrales	f__UBA7541	SPL	Soil	88.87	56.64	3023281	3106	873.86	32	0	36	23	33	26.42	5.39	Aerobic	Scarcity
GCA_003156805	c__Terriglobia	o__Terriglobales	f__SbA1	SPL	Soil	86.75	58.53	5032658	4469	978.68	29	1	69	65	68	27.04	4.97	Aerobic	Competitor
GCA_003156935	c__Terriglobia	o__Terriglobales	f__SbA1	SPL	Soil	86.30	57.71	5191133	5077	888.48	35	2	34	57	70	27.08	4.99	Aerobic	Competitor
GCA_003157135	c__Terriglobia	o__Terriglobales	f__SbA1	SPL	Soil	85.33	58.01	5661641	5916	823.20	46	1	46	115	79	27.69	5.38	Aerobic	Competitor
GCA_003157545	c__Terriglobia	o__Terriglobales	f__SbA1	SPL	Soil	85.64	57.96	5668081	5832	835.63	41	2	48	109	74	27.03	5.14	Anaerobic	Competitor
GCA_003157745	c__Terriglobia	o__Bryobacterales	f__Bryobacteraceae	SPL	Soil	88.57	63.26	5816078	5357	963.55	42	2	12	125	53	25.93	4.71	Anaerobic	Competitor
GCA_003158075	c__Terriglobia	o__Terriglobales	f__SbA1	SPL	Soil	86.77	58.94	2866633	2646	943.53	20	0	30	22	49	28.48	5.43	Aerobic	Competitor
GCA_003158125	c__Terriglobia	o__Terriglobales	f__Acidobacteriaceae	SPL	Soil	85.22	59.86	4477198	4507	852.00	28	0	24	124	59	25.09	5.21	Aerobic	Competitor
GCA_003158205	c__Terriglobia	o__Acidoferrales	f__UBA7541	SPL	Soil	87.35	58.68	4003027	4099	855.31	29	0	49	46	41	23.73	5.50	Aerobic	Scarcity
GCA_003158215	c__Terriglobia	o__Acidoferrales	f__UBA7541	SPL	Soil	87.08	56.68	3035955	3421	787.25	26	0	52	20	39	24.71	5.36	Aerobic	Scarcity
GCA_003159275	c__Terriglobia	o__Terriglobales	f__Acidobacteriaceae	SPL	Soil	86.95	60.03	3240527	3081	915.44	21	2	20	32	58	25.65	5.21	Aerobic	Competitor
GCA_003159355	c__Terriglobia	o__Terriglobales	f__Acidobacteriaceae	SPL	Soil	86.25	59.93	3575196	3593	862.45	25	5	24	63	36	26.71	5.47	Anaerobic	Competitor
GCA_003159365	c__Terriglobia	o__Terriglobales	f__Acidobacteriaceae	SPL	Soil	86.02	59.13	5780977	5666	888.17	52	2	48	124	85	23.58	5.50	Aerobic	Competitor
GCA_003159515	c__Terriglobia	o__Terriglobales	f__Acidobacteriaceae	SPL	Soil	86.70	61.41	3500242	3271	927.00	17	1	28	89	42	24.34	5.46	Anaerobic	Competitor
GCA_003159545	c__Terriglobia	o__Bryobacterales	f__Bryobacteraceae	SPL	Soil	88.45	62.40	5764657	5083	1003.04	37	2	12	141	61	29.32	5.18	Anaerobic	Competitor
GCA_003159575	c__Terriglobia	o__Terriglobales	f__Acidobacteriaceae	SPL	Soil	85.81	62.54	2868267	2599	947.76	15	1	12	67	39	24.97	5.40	Anaerobic	Competitor
GCA_003159715	c__Terriglobia	o__Bryobacterales	f__Bryobacteraceae	SPL	Soil	87.62	62.45	5331570	5149	912.98	32	0	12	133	62	29.01	5.22	Aerobic	Competitor
GCA_003159735	c__Terriglobia	o__Bryobacterales	f__Bryobacteraceae	SPL	Soil	88.47	62.52	6900820	6898	886.25	46	1	28	179	90	28.12	5.79	Aerobic	Scarcity
GCA_003159755	c__Terriglobia	o__Terriglobales	f__Acidobacteriaceae	SPL	Soil	85.93	60.08	3720188	3751	850.97	30	2	24	65	46	24.96	4.87	Aerobic	Competitor
GCA_003159785	c__Terriglobia	o__Terriglobales	f__Acidobacteriaceae	SPL	Soil	85.48	59.85	4557546	4349	902.20	27	1	20	123	60	25.3	5.24	Aerobic	Competitor
GCA_003159855	c__Terriglobia	o__Terriglobales	f__Acidobacteriaceae	SPL	Soil	86.82	59.85	3387116	3230	911.88	24	2	20	32	64	25.3	4.96	Anaerobic	Competitor
GCA_003159875	c__Terriglobia	o__Terriglobales	f__Acidobacteriaceae	SPL	Soil	86.16	61.66	3069548	2849	930.92	15	0	20	65	37	25.3	6.03	Anaerobic	Competitor
GCA_003159895	c__Terriglobia	o__Terriglobales	f__Acidobacteriaceae	SPL	Soil	86.02	60.11	3840820	3755	880.99	25	0	32	67	51	25.43	4.64	Aerobic	Scarcity
GCA_003159945	c__Terriglobia	o__Terriglobales	f__Acidobacteriaceae	SPL	Soil	85.85	59.82	4666888	4401	913.44	30	1	16	123	55	24.7	5.23	Anaerobic	Competitor
GCA_003159975	c__Terriglobia	o__Terriglobales	f__Acidobacteriaceae	SPL	Soil	85.80	59.12	5742999	5697	877.87	50	2	37	111	80	23.7	5.29	Aerobic	Competitor
GCA_003159995	c__Terriglobia	o__Terriglobales	f__Acidobacteriaceae	SPL	Soil	86.71	59.17	4076722	4072	869.53	31	6	20	77	39	27.67	5.48	Anaerobic	Competitor

GCA_003160025	c__Terriglobia	o__Terriglobales	f__Acidobacteriaceae	SPL	Soil	86.71	61.25	3486375	3120	969.87	20	2	22	62	34	26.19	5.17	Anaerobic	Competitor
GCA_003160185	c__Terriglobia	o__Bryobacterales	f__Bryobacteraceae	SPL	Soil	87.48	62.41	5456354	5199	925.52	37	1	16	137	67	29.36	5.12	Anaerobic	Competitor
GCA_003160215	c__Terriglobia	o__Terriglobales	f__Koribacteraceae	SPL	Soil	83.52	58.00	5343426	5258	850.52	39	5	12	37	68	32.32	6.42	Aerobic	Competitor
GCA_003160315	c__Terriglobia	o__Bryobacterales	f__Bryobacteraceae	SPL	Soil	87.80	62.61	4737408	4508	927.99	32	0	8	111	60	28.27	5.28	Anaerobic	Competitor
GCA_003160345	c__Terriglobia	o__Bryobacterales	f__Bryobacteraceae	SPL	Soil	88.42	62.59	7275386	7209	897.42	46	3	24	178	102	28.08	5.92	Aerobic	Scarcity
GCA_003160385	c__Terriglobia	o__Terriglobales	f__SbA1	SPL	Soil	85.61	58.11	5462416	4857	961.97	49	0	16	96	61	30.88	4.97	Anaerobic	Competitor
GCA_003160495	c__Terriglobia	o__Terriglobales	f__SbA1	SPL	Soil	87.49	58.84	4285919	3982	945.28	21	1	37	41	69	27.27	4.88	Aerobic	Scarcity
GCA_003160555	c__Terriglobia	o__Terriglobales	f__SbA1	SPL	Soil	86.92	58.88	4271862	3994	935.17	21	0	40	43	65	27.21	4.95	Aerobic	Scarcity
GCA_003160595	c__Terriglobia	o__Terriglobales	f__Acidobacteriaceae	SPL	Soil	86.51	61.30	3331541	3027	953.68	19	2	22	58	36	26.6	5.17	Aerobic	Competitor
GCA_003160875	c__Terriglobia	o__Bryobacterales	f__Bryobacteraceae	SPL	Soil	86.63	62.42	4383505	4519	857.81	34	1	8	98	63	28.76	5.57	Anaerobic	Competitor
GCA_003160955	c__Terriglobia	o__Terriglobales	f__Acidobacteriaceae	SPL	Soil	85.46	59.79	4531811	4496	868.39	34	0	28	119	58	24.72	5.17	Anaerobic	Competitor
GCA_003160995	c__Terriglobia	o__Terriglobales	f__SbA1	SPL	Soil	86.95	58.81	4236247	3988	927.85	23	0	28	42	65	27.33	5.11	Aerobic	Scarcity
GCA_003161035	c__Terriglobia	o__Terriglobales	f__Acidobacteriaceae	SPL	Soil	86.12	62.27	2729199	2590	908.66	15	0	12	41	34	25.56	5.46	Anaerobic	Ruderal
GCA_003161045	c__Terriglobia	o__Terriglobales	f__Acidobacteriaceae	SPL	Soil	83.62	62.30	2724402	2471	923.33	19	1	12	14	28	26.78	5.36	Anaerobic	Competitor
GCA_003161075	c__Terriglobia	o__Terriglobales	f__Acidobacteriaceae	SPL	Soil	86.79	62.17	4599469	4247	941.51	27	2	16	145	54	23.67	5.04	Aerobic	Scarcity
GCA_003161105	c__Terriglobia	o__Terriglobales	f__Acidobacteriaceae	SPL	Soil	86.82	59.71	3614498	3681	853.07	29	3	12	65	43	27.81	5.44	Anaerobic	Competitor
GCA_003161195	c__Terriglobia	o__Acidoferrales	f__UBA7541	SPL	Soil	86.34	59.95	4354418	4084	927.58	30	3	36	61	57	24.79	5.01	Aerobic	Scarcity
GCA_003161315	c__Terriglobia	o__Bryobacterales	f__Bryobacteraceae	SPL	Soil	86.17	62.49	4163257	4349	841.76	32	1	12	89	52	29.59	5.21	Anaerobic	Scarcity
GCA_003161365	c__Terriglobia	o__Terriglobales	f__Acidobacteriaceae	SPL	Soil	86.82	60.32	3078575	2914	919.88	18	0	20	28	64	25.46	5.15	Aerobic	Competitor
GCA_003161455	c__Terriglobia	o__Terriglobales	f__SbA1	SPL	Soil	87.18	56.84	5586781	5157	945.99	33	2	36	62	69	25.74	4.72	Aerobic	Competitor
GCA_003161495	c__Terriglobia	o__Acidoferrales	f__UBA7541	SPL	Soil	85.79	56.85	3933913	4053	841.70	20	3	40	19	33	25.18	5.96	Aerobic	Scarcity
GCA_003161595	c__Terriglobia	o__Terriglobales	f__Koribacteraceae	SPL	Soil	85.45	58.82	3896755	3939	846.51	27	1	16	21	66	32.1	5.59	Anaerobic	Scarcity
GCA_003162255	c__Terriglobia	o__Terriglobales	f__SbA1	SPL	Soil	85.72	58.01	6056979	5978	870.51	46	1	52	117	86	27.39	4.85	Anaerobic	Competitor
GCA_003162425	c__Terriglobia	o__Bryobacterales	f__Bryobacteraceae	SPL	Soil	90.56	58.21	8721485	8065	980.77	82	4	77	122	99	26.75	4.66	Aerobic	Ruderal
GCA_003162495	c__Terriglobia	o__Terriglobales	f__Acidobacteriaceae	SPL	Soil	86.41	60.56	2822123	2837	871.84	24	2	4	58	56	23.31	5.73	Aerobic	Competitor
GCA_003162515	c__Terriglobia	o__Terriglobales	f__SbA1	SPL	Soil	87.39	57.23	4938030	4965	871.84	33	1	80	57	63	25.92	5.24	Aerobic	Scarcity
GCA_003162795	c__Terriglobia	o__Bryobacterales	f__Bryobacteraceae	SPL	Soil	88.54	62.45	5715130	5035	1004.87	37	2	16	139	62	29.39	5.18	Anaerobic	Ruderal
GCA_003162855	c__Terriglobia	o__Terriglobales	f__Acidobacteriaceae	SPL	Soil	86.99	60.42	2985672	2747	946.87	23	3	20	29	56	25.01	5.74	Aerobic	Competitor
GCA_003162875	c__Terriglobia	o__Terriglobales	f__Acidobacteriaceae	SPL	Soil	86.16	61.85	4837229	4452	937.90	21	2	20	151	52	23.59	4.97	Aerobic	Scarcity
GCA_003162935	c__Terriglobia	o__Terriglobales	f__SbA1	SPL	Soil	87.37	58.90	3639840	3295	969.48	20	1	24	36	55	27.35	5.13	Aerobic	Competitor
GCA_003163055	c__Terriglobia	o__Bryobacterales	f__Bryobacteraceae	SPL	Soil	88.42	62.43	5637098	5077	983.92	37	2	20	143	65	29.35	5.18	Anaerobic	Competitor
GCA_003163135	c__Terriglobia	o__Bryobacterales	f__Bryobacteraceae	SPL	Soil	87.43	62.40	5250976	5216	886.09	41	1	8	116	61	29.72	5.09	Aerobic	Competitor
GCA_003163155	c__Terriglobia	o__Bryobacterales	f__Bryobacteraceae	SPL	Soil	88.54	62.49	7436385	7265	908.65	50	1	36	176	90	27.8	5.78	Anaerobic	Scarcity
GCA_003163175	c__Terriglobia	o__Terriglobales	f__Acidobacteriaceae	SPL	Soil	82.44	60.92	3342643	3153	874.82	27	5	12	24	42	27.92	4.96	Anaerobic	Competitor
GCA_003163195	c__Terriglobia	o__Terriglobales	f__SbA1	SPL	Soil	85.50	58.12	4558710	4147	939.32	39	1	20	80	55	30.54	4.81	Anaerobic	Competitor
GCA_003163235	c__Terriglobia	o__Terriglobales	f__Acidobacteriaceae	SPL	Soil	84.93	60.42	6202278	5957	886.35	56	1	12	171	68	24.97	5.05	Aerobic	Competitor
GCA_003163435	c__Terriglobia	o__Bryobacterales	f__Bryobacteraceae	SPL	Soil	86.31	62.51	4563682	4633	861.82	34	0	4	102	58	29.09	5.03	Anaerobic	Scarcity
GCA_003163505	c__Terriglobia	o__Terriglobales	f__Acidobacteriaceae	SPL	Soil	85.96	59.92	4317540	4106	907.52	29	1	20	117	56	25.03	5.23	Anaerobic	Competitor
GCA_003163535	c__Terriglobia	o__Bryobacterales	f__Bryobacteraceae	SPL	Soil	87.45	62.69	4418030	4460	874.01	26	2	8	106	63	28.95	5.14	Aerobic	Scarcity
GCA_003163575	c__Terriglobia	o__Terriglobales	f__SbA1	SPL	Soil	86.98	58.92	4153523	4125	876.18	20	1	36	37	66	27.31	4.99	Aerobic	Competitor
GCA_003163615	c__Terriglobia	o__Terriglobales	f__Acidobacteriaceae	SPL	Soil	86.29	59.36	3982956	3984	868.78	32	1	24	78	40	26.58	5.57	Anaerobic	Competitor
GCA_003163715	c__Terriglobia	o__Terriglobales	f__Acidobacteriaceae	SPL	Soil	86.17	59.19	4033443	4126	848.29	27	3	16	74	41	26.81	4.99	Anaerobic	Competitor
GCA_003163735	c__Terriglobia	o__Terriglobales	f__Acidobacteriaceae	SPL	Soil	85.60	60.17	4026386	3935	878.69	27	1	28	74	58	25.41	4.63	Aerobic	Scarcity
GCA_003163835	c__Terriglobia	o__Bryobacterales	f__Bryobacteraceae	SPL	Soil	87.11	62.60	4751335	4811	869.36	35	0	20	113	61	29.26	4.98	Anaerobic	Competitor
GCA_003163855	c__Terriglobia	o__Bryobacterales	f__Bryobacteraceae	SPL	Soil	92.32	56.23	6334092	5761	1019.17	53	1	32	123	76	23.37	4.99	Aerobic	Scarcity
GCA_003163995	c__Terriglobia	o__Terriglobales	f__SbA1	SPL	Soil	87.51	58.85	4292572	3956	951.36	19	1	40	40	66	27.43	4.88	Aerobic	Competitor
GCA_003164035	c__Terriglobia	o__Bryobacterales	f__Bryobacteraceae	SPL	Soil	88.28	62.84	8012244	7148	992.13	56	8	41	171	89	27.57	5.40	Aerobic	Competitor
GCA_003164075	c__Terriglobia	o__Bryobacterales	f__Bryobacteraceae	SPL	Soil	84.75	61.74	7223734	7231	858.34	38	0	12	155	83	26.89	4.88	Aerobic	Competitor
GCA_003164255	c__Terriglobia	o__Terriglobales	f__Acidobacteriaceae	SPL	Soil	84.89	58.82	5112986	5067	872.51	41	0	36	149	74	24.58	4.58	Aerobic	Competitor
GCA_003164605	c__Terriglobia	o__Terriglobales	f__SbA1	SPL	Soil	86.48	58.80	4192994	4117	891.52	21	0	32	40	69	26.93	4.74	Aerobic	Scarcity
GCA_003164635	c__Terriglobia	o__Terriglobales	f__Acidobacteriaceae	SPL	Soil	86.65	58.97	4821837	4498	937.73	28	1	16	127	79	20.46	4.77	Aerobic	Competitor
GCA_003164855	c__Terriglobia	o__Terriglobales	f__SbA1	SPL	Soil	87.54	57.26	5230759	5061	907.41	38	1	61	74	77	25.05	4.96	Aerobic	Scarcity
GCA_003165005	c__Terriglobia	o__Terriglobales	f__Acidobacteriaceae	SPL	Soil	84.73	61.56	3898469	3643	907.26	21	0	28	63	35	25.9	5.44	Aerobic	Competitor
GCA_003165035	c__Terriglobia	o__Terriglobales	f__Koribacteraceae	SPL	Soil	84.04	58.40	4737259	4412	904.44	32	2	16	37	61	31.59	5.67	Aerobic	Scarcity
GCA_003165075	c__Terriglobia	o__Terriglobales	f__Acidobacteriaceae	SPL	Soil	86.54	58.77	5677172	4860	1011.56	34	6	36	173	65	26.87	4.71	Aerobic	Competitor
GCA_003165095	c__Terriglobia	o__Terriglobales	f__Acidobacteriaceae	SPL	Soil	85.65	60.25	4608566	4395	905.56	23	1	20	128	66	24	5.25	Aerobic	Competitor
GCA_003165175	c__Terriglobia	o__Terriglobales	f__Acidobacteriaceae	SPL	Soil	86.13	61.52	4824528	4611	905.24	27	4	16	158	52	23.33	5.44	Aerobic	Competitor
GCA_003165315	c__Terriglobia	o__UBA7540	f__UBA7540	SPL	Soil	83.71	56.45	6186033	5284	980.83	49	2	28	169	43	26.22	5.29	Anaerobic	Scarcity
GCA_003165455	c__Terriglobia	o__Terriglobales	f__SbA1	SPL	Soil	87.00	55.52	4101074	4134	863.11	29	0	32	44	55	25.46	5.09	Aerobic	Scarcity
GCA_003165465	c__Terriglobia	o__Acidoferrales	f__UBA7541	SPL	Soil	85.06	58.00	4617235	4045	971.70	30	2	36	25	43	26.63	5.57	Aerobic	Scarcity
GCA_003165535	c__Terriglobia	o__Terriglobales	f__Koribacteraceae	SPL	Soil	85.17	58.80	4662616	4335	916.30	41	3	12	23	58	32.43	6.32	Aerobic	Scarcity
GCA_003165555	c__Terriglobia	o__Terriglobales	f__Acidobacteriaceae	SPL	Soil	85.24	61.42	4228821	3920	918.93	24	0	28	70	40	26.98	5.06	Anaerobic	Competitor
GCA_003165575	c__Terriglobia	o__Terriglobales	f__Acidobacteriaceae	SPL	Soil	85.79	59.81	4642096	4546	877.41	29	2	20	123	58	24.67	5.48	Anaerobic	Competitor
GCA_003165615	c__Terriglobia	o__UBA7540	f__UBA7540	SPL	Soil	83.63	58.14	6236720	6029	864.96	46	7	43	161	50	30.32	5.52	Anaerobic	Scarcity
GCA_003165705	c__Terriglobia	o__Acidoferrales	f__UBA7541	SPL	Soil	85.62	58.24	4141319	3625	979.29	28	4	24	64	42	30.01	5.25	Aerobic	Competitor
GCA_003165755	c__Terriglobia	o__Terriglobales	f__Acidobacteriaceae	SPL	Soil	86.12	59.92	4380163	4294	879.17	27	1	33	123	53	24.91	5.21	Aerobic	Competitor
GCA_003165775	c__Terriglobia	o__Bryobacterales	f__Bryobacteraceae	SPL	Soil	88.11	62.48	5309478	4917	952.68	35	0	8	127	58	29.46	5.09	Aerobic	Competitor
GCA_003165795	c__Terriglobia	o__Terriglobales	f__Koribacteraceae	SPL	Soil	84.22	58.32	4953686	4646	897.99	34	1	20	37	56	31.52	5.65	Aerobic	Scarcity
GCA_003165855	c__Terriglobia	o__Terriglobales	f__SbA1	SPL	Soil	86.79	58.32	4874894	4999	846.08	32	1	61	66	68	26.56	5.43	Aerobic	Scarcity
GCA_003165935	c__Terriglobia	o__Terriglobales	f__Acidobacteriaceae	SPL	Soil	86.46	59.64	4757570	4635	888.98	32	2	28	126	63	24.34	5.20	Aerobic	Scarcity
GCA_003165995	c__Terriglobia	o__Terriglobales	f__Acidobacteriaceae	SPL	Soil	86.51	62.69	2595138	2435	921.39	13	2	20	61	37	24.23	5.57	Anaerobic	Scarcity
GCA_003166015	c__Terriglobia	o__Terriglobales	f__Acidobacteriaceae	SPL	Soil	86.95	61.61	4287228	3950	943.34	29	1	20	142	43	23.77	4.88	Aerobic	Competitor
GCA_003166055	c__Terriglobia	o__Terriglobales	f__Acidobacteriaceae	SPL	Soil	86.59	59.21	5329118	5371	860.42	39	4	45	113	86	23.86	4.47	Aerobic	Competitor
GCA_003166115	c__Terriglobia	o__Terriglobales	f__Acidobacteriaceae	SPL	Soil	85.76	59.92	4506350	4476	863.46	30	0	20	114	62	25.03	5.51	Aerobic	Competitor
GCA_003166155	c__Terriglobia	o__Terriglobales	f__SbA1	SPL	Soil	86.22	58.85	5290860	4870	937.04	33	3	32	28	62	27.31	4.93	Aerobic	Competitor
GCA_003166195	c__Terriglobia	o__Bryobacterales	f__Bryobacteraceae	SPL	Soil	88.86	62.59	7107763	7235	874.03	41	2	40	169	99	27.9	5.81	Aerobic	Scarcity
GCA_003166385	c__Terriglobia	o__Terriglobales	f__SbA1	SPL	Soil	86.41	58.87	5523653	4837	987.03	33	2	34	32	55	27.34	4.80	Aerobic	Competitor
GCA_003166415	c__Terriglobia	o__Terriglobales	f__Acidobacteriaceae	SPL	Soil	86.12	60.14	4186064	3946	914.37	29	2	24	78	57	25.46	4.95	Aerobic	Competitor
GCA_003166475	c__Terriglobia	o__Bryobacterales	f__Bryobacteraceae	SPL	Soil	88.84	62.59	7561945	7219	931.69	45	1	24	197	97	28.13	5.78	Aerobic	Scarcity
GCA_003166525	c__Terriglobia	o__Terriglobales	f__SbA1	SPL	Soil	85.53	58.10	5521633	4990	947.36	46	0	16	102	66	31.04	4.97	Anaerobic	Competitor
GCA_003166575	c__Terriglobia	o__Bryobacterales	f__Bryobacteraceae	SPL	Soil	87.69	62.38	5385439	5255	904.20	33	1	12	127	68	28.76	5.22	Anaerobic	Scarcity
GCA_003166675	c__Terriglobia	o__Terriglobales	f__SbA1	SPL	Soil	87.44	58.83	4233600	3963	933.81	20	0	36	42	66	27.4	4.88	Aerobic	Competitor
GCA_003166695	c__Terriglobia	o__Terriglobales	f__Acidobacteriaceae	SPL	Soil	85.54	59.43	5042223	5080	856.23	37	3	20	122	63	25.46	5.23	Anaerobic	Competitor
GCA_003166755	c__Terriglobia	o__Terriglobales	f__Acidobacteriaceae	SPL	Soil	86.64	61.60	4963643	4571	942.98	27	3	20	158	53	23.64	4.97	Aerobic	Scarcity
GCA_003166775	c__Terriglobia	o__Acidoferrales	f__UBA7541	SPL	Soil	87.73	57.44	4139356	4111	884.98	49	1	36	45	67	25.37	5.33	Aerobic	Scarcity
GCA_003166795	c__Terriglobia	o__Acidoferrales	f__UBA7541	SPL	Soil	88.22	59.95	3924320	3628	955.32	30	1	32	25	55	25.36	5.38	Aerobic	Competitor
GCA_003166815	c__Terriglobia	o__Terriglobales	f__SbA1	SPL	Soil	86.47	57.69	5249444	5124	889.26	45	3	32	60	70	27.26	5.23	Aerobic	Competitor

GCA_003166855	c__Terriglobia	o__Terriglobales	f__SbA1	SPL	Soil	86.70	58.33	6936785	6371	943.67	57	0	40	127	97	25.32	4.85	Aerobic	Competitor
GCA_003166865	c__Terriglobia	o__Bryobacterales	f__Bryobacteraceae	SPL	Soil	91.55	59.63	6674750	6384	959.44	52	4	65	46	98	27.51	5.88	Aerobic	Competitor
GCA_003166875	c__Terriglobia	o__Bryobacterales	f__Bryobacteraceae	SPL	Soil	90.20	58.28	8015520	7537	959.48	69	1	68	113	94	27.14	4.77	Aerobic	Competitor
GCA_003166935	c__Terriglobia	o__Terriglobales	f__SbA1	SPL	Soil	86.39	56.72	5576224	5360	899.31	42	1	36	79	77	26.75	4.93	Aerobic	Competitor
GCA_003166955	c__Terriglobia	o__Terriglobales	f__Acidobacteriaceae	SPL	Soil	87.11	61.72	5409550	4653	1013.56	40	0	28	120	76	25.02	5.14	Aerobic	Competitor
GCA_003166975	c__Terriglobia	o__Terriglobales	f__Acidobacteriaceae	SPL	Soil	86.48	57.93	5194164	5206	864.36	35	4	28	119	64	23.97	4.74	Aerobic	Competitor
GCA_003166995	c__Terriglobia	o__Acidoferrales	f__UBA7541	SPL	Soil	87.75	56.21	3943197	3932	881.28	30	1	48	23	56	23.22	5.81	Aerobic	Scarcity
GCA_003167075	c__Terriglobia	o__Terriglobales	f__SbA1	SPL	Soil	86.57	57.25	5187149	5233	872.21	34	1	72	59	63	25.61	5.91	Aerobic	Scarcity
GCA_003167095	c__Terriglobia	o__Terriglobales	f__SbA1	SPL	Soil	86.72	57.68	5148011	5006	892.43	37	1	40	62	73	27.03	5.30	Aerobic	Competitor
GCA_003167175	c__Terriglobia	o__Bryobacterales	f__Bryobacteraceae	SPL	Soil	91.37	59.66	6407025	6587	890.14	52	3	53	47	92	27.33	5.93	Aerobic	Competitor
GCA_003167185	c__Terriglobia	o__Bryobacterales	f__Bryobacteraceae	SPL	Soil	90.50	58.35	7976792	7444	968.69	70	0	72	121	97	27.09	5.23	Aerobic	Ruderal
GCA_003167215	c__Terriglobia	o__Acidoferrales	f__UBA7541	SPL	Soil	89.73	56.43	3779963	3389	1002.60	37	2	30	20	39	26.82	5.10	Aerobic	Competitor
GCA_003167225	c__Terriglobia	o__Terriglobales	f__Koribacteraceae	SPL	Soil	85.76	58.72	4322897	4109	902.10	31	2	16	24	66	32.71	5.68	Anaerobic	Scarcity
GCA_003167255	c__Terriglobia	o__Bryobacterales	f__Bryobacteraceae	SPL	Soil	88.79	62.24	7351839	6425	1016.15	54	1	36	169	80	26.43	5.33	Anaerobic	Competitor
GCA_003167275	c__Terriglobia	o__Bryobacterales	f__Bryobacteraceae	SPL	Soil	87.60	61.69	11508716	10529	962.86	84	3	48	203	123	25.37	5.09	Anaerobic	Ruderal
GCA_003167305	c__Terriglobia	o__Terriglobales	f__Acidobacteriaceae	SPL	Soil	85.93	60.63	2478023	2434	876.43	17	1	12	45	27	28.32	5.77	Anaerobic	Competitor
GCA_003167375	c__Terriglobia	o__Bryobacterales	f__Bryobacteraceae	SPL	Soil	90.92	62.92	7126901	6366	1019.42	47	6	28	195	91	27.97	5.69	Anaerobic	Competitor
GCA_003167495	c__Terriglobia	o__Bryobacterales	f__Bryobacteraceae	SPL	Soil	90.63	55.98	9386293	8791	970.32	84	4	52	126	105	22.63	5.15	Aerobic	Ruderal
GCA_003167515	c__Terriglobia	o__Terriglobales	f__Acidobacteriaceae	SPL	Soil	87.11	60.74	5028386	4881	897.46	37	0	28	96	87	24.92	4.86	Aerobic	Competitor
GCA_003167545	c__Terriglobia	o__Terriglobales	f__Acidobacteriaceae	SPL	Soil	86.45	58.11	4437340	4584	838.28	33	1	41	123	46	24.84	5.83	Aerobic	Competitor
GCA_003167655	c__Terriglobia	o__Terriglobales	f__Acidobacteriaceae	SPL	Soil	85.44	60.40	6346841	5774	938.79	54	2	8	173	64	25.46	5.25	Aerobic	Ruderal
GCA_003167755	c__Terriglobia	o__Terriglobales	f__Acidobacteriaceae	SPL	Soil	82.94	62.02	2753970	2612	874.30	18	1	12	15	32	25.83	5.36	Anaerobic	Competitor
GCA_003167855	c__Terriglobia	o__Terriglobales	f__Koribacteraceae	SPL	Soil	84.56	58.52	4131384	4232	826.09	31	0	20	34	55	32.28	5.84	Aerobic	Scarcity
GCA_003167875	c__Terriglobia	o__Terriglobales	f__SbA1	SPL	Soil	87.53	58.87	4270125	3984	940.25	21	0	36	38	64	27.26	5.00	Aerobic	Competitor
GCA_003167885	c__Terriglobia	o__Bryobacterales	f__Bryobacteraceae	SPL	Soil	88.42	62.40	5649593	5042	991.41	38	1	12	137	58	29.82	5.24	Anaerobic	Competitor
GCA_003167955	c__Terriglobia	o__Terriglobales	f__SbA1	SPL	Soil	87.16	56.87	5519839	5157	934.52	34	3	32	62	69	25.61	4.85	Aerobic	Competitor
GCA_003168015	c__Terriglobia	o__Terriglobales	f__SbA1	SPL	Soil	86.53	56.63	6021114	5583	933.48	46	3	32	89	82	27.03	4.90	Aerobic	Competitor
GCA_003168235	c__Terriglobia	o__Terriglobales	f__SbA1	SPL	Soil	86.72	57.71	3730614	3848	842.76	30	2	28	29	66	25.84	5.71	Aerobic	Competitor
GCA_003168355	c__Terriglobia	o__Terriglobales	f__SbA1	SPL	Soil	86.90	57.69	5300178	5034	916.64	38	1	36	64	70	26.95	5.25	Aerobic	Ruderal
GCA_003168655	c__Terriglobia	o__Terriglobales	f__Acidobacteriaceae	SPL	Soil	82.40	62.21	2526683	2521	828.18	19	2	8	13	32	25.45	5.24	Anaerobic	Competitor
GCA_003168695	c__Terriglobia	o__Terriglobales	f__SbA1	SPL	Soil	86.39	58.86	5515153	4860	980.99	33	2	38	32	59	27.29	5.01	Aerobic	Competitor
GCA_003168715	c__Terriglobia	o__Bryobacterales	f__Bryobacteraceae	SPL	Soil	88.60	62.50	7838404	7548	923.80	53	1	32	196	94	28.28	5.78	Aerobic	Scarcity
GCA_003168875	c__Terriglobia	o__Terriglobales	f__Acidobacteriaceae	SPL	Soil	87.33	62.67	3828712	3597	931.42	20	2	20	115	51	23.84	4.97	Aerobic	Scarcity
GCA_003168935	c__Terriglobia	o__Terriglobales	f__SbA1	SPL	Soil	87.45	58.86	4331579	3988	952.08	22	0	38	43	66	27	4.88	Aerobic	Competitor
GCA_003168955	c__Terriglobia	o__Bryobacterales	f__Bryobacteraceae	SPL	Soil	92.47	59.69	5276219	5342	913.22	40	2	28	99	65	23.79	4.99	Aerobic	Scarcity
GCA_003169015	c__Terriglobia	o__Terriglobales	f__SbA1	SPL	Soil	86.35	58.94	5238957	4896	924.97	33	2	24	30	66	27.32	4.95	Aerobic	Competitor
GCA_003169125	c__Terriglobia	o__Bryobacterales	f__Bryobacteraceae	SPL	Soil	88.69	62.59	7365013	7103	921.15	51	0	28	184	88	27.98	5.63	Anaerobic	Scarcity
GCA_003169195	c__Terriglobia	o__Bryobacterales	f__Bryobacteraceae	SPL	Soil	91.19	55.37	7819467	7843	912.17	57	3	52	124	108	21.19	5.75	Aerobic	Competitor
GCA_003169315	c__Terriglobia	o__Terriglobales	f__SbA1	SPL	Soil	87.01	58.78	4202907	4045	911.73	21	0	32	43	59	27.11	4.79	Aerobic	Scarcity
GCA_003169465	c__Terriglobia	o__Terriglobales	f__SbA1	SPL	Soil	87.17	58.84	4176028	3947	926.89	24	0	36	43	66	27.38	5.11	Aerobic	Scarcity
GCA_003169515	c__Terriglobia	o__Terriglobales	f__Acidobacteriaceae	SPL	Soil	82.65	61.78	2735094	2756	822.76	21	2	20	18	34	26.42	5.63	Anaerobic	Competitor
GCA_003169535	c__Terriglobia	o__Terriglobales	f__Acidobacteriaceae	SPL	Soil	86.62	61.24	3550282	3252	946.57	19	3	22	64	36	26.84	5.31	Anaerobic	Competitor
GCA_003169605	c__Terriglobia	o__Terriglobales	f__SbA1	SPL	Soil	87.48	58.85	4332602	4075	931.19	24	0	28	41	71	27.16	4.86	Aerobic	Scarcity
GCA_003169715	c__Terriglobia	o__Terriglobales	f__SbA1	SPL	Soil	87.57	53.83	4366525	4449	861.57	28	2	20	79	48	24.36	5.23	Aerobic	Scarcity
GCA_003170295	c__Terriglobia	o__Bryobacterales	f__Bryobacteraceae	SPL	Soil	90.47	58.53	6206968	5935	947.82	47	2	20	59	70	25.25	5.34	Aerobic	Competitor
GCA_003170435	c__Terriglobia	o__Acidoferrales	f__UBA7541	SPL	Soil	86.37	58.74	3412846	2966	995.48	23	1	28	57	43	30.53	5.43	Anaerobic	Scarcity
GCA_003170495	c__Terriglobia	o__Terriglobales	f__Acidobacteriaceae	SPL	Soil	86.76	61.20	3693469	3323	965.06	20	1	20	64	40	26.72	5.21	Aerobic	Ruderal
GCA_003170825	c__Terriglobia	o__Terriglobales	f__Acidobacteriaceae	SPL	Soil	86.72	61.26	3493398	3103	977.51	20	2	22	57	35	26.89	5.17	Aerobic	Competitor
GCA_003170915	c__Terriglobia	o__Bryobacterales	f__Bryobacteraceae	SPL	Soil	90.12	62.93	5061459	4813	950.43	26	4	8	123	67	30.11	5.75	Anaerobic	Scarcity
GCA_003171295	c__Terriglobia	o__UBA7540	f__UBA7540	SPL	Soil	83.37	59.03	7594287	7524	847.61	70	11	32	198	46	28.62	5.77	Anaerobic	Scarcity
GCA_003171315	c__Terriglobia	o__Terriglobales	f__Acidobacteriaceae	SPL	Soil	85.60	61.46	3286184	2961	950.76	21	1	12	61	38	29.67	5.37	Aerobic	Competitor
GCA_003171475	c__Terriglobia	o__Terriglobales	f__Acidobacteriaceae	SPL	Soil	85.69	58.68	5188055	4807	927.20	39	1	32	138	43	25.08	5.40	Anaerobic	Competitor
GCA_003171495	c__Terriglobia	o__Terriglobales	f__Acidobacteriaceae	SPL	Soil	86.74	59.58	3446686	3268	921.46	26	1	16	60	50	26.05	5.21	Anaerobic	Competitor
GCA_003175985	c__Terriglobia	o__Bryobacterales	f__Bryobacteraceae	SPL	Soil	93.24	60.09	7442430	6666	1042.43	44	4	44	55	91	28.13	6.09	Aerobic	Scarcity
GCA_003222755	c__Terriglobia	o__20CM_2_55_15	f__20CM_2_55_15	SPL	Soil	86.00	55.77	6806257	7265	850.63	41	1	40	33	75	29.18	6.07	Aerobic	Scarcity
GCA_003222765	c__Terriglobia	o__20CM_2_55_15	f__20CM_2_55_15	SPL	Soil	88.71	55.19	6014048	6195	872.45	41	0	28	28	72	29.66	6.43	Aerobic	Scarcity
GCA_003222815	c__Terriglobia	o__20CM_2_55_15	f__20CM_2_55_15	SPL	Soil	89.57	56.13	5673645	5814	878.73	33	0	20	28	73	29.29	5.62	Aerobic	Scarcity
GCA_003223065	c__Terriglobia	o__Bryobacterales	f__Bryobacteraceae	SPL	Soil	89.49	60.46	6205811	5979	952.51	43	3	24	79	82	30.64	6.05	Aerobic	Scarcity
GCA_003223115	c__Terriglobia	o__Bryobacterales	f__Bryobacteraceae	SPL	Soil	85.25	58.43	9137547	9309	839.67	71	1	16	109	82	27.24	5.65	Aerobic	Ruderal
GCA_003223195	c__Terriglobia	o__Acidoferrales	f__UBA7541	SPL	Soil	86.54	57.24	5775415	5520	919.31	45	0	32	92	62	25.77	5.88	Aerobic	Competitor
GCA_003223215	c__Terriglobia	o__Acidoferrales	f__UBA7541	SPL	Soil	84.38	57.75	8802287	8083	922.20	74	1	42	105	82	25.39	4.87	Aerobic	Competitor
GCA_003223225	c__Terriglobia	o__Acidoferrales	f__UBA7541	SPL	Soil	84.50	57.84	7832187	7745	857.19	70	1	32	96	91	25.46	4.89	Aerobic	Competitor
GCA_003223245	c__Terriglobia	o__Acidoferrales	f__UBA7541	SPL	Soil	86.00	57.51	6311796	5686	958.11	53	0	50	102	56	27.62	5.50	Aerobic	Competitor
GCA_003223255	c__Terriglobia	o__Acidoferrales	f__UBA7541	SPL	Soil	84.65	57.45	6661743	6578	865.37	50	1	52	94	63	27.03	5.35	Aerobic	Competitor
GCA_003223295	c__Terriglobia	o__Acidoferrales	f__UBA7541	SPL	Soil	86.91	57.14	6032980	5593	936.68	44	3	66	81	63	26.07	5.68	Aerobic	Ruderal
GCA_003223315	c__Terriglobia	o__Acidoferrales	f__UBA7541	SPL	Soil	86.65	58.63	4295915	4259	873.25	26	2	24	16	47	30.05	5.28	Aerobic	Scarcity
GCA_003223325	c__Terriglobia	o__Acidoferrales	f__UBA7541	SPL	Soil	82.77	57.44	6104530	6261	812.12	48	2	21	26	57	28.67	5.59	Anaerobic	Scarcity
GCA_003223345	c__Terriglobia	o__Acidoferrales	f__UBA7541	SPL	Soil	84.39	61.02	4147982	4277	831.94	28	0	28	33	57	35.53	5.95	Aerobic	Scarcity
GCA_003223515	c__Terriglobia	o__Terriglobales	f__Gp1_AA117	SPL	Soil	86.00	54.75	6482446	5578	1000.48	37	3	159	25	64	29.5	4.76	Aerobic	Scarcity
GCA_003223595	c__Terriglobia	o__Terriglobales	f__SbA1	SPL	Soil	88.24	57.61	4375404	4565	850.34	40	0	28	78	64	26.74	5.33	Aerobic	Scarcity
GCA_003223875	c__Terriglobia	o__20CM_2_55_15	f__20CM_2_55_15	SPL	Soil	88.89	55.29	6577170	6318	926.76	43	1	28	33	64	29.61	5.87	Aerobic	Scarcity
GCA_003223895	c__Terriglobia	o__20CM_2_55_15	f__20CM_2_55_15	SPL	Soil	88.98	57.10	7088249	7254	886.78	59	0	56	26	86	28.01	6.08	Aerobic	Scarcity
GCA_003224025	c__Terriglobia	o__Acidoferrales	f__UBA7541	SPL	Soil	87.58	57.31	4952046	4699	927.08	48	1	24	75	57	26.61	5.91	Aerobic	Competitor
GCA_003224055	c__Terriglobia	o__Acidoferrales	f__UBA7541	SPL	Soil	88.09	58.02	4527594	4150	964.09	41	2	24	49	52	26.63	5.42	Aerobic	Competitor
GCA_003224075	c__Terriglobia	o__Acidoferrales	f__UBA7541	SPL	Soil	87.45	58.37	3422297	3501	860.10	26	1	28	42	52	30.08	5.46	Aerobic	Competitor
GCA_003224085	c__Terriglobia	o__Acidoferrales	f__UBA7541	SPL	Soil	86.11	57.44	6802862	6100	960.96	69	1	46	85	67	25.77	5.49	Aerobic	Competitor
GCA_003224105	c__Terriglobia	o__Acidoferrales	f__UBA7541	SPL	Soil	88.38	57.28	5127231	5020	902.36	50	1	36	73	59	25.43	5.61	Aerobic	Competitor
GCA_003224135	c__Terriglobia	o__Acidoferrales	f__UBA7541	SPL	Soil	87.12	56.21	4543519	4915	813.98	49	0	36	63	73	23.82	5.70	Aerobic	Competitor
GCA_003224145	c__Terriglobia	o__Acidoferrales	f__UBA7541	SPL	Soil	87.55	57.67	4787338	4455	940.49	29	3	32	45	50	28.37	5.56	Aerobic	Scarcity
GCA_003224175	c__Terriglobia	o__Acidoferrales	f__UBA7541	SPL	Soil	85.24	57.79	5618620	5787	826.97	34	0	17	27	52	28.93	5.56	Aerobic	Scarcity
GCA_003224195	c__Terriglobia	o__Acidoferrales	f__UBA7541	SPL	Soil	86.86	60.36	3838184	3744	902.62	28	1	28	43	49	30.24	5.54	Aerobic	Scarcity
GCA_003224235	c__Terriglobia	o__Acidoferrales	f__UBA7541	SPL	Soil	85.49	60.78	4565077	4134	945.82	33	1	36	35	62	34.11	5.70	Aerobic	Scarcity
GCA_003224475	c__Terriglobia	o__Acidoferrales	f__UBA7541	SPL	Soil	85.87	57.52	8112309	7429	938.10	82	1	32	100	68	24.33	5.30	Aerobic	Ruderal
GCA_003224495	c__Terriglobia	o__Acidoferrales	f__UBA7541	SPL	Soil	87.88	58.21	4304774	4348	867.54	42	0	32	61	60	25.41	5.57	Aerobic	Scarcity
GCA_003224515	c__Terriglobia	o__Acidoferrales	f__UBA7541	SPL	Soil	83.26	57.27	5528223	5928	803.33	37	1	28	77	65	24.38	5.46	Aerobic	Scarcity
GCA_003224525	c__Terriglobia	o__Acidoferrales	f__UBA7541	SPL	Soil	85.07	57.47	6474271	6190	892.36	49	1	36	91	71	24.85	6.07	Aerobic	Competitor
GCA_003224585	c__Terriglobia	o__UBA7540	f__UBA7540	SPL	Soil	84.64	61.15	5149998	4965	881.51	52	2	36	86	49	28.42	5.44	Aerobic	Scarcity

GCA_003224635	c__Terriglobia	o__UBA7540	f__UBA7540	SPL	Soil	84.77	61.40	4387442	4164	894.64	40	3	28	69	33	28.54	5.93	Aerobic	Scarcity
GCA_003224665	c__Terriglobia	o__UBA7540	f__UBA7540	SPL	Soil	86.00	60.33	3206714	3255	847.17	32	2	12	58	29	35.17	5.93	Aerobic	Scarcity
GCA_003224695	c__Terriglobia	o__UBA7540	f__UBA7540	SPL	Soil	84.62	60.19	4619930	4607	868.61	45	2	14	89	47	27.59	5.20	Aerobic	Scarcity
GCA_003224715	c__Terriglobia	o__VXMN01	f__VXMN01	SPL	Soil	82.81	52.75	6637645	6474	864.92	70	0	80	86	54	27.54	5.80	Aerobic	Scarcity
GCA_003224725	c__Terriglobia	o__VXMN01	f__VXMN01	SPL	Soil	82.76	52.59	6626859	6475	857.98	62	0	24	81	49	28.85	6.06	Aerobic	Scarcity
GCA_003224755	c__Terriglobia	o__Terriglobales	f__SbA1	SPL	Soil	86.09	55.35	5680480	6026	814.96	38	1	28	100	60	25.14	5.61	Aerobic	Competitor
GCA_003224775	c__Terriglobia	o__Terriglobales	f__SbA1	SPL	Soil	85.38	55.14	8604078	9068	812.58	78	2	57	148	69	23.31	5.31	Aerobic	Scarcity
GCA_003224785	c__Terriglobia	o__Terriglobales	f__SbA1	SPL	Soil	87.63	55.26	2609585	2952	787.10	19	0	20	48	41	25.85	5.90	Aerobic	Scarcity
GCA_003224815	c__Terriglobia	o__Terriglobales	f__SbA1	SPL	Soil	87.73	55.22	4553509	4725	847.36	38	2	32	100	64	26.6	5.87	Aerobic	Scarcity
GCA_003224835	c__Terriglobia	o__Terriglobales	f__SbA1	SPL	Soil	88.07	54.38	3659279	3896	833.46	27	1	16	71	55	27.11	5.31	Aerobic	Scarcity
GCA_003224855	c__Terriglobia	o__Terriglobales	f__SbA1	SPL	Soil	89.90	56.34	3736551	3742	900.46	27	2	16	34	58	28.79	6.33	Aerobic	Scarcity
GCA_003224895	c__Terriglobia	o__Terriglobales	f__SbA1	SPL	Soil	86.27	55.15	4428978	4940	782.74	43	1	40	49	69	28.55	5.99	Aerobic	Scarcity
GCA_003224905	c__Terriglobia	o__Terriglobales	f__SbA1	SPL	Soil	87.22	55.88	5641131	5485	898.34	45	1	24	71	68	27.51	5.16	Aerobic	Competitor
GCA_003224935	c__Terriglobia	o__Terriglobales	f__SbA1	SPL	Soil	86.29	55.16	4117340	4713	759.73	39	0	48	50	67	27.37	5.79	Aerobic	Scarcity
GCA_003224975	c__Terriglobia	o__Terriglobales	f__SbA1	SPL	Soil	84.48	57.74	6040864	5846	872.86	41	5	53	81	78	27.53	5.58	Aerobic	Competitor
GCA_003224995	c__Terriglobia	o__Terriglobales	f__SbA1	SPL	Soil	84.04	57.38	4074419	4546	770.45	19	0	20	57	70	27.47	5.84	Aerobic	Scarcity
GCA_003225005	c__Terriglobia	o__Terriglobales	f__SbA1	SPL	Soil	83.54	56.63	7896092	7962	829.50	63	2	54	108	87	26.93	5.50	Aerobic	Scarcity
GCA_003225035	c__Terriglobia	o__Terriglobales	f__SbA1	SPL	Soil	85.24	55.57	2968528	3331	782.52	22	0	28	35	56	30.09	5.57	Aerobic	Scarcity
GCA_003225055	c__Terriglobia	o__Terriglobales	f__SbA1	SPL	Soil	83.47	57.51	7411304	6816	910.76	56	3	16	162	75	27.47	5.36	Aerobic	Competitor
GCA_003225075	c__Terriglobia	o__Terriglobales	f__SbA1	SPL	Soil	86.89	55.59	4789841	4729	880.40	34	4	50	52	70	29.22	5.96	Aerobic	Competitor
GCA_003225115	c__Terriglobia	o__Terriglobales	f__Gp1_AA112	SPL	Soil	86.34	55.23	4335137	4458	851.26	29	3	37	79	63	26.95	5.66	Aerobic	Scarcity
GCA_003225155	c__Terriglobia	o__Terriglobales	f__Gp1_AA112	SPL	Soil	86.77	55.14	3865378	4117	815.46	28	1	32	71	63	27.4	6.48	Aerobic	Scarcity
GCA_003225175	c__Terriglobia	o__Terriglobales	f__Gp1_AA112	SPL	Soil	85.17	54.35	5732271	5177	944.37	44	3	38	95	67	27.02	5.58	Aerobic	Competitor
GCA_003225195	c__Terriglobia	o__Terriglobales	f__Gp1_AA112	SPL	Soil	82.13	55.69	4787621	5288	784.91	39	2	34	87	58	26.6	5.85	Aerobic	Scarcity
GCA_003225215	c__Terriglobia	o__Terriglobales	f__Gp1_AA112	SPL	Soil	86.07	55.74	4943230	4782	890.73	41	3	25	85	63	27.64	5.99	Aerobic	Competitor
GCA_003225235	c__Terriglobia	o__Acidoferrales	f__UBA7541	SPL	Soil	84.53	57.89	6142482	6229	852.78	56	1	40	77	78	24.37	5.37	Aerobic	Competitor
GCA_003225255	c__Terriglobia	o__Acidoferrales	f__UBA7541	SPL	Soil	84.70	57.61	7259636	6929	902.88	69	1	44	97	67	23.84	5.42	Aerobic	Competitor
GCA_003225275	c__Terriglobia	o__Acidoferrales	f__UBA7541	SPL	Soil	85.93	57.64	6291825	6166	876.56	63	1	36	92	74	24.89	5.59	Aerobic	Competitor
GCA_003225295	c__Terriglobia	o__Acidoferrales	f__UBA7541	SPL	Soil	85.79	57.50	8134361	7411	942.46	85	2	40	99	71	24.49	5.53	Aerobic	Competitor
GCA_003225315	c__Terriglobia	o__Acidoferrales	f__UBA7541	SPL	Soil	88.83	57.07	4975678	4453	994.37	34	3	61	71	49	25.9	5.58	Aerobic	Competitor
GCA_003225365	c__Terriglobia	o__UBA7540	f__UBA7540	SPL	Soil	86.04	60.23	5164498	4836	918.63	48	2	20	92	45	28.89	5.56	Aerobic	Scarcity
GCA_003225435	c__Terriglobia	o__Terriglobales	f__SbA1	SPL	Soil	85.33	56.25	4374376	4582	845.13	29	1	12	38	67	26.89	5.86	Aerobic	Scarcity
GCA_003225455	c__Terriglobia	o__Terriglobales	f__SbA1	SPL	Soil	85.83	55.75	4993208	5026	867.18	45	0	36	45	69	28.66	5.26	Aerobic	Scarcity
GCA_003225465	c__Terriglobia	o__Terriglobales	f__SbA1	SPL	Soil	84.49	57.73	6149199	5900	880.15	44	2	49	72	88	27.24	5.33	Aerobic	Competitor
GCA_003225495	c__Terriglobia	o__Terriglobales	f__SbA1	SPL	Soil	88.02	56.64	4544596	4436	908.17	44	1	27	46	65	25.68	5.93	Aerobic	Competitor
GCA_003225515	c__Terriglobia	o__Terriglobales	f__SbA1	SPL	Soil	85.28	56.54	4546488	4713	845.88	32	0	44	60	68	25.04	5.92	Aerobic	Scarcity
GCA_003225535	c__Terriglobia	o__Terriglobales	f__SbA1	SPL	Soil	86.06	57.12	6833656	7004	847.70	52	2	32	119	94	25.5	5.02	Aerobic	Scarcity
GCA_003225555	c__Terriglobia	o__Terriglobales	f__SbA1	SPL	Soil	86.14	57.97	4424067	4041	943.69	51	1	44	88	49	27.49	5.94	Aerobic	Scarcity
GCA_003225565	c__Terriglobia	o__Terriglobales	f__SbA1	SPL	Soil	84.62	55.54	4159596	4551	788.21	28	0	36	46	71	29.68	5.96	Aerobic	Scarcity
GCA_003225595	c__Terriglobia	o__Terriglobales	f__SbA1	SPL	Soil	83.75	56.48	5049048	5367	792.76	38	2	32	44	67	28.87	6.23	Aerobic	Scarcity
GCA_003225615	c__Terriglobia	o__Terriglobales	f__SbA1	SPL	Soil	84.42	56.58	4025113	4442	799.49	34	1	32	36	65	30.47	5.97	Aerobic	Scarcity
GCA_003225635	c__Terriglobia	o__Terriglobales	f__SbA1	SPL	Soil	81.99	57.56	7256223	7014	867.96	52	2	24	148	74	26.84	5.36	Aerobic	Scarcity
GCA_003225655	c__Terriglobia	o__Terriglobales	f__QIAW01	SPL	Soil	85.07	56.26	4136379	4319	834.62	32	0	20	82	56	25.05	6.17	Aerobic	Scarcity
GCA_003330725	c__Terriglobia	o__Terriglobales	f__Acidobacteriaceae	SPL	Soil	85.54	56.87	7600726	6719	969.86	65	4	64	196	107	22.81	5.22	Aerobic	Ruderal
GCA_003428625	c__Terriglobia	o__Terriglobales	f__Acidobacteriaceae	SPL	Soil	88.74	60.47	5001827	4042	1104.14	32	4	44	95	62	25.48	5.13	Aerobic	Competitor
GCA_003487005	c__Terriglobia	o__Bryobacterales	f__Bryobacteraceae	SPL	Engineered	90.57	64.19	4466556	4339	954.74	35	3	24	97	59	33.7	5.79	Aerobic	Scarcity
GCA_003517725	c__Terriglobia	o__Bryobacterales	f__Bryobacteraceae	SPL	Soil	82.81	62.38	3924870	4015	921.16	31	1	16	78	42	36.23	5.66	Aerobic	Scarcity
GCA_003543335	c__Terriglobia	o__Terriglobales	f__Acidobacteriaceae	SPL	Soil	88.40	60.55	3991881	3281	1076.93	30	2	20	96	52	29.52	5.05	Aerobic	Ruderal
GCA_003858395	c__Terriglobia	o__Terriglobales	f__Acidobacteriaceae	SPL	Soil	86.74	62.55	4475085	3595	1080.54	25	3	16	131	58	22.17	4.32	Aerobic	Competitor
GCA_003945235	c__Terriglobia	o__Terriglobales	f__Acidobacteriaceae	SPL	Soil	87.96	57.25	6152256	5109	1060.65	53	4	28	158	85	22.25	5.47	Aerobic	Competitor
GCA_004115155	c__Terriglobia	o__Terriglobales	f__Acidobacteriaceae	SPL	Soil	88.94	59.82	6139630	5159	1060.08	47	2	44	153	81	20.94	5.15	Aerobic	Competitor
GCA_004123295	c__Terriglobia	o__Terriglobales	f__Acidobacteriaceae	SPL	Soil	88.63	60.50	5116187	4012	1130.89	33	1	40	180	71	25.71	4.95	Aerobic	Competitor
GCA_004217555	c__Terriglobia	o__Terriglobales	f__Acidobacteriaceae	SPL	Soil	85.76	57.10	7445115	6650	962.53	67	2	40	128	72	22.97	5.24	Aerobic	Ruderal
GCA_004298155	c__Terriglobia	o__UBA7540	f__UBA7540	SPL	Freshwater	86.80	56.74	5213467	4457	1016.40	53	3	16	116	42	31.5	5.66	Aerobic	Ruderal
GCA_004299485	c__Terriglobia	o__Terriglobales	f__SCQP01	SPL	Freshwater	92.96	64.61	3303212	3039	1013.09	20	1	4	28	38	27.83	5.19	Aerobic	Competitor
GCA_004339725	c__Terriglobia	o__Terriglobales	f__Acidobacteriaceae	SPL	Engineered	91.37	58.81	4213726	3527	1093.38	31	2	41	132	64	27.04	5.20	Aerobic	Ruderal
GCA_005773685	c__Terriglobia	o__Bryobacterales	f__Bryobacteraceae	SPL	Freshwater	88.71	60.61	6370035	5677	997.37	30	2	8	71	74	22.93	5.73	Aerobic	Scarcity
GCA_005777455	c__Terriglobia	o__Bryobacterales	f__Bryobacteraceae	SPL	Freshwater	93.86	65.35	7753165	6820	1067.24	46	1	40	127	65	25.44	6.00	Aerobic	Scarcity
GCA_005808475	c__Terriglobia	o__Bryobacterales	f__Bryobacteraceae	SPL	Freshwater	93.86	65.34	7910654	6888	1078.88	52	0	40	134	66	25.39	5.83	Aerobic	Scarcity
GCA_005808865	c__Terriglobia	o__Bryobacterales	f__Bryobacteraceae	SPL	Freshwater	88.83	60.60	5705908	5226	973.01	26	0	8	53	72	23.33	5.73	Aerobic	Scarcity
GCA_008000815	c__Terriglobia	o__Terriglobales	f__Acidobacteriaceae	SPL	Host	89.10	57.90	6405582	5225	1093.78	43	1	48	177	80	23.95	4.65	Aerobic	Ruderal
GCA_008682415	c__Terriglobia	o__Terriglobales	f__Acidobacteriaceae	SPL	Soil	87.21	61.86	6654408	5594	1038.23	60	3	26	144	72	25.29	4.64	Aerobic	Competitor
GCA_009691245	c__Terriglobia	o__2_12_FULL_54_10	f__2_12_FULL_54_10	SPL	Freshwater	86.63	59.11	3434154	2964	1002.66	26	0	16	8	18	26.62	6.01	Aerobic	Scarcity
GCA_009697455	c__Terriglobia	o__Bryobacterales	f__Bryobacteraceae	SPL	Freshwater	90.13	60.78	4486427	4073	993.82	25	0	12	49	65	23.89	5.63	Aerobic	Scarcity
GCA_009697495	c__Terriglobia	o__Bryobacterales	f__Bryobacteraceae	SPL	Freshwater	91.67	60.01	2364486	2434	890.40	19	0	4	33	26	28.04	6.22	Aerobic	Scarcity
GCA_009697505	c__Terriglobia	o__Bryobacterales	f__Bryobacteraceae	SPL	Freshwater	91.61	61.16	2557084	2581	907.91	21	0	8	12	42	28.9	6.02	Aerobic	Scarcity
GCA_009765785	c__Terriglobia	o__Terriglobales	f__Acidobacteriaceae	SPL	Soil	87.25	58.64	5479411	4617	1043.39	42	0	60	154	79	21.77	5.75	Aerobic	Competitor
GCA_009765825	c__Terriglobia	o__Terriglobales	f__Acidobacteriaceae	SPL	Soil	86.21	57.54	5896497	4990	1029.84	39	2	73	121	95	22.81	5.59	Aerobic	Competitor
GCA_009765835	c__Terriglobia	o__Terriglobales	f__Acidobacteriaceae	SPL	Soil	88.96	57.23	4715628	3948	1063.63	45	3	24	186	59	22.51	5.07	Aerobic	Competitor
GCA_009765985	c__Terriglobia	o__Terriglobales	f__Acidobacteriaceae	SPL	Soil	88.99	55.52	5437681	4686	1034.41	58	5	48	156	64	26.56	5.17	Aerobic	Competitor
GCA_009766095	c__Terriglobia	o__Terriglobales	f__Acidobacteriaceae	SPL	Soil	87.18	57.21	5054312	4311	1052.35	43	1	65	104	77	21.36	4.76	Aerobic	Competitor
GCA_009766105	c__Terriglobia	o__Terriglobales	f__Acidobacteriaceae	SPL	Soil	87.80	57.27	6436931	5213	1090.93	46	5	50	212	86	20.52	5.31	Aerobic	Ruderal
GCA_009781125	c__Terriglobia	o__Terriglobales	f__Acidobacteriaceae	SPL	Host	88.72	58.32	3095210	2949	929.35	28	2	20	78	32	28.76	6.02	Aerobic	Competitor
GCA_009837045	c__Terriglobia	o__VXMN01	f__VXMN01	SPL	Host	88.35	62.54	3680688	3341	970.51	27	2	32	84	36	32.45	6.35	Aerobic	Scarcity
GCA_009838515	c__Terriglobia	o__Bryobacterales	f__UBA6623	SPL	Host	88.94	63.57	5841024	4654	1116.68	37	4	40	86	36	25.41	6.21	Aerobic	Scarcity
GCA_009839945	c__Terriglobia	o__Bryobacterales	f__UBA6623	SPL	Host	89.52	63.68	5153820	4445	1038.58	34	1	32	83	34	25.7	6.21	Aerobic	Scarcity
GCA_009841055	c__Terriglobia	o__VXMN01	f__VXMN01	SPL	Host	88.03	62.34	4321718	3598	1052.98	34	1	40	95	36	32.22	6.46	Aerobic	Scarcity
GCA_009841225	c__Terriglobia	o__Bryobacterales	f__UBA6623	SPL	Host	89.40	63.64	5743471	4523	1136.48	36	3	46	85	34	25.25	6.21	Aerobic	Scarcity
GCA_009843165	c__Terriglobia	o__Bryobacterales	f__UBA6623	SPL	Host	88.79	63.62	5619358	4758	1048.97	33	4	36	81	36	25.46	6.21	Aerobic	Scarcity
GCA_009845805	c__Terriglobia	o__Bryobacterales	f__UBA6623	SPL	Host	88.97	63.59	5770385	4646	1105.96	35	4	42	86	35	25.43	6.21	Aerobic	Scarcity
GCA_009914315	c__Terriglobia	o__Terriglobales	f__Acidobacteriaceae	SPL	Terrestrial_n	87.96	60.66	4673153	3854	1067.56	31	4	43	93	68	23.51	4.69	Aerobic	Competitor
GCA_010084195	c__Terriglobia	o__Bryobacterales	f__Bryobacteraceae	SPL	Freshwater	91.77	59.39	6512058	6492	922.30	37	5	28	49	77	22.88	5.43	Aerobic	Scarcity
GCA_010093025	c__Terriglobia	o__Terriglobales	f__Acidobacteriaceae	SPL	Host	88.74	57.96	4554841	3785	1068.92	38	1	49	135	58	26.12	5.95	Aerobic	Ruderal
GCA_010365215	c__Terriglobia	o__Acidoferrales	f__UBA7541	SPL	Freshwater	89.96	59.81	4118028	3577	1038.41	27	3	34	33	33	26.64	5.82	Aerobic	Scarcity
GCA_013285705	c__Terriglobia	o__Terriglobales	f__Acidobacteriaceae	SPL	Soil	87.65	55.50	4467222	4004	977.93	37	1	28	101	60	25.11	5.03	Aerobic	Ruderal
GCA_013286165	c__Terriglobia	o__Acidoferrales	f__UBA7541	SPL	Soil	87.72	59.90	2191645	1919	1002.59	25	2	16	38	22	25.66	5.69	Aerobic	Competitor
GCA_013286465	c__Terriglobia	o__Acidoferrales	f__UBA7541	SPL	Soil	88.17	57.57	3014771	2611	1019.10	32	1	16	19	46	25.16	5.53	Aerobic	Competitor

GCA_013287135	c__Terriglobia	o__Acidoferrales	f__UBA7541	SPL	Soil	87.00	54.65	4379895	4215	905.46	39	6	50	34	47	25.22	4.99	Aerobic	Ruderal
GCA_013288805	c__Terriglobia	o__Terriglobales	f__SbA1	SPL	Soil	85.62	56.13	3533399	3505	863.73	19	2	16	28	45	25.8	5.15	Aerobic	Competitor
GCA_013289585	c__Terriglobia	o__Acidoferrales	f__UBA7541	SPL	Soil	88.21	57.54	4730425	4189	997.36	38	0	50	38	50	27.65	5.28	Aerobic	Competitor
GCA_013289665	c__Terriglobia	o__Acidoferrales	f__UBA7541	SPL	Soil	86.94	58.43	4518241	3900	1008.20	37	0	20	48	53	25.19	4.90	Aerobic	Competitor
GCA_013289685	c__Terriglobia	o__Terriglobales	f__SbA1	SPL	Soil	87.00	58.50	6846238	5926	1006.29	50	4	66	96	70	26.94	4.81	Aerobic	Competitor
GCA_013289695	c__Terriglobia	o__Acidoferrales	f__UBA7541	SPL	Soil	85.83	58.84	4515227	3987	972.35	39	1	43	27	51	24.93	5.78	Aerobic	Competitor
GCA_013289715	c__Terriglobia	o__Acidoferrales	f__UBA7541	SPL	Soil	86.12	58.78	4936869	4331	982.66	38	1	28	42	42	24.93	5.69	Aerobic	Competitor
GCA_013289765	c__Terriglobia	o__Terriglobales	f__SbA1	SPL	Soil	87.88	56.12	4805006	4305	981.28	29	0	36	47	60	26.12	5.20	Aerobic	Competitor
GCA_013289795	c__Terriglobia	o__Terriglobales	f__SbA1	SPL	Soil	89.16	57.76	4674986	4275	976.21	45	3	29	28	62	26.32	6.12	Aerobic	Competitor
GCA_013289825	c__Terriglobia	o__Acidoferrales	f__UBA7541	SPL	Soil	88.03	58.21	4531437	3823	1044.74	37	1	24	48	59	25.22	5.64	Aerobic	Competitor
GCA_013289835	c__Terriglobia	o__Acidoferrales	f__UBA7541	SPL	Soil	87.94	57.91	4533591	4141	963.96	40	4	40	35	51	27.38	5.86	Aerobic	Competitor
GCA_013289855	c__Terriglobia	o__Acidoferrales	f__UBA7541	SPL	Soil	87.15	57.90	5348996	4626	1008.79	31	2	58	60	57	25.53	5.37	Aerobic	Competitor
GCA_013289875	c__Terriglobia	o__Terriglobales	f__Acidobacteriaceae	SPL	Soil	89.24	58.56	5050189	4409	1021.94	40	2	52	106	79	27.98	5.06	Aerobic	Competitor
GCA_013289925	c__Terriglobia	o__Terriglobales	f__SbA1	SPL	Soil	85.38	58.42	4238659	3780	957.38	27	1	28	33	65	30.51	5.32	Aerobic	Competitor
GCA_013289955	c__Terriglobia	o__Acidoferrales	f__UBA7541	SPL	Soil	88.40	57.99	6511822	5449	1057.42	57	4	54	79	81	24.69	5.46	Aerobic	Ruderal
GCA_013290025	c__Terriglobia	o__Terriglobales	f__SbA1	SPL	Soil	87.11	56.40	4900674	4893	873.20	38	2	24	48	71	28.3	5.50	Aerobic	Competitor
GCA_013360315	c__Terriglobia	o__Bryobacterales	f__Bryobacteraceae	SPL	Engineered	93.10	59.28	5263547	4835	1015.05	39	4	24	142	60	33.54	5.46	Aerobic	Scarcity
GCA_013361705	c__Terriglobia	o__Terriglobales	f__Acidobacteriaceae	SPL	Soil	89.69	58.29	5168689	4556	1017.56	36	2	40	151	86	24.79	5.37	Aerobic	Ruderal
GCA_013388415	c__Terriglobia	o__Bryobacterales	f__Bryobacteraceae	SPL	Freshwater	92.94	63.72	4353313	4160	973.27	28	0	20	136	50	33.03	6.03	Anaerobic	Scarcity
GCA_013410065	c__Terriglobia	o__Terriglobales	f__Acidobacteriaceae	SPL	Soil	88.93	58.26	4305353	3646	1051.02	33	1	34	107	69	22.41	4.90	Aerobic	Competitor
GCA_013410115	c__Terriglobia	o__Terriglobales	f__Acidobacteriaceae	SPL	Soil	87.04	57.56	5285246	4466	1031.53	50	1	40	97	67	22.7	4.89	Aerobic	Competitor
GCA_013410875	c__Terriglobia	o__Terriglobales	f__Acidobacteriaceae	SPL	Soil	87.28	57.73	5073949	4322	1025.89	47	2	46	106	76	21.79	4.89	Aerobic	Ruderal
GCA_013822245	c__Terriglobia	o__Bryobacterales	f__Bryobacteraceae	SPL	Engineered	91.48	64.46	4468912	4045	1011.26	36	2	22	101	48	30.29	5.80	Aerobic	Competitor
GCA_014083945	c__Terriglobia	o__Terriglobales	f__Acidobacteriaceae	SPL	Terrestrial_n	88.29	61.09	3928701	3308	1049.40	32	1	20	97	66	23.95	5.64	Aerobic	Ruderal
GCA_014201315	c__Terriglobia	o__Terriglobales	f__Acidobacteriaceae	SPL	Soil	87.07	58.41	4880565	4146	1026.70	43	1	40	101	70	22.44	5.14	Aerobic	Competitor
GCA_014201335	c__Terriglobia	o__Terriglobales	f__Acidobacteriaceae	SPL	Soil	86.76	56.41	6178831	5418	991.81	56	5	44	140	89	21.52	5.39	Aerobic	Competitor
GCA_014201365	c__Terriglobia	o__Terriglobales	f__Acidobacteriaceae	SPL	Soil	86.99	57.64	5122604	4341	1030.31	42	2	44	96	66	22.76	4.89	Aerobic	Competitor
GCA_014201375	c__Terriglobia	o__Terriglobales	f__Acidobacteriaceae	SPL	Soil	87.07	58.18	4827422	4178	1009.46	41	0	40	95	75	22.91	4.96	Aerobic	Competitor
GCA_014201455	c__Terriglobia	o__Terriglobales	f__Acidobacteriaceae	SPL	Soil	88.05	58.23	6452904	5343	1064.39	37	4	78	148	95	24.05	4.61	Aerobic	Competitor
GCA_014203225	c__Terriglobia	o__Terriglobales	f__Acidobacteriaceae	SPL	Soil	86.72	57.80	6472662	5186	1083.57	36	3	121	195	97	21.23	4.96	Aerobic	Competitor
GCA_014203275	c__Terriglobia	o__Terriglobales	f__Acidobacteriaceae	SPL	Soil	87.76	58.59	7296843	6246	1026.42	55	4	53	183	89	20.02	5.06	Aerobic	Competitor
GCA_014274465	c__Terriglobia	o__Terriglobales	f__Acidobacteriaceae	SPL	Soil	88.29	55.79	6271114	5483	1011.58	61	1	28	138	87	25.6	5.36	Aerobic	Competitor
GCA_014274685	c__Terriglobia	o__Terriglobales	f__Acidobacteriaceae	SPL	Soil	89.86	55.03	4254614	3610	1060.35	39	3	32	95	57	25.79	5.15	Aerobic	Ruderal
GCA_014379815	c__Terriglobia	o__Bryobacterales	f__Bryobacteraceae	SPL	Soil	93.29	56.99	5112334	5202	917.00	52	0	40	56	65	25.43	6.12	Aerobic	Competitor
GCA_014584705	c__Terriglobia	o__Bryobacterales	f__Bryobacteraceae	SPL	Engineered	89.71	61.44	4877157	4031	1086.73	34	3	24	110	42	30.74	6.16	Aerobic	Competitor
GCA_014640335	c__Terriglobia	o__Terriglobales	f__Acidobacteriaceae	SPL	Soil	90.42	56.93	4535631	3726	1101.75	37	2	43	179	54	23.86	5.16	Aerobic	Competitor
GCA_014640355	c__Terriglobia	o__Terriglobales	f__Acidobacteriaceae	SPL	Soil	88.66	60.50	5114915	4011	1131.22	33	0	40	180	71	25.74	4.95	Aerobic	Competitor
GCA_014642855	c__Terriglobia	o__Terriglobales	f__Acidobacteriaceae	SPL	Soil	89.85	58.44	4196269	3486	1082.74	32	1	42	166	46	24.13	5.48	Aerobic	Competitor
GCA_014860455	c__Terriglobia	o__Bryobacterales	f__Bryobacteraceae	SPL	Engineered	91.94	63.11	5286262	4906	989.46	35	3	37	152	57	28.71	6.03	Aerobic	Scarcity
GCA_015075235	c__Terriglobia	o__Bryobacterales	f__Bryobacteraceae	SPL	Engineered	92.45	64.32	5436390	4907	1027.08	39	2	32	121	55	31.1	5.42	Anaerobic	Ruderal
GCA_015277775	c__Terriglobia	o__Bryobacterales	f__Bryobacteraceae	SPL	Freshwater	90.96	63.05	9532094	7616	1140.63	82	2	32	239	92	26.46	6.04	Aerobic	Ruderal
GCA_015654835	c__Terriglobia	o__Terriglobales	f__Acidobacteriaceae	SPL	Terrestrial_n	86.83	58.37	4710788	4085	1001.10	28	3	32	119	63	25.37	4.84	Aerobic	Competitor
GCA_015655195	c__Terriglobia	o__Terriglobales	f__Acidobacteriaceae	SPL	Terrestrial_n	88.33	59.79	4764362	4022	1045.14	25	2	24	126	59	22.94	4.77	Aerobic	Competitor
GCA_015655315	c__Terriglobia	o__Terriglobales	f__Acidobacteriaceae	SPL	Terrestrial_n	89.34	55.95	5300096	5003	946.46	46	8	49	132	85	24.79	4.89	Aerobic	Ruderal
GCA_015655365	c__Terriglobia	o__Terriglobales	f__Acidobacteriaceae	SPL	Terrestrial_n	85.75	60.39	2421653	2421	855.55	16	0	12	59	41	27.19	5.05	Anaerobic	Competitor
GCA_015655585	c__Terriglobia	o__Terriglobales	f__Acidobacteriaceae	SPL	Terrestrial_n	88.12	59.85	3122958	2763	996.80	30	2	32	59	49	25.6	5.08	Aerobic	Competitor
GCA_016125275	c__Terriglobia	o__Bryobacterales	f__UBA6623	SPL	Freshwater	93.25	67.07	7553324	6050	1165.70	56	4	57	82	58	26.79	6.09	Aerobic	Scarcity
GCA_016178155	c__Terriglobia	o__Bryobacterales	f__Bryobacteraceae	SPL	Freshwater	87.25	64.32	5604239	4877	1002.07	38	0	16	111	61	34.12	6.36	Aerobic	Scarcity
GCA_016178945	c__Terriglobia	o__Bryobacterales	f__Bryobacteraceae	SPL	Freshwater	92.61	64.99	7344081	6831	996.61	72	1	28	86	90	31.34	5.78	Aerobic	Scarcity
GCA_016178975	c__Terriglobia	o__Acidoferrales	f__UBA7541	SPL	Freshwater	88.56	62.82	2186460	1976	977.14	17	2	8	13	32	33.55	6.64	Aerobic	Competitor
GCA_016179005	c__Terriglobia	o__Acidoferrales	f__UBA7541	SPL	Freshwater	89.37	62.06	1633498	1711	851.81	16	0	12	12	24	33.34	6.54	Anaerobic	Scarcity
GCA_016179015	c__Terriglobia	o__Terriglobales	f__SbA1	SPL	Freshwater	87.88	60.55	2231170	2296	853.37	15	1	16	18	47	32.09	5.98	Aerobic	Scarcity
GCA_016185365	c__Terriglobia	o__Terriglobales	f__DSPA01	SPL	Freshwater	88.34	60.99	2384598	2235	944.32	19	0	4	18	45	31.65	6.40	Aerobic	Scarcity
GCA_016185395	c__Terriglobia	o__Terriglobales	f__JACPNR01	SPL	Freshwater	89.93	64.26	2475622	2261	985.20	12	0	20	11	45	32.71	6.14	Aerobic	Scarcity
GCA_016185425	c__Terriglobia	o__Bryobacterales	f__Bryobacteraceae	SPL	Freshwater	94.04	60.63	6643823	5754	1088.27	55	0	12	72	68	27.16	5.83	Aerobic	Ruderal
GCA_016185465	c__Terriglobia	o__Bryobacterales	f__Bryobacteraceae	SPL	Freshwater	93.13	61.04	2335330	2330	935.52	17	0	4	29	35	29.52	6.02	Aerobic	Scarcity
GCA_016189965	c__Terriglobia	o__Acidoferrales	f__2_02_FULL_67_57	SPL	Freshwater	87.94	66.37	1569195	1584	871.15	12	0	0	4	28	55.44	6.20	Aerobic	Scarcity
GCA_016191085	c__Terriglobia	o__2_12_FULL_54_10	f__2_12_FULL_54_10	SPL	Freshwater	88.13	57.11	3470763	3209	952.51	18	3	16	21	21	30.95	5.97	Aerobic	Scarcity
GCA_016191645	c__Terriglobia	o__Bryobacterales	f__Bryobacteraceae	SPL	Freshwater	93.91	59.12	4467732	3895	1075.75	27	2	8	56	48	25.25	6.08	Anaerobic	Scarcity
GCA_016195925	c__Terriglobia	o__Bryobacterales	f__Bryobacteraceae	SPL	Freshwater	93.40	65.30	4659981	4496	969.33	46	1	16	54	57	32.15	5.84	Aerobic	Scarcity
GCA_016196065	c__Terriglobia	o__Terriglobales	f__SbA1	SPL	Freshwater	89.54	57.20	5527405	4822	1027.08	39	2	34	79	79	25.14	5.65	Aerobic	Competitor
GCA_016197095	c__Terriglobia	o__Bryobacterales	f__Bryobacteraceae	SPL	Freshwater	87.98	64.53	5090806	4453	1005.97	26	2	16	95	56	35.14	6.38	Aerobic	Scarcity
GCA_016199675	c__Terriglobia	o__2_12_FULL_54_10	f__2_12_FULL_54_10	SPL	Freshwater	89.16	54.72	2505756	2450	911.15	14	2	20	11	23	32.89	6.14	Aerobic	Competitor
GCA_016201185	c__Terriglobia	o__Bryobacterales	f__JACQDG01	SPL	Freshwater	92.48	63.18	3644830	3710	910.37	20	0	24	57	46	38.35	5.63	Aerobic	Scarcity
GCA_016201195	c__Terriglobia	o__Bryobacterales	f__JACQDG01	SPL	Freshwater	90.84	64.19	5655286	5910	869.36	43	1	28	87	66	34.99	5.72	Aerobic	Scarcity
GCA_016201205	c__Terriglobia	o__Bryobacterales	f__Bryobacteraceae	SPL	Freshwater	92.51	62.58	7002066	6255	1036.96	54	1	20	113	69	31.23	6.36	Anaerobic	Scarcity
GCA_016201215	c__Terriglobia	o__Acidoferrales	f__UBA7541	SPL	Freshwater	87.11	63.62	3294321	3167	904.03	28	1	24	20	37	38.94	5.76	Aerobic	Scarcity
GCA_016201265	c__Terriglobia	o__Terriglobales	f__SbA1	SPL	Freshwater	88.10	60.44	2934454	2940	878.61	28	1	16	32	58	31.57	6.18	Aerobic	Scarcity
GCA_016201285	c__Terriglobia	o__Acidoferrales	f__UBA7541	SPL	Freshwater	87.32	62.45	3667939	3243	984.16	39	3	22	21	40	33.1	6.44	Anaerobic	Scarcity
GCA_016202975	c__Terriglobia	o__UBA7540	f__UBA7540	SPL	Freshwater	86.16	59.30	3467734	3495	853.99	24	0	4	66	35	31.59	5.63	Aerobic	Scarcity
GCA_016210855	c__Terriglobia	o__Acidoferrales	f__2_02_FULL_67_57	SPL	Freshwater	91.56	66.13	1878761	1921	896.05	13	0	4	7	32	51.23	5.81	Aerobic	Scarcity
GCA_016210895	c__Terriglobia	o__Acidoferrales	f__2_02_FULL_67_57	SPL	Freshwater	91.97	63.92	1724835	1867	848.23	15	1	12	9	23	50.9	6.19	Aerobic	Scarcity
GCA_016213245	c__Terriglobia	o__Bryobacterales	f__Bryobacteraceae	SPL	Freshwater	92.93	65.89	3829360	3466	1027.30	34	2	12	100	42	31.09	5.92	Anaerobic	Scarcity
GCA_016213725	c__Terriglobia	o__Bryobacterales	f__Bryobacteraceae	SPL	Freshwater	93.45	64.35	6049182	5220	1083.67	44	3	20	236	58	29.55	6.60	Aerobic	Scarcity
GCA_016213755	c__Terriglobia	o__Bryobacterales	f__Bryobacteraceae	SPL	Freshwater	93.97	66.04	5784026	5182	1050.70	39	0	12	204	61	32.55	5.55	Anaerobic	Scarcity
GCA_016213775	c__Terriglobia	o__Bryobacterales	f__Bryobacteraceae	SPL	Freshwater	92.02	60.62	10956185	9410	1072.09	93	6	32	179	92	26.24	5.55	Anaerobic	Ruderal
GCA_016215215	c__Terriglobia	o__Bryobacterales	f__Bryobacteraceae	SPL	Freshwater	93.19	64.34	4709714	4472	983.52	43	2	28	119	63	30.28	6.04	Aerobic	Ruderal
GCA_016650235	c__Terriglobia	o__Bryobacterales	f__Bryobacteraceae	SPL	Soil	90.89	60.20	6084103	5710	969.15	38	4	17	83	65	25.78	5.72	Aerobic	Scarcity
GCA_016713735	c__Terriglobia	o__Bryobacterales	f__Bryobacteraceae	SPL	Engineered	89.23	64.70	6847183	6439	952.10	41	3	24	124	66	26.9	5.77	Aerobic	Scarcity
GCA_016716385	c__Terriglobia	o__Bryobacterales	f__Bryobacteraceae	SPL	Engineered	93.63	64.89	6412234	5695	1057.04	39	4	34	103	76	32.29	5.55	Aerobic	Scarcity
GCA_016721425	c__Terriglobia	o__Bryobacterales	f__Bryobacteraceae	SPL	Engineered	91.00	63.35	7693013	6939	1012.07	57	3	28	246	71	27.33	5.97	Anaerobic	Scarcity
GCA_016789745	c__Terriglobia	o__Bryobacterales	f__Bryobacteraceae	SPL	Engineered	93.72	62.78	10058487	8678	1087.70	77	7	81	145	87	28.73	5.89	Aerobic	Scarcity
GCA_016789755	c__Terriglobia	o__Bryobacterales	f__Bryobacteraceae	SPL	Engineered	94.47	61.88	8229935	7082	1098.21	62	2	20	165	80	26.77	5.78	Aerobic	Competitor
GCA_016789785	c__Terriglobia	o__Bryobacterales	f__Bryobacteraceae	SPL	Engineered	91.19	60.97	10127521	9272	999.71	93	4	20	158	97	27.57	5.53	Aerobic	Competitor
GCA_016789905	c__Terriglobia	o__Bryobacterales	f__Bryobacteraceae	SPL	Engineered	93.40	61.31	8082235	6929	1090.59	60	2	20	114	73	25.22	5.65	Aerobic	Scarcity
GCA_016789925	c__Terriglobia	o__Bryobacterales	f__Bryobacteraceae	SPL	Engineered	93.29	61.85	6908704	6705	962.25	50	1	28	111	78	29.51	5.89	Aerobic	Scarcity
GCA_016865505	c__Terriglobia	o__Bryobacterales	f__Bryobacteraceae	SPL	Host	94.65	60.08	6281799	5515	1081.59	56	2	57	65	70	27.37	6.06	Aerobic	Ruderal

GCA_016870915	c__Terriglobia	o__Bryobacterales	f__Bryobacteraceae	SPL	Freshwater	92.99	64.05	6612146	5721	1074.93	44	2	16	103	67	26.86	5.66	Aerobic	Scarcity
GCA_016870995	c__Terriglobia	o__Bryobacterales	f__Bryobacteraceae	SPL	Freshwater	93.75	57.48	5723276	5451	986.03	44	0	16	75	64	25.78	6.11	Aerobic	Competitor
GCA_016871015	c__Terriglobia	o__Bryobacterales	f__Bryobacteraceae	SPL	Freshwater	93.84	63.65	8658050	7435	1090.41	47	4	40	164	72	28.26	5.66	Aerobic	Scarcity
GCA_016871055	c__Terriglobia	o__Bryobacterales	f__Bryobacteraceae	SPL	Freshwater	93.22	61.01	7144765	6810	973.78	52	2	12	94	73	24.53	5.83	Aerobic	Scarcity
GCA_016871095	c__Terriglobia	o__Bryobacterales	f__Bryobacteraceae	SPL	Freshwater	91.34	66.87	5928059	5935	911.28	49	6	8	141	54	40.73	5.94	Anaerobic	Scarcity
GCA_016871115	c__Terriglobia	o__Bryobacterales	f__Bryobacteraceae	SPL	Freshwater	90.81	63.49	6327301	6800	844.11	41	0	16	79	79	25.91	5.97	Aerobic	Scarcity
GCA_016871135	c__Terriglobia	o__Bryobacterales	f__Bryobacteraceae	SPL	Freshwater	94.67	60.99	6464961	6634	922.91	44	2	16	71	81	24.89	6.03	Aerobic	Scarcity
GCA_016871155	c__Terriglobia	o__Bryobacterales	f__Bryobacteraceae	SPL	Freshwater	94.18	62.04	7222881	6389	1064.61	55	3	20	107	58	24.94	6.15	Aerobic	Scarcity
GCA_016871215	c__Terriglobia	o__VXMN01	f__VXMN01	SPL	Freshwater	84.33	57.93	5425955	5034	903.51	43	2	16	158	46	27.51	6.57	Aerobic	Scarcity
GCA_016871315	c__Terriglobia	o__Bryobacterales	f__Bryobacteraceae	SPL	Freshwater	93.64	62.32	6247865	5558	1054.34	39	1	40	82	65	29.18	5.57	Aerobic	Scarcity
GCA_016880505	c__Terriglobia	o__Bryobacterales	f__Bryobacteraceae	SPL	Freshwater	92.49	62.19	5740825	6019	881.51	50	0	24	177	76	33.76	5.67	Aerobic	Scarcity
GCA_017304135	c__Terriglobia	o__Bryobacterales	f__Bryobacteraceae	SPL	Engineered	92.83	64.53	6493284	5853	1031.58	56	1	24	131	56	27.3	5.58	Aerobic	Scarcity
GCA_017307395	c__Terriglobia	o__Terriglobales	f__Acidobacteriaceae	SPL	Engineered	88.83	58.91	4873249	4322	1001.43	46	7	24	133	48	24.66	5.71	Aerobic	Competitor
GCA_017307505	c__Terriglobia	o__Bryobacterales	f__Bryobacteraceae	SPL	Engineered	91.31	62.44	9385101	7737	1108.34	85	3	32	242	80	27.68	6.15	Aerobic	Competitor
GCA_017883345	c__Terriglobia	o__Bryobacterales	f__Bryobacteraceae	SPL	Soil	88.80	63.34	5447107	5411	892.53	38	1	16	180	80	29.57	5.49	Anaerobic	Competitor
GCA_017883355	c__Terriglobia	o__Bryobacterales	f__Bryobacteraceae	SPL	Soil	91.46	63.33	6166549	5910	953.65	44	0	12	207	106	26.64	5.34	Aerobic	Competitor
GCA_017883385	c__Terriglobia	o__Terriglobales	f__SbA1	SPL	Soil	86.88	58.62	6800148	5943	993.08	65	0	40	148	80	25.18	5.01	Aerobic	Competitor
GCA_017883405	c__Terriglobia	o__Terriglobales	f__Acidobacteriaceae	SPL	Soil	89.12	57.00	3836886	3498	977.67	37	0	20	124	52	26.66	4.98	Aerobic	Competitor
GCA_017883415	c__Terriglobia	o__Bryobacterales	f__Bryobacteraceae	SPL	Soil	90.45	64.87	5842053	5594	945.00	44	0	28	89	78	31.12	5.68	Aerobic	Competitor
GCA_017883425	c__Terriglobia	o__Terriglobales	f__SbA1	SPL	Soil	85.70	59.13	5630780	5130	939.34	37	0	48	107	67	26.79	5.37	Aerobic	Competitor
GCA_018268715	c__Terriglobia	o__Bryobacterales	f__Bryobacteraceae	SPL	Engineered	93.30	64.49	7631511	6379	1118.46	74	2	32	212	94	28.59	5.78	Aerobic	Competitor
GCA_018268755	c__Terriglobia	o__Terriglobales	f__SbA1	SPL	Engineered	89.24	59.71	5364654	4489	1068.07	34	2	42	127	74	28.92	5.52	Aerobic	Competitor
GCA_018268775	c__Terriglobia	o__Terriglobales	f__Acidobacteriaceae	SPL	Engineered	88.52	60.13	3985596	3460	1026.20	28	3	28	75	63	26.32	5.88	Aerobic	Competitor
GCA_018268785	c__Terriglobia	o__Acidoferrales	f__UBA7541	SPL	Engineered	90.12	56.03	4236996	3958	964.98	33	2	38	48	53	25.65	5.75	Aerobic	Scarcity
GCA_018268815	c__Terriglobia	o__Acidoferrales	f__UBA7541	SPL	Engineered	89.37	56.01	4118987	3736	998.87	35	1	20	46	54	25.03	5.65	Aerobic	Competitor
GCA_018268835	c__Terriglobia	o__Bryobacterales	f__Bryobacteraceae	SPL	Engineered	93.00	65.40	7460912	6409	1083.04	56	3	16	212	95	29.37	6.03	Aerobic	Competitor
GCA_018268855	c__Terriglobia	o__Terriglobales	f__Acidobacteriaceae	SPL	Engineered	91.39	56.63	3524089	3117	1034.23	26	2	16	105	40	27.51	4.40	Aerobic	Competitor
GCA_018268875	c__Terriglobia	o__Bryobacterales	f__Bryobacteraceae	SPL	Engineered	94.43	61.24	5033196	5109	930.91	36	2	28	53	76	28.66	6.18	Aerobic	Scarcity
GCA_018268895	c__Terriglobia	o__Terriglobales	f__Acidobacteriaceae	SPL	Engineered	89.37	59.09	4296351	3579	1073.49	30	4	55	94	59	25.74	5.19	Aerobic	Competitor
GCA_018268915	c__Terriglobia	o__Terriglobales	f__Acidobacteriaceae	SPL	Engineered	89.23	57.42	5908015	5008	1053.61	46	2	35	160	58	25.04	6.18	Aerobic	Competitor
GCA_018268935	c__Terriglobia	o__Bryobacterales	f__Bryobacteraceae	SPL	Engineered	94.60	60.62	8991788	7509	1137.95	69	3	20	168	76	26.22	5.74	Anaerobic	Ruderal
GCA_018449545	c__Terriglobia	o__Terriglobales	f__Acidobacteriaceae	SPL	Terrestrial_n	87.08	57.65	6151722	5092	1052.99	53	2	40	137	75	21.48	5.09	Aerobic	Competitor
GCA_018449715	c__Terriglobia	o__Terriglobales	f__Acidobacteriaceae	SPL	Terrestrial_n	88.67	58.83	4923323	4139	1055.89	43	2	38	81	61	24.23	4.92	Aerobic	Ruderal
GCA_019187195	c__Terriglobia	o__Bryobacterales	f__Bryobacteraceae	SPL	Engineered	93.78	62.18	5828256	4925	1118.40	40	1	8	120	60	32.71	5.46	Anaerobic	Competitor
GCA_019232935	c__Terriglobia	o__Terriglobales	f__Acidobacteriaceae	SPL	Host	91.05	59.53	3897976	3700	957.96	36	1	39	73	66	29.37	5.34	Aerobic	Competitor
GCA_019233375	c__Terriglobia	o__Bryobacterales	f__Bryobacteraceae	SPL	Host	92.60	57.87	4786858	4609	960.84	41	1	40	83	54	28.61	5.74	Aerobic	Competitor
GCA_019233755	c__Terriglobia	o__Bryobacterales	f__Bryobacteraceae	SPL	Host	91.41	57.82	4947278	4396	1030.56	41	0	24	95	49	30.55	5.96	Aerobic	Ruderal
GCA_019234945	c__Terriglobia	o__Terriglobales	f__Acidobacteriaceae	SPL	Host	89.63	59.27	3500327	3188	984.23	31	0	24	77	54	29.51	4.86	Aerobic	Competitor
GCA_019236695	c__Terriglobia	o__Terriglobales	f__JAFAIN01	SPL	Soil	88.91	61.54	3639103	3393	953.21	27	1	16	37	52	30.04	5.56	Aerobic	Scarcity
GCA_019237345	c__Terriglobia	o__Bryobacterales	f__Bryobacteraceae	SPL	Soil	91.61	57.90	5918907	5881	921.77	49	3	60	91	60	29.32	5.65	Aerobic	Competitor
GCA_019237515	c__Terriglobia	o__Terriglobales	f__SbA1	SPL	Soil	90.35	55.19	4198576	4299	881.49	32	1	28	44	74	29.77	5.94	Aerobic	Competitor
GCA_019239585	c__Terriglobia	o__Bryobacterales	f__Bryobacteraceae	SPL	Host	91.74	54.40	3569695	3836	852.37	25	1	24	69	57	27.08	5.90	Aerobic	Competitor
GCA_019239815	c__Terriglobia	o__Acidoferrales	f__UBA7541	SPL	Host	87.99	57.89	4590517	4639	868.79	39	2	20	65	74	25.93	5.22	Aerobic	Competitor
GCA_019240405	c__Terriglobia	o__Bryobacterales	f__Bryobacteraceae	SPL	Host	93.42	55.46	4447216	3931	1059.40	33	1	20	75	57	27.54	5.85	Aerobic	Competitor
GCA_019240475	c__Terriglobia	o__Bryobacterales	f__Bryobacteraceae	SPL	Host	92.95	59.22	5055712	4385	1074.01	32	3	76	77	62	26.36	6.22	Aerobic	Competitor
GCA_019240515	c__Terriglobia	o__Bryobacterales	f__Bryobacteraceae	SPL	Host	92.46	55.85	5053526	5008	933.12	50	1	28	96	61	26.27	5.57	Aerobic	Competitor
GCA_019241535	c__Terriglobia	o__Terriglobales	f__SbA1	SPL	Host	86.56	57.82	4945828	4490	951.90	43	0	53	94	66	31.74	5.86	Aerobic	Scarcity
GCA_019241735	c__Terriglobia	o__Terriglobales	f__SbA1	SPL	Host	90.55	56.09	2618251	2730	866.46	18	1	16	29	43	27.07	5.97	Aerobic	Scarcity
GCA_019242155	c__Terriglobia	o__Bryobacterales	f__Bryobacteraceae	SPL	Host	92.07	59.08	5769545	5576	950.90	39	4	24	33	69	28.63	5.77	Aerobic	Scarcity
GCA_019242685	c__Terriglobia	o__Bryobacterales	f__Bryobacteraceae	SPL	Host	90.62	56.49	5867892	6311	841.24	51	1	44	99	72	28.12	5.51	Aerobic	Scarcity
GCA_019242875	c__Terriglobia	o__Bryobacterales	f__Bryobacteraceae	SPL	Host	90.14	58.76	7248162	7136	915.80	52	3	76	36	86	29.37	6.07	Aerobic	Scarcity
GCA_019243205	c__Terriglobia	o__Bryobacterales	f__Bryobacteraceae	SPL	Host	89.31	56.70	7798255	7965	874.67	65	1	53	155	96	26.39	5.51	Aerobic	Competitor
GCA_019243475	c__Terriglobia	o__Terriglobales	f__Gp1_AA112	SPL	Host	88.60	57.19	4178231	4043	913.34	30	4	20	66	64	27.8	5.50	Aerobic	Competitor
GCA_019244035	c__Terriglobia	o__Bryobacterales	f__Bryobacteraceae	SPL	Terrestrial_n	91.84	54.62	6519002	6912	865.61	61	1	72	140	83	26.54	5.96	Aerobic	Competitor
GCA_019244045	c__Terriglobia	o__Terriglobales	f__SbA1	SPL	Terrestrial_n	82.84	56.73	6357630	6171	851.38	48	3	60	64	65	29.37	5.82	Aerobic	Competitor
GCA_019244055	c__Terriglobia	o__Bryobacterales	f__Bryobacteraceae	SPL	Terrestrial_n	90.61	56.55	6205550	5966	943.91	49	2	32	103	71	27.98	5.72	Aerobic	Competitor
GCA_019244135	c__Terriglobia	o__Terriglobales	f__Gp1_AA112	SPL	Terrestrial_n	88.97	57.21	4193548	4171	893.58	27	1	32	72	64	28	5.76	Aerobic	Competitor
GCA_019244945	c__Terriglobia	o__Bryobacterales	f__Bryobacteraceae	SPL	Terrestrial_n	92.41	55.73	3548398	3626	902.58	31	0	20	75	47	26.13	5.57	Aerobic	Competitor
GCA_019245125	c__Terriglobia	o__Bryobacterales	f__Bryobacteraceae	SPL	Terrestrial_n	93.30	55.48	4616006	4145	1041.05	34	2	28	75	55	27.42	5.85	Aerobic	Competitor
GCA_019245255	c__Terriglobia	o__Bryobacterales	f__Bryobacteraceae	SPL	Terrestrial_n	92.41	56.87	5014570	5219	887.97	41	1	49	107	48	26.78	5.96	Aerobic	Competitor
GCA_019245295	c__Terriglobia	o__Terriglobales	f__Gp1_AA112	SPL	Terrestrial_n	88.77	57.22	4107158	4184	869.93	36	0	24	73	70	28.68	5.63	Aerobic	Competitor
GCA_019246575	c__Terriglobia	o__Bryobacterales	f__Bryobacteraceae	SPL	Terrestrial_n	92.19	57.41	4890538	5102	882.82	37	3	28	121	64	28.45	5.85	Aerobic	Scarcity
GCA_019247145	c__Terriglobia	o__Terriglobales	f__SbA1	SPL	Terrestrial_n	86.02	57.72	4674253	4566	879.66	37	0	49	97	70	31.13	5.88	Aerobic	Scarcity
GCA_019247235	c__Terriglobia	o__Terriglobales	f__SbA1	SPL	Terrestrial_n	86.52	55.79	4543754	4759	824.02	45	3	44	100	60	28.67	6.04	Aerobic	Ruderal
GCA_019261985	c__Terriglobia	o__Bryobacterales	f__Bryobacteraceae	SPL	Terrestrial_n	90.72	58.79	6487584	5941	992.50	45	0	32	30	72	29.5	5.75	Aerobic	Scarcity
GCA_019351915	c__Terriglobia	o__Terriglobales	f__Acidobacteriaceae	SPL	Soil	90.62	58.28	4136597	3402	1102.50	34	1	45	141	51	26.39	5.49	Aerobic	Competitor
GCA_019351975	c__Terriglobia	o__Terriglobales	f__Acidobacteriaceae	SPL	Soil	88.97	59.47	3900059	3406	1020.75	30	2	28	88	57	22.58	5.20	Aerobic	Competitor
GCA_019352005	c__Terriglobia	o__Terriglobales	f__Acidobacteriaceae	SPL	Soil	91.35	59.33	3638869	3058	1088.06	28	1	18	126	59	27.21	5.13	Aerobic	Ruderal
GCA_019510535	c__Terriglobia	o__Terriglobales	f__Acidobacteriaceae	SPL	Soil	89.11	58.84	4268830	4170	911.58	38	3	40	111	81	25.22	5.10	Aerobic	Competitor
GCA_019686145	c__Terriglobia	o__Bryobacterales	f__Bryobacteraceae	SPL	Terrestrial_n	93.36	59.92	3773584	3804	923.95	42	2	16	64	60	41.75	5.84	Aerobic	Scarcity
GCA_019689715	c__Terriglobia	o__Terriglobales	f__Acidobacteriaceae	SPL	Terrestrial_n	90.45	53.59	4373206	3997	989.46	35	23	26	136	47	34.92	4.71	Aerobic	Competitor
GCA_019748195	c__Terriglobia	o__Bryobacterales	f__Bryobacteraceae	SPL	Engineered	92.74	63.59	5832045	5301	1022.04	52	9	28	41	72	32	5.60	Aerobic	Competitor
GCA_019752335	c__Terriglobia	o__Bryobacterales	f__Bryobacteraceae	SPL	Engineered	92.52	62.18	7198979	6319	1056.15	62	3	28	67	84	25.59	6.06	Aerobic	Competitor
GCA_019931185	c__Terriglobia	o__Bryobacterales	f__Bryobacteraceae	SPL	Host	93.23	60.89	5053647	4491	1048.87	42	3	16	61	51	28.78	6.11	Aerobic	Scarcity
GCA_019931245	c__Terriglobia	o__Bryobacterales	f__Bryobacteraceae	SPL	Host	92.61	64.93	6067278	5163	1089.41	37	1	36	73	61	29.45	5.91	Aerobic	Scarcity
GCA_020072585	c__Terriglobia	o__Terriglobales	f__Gp1_AA117	SPL	Freshwater	87.37	55.40	6908691	6010	1005.30	57	4	173	24	72	26.1	5.95	Aerobic	Competitor
GCA_020072645	c__Terriglobia	o__Terriglobales	f__Gp1_AA117	SPL	Freshwater	87.13	54.97	6435735	5771	973.21	58	5	141	30	73	27.2	5.62	Aerobic	Scarcity
GCA_020072655	c__Terriglobia	o__UBA7540	f__UBA7540	SPL	Freshwater	85.83	61.25	4358077	3994	936.92	37	4	8	93	37	37.11	5.64	Anaerobic	Scarcity
GCA_020072765	c__Terriglobia	o__Acidoferrales	f__UBA7541	SPL	Freshwater	86.86	63.21	3780957	3338	983.99	26	1	29	20	41	29.73	5.66	Aerobic	Competitor
GCA_020072785	c__Terriglobia	o__Acidoferrales	f__UBA7541	SPL	Freshwater	88.50	63.63	3729688	3186	1036.97	24	3	28	17	43	32.55	5.98	Anaerobic	Competitor
GCA_020072805	c__Terriglobia	o__Acidoferrales	f__UBA7541	SPL	Freshwater	87.78	60.96	3975944	3522	991.99	34	5	44	21	43	28.19	5.35	Anaerobic	Scarcity
GCA_020072815	c__Terriglobia	o__Terriglobales	f__Gp1_AA117	SPL	Freshwater	86.15	59.03	5293082	4643	983.14	35	3	42	18	64	28.01	5.93	Aerobic	Scarcity
GCA_020072845	c__Terriglobia	o__Terriglobales	f__JAIQGF01	SPL	Freshwater	87.25	61.93	4230756	3763	981.72	29	1	28	23	55	32.6	5.71	Aerobic	Scarcity
GCA_020072905	c__Terriglobia	o__UBA7540	f__UBA7540	SPL	Freshwater	86.40	61.48	4754334	4587	894.99	42	2	4	98	41	33.45	6.51	Aerobic	Scarcity
GCA_020072925	c__Terriglobia	o__UBA7540	f__UBA7540	SPL	Freshwater	86.08	59.97	6497946	5787	965.31	52	1	16	197	51	33.88	5.61	Aerobic	Scarcity
GCA_020072965	c__Terriglobia	o__JAIQFV01	f__JAIQFV01	SPL	Freshwater	83.18	55.73	5917101	4955	995.76	44	2	16	39	49	29.5	6.16	Aerobic	Competitor
GCA_020073015	c__Terriglobia	o__Terriglobales	f__SbA1	SPL	Freshwater	86.88	59.33	5500238	5053	945.77	36	2	28	60	82	29.34	6.24	Anaerobic	Competitor

GCA_020073085	c__Terriglobia	o__JAIQFV01	f__JAIQFV01	SPL	Freshwater	83.65	56.41	5791051	4784	1012.34	60	1	12	32	57	29.87	6.25	Anaerobic	Competitor
GCA_020073105	c__Terriglobia	o__Terriglobales	f__SbA1	SPL	Freshwater	87.66	58.45	5140075	4952	909.25	35	0	37	37	74	28.62	5.54	Aerobic	Competitor
GCA_020073115	c__Terriglobia	o__Bryobacterales	f__Bryobacteraceae	SPL	Freshwater	89.66	64.59	7189069	6731	960.89	49	4	28	236	84	31.23	5.99	Aerobic	Scarcity
GCA_020073145	c__Terriglobia	o__Bryobacterales	f__Bryobacteraceae	SPL	Freshwater	91.13	63.30	6321548	5806	993.50	45	3	12	61	75	27.58	5.79	Aerobic	Ruderal
GCA_020073165	c__Terriglobia	o__Terriglobales	f__SbA1	SPL	Freshwater	88.42	60.21	4596788	4098	992.44	35	1	30	27	60	30.06	6.17	Aerobic	Competitor
GCA_020073185	c__Terriglobia	o__Terriglobales	f__JAIQGF01	SPL	Freshwater	87.13	61.54	4659711	4217	962.82	36	5	68	24	63	32.82	5.43	Aerobic	Scarcity
GCA_020073215	c__Terriglobia	o__Terriglobales	f__SbA1	SPL	Freshwater	86.72	58.82	5616618	4959	982.81	39	3	38	51	71	29.28	5.75	Aerobic	Competitor
GCA_020073265	c__Terriglobia	o__Bryobacterales	f__Bryobacteraceae	SPL	Freshwater	91.68	62.22	6410776	5814	1010.46	48	0	51	29	87	27.86	5.50	Aerobic	Scarcity
GCA_020073285	c__Terriglobia	o__Terriglobales	f__JAIQGF01	SPL	Freshwater	86.82	61.72	4631376	4375	919.65	33	1	24	34	67	32.28	5.24	Aerobic	Competitor
GCA_020073305	c__Terriglobia	o__Bryobacterales	f__Bryobacteraceae	SPL	Freshwater	92.09	60.70	6975661	6239	1031.59	56	3	38	154	87	28.89	5.27	Aerobic	Competitor
GCA_020073325	c__Terriglobia	o__Bryobacterales	f__Bryobacteraceae	SPL	Freshwater	92.69	61.50	6542020	6174	983.24	43	1	85	45	115	26.45	5.46	Aerobic	Ruderal
GCA_020073345	c__Terriglobia	o__Bryobacterales	f__Bryobacteraceae	SPL	Freshwater	91.85	60.08	6230231	5781	991.76	44	0	24	35	59	27.02	5.53	Aerobic	Competitor
GCA_020073365	c__Terriglobia	o__Bryobacterales	f__Bryobacteraceae	SPL	Freshwater	87.71	61.74	12094560	10817	981.27	89	7	52	258	110	26.45	5.17	Aerobic	Ruderal
GCA_020073375	c__Terriglobia	o__Acidoferrales	f__UBA7541	SPL	Freshwater	85.89	59.53	5286591	4624	982.37	32	3	37	29	49	24.05	5.71	Aerobic	Scarcity
GCA_020073385	c__Terriglobia	o__Terriglobales	f__SbA1	SPL	Freshwater	86.44	57.27	8232863	7295	976.06	75	1	66	149	79	26.36	4.82	Aerobic	Competitor
GCA_020073395	c__Terriglobia	o__Terriglobales	f__SbA1	SPL	Freshwater	86.86	58.56	5542370	5108	942.32	46	1	30	98	82	28.78	5.50	Aerobic	Scarcity
GCA_020073445	c__Terriglobia	o__Bryobacterales	f__Bryobacteraceae	SPL	Freshwater	91.71	59.83	6579597	6093	992.27	48	4	20	36	53	26.58	5.57	Aerobic	Competitor
GCA_020073465	c__Terriglobia	o__Terriglobales	f__SbA1	SPL	Freshwater	87.45	58.98	5911990	5206	994.35	46	2	42	92	64	28.31	4.74	Anaerobic	Scarcity
GCA_020073485	c__Terriglobia	o__Acidoferrales	f__UBA7541	SPL	Freshwater	86.47	59.05	5048047	4341	1006.06	33	5	30	23	40	24.68	5.29	Aerobic	Scarcity
GCA_020073495	c__Terriglobia	o__Terriglobales	f__JAIQFD01	SPL	Freshwater	87.90	63.05	3697974	3291	988.56	22	1	20	13	50	33.96	5.84	Anaerobic	Competitor
GCA_020073525	c__Terriglobia	o__Terriglobales	f__SbA1	SPL	Freshwater	87.68	57.92	6019296	5452	968.64	47	2	38	82	80	27.42	5.01	Aerobic	Competitor
GCA_020073545	c__Terriglobia	o__UBA7540	f__UBA7540	SPL	Freshwater	82.78	60.15	5281819	4835	904.28	52	4	25	100	40	33.57	5.46	Anaerobic	Scarcity
GCA_020073565	c__Terriglobia	o__Terriglobales	f__SbA1	SPL	Freshwater	87.70	58.74	6096585	5398	991.35	42	5	36	88	73	28.11	4.91	Aerobic	Competitor
GCA_020073585	c__Terriglobia	o__Acidoferrales	f__UBA7541	SPL	Freshwater	88.25	64.13	4029945	3555	1001.23	26	6	28	29	47	32.65	6.01	Aerobic	Ruderal
GCA_020073605	c__Terriglobia	o__Acidoferrales	f__UBA7541	SPL	Freshwater	89.01	61.27	3660007	3262	999.33	25	3	29	16	37	28.29	5.55	Aerobic	Scarcity
GCA_020073615	c__Terriglobia	o__Bryobacterales	f__Bryobacteraceae	SPL	Freshwater	89.55	64.50	6959529	6331	984.19	43	1	38	86	84	29.54	5.53	Aerobic	Ruderal
GCA_020073645	c__Terriglobia	o__Terriglobales	f__SbA1	SPL	Freshwater	87.85	60.16	4431635	4028	966.72	37	1	28	34	65	31.61	6.61	Anaerobic	Scarcity
GCA_020073665	c__Terriglobia	o__Terriglobales	f__SbA1	SPL	Freshwater	88.15	59.47	5422947	4796	997.73	46	3	48	28	62	29	6.45	Aerobic	Ruderal
GCA_020073705	c__Terriglobia	o__Acidoferrales	f__UBA7541	SPL	Freshwater	86.83	57.88	3786499	3454	952.79	28	3	24	18	29	31.27	6.07	Anaerobic	Competitor
GCA_020247045	c__Terriglobia	o__Bryobacterales	f__Bryobacteraceae	SPL	Host	93.48	65.20	5521032	4701	1099.13	29	0	28	63	58	29.92	6.02	Aerobic	Scarcity
GCA_020247055	c__Terriglobia	o__Bryobacterales	f__Bryobacteraceae	SPL	Host	92.21	64.46	6559110	5716	1059.66	44	11	48	79	63	29.03	6.45	Aerobic	Scarcity
GCA_020723085	c__Terriglobia	o__Bryobacterales	f__Bryobacteraceae	SPL	Terrestrial_n	91.63	66.38	4193760	3730	1031.53	35	1	18	121	42	41.73	5.93	Anaerobic	Scarcity
GCA_020846385	c__Terriglobia	o__Bryobacterales	f__Bryobacteraceae	SPL	Engineered	94.37	62.21	6049883	5060	1128.18	38	1	8	116	62	32.79	5.46	Anaerobic	Competitor
GCA_020846745	c__Terriglobia	o__Bryobacterales	f__Bryobacteraceae	SPL	Engineered	92.18	60.26	7828858	6750	1070.80	51	2	43	158	65	29.98	5.49	Aerobic	Scarcity
GCA_020847045	c__Terriglobia	o__Bryobacterales	f__Bryobacteraceae	SPL	Engineered	94.24	64.47	4799256	4114	1101.11	29	1	8	101	47	27.77	5.90	Aerobic	Scarcity
GCA_020847315	c__Terriglobia	o__Bryobacterales	f__Bryobacteraceae	SPL	Engineered	89.83	61.48	4753134	4167	1022.46	32	4	32	105	47	30.88	6.31	Aerobic	Scarcity
GCA_020847895	c__Terriglobia	o__Bryobacterales	f__Bryobacteraceae	SPL	Engineered	93.24	61.63	7003474	6668	979.37	48	5	36	140	96	32.02	5.26	Aerobic	Competitor
GCA_020847975	c__Terriglobia	o__Bryobacterales	f__Bryobacteraceae	SPL	Engineered	92.58	61.48	8672372	7685	1045.71	57	7	33	158	85	30.6	5.82	Aerobic	Ruderal
GCA_020851555	c__Terriglobia	o__Bryobacterales	f__Bryobacteraceae	SPL	Engineered	92.50	65.69	3689606	3244	1051.85	25	0	4	78	31	37.87	5.47	Anaerobic	Scarcity
GCA_020851655	c__Terriglobia	o__Bryobacterales	f__Bryobacteraceae	SPL	Engineered	94.82	62.12	5734486	5059	1074.75	45	3	12	157	58	31.27	5.71	Aerobic	Scarcity
GCA_020852175	c__Terriglobia	o__Bryobacterales	f__Bryobacteraceae	SPL	Engineered	94.06	64.17	4998167	4262	1103.62	36	3	8	105	48	27.81	5.75	Aerobic	Scarcity
GCA_020852755	c__Terriglobia	o__Bryobacterales	f__Bryobacteraceae	SPL	Engineered	92.64	66.21	7437602	6353	1086.18	46	4	40	233	77	38.44	5.71	Aerobic	Scarcity
GCA_020853155	c__Terriglobia	o__Bryobacterales	f__Bryobacteraceae	SPL	Engineered	89.85	61.46	4300626	4018	960.39	35	1	20	93	45	30.73	6.28	Anaerobic	Scarcity
GCA_020854055	c__Terriglobia	o__Bryobacterales	f__Bryobacteraceae	SPL	Engineered	93.78	61.51	4960235	4332	1076.02	24	3	32	191	49	33.19	5.40	Anaerobic	Scarcity
GCA_021323595	c__Terriglobia	o__Terriglobales	f__SCQP01	SPL	Soil	90.01	67.61	3550904	3040	1051.63	18	3	8	61	43	35.6	6.06	Aerobic	Competitor
GCA_021323655	c__Terriglobia	o__Terriglobales	f__Acidobacteriaceae	SPL	Soil	89.83	56.38	4590232	3988	1034.59	37	7	38	121	51	30.56	4.87	Aerobic	Competitor
GCA_021323935	c__Terriglobia	o__Terriglobales	f__JAFAIN01	SPL	Soil	90.21	62.47	3396652	3276	935.13	17	5	16	31	56	30.27	5.39	Aerobic	Competitor
GCA_021324015	c__Terriglobia	o__Bryobacterales	f__Bryobacteraceae	SPL	Soil	92.98	64.35	8228586	7036	1088.99	53	9	27	183	80	34.17	6.03	Anaerobic	Scarcity
GCA_021324185	c__Terriglobia	o__Bryobacterales	f__Bryobacteraceae	SPL	Soil	93.79	57.37	4703675	4241	1042.60	28	1	12	101	53	32.21	5.89	Aerobic	Competitor
GCA_021324365	c__Terriglobia	o__Terriglobales	f__JAJPJE01	SPL	Soil	88.38	55.74	4444665	4158	945.14	37	3	20	75	56	30.98	5.81	Aerobic	Competitor
GCA_021324615	c__Terriglobia	o__Bryobacterales	f__Bryobacteraceae	SPL	Soil	94.03	63.63	3936149	3579	1035.49	28	1	16	33	66	34.7	5.44	Aerobic	Scarcity
GCA_021324935	c__Terriglobia	o__Acidoferrales	f__UBA7541	SPL	Soil	88.18	59.01	4501011	3959	1003.34	34	1	34	37	56	28.04	4.98	Aerobic	Scarcity
GCA_021325035	c__Terriglobia	o__Terriglobales	f__SbA1	SPL	Soil	90.64	53.41	3864199	3702	945.16	38	0	24	39	48	27.11	5.94	Aerobic	Competitor
GCA_021325175	c__Terriglobia	o__Bryobacterales	f__Bryobacteraceae	SPL	Soil	93.94	61.17	4261054	3905	1026.33	36	2	16	38	66	32.59	4.89	Aerobic	Scarcity
GCA_021325235	c__Terriglobia	o__Bryobacterales	f__Bryobacteraceae	SPL	Soil	93.60	59.80	5033782	4520	1044.66	56	2	16	111	49	29.61	6.43	Aerobic	Competitor
GCA_021325275	c__Terriglobia	o__JAJPHL01	f__JAJPHL01	SPL	Soil	92.72	60.39	6857328	5701	1115.58	42	4	57	90	60	24.23	5.73	Aerobic	Ruderal
GCA_021372245	c__Terriglobia	o__Bryobacterales	f__Bryobacteraceae	SPL	Engineered	93.08	61.73	6358668	5689	1041.84	36	6	20	286	59	34.82	6.23	Anaerobic	Scarcity
GCA_021778155	c__Terriglobia	o__Terriglobales	f__Acidobacteriaceae	SPL	Engineered	88.37	59.30	7507974	7153	927.04	73	6	40	220	86	25.95	5.44	Aerobic	Ruderal
GCA_021778315	c__Terriglobia	o__Terriglobales	f__Acidobacteriaceae	SPL	Engineered	88.87	60.87	1702292	1459	1036.12	12	1	8	36	34	23.13	6.01	Aerobic	Competitor
GCA_021778335	c__Terriglobia	o__Bryobacterales	f__Bryobacteraceae	SPL	Engineered	91.87	63.40	5541366	5221	975.67	47	1	16	131	71	29.39	6.04	Aerobic	Ruderal
GCA_021778525	c__Terriglobia	o__Terriglobales	f__Acidobacteriaceae	SPL	Engineered	92.15	60.33	2637871	2466	985.52	20	2	20	75	52	28.26	5.35	Aerobic	Ruderal
GCA_021778815	c__Terriglobia	o__Terriglobales	f__Acidobacteriaceae	SPL	Engineered	89.89	56.96	3262424	3026	966.83	26	1	36	93	52	29.22	6.41	Aerobic	Competitor
GCA_021778845	c__Terriglobia	o__Terriglobales	f__Acidobacteriaceae	SPL	Engineered	88.32	60.20	3769750	3147	1058.13	25	2	26	101	55	23.17	5.44	Aerobic	Competitor
GCA_021778875	c__Terriglobia	o__Terriglobales	f__Acidobacteriaceae	SPL	Engineered	89.10	62.70	4077127	4159	871.77	33	3	28	151	81	27.76	5.02	Aerobic	Competitor
GCA_021779695	c__Terriglobia	o__Terriglobales	f__Acidobacteriaceae	SPL	Engineered	88.31	58.62	4494199	4281	926.31	41	3	44	100	37	26.69	4.57	Aerobic	Ruderal
GCA_021779935	c__Terriglobia	o__Terriglobales	f__Koribacteraceae	SPL	Engineered	87.60	57.99	4491725	4012	980.32	33	3	29	54	67	31.99	4.82	Anaerobic	Competitor
GCA_021780055	c__Terriglobia	o__UBA7540	f__UBA7540	SPL	Engineered	86.71	56.98	3427702	3329	890.34	28	0	16	88	43	32.56	5.40	Anaerobic	Competitor
GCA_021780075	c__Terriglobia	o__Terriglobales	f__Acidobacteriaceae	SPL	Engineered	88.05	60.09	3877341	3273	1044.65	27	2	8	102	52	25.91	5.00	Anaerobic	Competitor
GCA_021780095	c__Terriglobia	o__Terriglobales	f__Acidobacteriaceae	SPL	Engineered	89.30	56.96	4756948	4378	970.01	35	2	48	125	66	28.2	6.46	Aerobic	Scarcity
GCA_021780215	c__Terriglobia	o__Acidoferrales	f__UBA7541	SPL	Engineered	89.98	62.36	2971724	2784	959.91	19	0	38	12	32	29.69	5.47	Aerobic	Scarcity
GCA_021780315	c__Terriglobia	o__Terriglobales	f__Acidobacteriaceae	SPL	Engineered	89.04	60.08	4064112	3455	1046.93	29	2	32	95	69	24.19	4.64	Aerobic	Competitor
GCA_021780415	c__Terriglobia	o__Terriglobales	f__Acidobacteriaceae	SPL	Engineered	87.09	63.12	2465708	2272	944.72	14	5	8	29	31	22.13	5.92	Aerobic	Scarcity
GCA_021781075	c__Terriglobia	o__Terriglobales	f__Acidobacteriaceae	SPL	Engineered	90.20	59.79	2517170	2484	911.98	15	1	12	47	47	27.6	5.45	Aerobic	Competitor
GCA_021781255	c__Terriglobia	o__Terriglobales	f__Acidobacteriaceae	SPL	Engineered	87.45	60.19	8489013	8193	905.19	59	7	56	239	129	28.07	4.85	Aerobic	Ruderal
GCA_021781495	c__Terriglobia	o__Terriglobales	f__Acidobacteriaceae	SPL	Engineered	89.01	62.84	3597074	3155	1013.71	23	5	12	122	57	28.55	4.85	Anaerobic	Competitor
GCA_021781795	c__Terriglobia	o__Terriglobales	f__Acidobacteriaceae	SPL	Engineered	89.60	61.61	3398867	2945	1034.47	23	1	16	113	49	25.88	5.07	Aerobic	Ruderal
GCA_021782675	c__Terriglobia	o__Terriglobales	f__Acidobacteriaceae	SPL	Engineered	91.11	62.00	2558327	2490	934.05	16	1	45	47	50	28.95	5.98	Aerobic	Scarcity
GCA_021784615	c__Terriglobia	o__UBA7540	f__UBA7540	SPL	Engineered	86.99	57.64	3355801	3123	932.91	21	1	20	106	33	33.93	5.60	Aerobic	Scarcity
GCA_021785495	c__Terriglobia	o__Terriglobales	f__Acidobacteriaceae	SPL	Engineered	86.47	63.54	2943459	2531	1005.18	23	0	16	65	32	28.85	5.55	Anaerobic	Competitor
GCA_021786455	c__Terriglobia	o__UBA7540	f__UBA7540	SPL	Engineered	87.16	59.00	2887564	2802	896.29	24	2	8	55	26	36.09	5.80	Aerobic	Scarcity
GCA_021787555	c__Terriglobia	o__Bryobacterales	f__Bryobacteraceae	SPL	Engineered	91.77	63.92	5914038	5441	998.40	28	1	8	195	64	32.94	5.59	Aerobic	Ruderal
GCA_021787735	c__Terriglobia	o__UBA7540	f__UBA7540	SPL	Engineered	86.28	56.83	4870921	4342	969.21	52	4	16	136	42	32.26	5.96	Aerobic	Competitor
GCA_021788095	c__Terriglobia	o__Acidoferrales	f__UBA7541	SPL	Engineered	86.69	64.16	4239112	3505	1048.39	40	2	32	66	33	31.53	5.53	Aerobic	Competitor
GCA_021788955	c__Terriglobia	o__Bryobacterales	f__Bryobacteraceae	SPL	Engineered	92.20	63.01	3111962	3272	877.52	26	1	16	87	46	29.46	5.81	Anaerobic	Competitor
GCA_021789565	c__Terriglobia	o__UBA7540	f__UBA7540	SPL	Engineered	86.27	57.16	7724226	7384	901.80	67	5	36	211	55	31.88	5.61	Anaerobic	Competitor
GCA_021789895	c__Terriglobia	o__Terriglobales	f__SCQP01	SPL	Engineered	89.32	65.03	3747463	3076	1086.73	19	1	8	107	31	40.53	5.43	Aerobic	Competitor
GCA_021790655	c__Terriglobia	o__Terriglobales	f__Acidobacteriaceae	SPL	Engineered	89.59	55.90	3563933	3426	932.14	43	0	32	82	57	29.02	6.11	Aerobic	Competitor

GCA_021790995	c__Terriglobia	o__Terriglobales	f__Acidobacteriaceae	SPL	Engineered	87.15	60.51	4149611	3584	1008.96	31	0	24	139	45	28.11	5.73	Anaerobic	Competitor
GCA_021791875	c__Terriglobia	o__Terriglobales	f__Acidobacteriaceae	SPL	Engineered	87.59	59.62	4165849	3469	1052.13	32	2	18	123	43	31.29	4.77	Aerobic	Competitor
GCA_021791935	c__Terriglobia	o__Terriglobales	f__Acidobacteriaceae	SPL	Engineered	87.72	58.68	3165281	3013	920.01	26	0	4	77	47	30.56	6.20	Aerobic	Competitor
GCA_021792135	c__Terriglobia	o__Terriglobales	f__Acidobacteriaceae	SPL	Engineered	88.23	62.53	5109352	4240	1063.30	35	3	24	156	62	29.15	5.87	Aerobic	Competitor
GCA_021792795	c__Terriglobia	o__Terriglobales	f__Acidobacteriaceae	SPL	Engineered	87.88	58.22	4042029	3645	973.90	29	5	12	94	51	29.38	6.41	Aerobic	Competitor
GCA_021793405	c__Terriglobia	o__Terriglobales	f__Acidobacteriaceae	SPL	Engineered	88.35	56.67	4600532	4075	998.31	38	4	36	123	47	29.65	5.76	Aerobic	Competitor
GCA_021793595	c__Terriglobia	o__Terriglobales	f__Acidobacteriaceae	SPL	Engineered	88.83	56.44	5699479	5276	959.11	36	2	36	185	67	29.77	6.34	Aerobic	Ruderal
GCA_021798235	c__Terriglobia	o__Terriglobales	f__Acidobacteriaceae	SPL	Engineered	88.70	59.15	2690612	2593	924.69	23	5	12	45	34	28.28	5.26	Aerobic	Scarcity
GCA_021798655	c__Terriglobia	o__Terriglobales	f__Acidobacteriaceae	SPL	Engineered	89.23	63.43	2834218	2738	922.95	26	2	12	91	48	24.62	5.56	Aerobic	Competitor
GCA_021798735	c__Terriglobia	o__Terriglobales	f__Acidobacteriaceae	SPL	Engineered	88.53	56.84	3646263	3442	935.32	23	4	36	96	46	29.4	5.81	Aerobic	Competitor
GCA_021799405	c__Terriglobia	o__Terriglobales	f__Acidobacteriaceae	SPL	Engineered	87.27	58.87	5763055	4880	1029.65	42	3	26	228	56	29.21	4.95	Aerobic	Competitor
GCA_021799945	c__Terriglobia	o__Terriglobales	f__Acidobacteriaceae	SPL	Engineered	87.08	63.73	3171040	2904	947.42	23	4	24	83	43	31.36	5.21	Aerobic	Competitor
GCA_021800195	c__Terriglobia	o__Terriglobales	f__Acidobacteriaceae	SPL	Engineered	84.91	61.06	3018882	2657	963.18	16	1	24	74	50	27.38	5.90	Aerobic	Scarcity
GCA_021800255	c__Terriglobia	o__Terriglobales	f__Acidobacteriaceae	SPL	Engineered	86.66	61.94	3361861	2859	1018.52	24	0	17	76	34	32.96	5.37	Anaerobic	Competitor
GCA_021800465	c__Terriglobia	o__Acidoferrales	f__UBA7541	SPL	Engineered	86.09	63.05	3931351	3287	1029.42	24	0	32	39	40	37.98	5.61	Anaerobic	Competitor
GCA_021801945	c__Terriglobia	o__Terriglobales	f__Acidobacteriaceae	SPL	Engineered	84.82	61.11	3582735	3328	908.89	23	1	20	80	37	30.02	4.88	Anaerobic	Competitor
GCA_021802085	c__Terriglobia	o__Terriglobales	f__Acidobacteriaceae	SPL	Engineered	87.94	57.58	3398173	3099	961.46	20	2	20	94	44	29.71	6.30	Aerobic	Scarcity
GCA_021803035	c__Terriglobia	o__Terriglobales	f__Acidobacteriaceae	SPL	Engineered	85.49	61.35	3861661	3156	1045.58	20	1	20	112	37	33.2	4.94	Aerobic	Competitor
GCA_021804435	c__Terriglobia	o__Terriglobales	f__Acidobacteriaceae	SPL	Engineered	87.47	62.78	5005232	4702	929.88	33	0	52	160	76	27.89	4.74	Aerobic	Scarcity
GCA_021804655	c__Terriglobia	o__Terriglobales	f__Acidobacteriaceae	SPL	Engineered	90.06	60.27	2864843	2518	1022.15	12	2	12	69	44	24.83	5.65	Aerobic	Competitor
GCA_021804965	c__Terriglobia	o__Terriglobales	f__Acidobacteriaceae	SPL	Engineered	89.87	62.82	2973221	2493	1072.01	19	2	12	63	43	26.39	5.14	Aerobic	Competitor
GCA_021805025	c__Terriglobia	o__Terriglobales	f__Acidobacteriaceae	SPL	Engineered	87.86	60.16	4392903	3965	973.53	34	1	12	134	60	26.32	5.02	Aerobic	Competitor
GCA_021805265	c__Terriglobia	o__Terriglobales	f__Acidobacteriaceae	SPL	Engineered	88.50	61.06	3087407	2805	980.33	18	2	20	79	52	23.15	5.37	Aerobic	Competitor
GCA_021805955	c__Terriglobia	o__Terriglobales	f__Acidobacteriaceae	SPL	Engineered	88.79	56.59	5563798	5449	904.34	42	1	44	179	79	27.78	5.86	Aerobic	Competitor
GCA_021806005	c__Terriglobia	o__Terriglobales	f__Acidobacteriaceae	SPL	Engineered	86.25	61.76	4159611	3338	1072.89	24	3	20	129	57	26.49	5.75	Aerobic	Competitor
GCA_021807455	c__Terriglobia	o__Terriglobales	f__Acidobacteriaceae	SPL	Engineered	89.68	60.70	3342459	3187	936.91	22	0	20	108	54	26.72	5.41	Aerobic	Competitor
GCA_021809235	c__Terriglobia	o__Terriglobales	f__Acidobacteriaceae	SPL	Engineered	90.23	60.48	3004153	2701	1001.07	20	2	24	73	55	27.44	5.47	Aerobic	Scarcity
GCA_021810175	c__Terriglobia	o__Terriglobales	f__Acidobacteriaceae	SPL	Engineered	88.54	62.28	3950497	3825	912.71	27	3	32	116	56	30.29	5.76	Aerobic	Competitor
GCA_021810625	c__Terriglobia	o__Terriglobales	f__Acidobacteriaceae	SPL	Engineered	90.01	57.39	2449385	2162	1019.86	14	1	4	41	29	28.54	5.88	Aerobic	Competitor
GCA_021810735	c__Terriglobia	o__Terriglobales	f__Acidobacteriaceae	SPL	Engineered	88.39	58.07	4285704	4118	917.79	37	6	53	136	39	30.64	4.75	Aerobic	Competitor
GCA_021810815	c__Terriglobia	o__Terriglobales	f__Acidobacteriaceae	SPL	Engineered	87.68	62.03	2924105	2810	911.12	17	1	24	94	44	24.99	5.24	Aerobic	Competitor
GCA_021811745	c__Terriglobia	o__Terriglobales	f__Acidobacteriaceae	SPL	Engineered	86.26	60.89	5144696	5036	878.30	29	1	16	209	73	29.82	4.75	Anaerobic	Scarcity
GCA_021812285	c__Terriglobia	o__Acidoferrales	f__UBA7541	SPL	Engineered	88.87	62.64	3731039	3273	1013.32	28	2	28	48	46	30.73	5.47	Aerobic	Ruderal
GCA_021813485	c__Terriglobia	o__Terriglobales	f__Acidobacteriaceae	SPL	Engineered	88.25	59.71	6218394	6188	885.02	50	7	48	204	80	29.01	4.80	Aerobic	Ruderal
GCA_021813745	c__Terriglobia	o__Terriglobales	f__Acidobacteriaceae	SPL	Engineered	88.79	58.38	4618937	4238	969.81	53	6	26	105	45	27.33	5.14	Aerobic	Competitor
GCA_021813825	c__Terriglobia	o__Bryobacterales	f__Bryobacteraceae	SPL	Engineered	93.18	63.42	5118492	4840	986.99	49	2	20	122	70	30.19	6.09	Anaerobic	Ruderal
GCA_021814265	c__Terriglobia	o__Terriglobales	f__Acidobacteriaceae	SPL	Engineered	88.73	59.25	8955518	8342	951.50	75	12	36	288	97	27.79	4.92	Anaerobic	Ruderal
GCA_021816455	c__Terriglobia	o__Terriglobales	f__Acidobacteriaceae	SPL	Engineered	89.24	59.28	3424574	2885	1059.76	25	2	8	81	39	24.85	5.93	Aerobic	Competitor
GCA_021817045	c__Terriglobia	o__UBA7540	f__UBA7540	SPL	Engineered	86.55	58.03	5253883	4697	967.87	48	2	20	180	31	36.25	5.89	Anaerobic	Scarcity
GCA_021817525	c__Terriglobia	o__UBA7540	f__UBA7540	SPL	Engineered	87.82	59.75	3205717	2820	998.49	27	2	12	80	21	37.57	5.84	Aerobic	Competitor
GCA_021818105	c__Terriglobia	o__Terriglobales	f__Acidobacteriaceae	SPL	Engineered	88.67	59.90	3635135	3149	1026.31	24	1	16	99	47	29.35	5.02	Aerobic	Competitor
GCA_021818585	c__Terriglobia	o__Terriglobales	f__Acidobacteriaceae	SPL	Engineered	87.75	59.48	3213422	2808	1004.32	25	1	24	82	39	32.88	5.05	Aerobic	Competitor
GCA_021818645	c__Terriglobia	o__Terriglobales	f__SCQP01	SPL	Engineered	91.25	61.61	2077219	2138	886.05	17	1	0	67	20	37.44	6.07	Aerobic	Scarcity
GCA_021818865	c__Terriglobia	o__Acidoferrales	f__UBA7541	SPL	Engineered	88.73	62.41	3971114	3408	1033.45	30	4	24	43	41	33.03	6.09	Aerobic	Competitor
GCA_021818955	c__Terriglobia	o__Terriglobales	f__SCQP01	SPL	Engineered	88.06	61.94	3803244	3114	1076.90	29	5	4	89	34	38.68	5.75	Aerobic	Competitor
GCA_021819255	c__Terriglobia	o__Terriglobales	f__Acidobacteriaceae	SPL	Engineered	85.12	61.63	2736211	2516	921.61	14	3	12	87	37	29.21	5.97	Aerobic	Competitor
GCA_021819585	c__Terriglobia	o__Terriglobales	f__Acidobacteriaceae	SPL	Engineered	85.47	61.38	3880561	3252	1017.61	16	1	16	135	50	28.89	5.94	Aerobic	Ruderal
GCA_021823305	c__Terriglobia	o__Terriglobales	f__SCQP01	SPL	Engineered	92.63	69.67	2462326	2605	875.03	24	2	4	17	34	33.99	6.10	Aerobic	Competitor
GCA_021826505	c__Terriglobia	o__Terriglobales	f__SCQP01	SPL	Engineered	91.05	62.21	2752188	2493	1004.87	24	1	0	77	23	37.35	6.44	Aerobic	Competitor
GCA_021826695	c__Terriglobia	o__Terriglobales	f__Acidobacteriaceae	SPL	Engineered	89.25	56.08	3322090	3065	967.76	27	1	12	78	44	30.11	5.07	Aerobic	Competitor
GCA_021827245	c__Terriglobia	o__UBA7540	f__UBA7540	SPL	Engineered	85.41	56.67	7047779	6716	895.73	80	0	16	186	46	33.55	5.90	Anaerobic	Competitor
GCA_021827255	c__Terriglobia	o__UBA7540	f__UBA7540	SPL	Engineered	87.43	61.08	2931195	2851	897.82	26	2	12	66	28	34.84	5.52	Aerobic	Scarcity
GCA_021827785	c__Terriglobia	o__Terriglobales	f__SCQP01	SPL	Engineered	87.67	68.14	3233999	3005	943.35	23	1	16	60	39	39.68	6.13	Aerobic	Scarcity
GCA_021827845	c__Terriglobia	o__Terriglobales	f__Acidobacteriaceae	SPL	Engineered	90.69	61.52	2761318	2493	1012.84	25	3	28	68	48	29.12	6.08	Aerobic	Scarcity
GCA_021828225	c__Terriglobia	o__UBA7540	f__UBA7540	SPL	Engineered	86.65	57.25	3958659	3891	877.28	42	1	20	109	28	35.9	5.99	Anaerobic	Competitor
GCA_021828245	c__Terriglobia	o__UBA7540	f__UBA7540	SPL	Engineered	87.77	59.70	2188559	2136	899.02	18	2	8	60	17	37.06	5.93	Aerobic	Competitor
GCA_021828285	c__Terriglobia	o__UBA7540	f__UBA7540	SPL	Engineered	88.98	60.01	2967316	2636	1000.79	23	1	12	64	28	30.98	5.98	Aerobic	Competitor
GCA_021828835	c__Terriglobia	o__Terriglobales	f__Acidobacteriaceae	SPL	Engineered	88.06	62.94	3918657	3458	995.98	19	0	24	117	47	28.97	4.70	Anaerobic	Competitor
GCA_021831465	c__Terriglobia	o__Terriglobales	f__Acidobacteriaceae	SPL	Engineered	88.69	58.59	2274650	2245	895.41	21	1	12	51	42	29.83	5.90	Aerobic	Competitor
GCA_021832085	c__Terriglobia	o__Acidoferrales	f__UBA7541	SPL	Engineered	86.09	62.99	4022246	3358	1031.64	23	0	32	41	40	37.81	5.60	Anaerobic	Competitor
GCA_021832465	c__Terriglobia	o__Terriglobales	f__Acidobacteriaceae	SPL	Engineered	88.35	57.08	6665046	6397	919.63	47	9	64	164	64	29.18	5.71	Aerobic	Ruderal
GCA_021836865	c__Terriglobia	o__Terriglobales	f__Acidobacteriaceae	SPL	Engineered	88.54	60.82	3981265	3524	1001.57	25	3	18	105	50	25.7	4.77	Aerobic	Competitor
GCA_021837945	c__Terriglobia	o__Terriglobales	f__Acidobacteriaceae	SPL	Engineered	91.25	59.56	3420417	3439	905.93	26	4	12	105	60	28.19	5.33	Aerobic	Competitor
GCA_021838265	c__Terriglobia	o__Terriglobales	f__Acidobacteriaceae	SPL	Engineered	89.20	62.41	2063094	1952	944.26	16	1	12	49	42	27.05	5.64	Aerobic	Competitor
GCA_021838525	c__Terriglobia	o__Terriglobales	f__Acidobacteriaceae	SPL	Engineered	88.37	60.92	5436726	5051	948.37	30	3	16	164	83	26.31	4.75	Aerobic	Competitor
GCA_021838785	c__Terriglobia	o__UBA7540	f__UBA7540	SPL	Engineered	87.86	59.76	3418989	3011	997.54	28	2	12	85	26	37.55	5.81	Aerobic	Competitor
GCA_021838865	c__Terriglobia	o__Terriglobales	f__Acidobacteriaceae	SPL	Engineered	87.48	56.81	3685667	3673	877.76	31	1	16	111	35	30.21	6.28	Aerobic	Competitor
GCA_021839075	c__Terriglobia	o__Terriglobales	f__Acidobacteriaceae	SPL	Engineered	84.53	61.57	2471563	2293	903.67	14	1	12	71	38	28.84	5.93	Aerobic	Scarcity
GCA_021839125	c__Terriglobia	o__Terriglobales	f__Acidobacteriaceae	SPL	Engineered	90.16	57.31	2340636	2259	930.17	18	3	8	32	31	28.92	6.56	Aerobic	Scarcity
GCA_021840915	c__Terriglobia	o__Terriglobales	f__SCQP01	SPL	Engineered	91.31	62.16	2666620	2456	989.24	26	0	0	72	18	37.18	5.98	Aerobic	Competitor
GCA_021841795	c__Terriglobia	o__Terriglobales	f__Acidobacteriaceae	SPL	Engineered	86.74	63.51	2854683	2575	960.17	16	0	24	99	37	28.44	5.91	Aerobic	Scarcity
GCA_021844385	c__Terriglobia	o__Terriglobales	f__Acidobacteriaceae	SPL	Engineered	89.97	57.29	2656199	2380	1000.93	16	1	12	47	34	28.83	6.13	Aerobic	Competitor
GCA_021846065	c__Terriglobia	o__Terriglobales	f__Acidobacteriaceae	SPL	Engineered	91.48	61.12	1782677	1713	946.40	19	1	0	27	41	28.02	6.24	Aerobic	Scarcity
GCA_021846275	c__Terriglobia	o__Terriglobales	f__Acidobacteriaceae	SPL	Engineered	89.01	62.67	3336519	2719	1093.44	22	3	8	72	42	24.14	5.58	Aerobic	Competitor
GCA_021846945	c__Terriglobia	o__Terriglobales	f__Acidobacteriaceae	SPL	Engineered	86.93	59.77	5300585	4497	1024.40	37	4	16	237	49	29.35	4.75	Aerobic	Competitor
GCA_021846985	c__Terriglobia	o__Terriglobales	f__Acidobacteriaceae	SPL	Engineered	87.47	63.07	3788292	3143	1053.75	27	2	12	125	47	31.75	5.41	Anaerobic	Competitor
GCA_021851335	c__Terriglobia	o__Terriglobales	f__Acidobacteriaceae	SPL	Engineered	89.53	59.84	3114085	2610	1068.75	17	2	20	73	47	24.48	5.38	Aerobic	Competitor
GCA_021851385	c__Terriglobia	o__Terriglobales	f__Acidobacteriaceae	SPL	Engineered	87.17	61.89	6813192	6480	914.62	37	6	56	188	71	28.17	4.59	Aerobic	Competitor
GCA_021851465	c__Terriglobia	o__Terriglobales	f__Acidobacteriaceae	SPL	Engineered	86.63	63.38	2610388	2342	964.86	13	0	16	62	34	25.83	5.86	Aerobic	Scarcity
GCA_021851625	c__Terriglobia	o__Terriglobales	f__Acidobacteriaceae	SPL	Engineered	89.70	58.95	1894263	1891	897.37	14	1	8	33	23	26.28	5.40	Aerobic	Scarcity
GCA_022452285	c__Terriglobia	o__VXMN01	f__VXMN01	SPL	Marine	89.27	38.80	3244343	2822	1025.72	31	2	16	67	29	24.82	6.09	Aerobic	Scarcity
GCA_022545685	c__Terriglobia	o__Acidoferrales	f__2_02_FULL_67_57	SPL	Marine	86.64	62.58	2395031	2586	800.83	20	0	20	8	38	45.02	6.10	Aerobic	Scarcity
GCA_022562715	c__Terriglobia	o__Acidoferrales	f__2_02_FULL_67_57	SPL	Marine	87.77	63.06	2364529	2452	846.17	18	0	12	13	34	44.93	6.12	Aerobic	Scarcity
GCA_022564495	c__Terriglobia	o__2_12_FULL_54_10	f__2_12_FULL_54_10	SPL	Marine	85.54	58.65	2569202	2629	833.20	15	0	8	10	23	32.03	5.74	Aerobic	Scarcity
GCA_022564615	c__Terriglobia	o__Acidoferrales	f__2_02_FULL_67_57	SPL	Marine	85.58	62.85	2581082	2603	864.76	24	1	12	15	38	45.64	6.22	Aerobic	Scarcity
GCA_022567475	c__Terriglobia	o__2_12_FULL_54_10	f__2_12_FULL_54_10	SPL	Marine	85.25	58.38	4166097	3871	917.45	20	3	12	15	36	30.79	6.33	Aerobic	Scarcity
GCA_022571295	c__Terriglobia	o__Acidoferrales	f__2_02_FULL_67_57	SPL	Marine	86.04	61.59	3240507	3320	838.09	26	2	12	21	41	43.41	6.12	Aerobic	Competitor

GCA_022571585	c__Terriglobia	o__Acidoferrales	f__2_02_FULL_67_57	SPL	Marine	87.30	62.70	2765713	2684	901.45	23	0	12	18	36	44.11	6.12	Aerobic	Scarcity
GCA_022572385	c__Terriglobia	o__Acidoferrales	f__2_02_FULL_67_57	SPL	Marine	87.43	62.69	2277606	2391	831.70	16	0	8	15	36	44.4	6.12	Aerobic	Scarcity
GCA_022573735	c__Terriglobia	o__Acidoferrales	f__2_02_FULL_67_57	SPL	Marine	85.75	62.49	2755389	2873	821.46	24	0	8	15	38	44.51	6.34	Aerobic	Scarcity
GCA_022615935	c__Terriglobia	o__VXMN01	f__VXMN01	SPL	Terrestrial_n	84.12	56.61	11255037	10152	932.06	89	1	63	139	51	27.48	5.96	Aerobic	Competitor
GCA_022616615	c__Terriglobia	o__VXMN01	f__VXMN01	SPL	Terrestrial_n	84.08	56.40	9576230	8746	919.06	73	2	88	112	44	27.94	5.86	Aerobic	Ruderal
GCA_022736715	c__Terriglobia	o__Terriglobales	f__Acidobacteriaceae	SPL	Terrestrial_n	88.44	58.80	3561566	3318	953.30	31	5	16	61	48	27.92	4.50	Aerobic	Ruderal
GCA_022821145	c__Terriglobia	o__20CM_2_55_15	f__20CM_2_55_15	SPL	Host	91.08	67.23	4179646	3837	992.21	26	3	30	11	34	31.98	6.29	Aerobic	Scarcity
GCA_022821265	c__Terriglobia	o__20CM_2_55_15	f__20CM_2_55_15	SPL	Host	91.76	67.42	3875248	3880	916.70	24	1	28	11	35	31.71	6.29	Aerobic	Scarcity
GCA_022821775	c__Terriglobia	o__VXMN01	f__VXMN01	SPL	Host	87.29	62.44	4397938	3679	1041.76	34	1	28	91	37	31.85	6.50	Aerobic	Scarcity
GCA_022821925	c__Terriglobia	o__Bryobacterales	f__UBA6623	SPL	Host	88.32	63.53	6946379	6047	1013.27	49	7	58	97	41	25.69	6.53	Aerobic	Scarcity
GCA_022823265	c__Terriglobia	o__Bryobacterales	f__UBA6623	SPL	Host	87.91	64.37	5267836	4959	932.38	34	1	44	75	37	25.25	6.17	Aerobic	Scarcity
GCA_022823865	c__Terriglobia	o__Bryobacterales	f__UBA6623	SPL	Host	88.11	64.04	6067373	4919	1084.13	33	4	53	95	31	25.28	6.53	Aerobic	Scarcity
GCA_022824585	c__Terriglobia	o__Bryobacterales	f__UBA6623	SPL	Host	87.93	63.93	6183665	5246	1036.67	34	7	50	88	32	25.63	6.49	Aerobic	Scarcity
GCA_022825405	c__Terriglobia	o__Bryobacterales	f__UBA6623	SPL	Host	88.07	64.09	5360705	4372	1078.57	29	1	36	81	33	25.19	6.53	Aerobic	Scarcity
GCA_022825595	c__Terriglobia	o__Bryobacterales	f__UBA6623	SPL	Host	85.75	64.04	6008422	5383	954.93	39	1	24	68	39	26.75	6.32	Aerobic	Scarcity
GCA_022826755	c__Terriglobia	o__VXMN01	f__VXMN01	SPL	Host	87.51	62.38	4617834	3643	1107.74	37	1	46	97	34	32.16	6.45	Aerobic	Scarcity
GCA_022826925	c__Terriglobia	o__Bryobacterales	f__UBA6623	SPL	Host	88.15	63.87	5965398	4796	1092.99	33	3	42	82	32	25.96	5.73	Aerobic	Scarcity
GCA_022828025	c__Terriglobia	o__Bryobacterales	f__UBA6623	SPL	Host	87.48	64.09	6358836	5328	1043.17	47	4	63	88	36	25.48	6.00	Aerobic	Scarcity
GCA_022842005	c__Terriglobia	o__Bryobacterales	f__Bryobacteraceae	SPL	Engineered	92.68	62.06	7719285	7310	980.17	66	5	44	66	86	25.78	6.07	Aerobic	Competitor
GCA_022843605	c__Terriglobia	o__Bryobacterales	f__Bryobacteraceae	SPL	Engineered	93.13	61.12	6891814	6171	1041.20	55	3	32	71	77	26.35	6.06	Aerobic	Competitor
GCA_022843625	c__Terriglobia	o__Bryobacterales	f__Bryobacteraceae	SPL	Engineered	93.88	56.12	7781097	7088	1033.77	60	4	16	89	64	24.31	6.26	Aerobic	Ruderal
GCA_022866195	c__Terriglobia	o__UBA7540	f__UBA7540	SPL	Marine	86.35	61.18	3578058	3669	839.81	50	0	0	88	41	42.92	6.32	Anaerobic	Scarcity
GCA_022870745	c__Terriglobia	o__Terriglobales	f__Acidobacteriaceae	SPL	Soil	90.64	58.40	5359900	4344	1119.63	45	1	47	153	75	24.74	4.80	Aerobic	Ruderal
GCA_023265315	c__Terriglobia	o__Terriglobales	f__JAHFUA01	SPL	Freshwater	87.58	63.49	3164487	2988	927.79	28	2	20	16	45	37.95	5.96	Aerobic	Scarcity
GCA_023265355	c__Terriglobia	o__Terriglobales	f__Acidobacteriaceae	SPL	Freshwater	90.90	58.67	2709666	2773	887.72	14	2	16	82	64	25.02	5.94	Aerobic	Scarcity
GCA_023265365	c__Terriglobia	o__20CM_2_55_15	f__20CM_2_55_15	SPL	Freshwater	91.82	59.42	4719735	4323	1003.12	37	0	24	24	53	27.4	5.93	Aerobic	Scarcity
GCA_023380575	c__Terriglobia	o__Bryobacterales	f__Bryobacteraceae	SPL	Engineered	92.48	62.95	2074502	2144	895.65	21	0	4	66	29	34.31	6.21	Aerobic	Scarcity
GCA_023382255	c__Terriglobia	o__Acidoferrales	f__UBA7541	SPL	Engineered	89.38	60.78	2781746	2715	914.92	28	0	20	15	42	32.61	5.64	Anaerobic	Scarcity
GCA_023384655	c__Terriglobia	o__Bryobacterales	f__Bryobacteraceae	SPL	Engineered	93.54	64.44	5265624	4759	1035.98	38	1	28	126	50	31.37	5.42	Anaerobic	Competitor
GCA_023384675	c__Terriglobia	o__Bryobacterales	f__Bryobacteraceae	SPL	Engineered	92.26	64.03	5734819	5060	1047.00	45	6	44	147	48	33.58	5.61	Aerobic	Competitor
GCA_023384735	c__Terriglobia	o__Bryobacterales	f__Bryobacteraceae	SPL	Engineered	89.98	61.69	4491275	3847	1050.02	32	3	24	101	41	31.29	6.19	Aerobic	Scarcity
GCA_023410055	c__Terriglobia	o__Terriglobales	f__JAEZPW01	SPL	Host	88.54	56.57	5046213	4488	995.34	33	3	20	84	54	30.92	5.84	Anaerobic	Scarcity
GCA_023415215	c__Terriglobia	o__Terriglobales	f__JAEZPW01	SPL	Host	88.38	56.57	4994243	4345	1017.39	33	1	20	86	55	31.07	5.84	Anaerobic	Competitor
GCA_023436715	c__Terriglobia	o__Bryobacterales	f__Bryobacteraceae	SPL	Host	91.53	62.56	5988944	5477	999.65	45	11	20	138	60	33.76	6.20	Anaerobic	Competitor
GCA_023441255	c__Terriglobia	o__Terriglobales	f__JAEZPW01	SPL	Host	87.91	58.73	5607386	4987	987.91	46	1	20	148	65	30.51	5.97	Aerobic	Scarcity
GCA_023474345	c__Terriglobia	o__UBA7540	f__UBA7540	SPL	Engineered	85.48	56.38	1981897	1720	985.06	16	1	8	60	20	39.3	5.61	Aerobic	Scarcity
GCA_023481105	c__Terriglobia	o__Acidoferrales	f__UBA7541	SPL	Engineered	89.23	60.89	3445516	3143	981.28	32	1	20	20	39	32.97	5.58	Anaerobic	Scarcity
GCA_023511375	c__Terriglobia	o__Terriglobales	f__Acidobacteriaceae	SPL	Freshwater	92.48	57.57	2502442	2300	1005.55	21	1	12	66	36	33.32	5.88	Aerobic	Competitor
GCA_023511565	c__Terriglobia	o__Acidoferrales	f__UBA7541	SPL	Freshwater	90.15	63.92	2615109	2526	932.74	19	2	24	17	37	53.11	6.77	Aerobic	Scarcity
GCA_023511855	c__Terriglobia	o__Bryobacterales	f__Bryobacteraceae	SPL	Freshwater	91.02	63.74	4085096	3797	978.90	46	2	28	118	31	48.04	5.46	Anaerobic	Scarcity
GCA_023512135	c__Terriglobia	o__Acidoferrales	f__UBA7541	SPL	Freshwater	89.95	63.65	2828219	2560	993.34	26	1	32	17	37	54.78	6.16	Aerobic	Scarcity
GCA_023958395	c__Terriglobia	o__Bryobacterales	f__Bryobacteraceae	SPL	Engineered	93.18	64.35	5494946	4917	1043.85	41	4	32	125	53	31.32	5.42	Anaerobic	Competitor
GCA_024281115	c__Terriglobia	o__Terriglobales	f__SbA1	SPL	Soil	90.06	63.56	3575390	3401	946.46	23	0	28	45	55	34.29	6.45	Aerobic	Scarcity
GCA_024638215	c__Terriglobia	o__UBA7540	f__UBA7540	SPL	Freshwater	86.75	60.80	4898650	4531	935.50	36	3	12	107	47	31.49	5.33	Aerobic	Scarcity
GCA_025055455	c__Terriglobia	o__Bryobacterales	f__Bryobacteraceae	SPL	Freshwater	92.45	65.06	3950711	3593	1018.37	37	3	16	92	53	54.39	5.46	Anaerobic	Scarcity
GCA_025056455	c__Terriglobia	o__Bryobacterales	f__Bryobacteraceae	SPL	Freshwater	93.09	58.00	4984781	4061	1144.78	42	0	12	69	57	38.07	5.60	Aerobic	Scarcity
GCA_025056865	c__Terriglobia	o__Bryobacterales	f__Bryobacteraceae	SPL	Freshwater	92.44	67.39	4183825	3611	1072.90	39	3	16	110	49	48.64	5.73	Anaerobic	Scarcity
GCA_025062235	c__Terriglobia	o__Bryobacterales	f__Bryobacteraceae	SPL	Freshwater	91.90	66.70	3211205	2933	1007.14	23	0	8	88	41	58.68	5.81	Anaerobic	Scarcity
GCA_900102185	c__Terriglobia	o__Terriglobales	f__Acidobacteriaceae	SPL	Soil	91.15	56.45	4852066	4097	1080.56	40	2	28	115	65	23.51	5.74	Aerobic	Competitor
GCA_900105625	c__Terriglobia	o__Terriglobales	f__Acidobacteriaceae	SPL	Soil	89.44	59.89	4809171	4003	1075.82	35	4	36	103	66	23.34	4.64	Aerobic	Competitor
GCA_900108185	c__Terriglobia	o__Terriglobales	f__Acidobacteriaceae	SPL	Engineered	89.12	62.00	5669524	4549	1111.52	32	2	45	172	68	22.43	5.19	Aerobic	Competitor
GCA_900114625	c__Terriglobia	o__Terriglobales	f__Acidobacteriaceae	SPL	Soil	90.09	61.28	5275857	4290	1109.20	29	2	28	147	70	23.11	4.57	Aerobic	Competitor
GCA_900188085	c__Terriglobia	o__Terriglobales	f__Acidobacteriaceae	SPL	Soil	89.09	62.91	5293785	4310	1095.14	36	2	28	157	71	22.23	4.41	Aerobic	Competitor
GCA_900290245	c__Terriglobia	o__Terriglobales	f__Acidobacteriaceae	SPL	Soil	86.45	59.44	5286421	4495	1017.19	42	1	32	169	59	25.9	4.59	Anaerobic	Competitor
GCA_900290255	c__Terriglobia	o__Terriglobales	f__SbA1	SPL	Soil	86.47	59.31	5391637	4691	994.11	34	2	40	91	66	30.53	4.80	Aerobic	Competitor
GCA_900290305	c__Terriglobia	o__Bryobacterales	f__Bryobacteraceae	SPL	Soil	87.91	63.01	10082265	9233	960.27	81	1	52	154	95	24.54	5.37	Aerobic	Competitor
GCA_900290315	c__Terriglobia	o__Bryobacterales	f__Bryobacteraceae	SPL	Soil	88.52	61.74	8517636	8176	924.09	65	1	20	203	108	26.86	5.48	Anaerobic	Competitor
GCA_900696665	c__Terriglobia	o__Bryobacterales	f__Bryobacteraceae	SPL	Engineered	92.74	59.11	6037799	5474	1022.87	48	1	28	170	64	32	5.84	Anaerobic	Scarcity
GCA_902812365	c__Terriglobia	o__UBA7540	f__UBA7540	SPL	Soil	88.22	62.82	3495978	2968	1038.32	18	1	8	54	37	39.94	6.36	Aerobic	Competitor
GCA_902812415	c__Terriglobia	o__UBA7540	f__UBA7540	SPL	Soil	86.13	56.43	4844903	4121	1010.35	44	2	20	136	42	33.9	5.71	Aerobic	Competitor
GCA_902812465	c__Terriglobia	o__UBA7540	f__UBA7540	SPL	Soil	86.61	61.75	3916882	3414	993.32	41	2	4	56	39	36.6	5.66	Aerobic	Competitor
GCA_902825945	c__Terriglobia	o__Bryobacterales	f__Bryobacteraceae	SPL	Engineered	92.65	62.95	10154779	8401	1126.28	67	2	62	137	73	24.4	6.28	Anaerobic	Scarcity
GCA_902826605	c__Terriglobia	o__Bryobacterales	f__Bryobacteraceae	SPL	Engineered	91.69	58.17	4488589	4222	980.73	34	1	12	21	55	26.76	5.92	Aerobic	Scarcity
GCA_902826685	c__Terriglobia	o__Bryobacterales	f__Bryobacteraceae	SPL	Engineered	94.76	63.10	7420867	6159	1146.26	61	1	55	82	66	25.45	6.04	Aerobic	Scarcity
GCA_903642225	c__Terriglobia	o__Terriglobales	f__Acidobacteriaceae	SPL	Host	89.12	60.42	3656062	3142	1038.39	20	3	28	88	52	23.24	4.74	Aerobic	Scarcity
GCA_903642235	c__Terriglobia	o__Terriglobales	f__Acidobacteriaceae	SPL	Host	88.87	61.30	3786442	3201	1051.43	28	1	20	82	59	21.29	5.10	Aerobic	Scarcity
GCA_903642265	c__Terriglobia	o__Terriglobales	f__Acidobacteriaceae	SPL	Host	89.42	57.76	4643711	4063	1021.71	38	5	32	107	69	21.46	4.90	Aerobic	Scarcity
GCA_903822935	c__Terriglobia	o__Terriglobales	f__Acidobacteriaceae	SPL	Freshwater	87.07	58.46	5877027	5791	879.44	43	9	20	209	68	24.94	4.56	Anaerobic	Ruderal
GCA_903823185	c__Terriglobia	o__Bryobacterales	f__Bryobacteraceae	SPL	Freshwater	92.07	63.48	7404576	6984	975.01	43	6	20	205	121	24.83	5.42	Aerobic	Scarcity
GCA_903824165	c__Terriglobia	o__Terriglobales	f__Acidobacteriaceae	SPL	Freshwater	88.89	62.31	4341239	3542	1085.03	27	1	12	135	63	22.22	5.26	Aerobic	Competitor
GCA_903824335	c__Terriglobia	o__Terriglobales	f__Acidobacteriaceae	SPL	Freshwater	89.19	58.72	4517954	3708	1087.55	36	1	35	123	55	21.94	4.60	Aerobic	Ruderal
GCA_903825445	c__Terriglobia	o__Bryobacterales	f__Bryobacteraceae	SPL	Freshwater	92.43	58.37	6267350	5968	971.72	37	1	28	83	56	21.68	5.18	Aerobic	Ruderal
GCA_903828455	c__Terriglobia	o__Bryobacterales	f__Bryobacteraceae	SPL	Freshwater	91.33	62.85	4401036	3886	1035.83	23	3	24	27	35	25.26	5.48	Aerobic	Scarcity
GCA_903828675	c__Terriglobia	o__Terriglobales	f__Acidobacteriaceae	SPL	Freshwater	86.69	58.41	6388538	5493	1008.88	46	7	18	222	60	24.43	4.56	Anaerobic	Ruderal
GCA_903831625	c__Terriglobia	o__Terriglobales	f__Acidobacteriaceae	SPL	Freshwater	89.25	58.70	4635492	3830	1081.28	33	2	31	126	56	21.95	4.59	Aerobic	Competitor
GCA_903832055	c__Terriglobia	o__Terriglobales	f__Acidobacteriaceae	SPL	Freshwater	89.21	58.71	4568361	3761	1084.63	33	3	31	125	56	22.1	4.59	Aerobic	Competitor
GCA_903832295	c__Terriglobia	o__Terriglobales	f__Koribacteraceae	SPL	Freshwater	89.34	59.29	4866375	4693	927.08	39	12	20	54	64	27.03	5.55	Anaerobic	Scarcity
GCA_903832475	c__Terriglobia	o__Terriglobales	f__Acidobacteriaceae	SPL	Freshwater	88.30	59.62	5298434	4898	953.36	42	0	52	136	69	22.76	5.61	Aerobic	Ruderal
GCA_903834225	c__Terriglobia	o__Terriglobales	f__Acidobacteriaceae	SPL	Freshwater	87.04	58.27	5531244	5528	869.20	44	5	12	210	67	25.07	5.22	Aerobic	Competitor
GCA_903834955	c__Terriglobia	o__Bryobacterales	f__Bryobacteraceae	SPL	Freshwater	91.70	59.90	6044538	5655	981.74	40	6	24	70	48	22.45	5.56	Aerobic	Competitor
GCA_903834995	c__Terriglobia	o__Terriglobales	f__Acidobacteriaceae	SPL	Freshwater	88.17	63.54	4311077	3575	1063.51	26	2	8	123	56	24.03	4.87	Aerobic	Ruderal
GCA_903839445	c__Terriglobia	o__Bryobacterales	f__Bryobacteraceae	SPL	Freshwater	91.29	62.42	7537591	6806	1008.79	67	1	56	66	69	23.54	5.86	Aerobic	Scarcity
GCA_903840625	c__Terriglobia	o__Terriglobales	f__Acidobacteriaceae	SPL	Freshwater	89.18	58.74	4530651	3717	1088.05	33	2	31	124	55	22.18	4.65	Aerobic	Ruderal
GCA_903841435	c__Terriglobia	o__Terriglobales	f__Koribacteraceae	SPL	Freshwater	88.55	61.46	3151227	3014	924.67	26	7	16	23	48	30.9	5.40	Anaerobic	Scarcity
GCA_903842065	c__Terriglobia	o__Terriglobales	f__Acidobacteriaceae	SPL	Freshwater	86.71	60.36	4086920	3550	998.51	20	7	8	27	47	27.01	4.82	Anaerobic	Competitor
GCA_903842845	c__Terriglobia	o__Terriglobales	f__Acidobacteriaceae	SPL	Freshwater	89.75	57.02	3422741	3400	901.75	24	1	24	61	46	27.42	5.46	Aerobic	Scarcity
GCA_903843365	c__Terriglobia	o__Bryobacterales	f__Bryobacteraceae	SPL	Freshwater	91.62	59.89	6052602	5616	989.45	42	2	24	71	48	22.27	5.56	Aerobic	Ruderal

GCA_903843895	c__Terriglobia	o__Terriglobales	f__Koribacteraceae	SPL	Freshwater	88.45	61.64	2981301	3050	861.92	27	0	20	26	54	31.58	5.52	Anaerobic	Scarcity
GCA_903845665	c__Terriglobia	o__Bryobacterales	f__Bryobacteraceae	SPL	Freshwater	94.06	61.56	6165424	5391	1077.03	44	4	40	86	75	23.49	6.04	Aerobic	Scarcity
GCA_903846985	c__Terriglobia	o__Bryobacterales	f__Bryobacteraceae	SPL	Freshwater	91.33	62.86	4371368	3946	1012.23	23	3	28	29	41	25.25	5.48	Aerobic	Scarcity
GCA_903847135	c__Terriglobia	o__Bryobacterales	f__Bryobacteraceae	SPL	Freshwater	91.62	59.89	5969101	5533	989.94	40	2	24	70	48	22.2	5.71	Aerobic	Ruderal
GCA_903847195	c__Terriglobia	o__Terriglobales	f__CAINCZ01	SPL	Freshwater	87.11	62.60	2278268	2094	945.27	30	2	4	11	22	33.64	6.64	Anaerobic	Competitor
GCA_903848145	c__Terriglobia	o__Terriglobales	f__Acidobacteriaceae	SPL	Freshwater	89.29	61.55	4300035	3814	1007.26	29	2	16	39	59	22.78	5.08	Aerobic	Scarcity
GCA_903849645	c__Terriglobia	o__Terriglobales	f__Acidobacteriaceae	SPL	Freshwater	87.87	63.26	4537117	3760	1060.61	28	4	8	127	57	24.07	4.87	Aerobic	Ruderal
GCA_903851035	c__Terriglobia	o__Terriglobales	f__Koribacteraceae	SPL	Freshwater	88.25	61.19	3263078	2908	990.35	29	2	16	27	46	30.93	5.52	Anaerobic	Competitor
GCA_903851195	c__Terriglobia	o__Bryobacterales	f__Bryobacteraceae	SPL	Freshwater	93.03	62.31	7722420	6619	1086.73	55	5	111	91	88	23.49	5.72	Aerobic	Competitor
GCA_903856035	c__Terriglobia	o__Bryobacterales	f__Bryobacteraceae	SPL	Freshwater	93.07	62.33	7660618	6626	1077.25	55	4	113	90	88	23.47	5.72	Aerobic	Scarcity
GCA_903857425	c__Terriglobia	o__Terriglobales	f__Acidobacteriaceae	SPL	Freshwater	89.23	62.60	4001695	3732	952.98	26	1	16	126	80	23.02	5.64	Aerobic	Scarcity
GCA_903857935	c__Terriglobia	o__Bryobacterales	f__Bryobacteraceae	SPL	Freshwater	92.03	59.88	6723319	6514	950.51	49	0	48	51	78	21.94	5.68	Aerobic	Scarcity
GCA_903858665	c__Terriglobia	o__Terriglobales	f__Acidobacteriaceae	SPL	Freshwater	86.63	58.29	6647754	5823	988.82	52	8	18	221	60	25.5	4.56	Anaerobic	Ruderal
GCA_903862185	c__Terriglobia	o__Bryobacterales	f__Bryobacteraceae	SPL	Freshwater	94.22	61.60	6001272	5442	1040.65	45	2	36	85	80	23.53	6.04	Aerobic	Competitor
GCA_903862325	c__Terriglobia	o__Terriglobales	f__Acidobacteriaceae	SPL	Freshwater	86.99	58.47	6087698	5513	957.63	42	7	21	219	60	24.77	4.56	Anaerobic	Competitor
GCA_903864795	c__Terriglobia	o__Bryobacterales	f__Bryobacteraceae	SPL	Freshwater	89.20	61.64	6962229	6422	964.60	57	3	40	241	67	24.98	5.64	Anaerobic	Scarcity
GCA_903871075	c__Terriglobia	o__Bryobacterales	f__Bryobacteraceae	SPL	Freshwater	88.95	61.53	7304164	6169	1053.21	58	7	30	238	62	25.06	5.72	Anaerobic	Ruderal
GCA_903873705	c__Terriglobia	o__Terriglobales	f__Acidobacteriaceae	SPL	Freshwater	89.36	61.63	4181895	3696	1011.57	30	0	16	39	57	22.95	5.08	Aerobic	Scarcity
GCA_903875575	c__Terriglobia	o__Bryobacterales	f__Bryobacteraceae	SPL	Freshwater	91.61	59.90	5978723	5525	992.95	42	1	24	70	48	22.34	5.71	Aerobic	Competitor
GCA_903875595	c__Terriglobia	o__Terriglobales	f__Acidobacteriaceae	SPL	Freshwater	88.08	60.21	3251253	3269	873.56	22	2	48	74	55	23.71	5.35	Aerobic	Competitor
GCA_903876375	c__Terriglobia	o__Terriglobales	f__Acidobacteriaceae	SPL	Freshwater	89.05	62.43	4305939	3630	1052.86	26	1	16	135	66	22.49	5.10	Aerobic	Scarcity
GCA_903876865	c__Terriglobia	o__Bryobacterales	f__Bryobacteraceae	SPL	Freshwater	91.99	63.57	7265172	6797	982.25	46	6	12	196	130	24.67	5.61	Aerobic	Scarcity
GCA_903879965	c__Terriglobia	o__Bryobacterales	f__Bryobacteraceae	SPL	Freshwater	89.14	61.65	6268356	6187	902.32	58	6	44	214	62	24.67	5.60	Anaerobic	Scarcity
GCA_903881085	c__Terriglobia	o__Bryobacterales	f__Bryobacteraceae	SPL	Freshwater	90.85	57.60	11877886	10310	1045.43	81	9	44	233	102	24.03	5.19	Aerobic	Ruderal
GCA_903881655	c__Terriglobia	o__Terriglobales	f__Koribacteraceae	SPL	Freshwater	88.39	61.33	3250055	3053	938.34	28	4	16	25	49	30.77	5.52	Anaerobic	Competitor
GCA_903882645	c__Terriglobia	o__Bryobacterales	f__Bryobacteraceae	SPL	Freshwater	87.94	64.29	5269037	4951	935.54	44	0	20	132	56	32.58	5.47	Anaerobic	Ruderal
GCA_903890055	c__Terriglobia	o__Bryobacterales	f__Bryobacteraceae	SPL	Freshwater	94.30	61.56	6133833	5427	1067.24	45	3	37	85	76	23.47	5.85	Aerobic	Competitor
GCA_903890465	c__Terriglobia	o__Terriglobales	f__Acidobacteriaceae	SPL	Freshwater	88.93	62.42	4357436	3788	1019.93	26	2	12	137	73	22.66	5.36	Aerobic	Scarcity
GCA_903892565	c__Terriglobia	o__Bryobacterales	f__Bryobacteraceae	SPL	Freshwater	90.14	58.47	4625596	4535	917.44	27	4	24	17	51	26.11	5.90	Aerobic	Scarcity
GCA_903893525	c__Terriglobia	o__Bryobacterales	f__Bryobacteraceae	SPL	Freshwater	92.94	60.62	6666096	6577	942.21	61	4	52	64	80	21.81	6.45	Aerobic	Scarcity
GCA_903895925	c__Terriglobia	o__Terriglobales	f__Acidobacteriaceae	SPL	Freshwater	89.36	61.61	4333924	3981	972.04	29	2	16	38	59	22.7	4.97	Aerobic	Scarcity
GCA_903900495	c__Terriglobia	o__Bryobacterales	f__Bryobacteraceae	SPL	Freshwater	90.96	57.60	11844676	10817	992.29	84	6	48	227	109	24.1	5.79	Aerobic	Scarcity
GCA_903900705	c__Terriglobia	o__Bryobacterales	f__Bryobacteraceae	SPL	Freshwater	94.14	61.62	6108339	5311	1084.70	48	1	40	86	76	23.44	6.04	Aerobic	Scarcity
GCA_903903725	c__Terriglobia	o__Bryobacterales	f__Bryobacteraceae	SPL	Freshwater	94.13	61.76	5505485	4850	1070.05	38	0	36	77	73	23.56	5.89	Aerobic	Competitor
GCA_903904755	c__Terriglobia	o__Terriglobales	f__Acidobacteriaceae	SPL	Freshwater	88.92	62.44	4224583	3466	1078.75	27	1	12	130	63	22.53	5.26	Aerobic	Ruderal
GCA_903907865	c__Terriglobia	o__Bryobacterales	f__Bryobacteraceae	SPL	Freshwater	91.40	62.45	7506831	6797	1008.39	68	2	60	66	69	23.55	5.86	Aerobic	Scarcity
GCA_903909435	c__Terriglobia	o__Bryobacterales	f__Bryobacteraceae	SPL	Freshwater	88.89	61.58	7589053	6429	1047.97	62	6	30	247	61	24.79	5.64	Anaerobic	Competitor
GCA_903910425	c__Terriglobia	o__Bryobacterales	f__Bryobacteraceae	SPL	Freshwater	91.86	59.85	6862357	6278	1006.08	38	3	36	57	65	22.04	5.54	Aerobic	Scarcity
GCA_903910605	c__Terriglobia	o__Bryobacterales	f__Bryobacteraceae	SPL	Freshwater	91.95	63.49	7530912	7158	966.69	42	4	20	207	141	24.45	6.14	Anaerobic	Scarcity
GCA_903911925	c__Terriglobia	o__Terriglobales	f__Acidobacteriaceae	SPL	Freshwater	87.95	59.20	5794370	4880	1044.85	42	5	24	178	62	24.21	5.01	Anaerobic	Ruderal
GCA_903912655	c__Terriglobia	o__Terriglobales	f__Koribacteraceae	SPL	Freshwater	88.33	61.20	3257868	2996	958.78	29	4	20	24	53	31.05	5.52	Anaerobic	Competitor
GCA_903914195	c__Terriglobia	o__Bryobacterales	f__Bryobacteraceae	SPL	Freshwater	91.66	59.90	5894176	5776	936.28	42	6	24	76	55	22.08	5.71	Aerobic	Competitor
GCA_903914685	c__Terriglobia	o__Bryobacterales	f__Bryobacteraceae	SPL	Freshwater	92.04	59.96	6390325	6534	899.32	43	6	56	52	78	22.2	5.54	Aerobic	Scarcity
GCA_903920995	c__Terriglobia	o__Bryobacterales	f__Bryobacteraceae	SPL	Freshwater	91.45	62.45	7476662	6759	1009.72	68	2	60	66	70	23.55	5.86	Aerobic	Scarcity
GCA_903921705	c__Terriglobia	o__Terriglobales	f__Acidobacteriaceae	SPL	Freshwater	88.62	62.85	2534396	2108	1060.93	16	1	12	80	37	22.69	5.68	Aerobic	Scarcity
GCA_903923365	c__Terriglobia	o__Bryobacterales	f__Bryobacteraceae	SPL	Freshwater	93.47	60.60	6258048	5801	1008.38	47	3	52	54	72	21.94	6.03	Aerobic	Scarcity
GCA_903923385	c__Terriglobia	o__Bryobacterales	f__Bryobacteraceae	SPL	Freshwater	93.05	62.27	7686247	6612	1083.44	57	4	105	89	87	23.49	5.72	Aerobic	Scarcity
GCA_903923395	c__Terriglobia	o__Bryobacterales	f__Bryobacteraceae	SPL	Freshwater	93.82	58.66	6152153	5504	1051.18	47	8	28	104	78	24.75	5.76	Aerobic	Scarcity
GCA_903925015	c__Terriglobia	o__Bryobacterales	f__Bryobacteraceae	SPL	Freshwater	93.35	60.61	6331154	5886	1004.26	51	4	48	56	71	21.9	6.03	Aerobic	Scarcity
GCA_903925975	c__Terriglobia	o__Bryobacterales	f__Bryobacteraceae	SPL	Freshwater	88.96	62.45	3866401	3634	947.32	31	4	20	29	51	28.42	5.86	Anaerobic	Competitor
GCA_903926625	c__Terriglobia	o__Terriglobales	f__Acidobacteriaceae	SPL	Freshwater	86.52	58.62	5731285	4948	1001.59	35	8	17	217	63	24.9	4.67	Anaerobic	Competitor
GCA_903927035	c__Terriglobia	o__Bryobacterales	f__Bryobacteraceae	SPL	Freshwater	94.40	61.64	6018601	5294	1074.99	45	2	36	86	80	23.34	5.85	Aerobic	Scarcity
GCA_903929755	c__Terriglobia	o__Bryobacterales	f__Bryobacteraceae	SPL	Freshwater	91.84	59.88	6855185	6222	1012.60	38	3	36	57	65	22.02	5.54	Aerobic	Scarcity
GCA_903932155	c__Terriglobia	o__Terriglobales	f__Acidobacteriaceae	SPL	Freshwater	88.38	59.21	3881410	3550	963.63	18	3	28	125	45	24.22	5.25	Aerobic	Competitor
GCA_903932385	c__Terriglobia	o__Terriglobales	f__Acidobacteriaceae	SPL	Freshwater	91.24	57.34	2925465	3014	884.22	22	2	28	77	46	27.68	5.34	Aerobic	Scarcity
GCA_903970165	c__Terriglobia	o__Acidoferrales	f__UBA7541	SPL	Soil	88.62	56.96	5607130	4905	1013.80	49	0	28	79	82	24.74	5.28	Aerobic	Competitor
GCA_903970205	c__Terriglobia	o__Bryobacterales	f__Bryobacteraceae	SPL	Soil	91.89	59.26	4156146	4033	947.72	40	2	28	84	49	24.11	5.72	Aerobic	Scarcity
GCA_903970285	c__Terriglobia	o__Terriglobales	f__Acidobacteriaceae	SPL	Soil	89.31	63.13	4133772	3522	1049.02	32	2	30	100	59	22.96	4.83	Aerobic	Scarcity
GCA_903970395	c__Terriglobia	o__Terriglobales	f__Acidobacteriaceae	SPL	Soil	88.15	60.21	3824088	3425	983.70	28	0	28	93	74	22.87	4.34	Aerobic	Competitor
GCA_903970405	c__Terriglobia	o__Bryobacterales	f__Bryobacteraceae	SPL	Soil	88.62	59.57	5452513	4889	987.29	43	2	32	92	62	26.45	5.96	Aerobic	Competitor
GCA_903970445	c__Terriglobia	o__Terriglobales	f__Acidobacteriaceae	SPL	Soil	90.50	57.91	3678110	3396	981.94	19	1	24	80	64	23.77	5.16	Aerobic	Scarcity
GCA_913048155	c__Terriglobia	o__Bryobacterales	f__UBA6623	SPL	Marine	90.37	47.52	2164984	1830	1069.09	16	0	16	30	20	24.77	6.09	Aerobic	Scarcity
GCA_913056065	c__Terriglobia	o__Acidoferrales	f__2_02_FULL_67_57	SPL	Marine	86.71	62.47	2462971	2663	799.21	25	2	8	10	39	44.55	6.09	Aerobic	Scarcity
GCA_945861255	c__Terriglobia	o__Bryobacterales	f__Bryobacteraceae	SPL	Freshwater	90.19	61.24	3665977	3762	879.24	18	2	8	15	50	27.33	5.99	Aerobic	Scarcity
GCA_945861845	c__Terriglobia	o__20CM_2_55_15	f__20CM_2_55_15	SPL	Freshwater	91.35	57.24	3314622	3392	891.95	52	0	8	12	29	27.78	6.34	Anaerobic	Scarcity
GCA_945865815	c__Terriglobia	o__20CM_2_55_15	f__20CM_2_55_15	SPL	Freshwater	92.44	58.03	4342588	3993	1004.79	42	1	4	14	38	28.36	6.09	Aerobic	Scarcity
GCA_945874415	c__Terriglobia	o__Bryobacterales	f__Bryobacteraceae	SPL	Freshwater	91.92	59.45	6005213	5828	948.71	31	1	36	49	83	22.63	5.56	Aerobic	Scarcity
GCA_945890485	c__Terriglobia	o__Bryobacterales	f__Bryobacteraceae	SPL	Freshwater	94.65	61.70	7125751	6226	1086.58	56	1	46	71	73	25.44	6.05	Aerobic	Scarcity
GCA_945893365	c__Terriglobia	o__Bryobacterales	f__Bryobacteraceae	SPL	Freshwater	89.39	61.24	7882498	6891	1021.64	54	2	16	65	69	24.62	5.79	Aerobic	Scarcity
GCA_945905395	c__Terriglobia	o__Bryobacterales	f__Bryobacteraceae	SPL	Freshwater	92.10	59.55	6053857	5617	995.09	35	1	30	48	79	22.46	5.56	Aerobic	Scarcity
GCA_000687145	c__Thermoanaerobaculia	o__Thermoanaerobaculales	f__Thermoanaerobaculaceae	SPL	Freshwater	92.85	63.06	2660928	2383	1040.26	28	1	10	15	30	58.64	6.34	Anaerobic	Scarcity
GCA_001766905	c__Thermoanaerobaculia	o__Thermoanaerobaculales	f__Thermoanaerobaculaceae	SPL	Freshwater	87.46	67.63	3610782	3313	998.00	21	7	16	27	33	41.43	5.39	Anaerobic	Scarcity
GCA_002071135	c__Thermoanaerobaculia	o__UBA5704	f__UBA5704	SPL	Engineered	93.63	72.28	3232885	3288	921.28	26	9	20	17	39	43.51	5.70	Anaerobic	Scarcity
GCA_002279205	c__Thermoanaerobaculia	o__Thermoanaerobaculales	f__Thermoanaerobaculaceae	SPL	Engineered	90.27	70.00	2805258	2852	887.32	18	5	16	17	41	39.7	6.13	Anaerobic	Scarcity
GCA_002279285	c__Thermoanaerobaculia	o__Thermoanaerobaculales	f__Thermoanaerobaculaceae	SPL	Engineered	90.02	70.37	3016655	2902	935.03	24	0	16	18	44	39.78	6.15	Anaerobic	Scarcity
GCA_002327305	c__Thermoanaerobaculia	o__UBA2201	f__UBA2201	SPL	Engineered	89.84	65.51	3646212	3102	1061.36	33	0	12	14	25	32.83	6.20	Anaerobic	Scarcity
GCA_002402485	c__Thermoanaerobaculia	o__UBA2201	f__UBA2201	SPL	Engineered	89.81	65.53	3513415	2964	1069.68	31	0	12	14	21	32.39	6.20	Anaerobic	Scarcity
GCA_002414905	c__Thermoanaerobaculia	o__UBA5066	f__UBA5066	SPL	Engineered	90.14	69.02	4270506	3861	1031.54	32	2	20	23	37	31.68	6.37	Anaerobic	Competitor
GCA_002420005	c__Thermoanaerobaculia	o__UBA5704	f__UBA5704	SPL	Freshwater	91.20	69.38	8024947	6247	1190.87	68	1	348	28	52	37.08	5.80	Aerobic	Scarcity
GCA_002424725	c__Thermoanaerobaculia	o__UBA2201	f__UBA2201	SPL	Engineered	90.02	65.49	3330969	2951	1016.53	23	0	12	12	20	32.16	6.15	Anaerobic	Scarcity
GCA_002923295	c__Thermoanaerobaculia	o__Thermoanaerobaculales	f__Thermoanaerobaculaceae	SPL	Engineered	93.82	63.09	2347125	2336	945.01	29	2	4	9	28	58.91	6.25	Anaerobic	Scarcity
GCA_003105185	c__Thermoanaerobaculia	o__Thermoanaerobaculales	f__Sulfomarinibacteraceae	SPL	Engineered	90.48	69.85	4236048	3653	1049.13	32	2	8	32	32	32.8	6.11	Anaerobic	Scarcity
GCA_003133645	c__Thermoanaerobaculia	o__Thermoanaerobaculales	f__Thermoanaerobaculaceae	SPL	Soil	89.59	69.45	3690370	3436	963.05	25	3	16	18	49	38	6.15	Anaerobic	Scarcity
GCA_003152095	c__Thermoanaerobaculia	o__UBA5066	f__UBA5066	SPL	Soil	92.31	67.67	3637326	3500	963.71	19	4	12	15	38	28.69	5.96	Anaerobic	Scarcity
GCA_003154505	c__Thermoanaerobaculia	o__Thermoanaerobaculales	f__Thermoanaerobaculaceae	SPL	Soil	88.05	66.89	3789588	3512	952.50	23	1	12	22	44	35.5	5.62	Anaerobic	Scarcity
GCA_003155355	c__Thermoanaerobaculia	o__Thermoanaerobaculales	f__Thermoanaerobaculaceae	SPL	Soil	87.77	67.13	3219389	3286	860.71	22	0	8	19	41	35.13	6.15	Anaerobic	Scarcity
GCA_003157625	c__Thermoanaerobaculia	o__Thermoanaerobaculales	f__Thermoanaerobaculaceae	SPL	Soil	88.60	66.86	3840444	3390	1004.59	27	0	12	20	41	35.75	6.31	Anaerobic	Scarcity

GCA_003158325	c__Thermoanaerobaculia	o__Thermoanaerobaculales	f__Thermoanaerobaculaceae	SPL	Soil	88.78	66.88	3808614	3408	992.66	26	0	12	22	45	35.84	6.31	Anaerobic	Scarcity
GCA_003158745	c__Thermoanaerobaculia	o__Thermoanaerobaculales	f__Thermoanaerobaculaceae	SPL	Soil	88.65	66.91	3735526	3306	1004.51	28	0	8	19	39	35.87	6.31	Anaerobic	Scarcity
GCA_003170815	c__Thermoanaerobaculia	o__UBA5066	f__UBA5066	SPL	Soil	92.40	67.61	3832128	3652	972.52	19	4	4	16	35	28.9	6.31	Anaerobic	Scarcity
GCA_003222355	c__Thermoanaerobaculia	o__UBA5066	f__Gp7_AA6	SPL	Soil	91.88	67.57	2635368	2746	894.31	18	0	0	21	35	34.97	6.63	Aerobic	Scarcity
GCA_003222375	c__Thermoanaerobaculia	o__Gp7_AA8	f__Gp7_AA8	SPL	Soil	92.54	61.01	3767804	4210	840.80	36	0	12	45	56	31.16	6.33	Aerobic	Scarcity
GCA_003222385	c__Thermoanaerobaculia	o__UBA5066	f__Gp7_AA6	SPL	Soil	91.74	66.86	3600504	3549	940.35	32	0	0	26	44	32.75	6.86	Aerobic	Scarcity
GCA_003223635	c__Thermoanaerobaculia	o__Gp7_AA8	f__Gp7_AA8	SPL	Soil	93.07	62.40	3729145	3863	902.78	34	0	4	40	51	34.49	7.23	Aerobic	Scarcity
GCA_003223645	c__Thermoanaerobaculia	o__Gp7_AA8	f__Gp7_AA8	SPL	Soil	90.50	63.61	6379710	5544	1044.16	60	4	59	46	49	30.22	5.83	Aerobic	Competitor
GCA_003223675	c__Thermoanaerobaculia	o__UBA5704	f__UBA5704	SPL	Soil	88.27	68.61	6017198	6240	854.74	66	0	156	45	73	35.09	6.37	Aerobic	Scarcity
GCA_003223695	c__Thermoanaerobaculia	o__Gp7_AA8	f__Gp7_AA8	SPL	Soil	91.26	60.27	3877815	4326	831.92	31	0	12	46	61	30.96	6.16	Aerobic	Scarcity
GCA_003231035	c__Thermoanaerobaculia	o__UBA5704	f__UBA5704	SPL	Marine	90.75	63.43	4991115	4751	953.32	53	1	149	19	42	26.1	5.80	Aerobic	Competitor
GCA_003385675	c__Thermoanaerobaculia	o__UBA5704	f__QQVD01	SPL	Engineered	88.74	70.54	6717532	5125	1164.00	46	3	84	21	55	25.74	5.93	Aerobic	Competitor
GCA_003388555	c__Thermoanaerobaculia	o__UBA5704	f__QQVD01	SPL	Engineered	88.74	70.56	6802728	5172	1167.54	48	2	96	21	55	25.61	5.93	Aerobic	Competitor
GCA_003697015	c__Thermoanaerobaculia	o__UBA5704	f__UBA5704	SPL	Freshwater	91.96	71.53	5342023	4400	1116.71	57	0	82	16	42	43.69	6.57	Aerobic	Scarcity
GCA_004298115	c__Thermoanaerobaculia	o__Thermoanaerobaculales	f__Thermoanaerobaculaceae	SPL	Freshwater	90.84	71.18	3974803	3504	1032.12	36	3	12	19	42	37.4	5.99	Anaerobic	Scarcity
GCA_006227875	c__Thermoanaerobaculia	o__UBA5704	f__UBA5704	SPL	Freshwater	92.59	63.65	2952088	2970	921.31	27	1	28	16	40	29.97	6.49	Anaerobic	Competitor
GCA_008363385	c__Thermoanaerobaculia	o__UBA5066	f__UBA5066	SPL	Engineered	92.94	73.10	5897115	4894	1121.45	47	6	36	24	40	33.84	5.42	Anaerobic	Scarcity
GCA_009835535	c__Thermoanaerobaculia	o__UBA5704	f__QQVD01	SPL	Host	94.83	68.34	4486152	3734	1140.56	36	2	20	23	52	26.31	5.98	Aerobic	Scarcity
GCA_009836625	c__Thermoanaerobaculia	o__UBA5704	f__QQVD01	SPL	Host	94.94	68.40	4317546	3567	1151.82	41	1	20	22	52	26.37	5.98	Aerobic	Scarcity
GCA_009837085	c__Thermoanaerobaculia	o__UBA5704	f__QQVD01	SPL	Host	93.92	68.32	4322360	3706	1096.82	35	2	16	18	53	25.84	5.88	Aerobic	Scarcity
GCA_009837885	c__Thermoanaerobaculia	o__UBA5704	f__QQVD01	SPL	Host	93.73	68.44	4556516	3715	1153.77	39	0	16	19	50	25.99	5.88	Aerobic	Scarcity
GCA_009838785	c__Thermoanaerobaculia	o__UBA5704	f__QQVD01	SPL	Host	94.63	68.32	4326598	3547	1155.61	36	2	16	21	48	25.9	5.98	Aerobic	Scarcity
GCA_009839125	c__Thermoanaerobaculia	o__UBA5704	f__QQVD01	SPL	Host	94.35	68.33	4304126	3692	1100.44	38	2	20	19	49	26.03	5.98	Aerobic	Scarcity
GCA_009840205	c__Thermoanaerobaculia	o__UBA5704	f__QQVD01	SPL	Host	94.71	68.40	4414988	3676	1139.00	35	4	16	22	52	26.32	5.98	Aerobic	Scarcity
GCA_009841385	c__Thermoanaerobaculia	o__UBA5704	f__QQVD01	SPL	Host	94.56	68.20	4435576	3666	1145.54	41	3	28	22	52	26.5	5.98	Aerobic	Scarcity
GCA_009842005	c__Thermoanaerobaculia	o__UBA5704	f__QQVD01	SPL	Host	93.48	68.48	3332950	3250	960.35	23	0	16	16	44	26.18	5.88	Aerobic	Scarcity
GCA_009842435	c__Thermoanaerobaculia	o__UBA5704	f__QQVD01	SPL	Host	93.39	68.46	4349466	3839	1059.23	40	0	20	18	50	26.49	5.88	Aerobic	Scarcity
GCA_009843175	c__Thermoanaerobaculia	o__UBA5704	f__QQVD01	SPL	Host	94.30	68.27	4370809	3624	1140.47	36	5	16	21	49	26.38	6.35	Aerobic	Scarcity
GCA_009843505	c__Thermoanaerobaculia	o__UBA5704	f__QQVD01	SPL	Host	94.30	68.31	4522615	3762	1139.89	40	2	20	19	53	25.72	5.98	Aerobic	Scarcity
GCA_009844385	c__Thermoanaerobaculia	o__UBA5704	f__QQVD01	SPL	Host	95.01	68.43	4295420	3558	1148.57	39	1	20	21	52	26.3	5.98	Aerobic	Scarcity
GCA_009845005	c__Thermoanaerobaculia	o__UBA5704	f__QQVD01	SPL	Host	93.58	68.35	3768611	3498	1008.11	35	1	24	18	59	25.73	5.98	Aerobic	Scarcity
GCA_009845425	c__Thermoanaerobaculia	o__UBA5704	f__QQVD01	SPL	Host	94.61	68.31	4305762	3546	1151.49	36	2	16	21	49	25.97	5.98	Aerobic	Scarcity
GCA_011523565	c__Thermoanaerobaculia	o__UBA5704	f__QQVD01	SPL	Host	92.51	68.09	3719825	3685	936.29	24	1	16	16	46	27.56	5.98	Aerobic	Scarcity
GCA_011525365	c__Thermoanaerobaculia	o__UBA5704	f__QQVD01	SPL	Host	94.40	68.47	4223613	3531	1131.60	33	1	16	20	49	26.93	5.88	Aerobic	Scarcity
GCA_011525905	c__Thermoanaerobaculia	o__UBA5704	f__UBA5704	SPL	Engineered	93.80	73.41	3744846	3405	1032.89	26	0	16	19	36	35.61	5.71	Aerobic	Competitor
GCA_012270995	c__Thermoanaerobaculia	o__UBA5704	f__QQVD01	SPL	Host	91.91	68.27	5271755	4553	1066.97	28	0	12	17	57	27.13	6.35	Aerobic	Scarcity
GCA_012516935	c__Thermoanaerobaculia	o__Thermoanaerobaculales	f__Thermoanaerobaculaceae	SPL	Engineered	90.90	68.38	3719233	3239	1044.26	25	2	20	14	35	40.31	5.77	Anaerobic	Scarcity
GCA_012521815	c__Thermoanaerobaculia	o__UBA5704	f__UBA5704	SPL	Engineered	94.09	72.69	2952919	2692	1032.87	23	0	13	14	36	44.13	5.70	Anaerobic	Scarcity
GCA_013152895	c__Thermoanaerobaculia	o__Thermoanaerobaculales	f__Sulfomarinibacteraceae	SPL	Marine	90.29	65.19	2993163	2593	1042.09	34	3	16	14	21	41.51	6.03	Anaerobic	Scarcity
GCA_013152925	c__Thermoanaerobaculia	o__Thermoanaerobaculales	f__Sulfomarinibacteraceae	SPL	Marine	89.62	67.61	3676852	3152	1045.90	30	0	16	20	27	40.91	6.39	Anaerobic	Competitor
GCA_013388425	c__Thermoanaerobaculia	o__Thermoanaerobaculales	f__Thermoanaerobaculaceae	SPL	Freshwater	93.47	69.38	2141126	1939	1031.93	20	0	8	11	26	49.75	6.07	Anaerobic	Scarcity
GCA_014584695	c__Thermoanaerobaculia	o__UBA5704	f__UBA5704	SPL	Engineered	91.62	71.90	4352636	3707	1077.61	32	0	12	19	35	33.45	5.82	Anaerobic	Competitor
GCA_014764485	c__Thermoanaerobaculia	o__Thermoanaerobaculales	f__Sulfomarinibacteraceae	SPL	Marine	90.95	60.86	1778723	1608	1005.10	11	0	12	15	17	32.64	6.03	Anaerobic	Competitor
GCA_014764495	c__Thermoanaerobaculia	o__Thermoanaerobaculales	f__Sulfomarinibacteraceae	SPL	Engineered	89.97	60.70	3393966	3438	889.76	24	0	20	25	48	31.32	5.93	Anaerobic	Scarcity
GCA_015493585	c__Thermoanaerobaculia	o__Thermoanaerobaculales	f__Sulfomarinibacteraceae	SPL	Marine	89.66	62.86	3102394	2676	1038.24	32	2	12	13	21	42.29	5.97	Anaerobic	Competitor
GCA_016184115	c__Thermoanaerobaculia	o__Gp7_AA8	f__Gp7_AA8	SPL	Freshwater	89.71	63.71	4422315	3716	1067.60	34	0	20	16	38	26.68	5.40	Anaerobic	Ruderal
GCA_016213675	c__Thermoanaerobaculia	o__Thermoanaerobaculales	f__Thermoanaerobaculaceae	SPL	Freshwater	91.21	70.83	4967804	4143	1094.70	36	2	28	26	40	33.11	5.74	Anaerobic	Scarcity
GCA_016650215	c__Thermoanaerobaculia	o__Gp7_AA8	f__Gp7_AA8	SPL	Soil	92.06	62.73	3189131	3038	966.16	27	0	4	28	35	28.28	6.15	Aerobic	Scarcity
GCA_016699405	c__Thermoanaerobaculia	o__UBA5704	f__UBA5704	SPL	Engineered	91.86	70.21	5224191	4274	1124.32	40	1	24	32	39	28.36	5.56	Aerobic	Ruderal
GCA_016705025	c__Thermoanaerobaculia	o__UBA5066	f__UBA5066	SPL	Engineered	87.92	70.40	5617161	5294	936.60	36	4	34	29	62	27.77	5.88	Anaerobic	Scarcity
GCA_016714815	c__Thermoanaerobaculia	o__UBA5066	f__UBA5066	SPL	Engineered	91.51	70.35	6725827	5431	1135.43	68	3	48	38	55	28.59	5.62	Anaerobic	Scarcity
GCA_016716705	c__Thermoanaerobaculia	o__UBA5066	f__UBA5066	SPL	Engineered	90.95	70.33	4586032	3988	1048.10	26	0	24	29	47	29.22	6.62	Anaerobic	Scarcity
GCA_016716715	c__Thermoanaerobaculia	o__UBA5066	f__UBA5066	SPL	Engineered	94.27	70.18	4261279	3793	1061.41	25	1	16	17	45	27.01	6.04	Aerobic	Scarcity
GCA_016719485	c__Thermoanaerobaculia	o__UBA5066	f__UBA5066	SPL	Engineered	90.90	70.39	5710626	4902	1061.33	41	4	20	34	53	28.89	5.99	Anaerobic	Scarcity
GCA_016722225	c__Thermoanaerobaculia	o__UBA5066	f__UBA5066	SPL	Engineered	89.55	70.17	7489364	6535	1029.00	65	6	40	37	64	27.72	5.64	Anaerobic	Scarcity
GCA_016789625	c__Thermoanaerobaculia	o__UBA5066	f__UBA5066	SPL	Engineered	95.24	69.65	5072653	4790	1009.12	50	0	36	17	51	28.92	6.20	Aerobic	Competitor
GCA_017860005	c__Thermoanaerobaculia	o__UBA5704	f__UBA5704	SPL	Soil	94.21	73.83	3276508	3098	997.95	30	0	16	15	40	33.58	5.61	Anaerobic	Scarcity
GCA_017883205	c__Thermoanaerobaculia	o__Gp7_AA8	f__Gp7_AA8	SPL	Soil	88.85	62.08	6374606	5826	973.61	50	2	20	43	62	25.93	5.80	Anaerobic	Scarcity
GCA_017883215	c__Thermoanaerobaculia	o__Gp7_AA8	f__Gp7_AA8	SPL	Soil	87.86	64.51	3681589	3436	936.35	29	1	4	30	55	30.65	6.14	Anaerobic	Scarcity
GCA_017883235	c__Thermoanaerobaculia	o__Gp7_AA8	f__Gp7_AA8	SPL	Soil	87.73	62.87	6923523	6253	972.97	58	0	84	38	60	26.27	5.40	Anaerobic	Scarcity
GCA_017883265	c__Thermoanaerobaculia	o__Gp7_AA8	f__Gp7_AA8	SPL	Soil	89.88	62.77	3927193	3778	933.94	34	0	4	26	51	26.56	6.31	Aerobic	Scarcity
GCA_017998715	c__Thermoanaerobaculia	o__UBA5704	f__UBA5704	SPL	Engineered	93.12	69.18	4009883	3743	1003.59	26	2	20	14	39	24.96	5.71	Aerobic	Competitor
GCA_017999215	c__Thermoanaerobaculia	o__UBA5066	f__UBA5066	SPL	Engineered	93.24	71.86	4528593	4257	991.46	32	6	44	20	46	32.02	6.06	Anaerobic	Scarcity
GCA_018000195	c__Thermoanaerobaculia	o__UBA5704	f__UBA5704	SPL	Engineered	93.79	72.57	2904514	2477	1103.18	23	1	18	14	30	43.97	5.70	Anaerobic	Scarcity
GCA_018000955	c__Thermoanaerobaculia	o__Thermoanaerobaculales	f__Thermoanaerobaculaceae	SPL	Engineered	90.40	68.49	3688908	3039	1100.17	24	2	20	12	35	41.17	5.77	Anaerobic	Scarcity
GCA_018001035	c__Thermoanaerobaculia	o__UBA5704	f__UBA5704	SPL	Engineered	93.67	72.62	2919194	2514	1090.95	26	0	18	14	31	44.06	5.70	Anaerobic	Scarcity
GCA_018055265	c__Thermoanaerobaculia	o__UBA5704	f__UBA5704	SPL	Engineered	93.80	72.83	2505468	2357	998.20	28	0	8	13	35	44.63	5.70	Anaerobic	Scarcity
GCA_018057705	c__Thermoanaerobaculia	o__UBA5704	f__UBA5704	SPL	Engineered	91.86	69.12	5082361	4353	1076.97	32	0	23	21	38	23.21	5.64	Aerobic	Ruderal
GCA_018062225	c__Thermoanaerobaculia	o__UBA5704	f__UBA5704	SPL	Engineered	92.50	69.12	4748847	4395	998.20	34	4	22	20	38	23.21	5.61	Aerobic	Scarcity
GCA_019233845	c__Thermoanaerobaculia	o__Gp7_AA8	f__Gp7_AA8	SPL	Host	92.01	62.88	4718049	4832	899.83	48	3	20	37	62	28.06	5.92	Aerobic	Scarcity
GCA_019233985	c__Thermoanaerobaculia	o__Gp7_AA8	f__Gp7_AA8	SPL	Host	94.58	67.07	5372373	4785	1064.72	46	3	42	39	53	30.86	6.07	Aerobic	Competitor
GCA_019242175	c__Thermoanaerobaculia	o__Gp7_AA8	f__Gp7_AA8	SPL	Host	92.02	65.78	5607540	5268	979.64	58	0	112	35	62	30.36	5.80	Aerobic	Competitor
GCA_019243545	c__Thermoanaerobaculia	o__Gp7_AA8	f__Gp7_AA8	SPL	Host	91.10	62.88	5477439	4958	1008.09	46	4	24	38	58	27.18	5.92	Aerobic	Scarcity
GCA_019244185	c__Thermoanaerobaculia	o__Gp7_AA8	f__Gp7_AA8	SPL	Terrestrial_n	94.61	67.10	5304552	4714	1067.34	44	3	42	37	53	31	6.07	Aerobic	Competitor
GCA_019247495	c__Thermoanaerobaculia	o__Gp7_AA8	f__Gp7_AA8	SPL	Terrestrial_n	91.85	62.77	5299842	4893	996.31	47	3	24	41	59	27.22	5.92	Aerobic	Scarcity
GCA_019347945	c__Thermoanaerobaculia	o__Gp7_AA8	f__JAHWKK01	SPL	Terrestrial_n	89.74	61.89	3724673	3297	1018.48	26	1	8	29	38	31.39	6.32	Aerobic	Scarcity
GCA_019509815	c__Thermoanaerobaculia	o__UBA5704	f__UBA5704	SPL	Soil	89.41	68.20	7798914	6631	1053.35	57	0	201	51	63	34.95	5.96	Aerobic	Scarcity
GCA_019911795	c__Thermoanaerobaculia	o__UBA5704	f__JAIOJS01	SPL	Engineered	90.35	62.52	5416258	4714	1036.85	53	1	32	24	34	26.58	5.92	Aerobic	Ruderal
GCA_019911865	c__Thermoanaerobaculia	o__UBA5704	f__UBA5704	SPL	Engineered	91.60	71.01	3764310	3571	966.78	41	2	16	15	25	28.49	6.17	Anaerobic	Scarcity
GCA_020073245	c__Thermoanaerobaculia	o__Thermoanaerobaculales	f__Thermoanaerobaculaceae	SPL	Freshwater	89.60	67.36	4378248	3757	1045.53	33	3	34	25	39	34.79	5.60	Anaerobic	Scarcity
GCA_020199905	c__Thermoanaerobaculia	o__UBA5066	f__Gp7_AA6	SPL	Soil	90.47	67.82	3918712	3511	1011.65	30	0	50	28	45	29.81	6.58	Aerobic	Scarcity
GCA_020200255	c__Thermoanaerobaculia	o__UBA5066	f__Gp7_AA6	SPL	Soil	90.40	67.86	3679405	3411	977.96	28	0	24	24	42	30.36	6.46	Aerobic	Scarcity
GCA_020439725	c__Thermoanaerobaculia	o__UBA5704	f__UBA5704	SPL	Engineered	89.00	68.90	5370608	4852	1015.09	50	1	292	25	44	28.62	6.36	Aerobic	Competitor
GCA_020845555	c__Thermoanaerobaculia	o__UBA5066	f__UBA5066	SPL	Engineered	92.10	65.71	6790479	5633	1110.00	53	4	40	37	43	28.57	6.24	Anaerobic	Scarcity
GCA_021107705	c__Thermoanaerobaculia	o__Thermoanaerobaculales	f__Sulfomarinibacteraceae	SPL	Engineered	89.52	58.85	4322784	3916	984.96	45	1	12	18	37	29.68	6.43	Anaerobic	Competitor
GCA_021154885	c__Thermoanaerobaculia	o__UBA5704	f__UBA5704	SPL	Host	89.54	68.03	8215911	6774	1087.90	59	0	277	50	65	34.55	5.52	Aerobic	Competitor
GCA_021296535	c__Thermoanaerobaculia	o__UBA5704	f__QQVD01	SPL	Marine	89.56	67.93	3758882	3429	1002.63	25	2	8	13	46	27.87	6.20	Aerobic	Scarcity

GCA_021325515	c__Thermoanaerobaculia	o__Gp7_AA8	f__Gp7_AA8	SPL	Soil	93.46	63.28	5377942	4722	1066.64	47	3	28	39	43	29.2	6.20	Anaerobic	Scarcity
GCA_021405005	c__Thermoanaerobaculia	o__UBA5066	f__UBA5066	SPL	Engineered	92.75	72.59	4886814	4839	936.58	41	1	40	22	44	34.22	5.74	Anaerobic	Scarcity
GCA_021781455	c__Thermoanaerobaculia	o__Gp7_AA8	f__Gp7_AA8	SPL	Engineered	87.28	64.57	4880362	4167	1022.69	40	2	20	42	53	28.73	6.24	Anaerobic	Scarcity
GCA_021782525	c__Thermoanaerobaculia	o__Thermoanaerobaculales	f__Thermoanaerobaculaceae	SPL	Engineered	89.69	71.04	2544998	2600	879.42	27	4	0	12	29	37.94	6.08	Anaerobic	Scarcity
GCA_021818005	c__Thermoanaerobaculia	o__Thermoanaerobaculales	f__Thermoanaerobaculaceae	SPL	Engineered	91.45	71.72	3186669	3109	940.94	28	4	8	16	43	39.39	6.57	Anaerobic	Scarcity
GCA_022071945	c__Thermoanaerobaculia	o__UBA5066	f__UBA5066	SPL	Engineered	92.45	65.82	6170929	4774	1195.20	49	2	46	34	37	28.52	5.47	Anaerobic	Scarcity
GCA_022340665	c__Thermoanaerobaculia	o__Thermoanaerobaculales	f__Sulfomarinibacteraceae	SPL	Host	90.79	61.73	3947477	3511	1021.88	32	0	16	29	35	33.25	5.55	Anaerobic	Competitor
GCA_022340775	c__Thermoanaerobaculia	o__Thermoanaerobaculales	f__Sulfomarinibacteraceae	SPL	Host	89.96	66.63	3825667	3330	1034.24	38	0	36	16	35	37.53	6.06	Anaerobic	Scarcity
GCA_022561975	c__Thermoanaerobaculia	o__UBA5704	f__UBA5704	SPL	Marine	90.75	62.80	3659048	3385	980.10	34	1	21	19	33	24.67	5.97	Aerobic	Competitor
GCA_022601195	c__Thermoanaerobaculia	o__UBA5704	f__UBA5704	SPL	Host	89.34	59.23	8010524	5839	1226.52	95	3	158	26	37	23.91	5.71	Aerobic	Ruderal
GCA_022708945	c__Thermoanaerobaculia	o__UBA5704	f__UBA5704	SPL	Engineered	92.89	72.30	2847190	2433	1087.93	24	2	17	12	25	44.21	5.83	Anaerobic	Scarcity
GCA_022709965	c__Thermoanaerobaculia	o__Thermoanaerobaculales	f__Sulfomarinibacteraceae	SPL	Engineered	89.66	71.19	3579096	3160	1015.39	27	0	0	30	30	32.22	6.17	Anaerobic	Scarcity
GCA_022711615	c__Thermoanaerobaculia	o__UBA5066	f__UBA5066	SPL	Engineered	93.94	72.42	4537151	4197	1016.84	41	2	36	22	54	31.65	6.33	Anaerobic	Scarcity
GCA_022820205	c__Thermoanaerobaculia	o__UBA5704	f__QQVD01	SPL	Host	94.24	68.76	4166888	3399	1156.99	27	1	16	18	51	26.06	6.20	Aerobic	Scarcity
GCA_022820605	c__Thermoanaerobaculia	o__UBA5704	f__QQVD01	SPL	Host	94.27	68.81	4238389	3465	1154.04	31	1	12	20	50	25.58	6.20	Aerobic	Scarcity
GCA_022821625	c__Thermoanaerobaculia	o__UBA5704	f__QQVD01	SPL	Host	93.56	68.63	4477025	3654	1147.31	35	0	16	20	46	25.65	6.20	Aerobic	Scarcity
GCA_022822365	c__Thermoanaerobaculia	o__UBA5704	f__QQVD01	SPL	Host	93.93	68.73	4380128	3604	1141.86	35	0	21	18	47	26.1	6.20	Aerobic	Scarcity
GCA_022822945	c__Thermoanaerobaculia	o__UBA5704	f__QQVD01	SPL	Host	93.70	68.75	4378361	3611	1137.07	29	1	20	20	51	26.12	6.35	Aerobic	Scarcity
GCA_022823665	c__Thermoanaerobaculia	o__UBA5704	f__QQVD01	SPL	Host	94.26	68.78	4507562	3600	1181.56	32	0	16	19	55	25.77	6.20	Aerobic	Scarcity
GCA_022824725	c__Thermoanaerobaculia	o__UBA5704	f__QQVD01	SPL	Host	93.11	68.93	3728583	3430	1012.63	27	1	8	16	46	26.56	6.20	Aerobic	Scarcity
GCA_022825485	c__Thermoanaerobaculia	o__UBA5704	f__QQVD01	SPL	Host	93.84	68.74	4506548	3611	1170.76	31	2	20	21	49	25.22	5.98	Aerobic	Scarcity
GCA_022826065	c__Thermoanaerobaculia	o__UBA5704	f__QQVD01	SPL	Host	93.93	68.61	4265850	3493	1148.59	31	0	16	18	50	26.14	6.20	Aerobic	Scarcity
GCA_022826745	c__Thermoanaerobaculia	o__UBA5704	f__QQVD01	SPL	Host	94.13	68.65	4388733	3446	1200.22	34	0	16	20	49	25.79	6.20	Aerobic	Scarcity
GCA_022827455	c__Thermoanaerobaculia	o__UBA5704	f__QQVD01	SPL	Host	93.83	68.70	4488167	3821	1101.52	38	1	20	18	54	26.1	6.48	Aerobic	Scarcity
GCA_022865555	c__Thermoanaerobaculia	o__Thermoanaerobaculales	f__Thermoanaerobaculaceae	SPL	Marine	90.93	68.63	3083150	3137	892.91	19	1	12	19	49	42.44	5.66	Anaerobic	Scarcity
GCA_023151375	c__Thermoanaerobaculia	o__UBA5066	f__UBA5066	SPL	Engineered	92.44	65.69	6812447	5479	1150.83	57	4	40	38	41	28.51	5.47	Anaerobic	Scarcity
GCA_023384635	c__Thermoanaerobaculia	o__UBA5704	f__UBA5704	SPL	Engineered	91.94	73.23	3944608	3917	928.19	30	7	12	16	42	38.07	5.78	Anaerobic	Scarcity
GCA_023700825	c__Thermoanaerobaculia	o__Gp7_AA8	f__Gp7_AA8	SPL	Freshwater	89.43	65.83	4725549	4335	972.28	48	0	20	20	55	26.83	5.64	Anaerobic	Scarcity
GCA_023717585	c__Thermoanaerobaculia	o__UBA5066	f__UBA5066	SPL	Freshwater	94.99	68.48	3735478	3838	925.71	29	0	12	13	52	27.56	5.54	Anaerobic	Scarcity
GCA_024224155	c__Thermoanaerobaculia	o__UBA5704	f__UBA5704	SPL	Marine	90.68	64.05	5714182	5354	968.56	49	4	37	24	44	24.45	5.86	Anaerobic	Competitor
GCA_024226315	c__Thermoanaerobaculia	o__UBA5704	f__UBA5704	SPL	Marine	88.77	66.41	10515415	8690	1074.43	104	8	96	22	59	23.95	6.34	Anaerobic	Ruderal
GCA_024229275	c__Thermoanaerobaculia	o__Thermoanaerobaculales	f__Sulfomarinibacteraceae	SPL	Marine	88.82	56.98	8650952	8941	858.11	113	21	20	37	59	28.43	6.37	Anaerobic	Ruderal
GCA_024277255	c__Thermoanaerobaculia	o__UBA5704	f__UBA5704	SPL	Engineered	90.94	64.04	5950048	4905	1102.89	51	0	72	31	38	24.95	6.09	Aerobic	Competitor
GCA_024638355	c__Thermoanaerobaculia	o__UBA5704	f__UBA5704	SPL	Freshwater	91.68	69.13	4268080	4167	959.87	36	0	48	31	51	28.55	6.32	Aerobic	Scarcity
GCA_024641545	c__Thermoanaerobaculia	o__Thermoanaerobaculales	f__Sulfomarinibacteraceae	SPL	Freshwater	90.08	65.56	5383841	4428	1095.45	49	1	12	43	45	27.64	6.18	Anaerobic	Scarcity
GCA_024763555	c__Thermoanaerobaculia	o__Thermoanaerobaculales	f__Sulfomarinibacteraceae	SPL	Marine	89.33	59.34	3898801	3439	1013.05	38	0	12	15	22	31.71	6.25	Anaerobic	Ruderal
GCA_025060245	c__Thermoanaerobaculia	o__Thermoanaerobaculales	f__Thermoanaerobaculaceae	SPL	Freshwater	94.35	63.86	2351438	2296	968.61	28	0	4	14	31	62.21	6.40	Anaerobic	Scarcity
GCA_903843255	c__Thermoanaerobaculia	o__UBA5066	f__UBA5066	SPL	Freshwater	91.79	69.85	6202376	5247	1085.65	50	5	147	24	49	28.2	5.61	Anaerobic	Scarcity
GCA_903845115	c__Thermoanaerobaculia	o__UBA5066	f__UBA5066	SPL	Freshwater	92.00	69.95	5846797	4925	1093.28	44	1	125	24	44	28.08	5.61	Anaerobic	Scarcity
GCA_903877375	c__Thermoanaerobaculia	o__UBA5066	f__UBA5066	SPL	Freshwater	91.83	69.88	6185326	5226	1088.92	50	4	146	25	45	28.38	5.95	Anaerobic	Scarcity
GCA_903894175	c__Thermoanaerobaculia	o__UBA5066	f__UBA5066	SPL	Freshwater	91.56	69.63	6306379	5248	1101.65	48	5	128	22	40	28.02	5.61	Anaerobic	Scarcity
GCA_903896095	c__Thermoanaerobaculia	o__UBA5066	f__UBA5066	SPL	Freshwater	91.73	69.87	5949241	4960	1102.06	46	2	114	23	43	27.94	5.61	Anaerobic	Scarcity
GCA_903906115	c__Thermoanaerobaculia	o__UBA5066	f__UBA5066	SPL	Freshwater	91.86	69.79	6010540	5397	1022.69	47	3	129	23	49	28	5.61	Anaerobic	Scarcity
GCA_903912895	c__Thermoanaerobaculia	o__UBA5066	f__UBA5066	SPL	Freshwater	91.86	69.90	5940869	4990	1094.93	47	2	134	23	44	28.2	5.61	Anaerobic	Scarcity
GCA_905479655	c__Thermoanaerobaculia	o__UBA5704	f__UBA5704	SPL	Marine	90.46	70.56	4896022	4254	1041.02	51	5	54	37	36	32.39	6.02	Aerobic	Ruderal
GCA_945786845	c__Thermoanaerobaculia	o__UBA5704	f__UBA5704	SPL	Marine	93.37	65.13	3488827	3111	1048.06	29	0	20	17	29	23.13	5.69	Aerobic	Scarcity
GCA_945787525	c__Thermoanaerobaculia	o__UBA5704	f__UBA5704	SPL	Marine	91.78	68.91	4430995	4144	982.34	34	0	44	30	45	27.07	5.76	Aerobic	Scarcity
GCA_945787575	c__Thermoanaerobaculia	o__UBA5704	f__UBA5704	SPL	Marine	91.17	66.14	5592621	5075	1004.81	44	2	40	28	48	24.86	5.87	Aerobic	Scarcity
GCA_945787635	c__Thermoanaerobaculia	o__UBA5704	f__UBA5704	SPL	Marine	92.52	65.00	4355432	3829	1052.55	33	2	24	25	37	22.37	5.14	Anaerobic	Scarcity
GCA_001464065	c__Vicinamibacteria	o__Vicinamibacterales	f__Vicinamibacteraceae	SPL	Engineered	93.12	69.95	5444093	4928	1029.64	34	0	21	84	66	29.63	5.89	Aerobic	Scarcity
GCA_001618865	c__Vicinamibacteria	o__Vicinamibacterales	f__Vicinamibacteraceae	SPL	Soil	88.90	67.22	7480314	6270	1062.44	32	5	16	115	74	29.89	5.97	Aerobic	Competitor
GCA_001724025	c__Vicinamibacteria	o__Vicinamibacterales	f__SCN_69_37	SPL	Engineered	90.89	68.48	4432721	3894	1036.30	39	4	39	35	27	29.11	6.43	Aerobic	Ruderal
GCA_001767055	c__Vicinamibacteria	o__Vicinamibacterales	f__2_12_FULL_66_21	SPL	Freshwater	90.17	67.11	6381533	5597	1030.51	48	3	16	70	60	31.92	6.27	Anaerobic	Competitor
GCA_001767065	c__Vicinamibacteria	o__Vicinamibacterales	f__UBA2999	SPL	Freshwater	90.57	68.11	4409325	4120	973.64	32	3	4	16	40	40.62	6.14	Anaerobic	Scarcity
GCA_001767165	c__Vicinamibacteria	o__Vicinamibacterales	f__UBA2999	SPL	Freshwater	90.76	64.97	3950190	3779	955.09	43	1	26	17	28	32.37	6.30	Anaerobic	Ruderal
GCA_001767195	c__Vicinamibacteria	o__Vicinamibacterales	f__UBA2999	SPL	Freshwater	90.63	67.46	4193868	4023	955.46	34	1	8	24	33	35.95	6.27	Anaerobic	Scarcity
GCA_001767435	c__Vicinamibacteria	o__Vicinamibacterales	f__UBA2999	SPL	Freshwater	88.95	65.50	5508327	5140	957.99	42	0	24	17	43	29.88	6.52	Anaerobic	Scarcity
GCA_001767455	c__Vicinamibacteria	o__Vicinamibacterales	f__2_12_FULL_66_21	SPL	Freshwater	90.37	67.14	6475179	5604	1046.23	54	1	16	71	58	31.96	6.21	Anaerobic	Ruderal
GCA_001767495	c__Vicinamibacteria	o__Vicinamibacterales	f__UBA2999	SPL	Freshwater	90.82	65.09	3554858	3461	936.43	42	0	16	15	28	32.31	6.59	Aerobic	Ruderal
GCA_001767515	c__Vicinamibacteria	o__Vicinamibacterales	f__UBA2999	SPL	Freshwater	90.66	67.36	4106083	3978	940.34	34	3	8	24	36	35.84	6.27	Anaerobic	Scarcity
GCA_001767525	c__Vicinamibacteria	o__Vicinamibacterales	f__UBA2999	SPL	Freshwater	91.42	68.26	4212118	3828	1007.27	31	3	4	15	39	40.4	6.41	Anaerobic	Scarcity
GCA_001768615	c__Vicinamibacteria	o__Vicinamibacterales	f__UBA2999	SPL	Freshwater	91.73	67.21	5309218	4882	1009.85	49	4	25	25	64	30.12	6.25	Aerobic	Scarcity
GCA_001917905	c__Vicinamibacteria	o__Vicinamibacterales	f__UBA2999	SPL	Soil	89.33	64.91	6334740	5642	1011.33	38	0	28	53	65	30.88	6.84	Aerobic	Scarcity
GCA_001918375	c__Vicinamibacteria	o__Vicinamibacterales	f__UBA2999	SPL	Soil	89.33	65.09	5156618	4617	1012.99	34	0	28	48	54	31.69	6.58	Aerobic	Scarcity
GCA_001920805	c__Vicinamibacteria	o__Vicinamibacterales	f__UBA2999	SPL	Soil	89.72	64.95	4491013	4112	990.28	24	0	20	38	49	31.01	6.49	Aerobic	Scarcity
GCA_002238705	c__Vicinamibacteria	o__Bin61	f__Bin61	SPL	Host	88.60	67.65	2880151	2586	986.63	28	2	35	11	21	32.42	6.31	Aerobic	Scarcity
GCA_002296225	c__Vicinamibacteria	o__Vicinamibacterales	f__UBA823	SPL	Marine	89.33	59.67	2537840	2311	1009.47	12	0	12	13	38	35.97	5.94	Aerobic	Scarcity
GCA_002327665	c__Vicinamibacteria	o__Vicinamibacterales	f__UBA823	SPL	Marine	88.87	67.55	3456171	3068	1034.87	23	0	16	23	47	35.54	6.40	Anaerobic	Scarcity
GCA_002328835	c__Vicinamibacteria	o__Vicinamibacterales	f__UBA823	SPL	Marine	91.88	67.61	3631834	3220	1043.15	27	0	16	25	45	30.85	6.40	Aerobic	Scarcity
GCA_002346475	c__Vicinamibacteria	o__Vicinamibacterales	f__UBA2999	SPL	Marine	92.40	46.15	1936883	1810	995.38	15	0	4	5	16	29.2	5.78	Aerobic	Scarcity
GCA_002346545	c__Vicinamibacteria	o__Vicinamibacterales	f__UBA8438	SPL	Marine	89.69	64.48	4120496	3777	1004.73	28	3	16	14	44	33.29	6.52	Aerobic	Scarcity
GCA_002347025	c__Vicinamibacteria	o__Vicinamibacterales	f__UBA8438	SPL	Marine	88.05	53.86	3627052	3076	1047.95	21	0	30	10	45	28.37	6.05	Aerobic	Scarcity
GCA_002413505	c__Vicinamibacteria	o__Vicinamibacterales	f__UBA823	SPL	Marine	84.81	59.53	2226119	2219	925.94	20	0	4	9	27	41.16	6.13	Aerobic	Scarcity
GCA_002456055	c__Vicinamibacteria	o__Vicinamibacterales	f__UBA8438	SPL	Marine	86.19	53.37	2592666	2370	971.63	16	0	20	7	30	30.26	6.37	Aerobic	Scarcity
GCA_002687695	c__Vicinamibacteria	o__Vicinamibacterales	f__UBA823	SPL	Marine	91.89	63.70	4716224	4175	1037.95	30	1	16	26	46	30.2	6.40	Aerobic	Scarcity
GCA_002693525	c__Vicinamibacteria	o__Vicinamibacterales	f__UBA823	SPL	Marine	92.18	59.52	2860686	2745	961.50	26	10	8	13	38	31.53	5.92	Aerobic	Scarcity
GCA_002696705	c__Vicinamibacteria	o__Vicinamibacterales	f__UBA823	SPL	Marine	93.13	49.94	1721444	1605	999.09	10	0	8	15	28	30.98	5.90	Aerobic	Scarcity
GCA_002712885	c__Vicinamibacteria	o__Vicinamibacterales	f__UBA8438	SPL	Marine	89.68	64.89	3834755	3446	998.79	18	1	30	13	50	29.6	6.34	Aerobic	Scarcity
GCA_002714165	c__Vicinamibacteria	o__Vicinamibacterales	f__UBA823	SPL	Marine	92.55	54.40	2043271	1843	1027.22	18	0	8	10	31	32.29	6.12	Aerobic	Scarcity
GCA_002715285	c__Vicinamibacteria	o__Vicinamibacterales	f__UBA823	SPL	Marine	93.48	52.60	3744594	3904	898.91	37	32	12	17	35	28.47	6.08	Aerobic	Scarcity
GCA_002717745	c__Vicinamibacteria	o__Vicinamibacterales	f__UBA823	SPL	Marine	92.89	55.29	1512901	1391	1011.76	11	1	8	8	21	31.98	5.94	Aerobic	Scarcity
GCA_002720285	c__Vicinamibacteria	o__Vicinamibacterales	f__UBA823	SPL	Marine	92.48	54.13	1496158	1419	975.46	6	0	0	4	20	33.59	5.94	Aerobic	Scarcity
GCA_002720815	c__Vicinamibacteria	o__Vicinamibacterales	f__UBA823	SPL	Marine	93.37	50.95	2418508	2173	1039.12	14	0	8	17	34	30.87	6.05	Aerobic	Scarcity
GCA_002722875	c__Vicinamibacteria	o__Vicinamibacterales	f__UBA8438	SPL	Marine	89.64	53.93	3802615	3433	994.71	23	9	28	14	41	27.98	6.37	Aerobic	Scarcity
GCA_002722915	c__Vicinamibacteria	o__Vicinamibacterales	f__UBA8438	SPL	Marine	92.46	63.95	3557150	3231	1019.31	22	1	20	12	44	30.27	6.41	Aerobic	Scarcity
GCA_002726085	c__Vicinamibacteria	o__Vicinamibacterales	f__UBA8438	SPL	Marine	89.65	65.28	4639937	4226	984.28	34	1	20	14	46	28.42	6.07	Aerobic	Scarcity
GCA_002730525	c__Vicinamibacteria	o__Vicinamibacterales	f__UBA823	SPL	Marine	92.50	67.05	3311738	3045	1007.39	27	0	16	23	42	29.25	6.11	Aerobic	Scarcity

GCA_002730735	c__Vicinamibacteria	o__Vicinamibacterales	f__UBA823	SPL	Marine	91.80	55.26	1584695	1402	1037.29	10	0	8	8	21	30.58	6.00	Aerobic	Scarcity
GCA_002737365	c__Vicinamibacteria	o__Vicinamibacterales	f__UBA2999	SPL	Freshwater	93.93	65.13	3053113	2858	1005.43	34	0	17	18	46	28.89	6.40	Aerobic	Scarcity
GCA_003155335	c__Vicinamibacteria	o__Fen_336	f__Fen_336	SPL	Soil	88.06	66.93	2703147	2966	819.70	23	0	4	62	27	32.47	5.94	Anaerobic	Scarcity
GCA_003157675	c__Vicinamibacteria	o__Vicinamibacterales	f__Fen_181	SPL	Soil	90.16	68.78	3307620	3373	887.70	28	0	20	25	56	31.72	6.35	Anaerobic	Scarcity
GCA_003170135	c__Vicinamibacteria	o__Vicinamibacterales	f__Fen_181	SPL	Soil	89.10	67.53	5795962	5083	1016.56	36	9	16	54	65	32.22	6.04	Anaerobic	Competitor
GCA_003171275	c__Vicinamibacteria	o__Vicinamibacterales	f__Fen_181	SPL	Soil	89.31	67.51	5815022	5082	1022.80	38	9	20	56	64	32.28	6.04	Anaerobic	Competitor
GCA_003222275	c__Vicinamibacteria	o__Fen_336	f__Fen_336	SPL	Soil	92.93	69.62	6756227	6310	997.54	51	1	32	99	73	36.53	5.82	Aerobic	Scarcity
GCA_003222295	c__Vicinamibacteria	o__Fen_336	f__Fen_336	SPL	Soil	90.76	70.27	5279708	5444	894.44	45	0	50	74	83	35.32	6.32	Aerobic	Scarcity
GCA_003222395	c__Vicinamibacteria	o__Vicinamibacterales	f__UBA2999	SPL	Soil	90.47	65.11	7741373	6774	1036.60	52	3	67	66	72	30.23	6.65	Aerobic	Scarcity
GCA_003222435	c__Vicinamibacteria	o__Vicinamibacterales	f__UBA2999	SPL	Soil	89.36	65.44	6410125	6378	909.74	44	0	28	50	67	31.1	6.72	Aerobic	Scarcity
GCA_003222455	c__Vicinamibacteria	o__Vicinamibacterales	f__UBA2999	SPL	Soil	88.10	64.33	6277096	5881	943.54	29	2	24	49	70	29.55	6.20	Aerobic	Scarcity
GCA_003222465	c__Vicinamibacteria	o__Vicinamibacterales	f__UBA2999	SPL	Soil	90.39	65.32	7140855	6433	1005.29	48	4	30	62	78	30.45	6.56	Aerobic	Scarcity
GCA_003222505	c__Vicinamibacteria	o__Vicinamibacterales	f__UBA2999	SPL	Soil	88.23	65.46	7168384	6505	976.82	44	1	32	54	69	29.27	5.83	Aerobic	Scarcity
GCA_003222535	c__Vicinamibacteria	o__Vicinamibacterales	f__UBA2999	SPL	Soil	88.57	65.38	8058377	7019	1017.90	49	1	28	56	78	28.96	6.54	Aerobic	Scarcity
GCA_003222555	c__Vicinamibacteria	o__Vicinamibacterales	f__UBA2999	SPL	Soil	85.99	63.83	4792578	4875	859.93	30	0	20	19	54	31.85	6.23	Aerobic	Scarcity
GCA_003222635	c__Vicinamibacteria	o__Vicinamibacterales	f__UBA2999	SPL	Soil	92.24	65.46	5627419	5633	927.09	36	0	36	27	61	32.08	6.23	Aerobic	Scarcity
GCA_003222655	c__Vicinamibacteria	o__Vicinamibacterales	f__2_12_FULL_66_21	SPL	Soil	90.23	67.67	4493384	4567	908.65	36	0	16	65	67	34.83	6.69	Aerobic	Scarcity
GCA_003222675	c__Vicinamibacteria	o__Vicinamibacterales	f__2_12_FULL_66_21	SPL	Soil	88.73	64.54	7161935	6328	1006.71	57	6	24	92	51	29.82	6.02	Aerobic	Ruderal
GCA_003223535	c__Vicinamibacteria	o__Fen_336	f__Fen_336	SPL	Soil	86.89	71.13	5326184	5539	892.24	33	0	48	78	72	39.8	6.21	Aerobic	Scarcity
GCA_003223555	c__Vicinamibacteria	o__Fen_336	f__Fen_336	SPL	Soil	92.22	71.04	5388591	4821	1039.95	27	0	30	79	55	37.95	5.85	Aerobic	Scarcity
GCA_003223715	c__Vicinamibacteria	o__Vicinamibacterales	f__UBA2999	SPL	Soil	90.75	65.35	6860171	6291	992.35	45	2	41	60	67	30.47	6.80	Aerobic	Scarcity
GCA_003223735	c__Vicinamibacteria	o__Vicinamibacterales	f__UBA2999	SPL	Soil	89.16	65.56	6146729	6054	919.99	40	0	32	54	77	31.39	6.72	Aerobic	Scarcity
GCA_003223755	c__Vicinamibacteria	o__Vicinamibacterales	f__UBA2999	SPL	Soil	88.41	65.36	7911612	7305	960.79	60	0	32	53	81	29.4	6.26	Aerobic	Scarcity
GCA_003223775	c__Vicinamibacteria	o__Vicinamibacterales	f__UBA2999	SPL	Soil	91.32	68.39	5173357	5289	898.51	37	0	24	53	75	31.19	6.14	Aerobic	Scarcity
GCA_003223785	c__Vicinamibacteria	o__Vicinamibacterales	f__UBA2999	SPL	Soil	88.06	65.63	6128909	6221	899.82	37	0	32	55	76	32.03	6.55	Aerobic	Scarcity
GCA_003223815	c__Vicinamibacteria	o__Vicinamibacterales	f__UBA2999	SPL	Soil	90.32	65.40	5025571	5517	840.03	30	2	32	24	67	31.69	6.46	Aerobic	Scarcity
GCA_003223835	c__Vicinamibacteria	o__Vicinamibacterales	f__2_12_FULL_66_21	SPL	Soil	91.15	67.57	5754981	5291	1006.68	43	0	24	78	65	34.21	6.33	Aerobic	Ruderal
GCA_003242705	c__Vicinamibacteria	o__Vicinamibacterales	f__SCN_69_37	SPL	Engineered	93.40	69.42	3486942	3211	1015.17	34	0	28	38	34	39.11	6.41	Aerobic	Competitor
GCA_003453375	c__Vicinamibacteria	o__Vicinamibacterales	f__UBA823	SPL	Marine	85.65	67.51	3801042	3457	1012.37	26	1	16	24	44	40.43	6.40	Aerobic	Scarcity
GCA_003509065	c__Vicinamibacteria	o__Vicinamibacterales	f__UBA823	SPL	Marine	86.53	49.15	2565388	2512	909.22	23	0	8	16	33	35.99	6.09	Aerobic	Scarcity
GCA_003533545	c__Vicinamibacteria	o__Vicinamibacterales	f__UBA8438	SPL	Marine	85.91	60.26	3236419	3141	949.40	21	0	16	14	38	39.66	5.98	Aerobic	Scarcity
GCA_004297655	c__Vicinamibacteria	o__Vicinamibacterales	f__SCN_69_37	SPL	Freshwater	95.97	66.62	2792920	2773	967.52	33	0	16	20	55	28.05	6.36	Aerobic	Scarcity
GCA_004356105	c__Vicinamibacteria	o__Bin61	f__SMYC01	SPL	Marine	89.74	60.49	6767314	6605	920.70	56	1	50	28	78	29.83	5.43	Aerobic	Scarcity
GCA_005778315	c__Vicinamibacteria	o__Vicinamibacterales	f__UBA8438	SPL	Freshwater	91.43	65.86	3703507	3778	895.90	23	0	40	10	45	29.6	6.04	Aerobic	Scarcity
GCA_005778535	c__Vicinamibacteria	o__Vicinamibacterales	f__SCN_69_37	SPL	Freshwater	93.10	63.71	1428777	1404	948.50	16	0	4	13	15	27.85	6.50	Aerobic	Scarcity
GCA_005779205	c__Vicinamibacteria	o__Vicinamibacterales	f__UBA2999	SPL	Freshwater	93.62	65.01	3156896	3083	960.54	26	0	29	15	51	27.96	6.34	Aerobic	Scarcity
GCA_005790575	c__Vicinamibacteria	o__Vicinamibacterales	f__UBA2999	SPL	Freshwater	93.58	65.03	3088070	3038	953.00	27	0	21	16	49	28.07	6.40	Aerobic	Scarcity
GCA_005793715	c__Vicinamibacteria	o__Vicinamibacterales	f__SCN_69_37	SPL	Freshwater	93.59	63.76	2612640	2539	964.82	21	0	8	19	36	27.02	6.59	Aerobic	Scarcity
GCA_005798135	c__Vicinamibacteria	o__Vicinamibacterales	f__SCN_69_37	SPL	Freshwater	93.68	63.84	2773619	2679	970.17	25	0	8	23	37	27.36	6.59	Aerobic	Scarcity
GCA_005799825	c__Vicinamibacteria	o__Vicinamibacterales	f__SCN_69_37	SPL	Freshwater	93.37	63.77	2926981	2813	972.73	25	0	8	22	36	27.28	6.67	Aerobic	Scarcity
GCA_005800845	c__Vicinamibacteria	o__Vicinamibacterales	f__UBA2999	SPL	Freshwater	93.67	65.03	3057559	2996	957.88	30	0	17	16	46	27.79	6.40	Aerobic	Scarcity
GCA_005804325	c__Vicinamibacteria	o__Vicinamibacterales	f__UBA2999	SPL	Freshwater	93.52	64.99	3154478	3094	955.66	28	0	21	17	51	28.12	6.34	Aerobic	Scarcity
GCA_009377495	c__Vicinamibacteria	o__Vicinamibacterales	f__Vicinamibacteraceae	SPL	Host	90.61	64.38	6522350	5449	1086.61	54	0	30	108	49	34.85	6.21	Aerobic	Ruderal
GCA_009377605	c__Vicinamibacteria	o__Vicinamibacterales	f__Vicinamibacteraceae	SPL	Host	89.60	64.52	6786431	6115	995.26	64	4	36	111	58	34.6	6.09	Aerobic	Ruderal
GCA_009380045	c__Vicinamibacteria	o__Vicinamibacterales	f__UBA2999	SPL	Host	89.69	66.09	4299268	4023	958.03	36	0	24	27	41	31.33	6.62	Aerobic	Scarcity
GCA_009694685	c__Vicinamibacteria	o__Vicinamibacterales	f__UBA2999	SPL	Freshwater	93.88	65.14	2985608	2875	976.67	33	0	17	17	44	28.21	6.40	Aerobic	Scarcity
GCA_009694735	c__Vicinamibacteria	o__Vicinamibacterales	f__UBA2999	SPL	Freshwater	92.59	65.39	3248293	3141	958.03	26	0	4	12	44	28.44	6.40	Aerobic	Scarcity
GCA_009694745	c__Vicinamibacteria	o__Vicinamibacterales	f__SCN_69_37	SPL	Freshwater	94.06	64.34	2792487	2669	985.70	20	0	4	21	34	27.88	6.86	Aerobic	Scarcity
GCA_009835985	c__Vicinamibacteria	o__Bin61	f__Bin61	SPL	Host	91.57	69.38	3339507	2833	1078.05	28	0	31	18	35	31.45	6.37	Aerobic	Scarcity
GCA_009836105	c__Vicinamibacteria	o__Bin61	f__Bin61	SPL	Host	90.88	69.19	3567093	2995	1082.46	27	3	34	13	37	31.35	6.43	Aerobic	Scarcity
GCA_009837215	c__Vicinamibacteria	o__Bin61	f__Bin61	SPL	Host	91.30	69.23	3473662	2871	1104.60	28	1	38	17	36	31.78	6.34	Aerobic	Scarcity
GCA_009837385	c__Vicinamibacteria	o__Vicinamibacterales	f__UBA8438	SPL	Host	93.52	67.52	4205500	3698	1065.24	25	1	38	10	44	31.75	6.18	Aerobic	Scarcity
GCA_009838025	c__Vicinamibacteria	o__Bin61	f__Bin61	SPL	Host	91.91	69.48	3392438	2859	1089.10	26	1	31	16	36	31.71	6.31	Aerobic	Scarcity
GCA_009838525	c__Vicinamibacteria	o__Vicinamibacterales	f__UBA8438	SPL	Host	93.38	67.44	4292814	3724	1077.20	26	2	38	10	44	31.57	6.18	Aerobic	Scarcity
GCA_009839025	c__Vicinamibacteria	o__Bin61	f__Bin61	SPL	Host	90.98	69.15	3045261	2900	957.35	26	0	32	14	41	31.73	6.53	Aerobic	Scarcity
GCA_009839885	c__Vicinamibacteria	o__Bin61	f__Bin61	SPL	Host	91.55	69.30	3286334	2889	1041.41	26	2	29	17	36	31.38	6.17	Aerobic	Scarcity
GCA_009840635	c__Vicinamibacteria	o__Bin61	f__Bin61	SPL	Host	90.97	69.19	3584940	2994	1089.18	28	2	35	14	36	31.62	6.43	Aerobic	Scarcity
GCA_009841675	c__Vicinamibacteria	o__Vicinamibacterales	f__UBA8438	SPL	Host	93.39	67.63	4210681	3692	1066.15	29	1	33	10	44	31.58	6.18	Aerobic	Scarcity
GCA_009842065	c__Vicinamibacteria	o__Bin61	f__Bin61	SPL	Host	91.71	69.33	3408465	2841	1100.57	27	0	38	15	40	31.71	6.34	Aerobic	Scarcity
GCA_009842615	c__Vicinamibacteria	o__Bin61	f__Bin61	SPL	Host	91.75	69.39	3316163	2897	1053.60	27	0	28	16	36	31.52	6.31	Aerobic	Scarcity
GCA_009843125	c__Vicinamibacteria	o__Bin61	f__Bin61	SPL	Host	91.24	69.49	3081749	2810	1000.45	28	1	24	15	39	31.91	6.37	Aerobic	Scarcity
GCA_009843885	c__Vicinamibacteria	o__Vicinamibacterales	f__UBA8438	SPL	Host	90.37	69.46	5874546	5293	1005.71	50	7	61	19	49	31.97	6.82	Aerobic	Scarcity
GCA_009844005	c__Vicinamibacteria	o__Bin61	f__Bin61	SPL	Host	90.80	69.14	3617733	3036	1081.92	30	0	53	15	37	31.93	6.43	Aerobic	Scarcity
GCA_009844305	c__Vicinamibacteria	o__Bin61	f__Bin61	SPL	Host	91.05	69.14	3575497	3027	1075.56	27	2	34	13	36	31.5	6.43	Aerobic	Scarcity
GCA_009845125	c__Vicinamibacteria	o__Bin61	f__Bin61	SPL	Host	91.71	69.30	3444422	2837	1114.16	26	1	41	15	38	31.94	6.34	Aerobic	Scarcity
GCA_009845395	c__Vicinamibacteria	o__Vicinamibacterales	f__UBA8438	SPL	Host	93.74	67.52	4253972	3709	1076.71	27	0	38	10	45	31.55	6.18	Aerobic	Scarcity
GCA_009846155	c__Vicinamibacteria	o__Bin61	f__Bin61	SPL	Host	90.71	69.11	3568807	3046	1062.97	31	2	42	16	38	31.8	6.43	Aerobic	Scarcity
GCA_009861545	c__Vicinamibacteria	o__Vicinamibacterales	f__UBA8438	SPL	Host	90.06	69.30	6353869	5480	1045.96	42	3	58	17	50	30.96	6.39	Aerobic	Scarcity
GCA_011523485	c__Vicinamibacteria	o__Vicinamibacterales	f__UBA8438	SPL	Host	89.04	69.41	5178805	5426	854.74	43	0	44	12	55	32.18	6.85	Aerobic	Scarcity
GCA_011523845	c__Vicinamibacteria	o__Vicinamibacterales	f__UBA8438	SPL	Host	87.44	69.30	7591109	6785	982.31	55	5	83	17	48	31.51	6.14	Aerobic	Scarcity
GCA_011526565	c__Vicinamibacteria	o__Fen_336	f__Fen_336	SPL	Engineered	94.39	72.83	5793572	5095	1073.83	45	3	44	29	55	31.36	5.97	Aerobic	Competitor
GCA_012271165	c__Vicinamibacteria	o__Vicinamibacterales	f__UBA8438	SPL	Host	92.00	71.38	3722749	3358	1021.21	21	1	24	6	50	33.76	6.22	Aerobic	Scarcity
GCA_012271265	c__Vicinamibacteria	o__Bin61	f__Bin61	SPL	Host	89.64	70.75	3400810	2940	1041.41	26	2	20	15	34	35.23	6.09	Aerobic	Scarcity
GCA_012963185	c__Vicinamibacteria	o__Vicinamibacterales	f__UBA8438	SPL	Marine	92.94	52.04	2757952	2937	870.62	24	0	8	12	34	29.31	5.99	Aerobic	Scarcity
GCA_013140775	c__Vicinamibacteria	o__Vicinamibacterales	f__UBA2999	SPL	Engineered	93.00	63.37	4862586	4479	1010.59	36	2	38	18	37	30.28	6.22	Aerobic	Scarcity
GCA_013140955	c__Vicinamibacteria	o__Vicinamibacterales	f__UBA2999	SPL	Engineered	91.54	69.69	3275261	3265	916.59	29	1	16	19	30	30.74	5.81	Aerobic	Ruderal
GCA_013203475	c__Vicinamibacteria	o__Vicinamibacterales	f__SCN_69_37	SPL	Freshwater	94.05	62.86	3581086	3483	968.59	25	0	20	30	49	27.63	6.33	Aerobic	Scarcity
GCA_013362195	c__Vicinamibacteria	o__Vicinamibacterales	f__2_12_FULL_66_21	SPL	Soil	91.97	68.29	4731495	4440	979.82	40	1	12	67	51	32.76	6.10	Aerobic	Scarcity
GCA_013694635	c__Vicinamibacteria	o__Vicinamibacterales	f__2_12_FULL_66_21	SPL	Soil	93.09	65.15	3718785	3980	869.65	26	2	12	31	51	32.6	5.87	Aerobic	Scarcity
GCA_013694845	c__Vicinamibacteria	o__Vicinamibacterales	f__2_12_FULL_66_21	SPL	Soil	90.35	65.28	5700196	5917	870.18	48	3	16	55	84	30.46	5.90	Aerobic	Scarcity
GCA_013695405	c__Vicinamibacteria	o__Vicinamibacterales	f__Vicinamibacteraceae	SPL	Soil	92.25	68.75	3860068	3955	899.53	18	0	16	54	63	29.69	6.20	Aerobic	Scarcity
GCA_013697225	c__Vicinamibacteria	o__Vicinamibacterales	f__2_12_FULL_66_21	SPL	Soil	92.20	66.61	3468349	3453	925.59	28	0	8	41	48	30.83	5.84	Aerobic	Scarcity
GCA_013813075	c__Vicinamibacteria	o__Vicinamibacterales	f__UBA2999	SPL	Soil	93.01	63.92	3359267	3580	871.94	29	0	16	24	66	29.24	6.45	Aerobic	Scarcity
GCA_013813355	c__Vicinamibacteria	o__Vicinamibacterales	f__2_12_FULL_66_21	SPL	Soil	90.68	66.90	1222635	1169	948.60	6	1	0	6	10	34.21	6.27	Anaerobic	Scarcity
GCA_013817065	c__Vicinamibacteria	o__Vicinamibacterales	f__2_12_FULL_66_21	SPL	Soil	90.44	64.78	5041640	5192	878.80	44	4	20	43	61	29.75	6.20	Aerobic	Scarcity
GCA_015659035	c__Vicinamibacteria	o__Vicinamibacterales	f__UBA8438	SPL	Marine	88.99	66.82	3177098	2781	1015.55	18	2	16	15	44	30.98	6.36	Aerobic	Scarcity
GCA_016178075	c__Vicinamibacteria	o__Vicinamibacterales	f__JACQTH01	SPL	Freshwater	89.08	65.53	4959914	4613	957.14	32	1	8	28	48	32.9	6.46	Aerobic	Scarcity

GCA_016184105	c__Vicinamibacteria	o__Vicinamibacterales	f__2_12_FULL_66_21	SPL	Freshwater	93.20	68.08	4241944	3809	1040.33	41	0	32	39	49	36.25	6.60	Anaerobic	Ruderal
GCA_016184455	c__Vicinamibacteria	o__Vicinamibacterales	f__UBA2999	SPL	Freshwater	92.02	67.72	4726838	4522	963.34	32	0	4	20	43	37.84	5.99	Anaerobic	Scarcity
GCA_016190945	c__Vicinamibacteria	o__Vicinamibacterales	f__UBA2999	SPL	Freshwater	90.71	65.50	1955564	1946	911.50	21	0	4	4	20	38.2	6.36	Aerobic	Ruderal
GCA_016190995	c__Vicinamibacteria	o__Vicinamibacterales	f__JACPPN01	SPL	Freshwater	88.24	65.55	3820008	3401	989.63	22	0	4	22	36	38.13	6.27	Aerobic	Scarcity
GCA_016192845	c__Vicinamibacteria	o__Vicinamibacterales	f__UBA2999	SPL	Freshwater	89.74	67.76	4505731	4248	953.15	32	3	0	20	41	41.37	6.53	Aerobic	Scarcity
GCA_016195265	c__Vicinamibacteria	o__Vicinamibacterales	f__UBA2999	SPL	Freshwater	92.26	66.77	4535142	4082	1026.57	40	0	38	16	47	29.16	6.08	Aerobic	Scarcity
GCA_016195295	c__Vicinamibacteria	o__Vicinamibacterales	f__UBA2999	SPL	Freshwater	91.20	65.48	4172369	4444	857.71	43	0	20	22	46	30.48	6.10	Anaerobic	Scarcity
GCA_016199625	c__Vicinamibacteria	o__Fen_336	f__Fen_336	SPL	Freshwater	94.61	71.10	1372268	1287	1008.52	9	1	8	5	13	42.31	6.09	Aerobic	Scarcity
GCA_016210845	c__Vicinamibacteria	o__Vicinamibacterales	f__UBA2999	SPL	Freshwater	89.92	68.30	4182526	4093	919.16	36	1	12	22	33	38	6.27	Anaerobic	Ruderal
GCA_016210935	c__Vicinamibacteria	o__Vicinamibacterales	f__JACQTH01	SPL	Freshwater	90.56	65.16	3058887	3041	909.81	22	0	8	24	38	34.94	6.21	Aerobic	Scarcity
GCA_016213695	c__Vicinamibacteria	o__Vicinamibacterales	f__UBA2999	SPL	Freshwater	94.29	68.65	3751059	3546	998.89	25	0	4	15	42	35.96	6.27	Aerobic	Scarcity
GCA_016650185	c__Vicinamibacteria	o__Fen_336	f__JAGNNV01	SPL	Soil	91.59	63.85	3851640	3729	942.87	29	0	12	31	69	23.18	6.65	Aerobic	Scarcity
GCA_016650245	c__Vicinamibacteria	o__Vicinamibacterales	f__UBA2999	SPL	Soil	93.08	67.48	4256965	4135	959.01	37	0	12	70	51	28.68	5.99	Aerobic	Scarcity
GCA_016716905	c__Vicinamibacteria	o__Vicinamibacterales	f__SCN_69_37	SPL	Engineered	92.33	64.42	5251049	4736	1026.91	55	1	41	31	63	27.91	5.78	Aerobic	Scarcity
GCA_016789685	c__Vicinamibacteria	o__Vicinamibacterales	f__JAFNAJ01	SPL	Engineered	92.11	69.48	6171741	5401	1052.24	53	0	20	54	75	30.34	5.91	Anaerobic	Scarcity
GCA_016789705	c__Vicinamibacteria	o__Vicinamibacterales	f__UBA2999	SPL	Engineered	94.08	70.05	5034211	4540	1044.48	43	0	20	19	68	30.56	5.52	Aerobic	Scarcity
GCA_016865485	c__Vicinamibacteria	o__Vicinamibacterales	f__Vicinamibacteraceae	SPL	Freshwater	91.78	70.46	6468984	5308	1120.01	31	2	16	106	69	31.22	6.07	Aerobic	Competitor
GCA_016871175	c__Vicinamibacteria	o__Vicinamibacterales	f__UBA2999	SPL	Freshwater	90.83	65.49	4440616	4178	966.07	18	0	24	12	48	30.7	6.34	Aerobic	Scarcity
GCA_016871195	c__Vicinamibacteria	o__Vicinamibacterales	f__UBA2999	SPL	Freshwater	94.72	66.92	3084019	2715	1078.21	26	0	30	17	38	32.09	6.62	Aerobic	Scarcity
GCA_016871255	c__Vicinamibacteria	o__Vicinamibacterales	f__UBA2999	SPL	Freshwater	91.62	69.75	3910054	3617	991.43	28	1	28	10	31	32.9	6.05	Aerobic	Scarcity
GCA_016871275	c__Vicinamibacteria	o__Vicinamibacterales	f__UBA2999	SPL	Freshwater	93.28	65.81	3963882	3580	1034.31	32	0	36	23	58	29.51	6.13	Aerobic	Scarcity
GCA_016871335	c__Vicinamibacteria	o__Vicinamibacterales	f__SCN_69_37	SPL	Freshwater	94.13	65.11	3113930	2895	1014.42	32	0	12	27	44	28.62	6.56	Aerobic	Scarcity
GCA_016871375	c__Vicinamibacteria	o__Vicinamibacterales	f__UBA2999	SPL	Freshwater	93.11	68.86	4217311	4078	965.35	27	0	8	13	42	35.11	6.27	Aerobic	Scarcity
GCA_016934575	c__Vicinamibacteria	o__Fen_336	f__Fen_336	SPL	Freshwater	90.47	62.60	3977865	3429	1048.62	33	1	12	84	24	35.41	6.15	Anaerobic	Ruderal
GCA_017860085	c__Vicinamibacteria	o__Vicinamibacterales	f__JAFNAJ01	SPL	Soil	93.19	68.34	3271865	3165	962.78	25	0	16	43	43	34.2	6.32	Anaerobic	Scarcity
GCA_017883165	c__Vicinamibacteria	o__Vicinamibacterales	f__UBA2999	SPL	Soil	93.62	66.42	4242456	4053	981.69	41	0	20	24	58	30.5	6.08	Anaerobic	Scarcity
GCA_017883185	c__Vicinamibacteria	o__Vicinamibacterales	f__Fen_181	SPL	Soil	88.65	66.78	4847770	4488	957.64	38	0	28	56	69	32.67	6.24	Anaerobic	Competitor
GCA_017990495	c__Vicinamibacteria	o__Fen_336	f__JAGNNV01	SPL	Engineered	93.35	65.56	4204492	3886	1020.01	46	3	24	35	67	24.07	7.00	Aerobic	Scarcity
GCA_018000455	c__Vicinamibacteria	o__Vicinamibacterales	f__UBA2999	SPL	Engineered	92.54	68.93	5188020	4485	1072.11	37	1	21	32	54	28.11	5.93	Aerobic	Scarcity
GCA_018268955	c__Vicinamibacteria	o__Vicinamibacterales	f__UBA2999	SPL	Engineered	91.47	66.96	4773142	4136	1057.45	36	1	28	19	35	30.83	6.49	Aerobic	Competitor
GCA_019694155	c__Vicinamibacteria	o__Fen_336	f__JAGNNV01	SPL	Engineered	93.66	66.74	5272769	4532	1090.74	54	2	32	43	68	25.44	6.30	Aerobic	Ruderal
GCA_019752015	c__Vicinamibacteria	o__Vicinamibacterales	f__UBA2999	SPL	Engineered	92.62	64.20	5917992	5425	1012.42	44	0	41	27	63	29.12	6.49	Aerobic	Competitor
GCA_020073005	c__Vicinamibacteria	o__Vicinamibacterales	f__UBA2999	SPL	Freshwater	93.79	67.07	5857670	5225	1052.50	40	3	28	30	70	28.36	6.30	Aerobic	Scarcity
GCA_020200025	c__Vicinamibacteria	o__Vicinamibacterales	f__UBA2999	SPL	Soil	88.13	65.28	4340755	4322	889.60	30	0	28	30	51	32.07	6.33	Aerobic	Scarcity
GCA_020200195	c__Vicinamibacteria	o__Vicinamibacterales	f__2_12_FULL_66_21	SPL	Soil	90.47	64.78	4005786	4260	855.80	39	1	8	51	63	32.36	6.49	Aerobic	Scarcity
GCA_020845575	c__Vicinamibacteria	o__Vicinamibacterales	f__Vicinamibacteraceae	SPL	Engineered	93.05	68.23	4567922	3923	1085.09	30	0	24	55	51	31.05	6.25	Aerobic	Competitor
GCA_020850715	c__Vicinamibacteria	o__Vicinamibacterales	f__UBA2999	SPL	Engineered	92.73	70.00	3601185	3177	1053.19	33	1	36	22	37	33.65	6.35	Aerobic	Scarcity
GCA_020851325	c__Vicinamibacteria	o__Vicinamibacterales	f__UBA2999	SPL	Engineered	92.01	67.53	4459481	4096	1001.53	35	1	28	24	49	31.13	6.19	Aerobic	Scarcity
GCA_020851565	c__Vicinamibacteria	o__Vicinamibacterales	f__UBA2999	SPL	Engineered	91.89	69.44	6059921	5272	1056.95	48	3	40	25	69	31.36	6.14	Aerobic	Competitor
GCA_020851835	c__Vicinamibacteria	o__Vicinamibacterales	f__UBA2999	SPL	Engineered	93.16	68.51	3877841	3861	936.46	37	1	28	23	53	31.73	6.36	Aerobic	Competitor
GCA_020853395	c__Vicinamibacteria	o__Vicinamibacterales	f__SCN_69_37	SPL	Engineered	91.80	69.52	4281051	3777	1041.97	30	0	24	33	34	33.76	6.29	Aerobic	Competitor
GCA_021296475	c__Vicinamibacteria	o__Vicinamibacterales	f__UBA8438	SPL	Marine	88.10	70.87	3172228	2882	990.05	21	0	16	9	38	35.39	6.07	Aerobic	Scarcity
GCA_021296485	c__Vicinamibacteria	o__Vicinamibacterales	f__UBA8438	SPL	Marine	90.78	68.73	2383979	2172	1015.91	14	0	8	6	27	37.05	5.89	Aerobic	Scarcity
GCA_021296515	c__Vicinamibacteria	o__Vicinamibacterales	f__UBA8438	SPL	Marine	89.14	69.95	3308357	3169	949.53	21	0	30	10	41	35.11	6.57	Aerobic	Scarcity
GCA_021730945	c__Vicinamibacteria	o__Vicinamibacterales	f__UBA2999	SPL	Engineered	94.27	66.31	4728937	4124	1082.53	37	3	21	27	51	28.29	6.32	Aerobic	Scarcity
GCA_021731095	c__Vicinamibacteria	o__Vicinamibacterales	f__UBA2999	SPL	Engineered	93.76	66.31	3614001	3600	941.07	36	5	28	17	31	28.08	6.22	Aerobic	Scarcity
GCA_021731165	c__Vicinamibacteria	o__Vicinamibacterales	f__UBA2999	SPL	Engineered	92.87	65.05	3008190	2795	1001.88	25	0	32	14	21	31.98	6.44	Aerobic	Scarcity
GCA_021779795	c__Vicinamibacteria	o__Vicinamibacterales	f__Fen_181	SPL	Engineered	90.54	71.12	3234140	3048	959.57	29	4	12	30	46	34.21	6.40	Anaerobic	Scarcity
GCA_021824055	c__Vicinamibacteria	o__Vicinamibacterales	f__SCN_69_37	SPL	Engineered	93.01	66.58	3443626	3347	958.14	29	0	16	32	38	27.71	7.09	Aerobic	Scarcity
GCA_022340545	c__Vicinamibacteria	o__Fen_336	f__Fen_336	SPL	Host	92.56	69.48	5041674	4379	1066.03	37	0	24	128	41	33.41	6.58	Anaerobic	Scarcity
GCA_022340585	c__Vicinamibacteria	o__Fen_336	f__Fen_336	SPL	Host	92.39	70.23	6261715	5341	1083.72	40	0	40	188	57	32.51	6.15	Anaerobic	Scarcity
GCA_022447125	c__Vicinamibacteria	o__Vicinamibacterales	f__UBA823	SPL	Marine	91.93	59.89	2931758	2943	914.99	13	0	8	13	52	30.87	5.67	Aerobic	Scarcity
GCA_022448185	c__Vicinamibacteria	o__Vicinamibacterales	f__UBA8438	SPL	Marine	88.77	66.71	4090686	3572	1016.04	26	0	30	15	53	30.44	6.38	Aerobic	Scarcity
GCA_022562495	c__Vicinamibacteria	o__Vicinamibacterales	f__UBA8438	SPL	Marine	86.26	66.84	4737261	4241	963.75	37	1	32	18	48	30.19	6.24	Aerobic	Scarcity
GCA_022819525	c__Vicinamibacteria	o__Bin61	f__Bin61	SPL	Host	91.92	70.05	3000371	2654	1037.85	22	1	21	15	34	33.57	6.31	Aerobic	Scarcity
GCA_022820545	c__Vicinamibacteria	o__Bin61	f__Bin61	SPL	Host	90.86	69.74	3455068	2930	1071.49	28	2	25	16	36	32.61	6.31	Aerobic	Scarcity
GCA_022820675	c__Vicinamibacteria	o__Vicinamibacterales	f__UBA8438	SPL	Host	84.41	71.26	6554762	5948	929.97	44	2	36	10	53	30.22	6.38	Aerobic	Scarcity
GCA_022820785	c__Vicinamibacteria	o__Bin61	f__Bin61	SPL	Host	91.48	69.99	3509756	3026	1060.31	27	0	41	19	38	32.4	6.42	Aerobic	Scarcity
GCA_022820825	c__Vicinamibacteria	o__Vicinamibacterales	f__UBA8438	SPL	Host	93.27	69.46	4230660	3686	1071.11	30	1	30	8	44	34.53	6.43	Aerobic	Scarcity
GCA_022821235	c__Vicinamibacteria	o__Vicinamibacterales	f__UBA8438	SPL	Host	85.08	71.34	6621045	6011	936.90	39	0	32	11	54	30.52	6.14	Aerobic	Scarcity
GCA_022822745	c__Vicinamibacteria	o__Bin61	f__Bin61	SPL	Host	91.12	70.48	3535856	2970	1086.07	27	1	40	9	34	35.22	6.11	Aerobic	Scarcity
GCA_022824695	c__Vicinamibacteria	o__Bin61	f__Bin61	SPL	Host	90.83	69.37	3651323	3088	1074.34	31	1	38	15	38	31.89	6.34	Aerobic	Scarcity
GCA_022824885	c__Vicinamibacteria	o__Bin61	f__Bin61	SPL	Host	91.47	69.93	3385631	2882	1074.53	23	1	30	16	38	32.71	5.82	Aerobic	Scarcity
GCA_022825285	c__Vicinamibacteria	o__Vicinamibacterales	f__UBA8438	SPL	Host	92.77	69.26	4466714	3925	1056.43	34	5	24	7	46	34.32	6.43	Aerobic	Scarcity
GCA_022826195	c__Vicinamibacteria	o__Bin61	f__Bin61	SPL	Host	90.28	69.68	3850216	3261	1065.28	29	3	55	17	37	32.7	6.34	Aerobic	Scarcity
GCA_022826825	c__Vicinamibacteria	o__Bin61	f__Bin61	SPL	Host	89.12	69.44	3984390	3322	1069.31	33	2	67	15	39	32.27	6.30	Aerobic	Scarcity
GCA_022828085	c__Vicinamibacteria	o__Bin61	f__Bin61	SPL	Host	90.35	69.46	3570990	2968	1087.50	25	3	30	14	39	31.82	6.34	Aerobic	Scarcity
GCA_022842525	c__Vicinamibacteria	o__Vicinamibacterales	f__Vicinamibacteraceae	SPL	Engineered	92.35	69.39	7079960	6282	1041.76	41	11	39	98	71	29.55	5.77	Aerobic	Ruderal
GCA_022842985	c__Vicinamibacteria	o__Vicinamibacterales	f__UBA2999	SPL	Engineered	93.57	70.11	4965071	4663	996.93	48	2	24	19	64	29.74	5.92	Aerobic	Scarcity
GCA_023265015	c__Vicinamibacteria	o__Vicinamibacterales	f__UBA2999	SPL	Freshwater	91.47	65.86	3179667	3009	976.07	21	2	8	15	37	34.81	6.15	Aerobic	Scarcity
GCA_023265105	c__Vicinamibacteria	o__Vicinamibacterales	f__UBA2999	SPL	Freshwater	87.69	64.41	4958481	4698	944.73	33	0	24	19	38	31.91	6.52	Aerobic	Scarcity
GCA_023265155	c__Vicinamibacteria	o__Vicinamibacterales	f__UBA2999	SPL	Freshwater	89.34	64.65	2533192	2315	995.59	22	0	12	10	29	31.52	6.36	Aerobic	Scarcity
GCA_023265175	c__Vicinamibacteria	o__Vicinamibacterales	f__UBA2999	SPL	Freshwater	89.12	64.63	4924294	4786	921.79	32	0	8	22	39	31.97	6.55	Aerobic	Competitor
GCA_023384575	c__Vicinamibacteria	o__Vicinamibacterales	f__JAFNAJ01	SPL	Engineered	90.49	70.32	5887706	4895	1098.29	38	2	28	48	57	33.05	6.15	Anaerobic	Scarcity
GCA_023384595	c__Vicinamibacteria	o__Vicinamibacterales	f__JAFNAJ01	SPL	Engineered	89.04	70.45	4826633	4300	1054.60	44	1	16	64	50	35.34	6.03	Aerobic	Scarcity
GCA_023384615	c__Vicinamibacteria	o__Fen_336	f__Fen_336	SPL	Engineered	94.76	72.78	5942579	5108	1105.09	42	4	44	35	54	31.14	5.57	Aerobic	Scarcity
GCA_023418055	c__Vicinamibacteria	o__Vicinamibacterales	f__Vicinamibacteraceae	SPL	Host	93.53	69.09	4487501	4177	1005.57	37	1	16	66	62	32.55	6.32	Aerobic	Scarcity
GCA_023427865	c__Vicinamibacteria	o__Vicinamibacterales	f__Vicinamibacteraceae	SPL	Host	91.65	69.45	4505828	4156	994.32	31	0	8	73	54	32.06	5.99	Aerobic	Scarcity
GCA_023429845	c__Vicinamibacteria	o__Vicinamibacterales	f__Vicinamibacteraceae	SPL	Host	92.30	68.87	4991299	4390	1048.91	35	2	8	76	53	31	5.93	Aerobic	Scarcity
GCA_023701125	c__Vicinamibacteria	o__Fen_336	f__Fen_336	SPL	Freshwater	94.87	72.34	5409304	4861	1056.68	44	1	68	22	66	28.54	5.85	Anaerobic	Scarcity
GCA_023717525	c__Vicinamibacteria	o__Vicinamibacterales	f__UBA2999	SPL	Freshwater	94.70	62.54	3781114	3660	980.72	41	1	16	22	43	29.29	6.00	Aerobic	Scarcity
GCA_024640135	c__Vicinamibacteria	o__Vicinamibacterales	f__SCN_69_37	SPL	Freshwater	94.02	64.77	4320126	3953	1027.96	32	1	46	35	54	28.73	6.53	Aerobic	Scarcity
GCA_024641665	c__Vicinamibacteria	o__Vicinamibacterales	f__2_12_FULL_66_21	SPL	Freshwater	88.59	69.86	6232103	5434	1016.38	43	3	16	67	74	34.45	5.87	Anaerobic	Competitor
GCA_024653875	c__Vicinamibacteria	o__Vicinamibacterales	f__SCN_69_37	SPL	Freshwater	94.73	67.32	3213183	3399	895.77	26	7	16	29	50	28.87	6.77	Anaerobic	Scarcity
GCA_902825985	c__Vicinamibacteria	o__Vicinamibacterales	f__UBA2999	SPL	Engineered	92.23	68.72	5847276	5067	1069.06	37	0	28	26	51	29.74	6.06	Aerobic	Scarcity
GCA_902826065	c__Vicinamibacteria	o__Vicinamibacterales	f__UBA2999	SPL	Engineered	93.92	70.77	5645155	5187	1026.37	38	0	33	25	76	29.5	6.01	Aerobic	Competitor
GCA_902826455	c__Vicinamibacteria	o__Fen_336	f__Fen_336	SPL	Engineered	95.76	70.78	5258802	4716	1069.74	46	3	51	47	58	28.72	6.06	Aerobic	Scarcity

GCA_902826465	c__Vicinamibacteria	o__Vicinamibacterales	f__UBA2999	SPL	Engineered	91.86	65.02	4934225	4969	917.46	36	0	51	25	46	27.48	5.69	Aerobic	Scarcity
GCA_902826515	c__Vicinamibacteria	o__Vicinamibacterales	f__UBA2999	SPL	Engineered	92.79	70.19	5853491	5379	1021.82	50	2	28	30	66	29.35	4.99	Aerobic	Scarcity
GCA_913030755	c__Vicinamibacteria	o__Vicinamibacterales	f__UBA823	SPL	Marine	93.05	55.25	1895174	1839	959.02	15	0	8	9	31	31.44	6.18	Aerobic	Scarcity
GCA_913031175	c__Vicinamibacteria	o__Vicinamibacterales	f__UBA2999	SPL	Marine	93.58	46.54	1618443	1488	1017.27	13	1	4	5	16	29.79	5.96	Aerobic	Scarcity
GCA_913043065	c__Vicinamibacteria	o__Vicinamibacterales	f__UBA8438	SPL	Marine	89.25	53.69	3705259	3176	1042.65	22	0	30	11	44	28.17	6.37	Aerobic	Scarcity
GCA_913043195	c__Vicinamibacteria	o__Vicinamibacterales	f__UBA8438	SPL	Marine	90.04	65.01	3710071	3622	918.47	15	0	28	15	48	29.29	6.10	Aerobic	Scarcity
GCA_913043225	c__Vicinamibacteria	o__Vicinamibacterales	f__UBA8438	SPL	Marine	92.32	64.26	3853486	3432	1038.05	21	0	20	12	43	29.77	6.41	Aerobic	Scarcity
GCA_913043805	c__Vicinamibacteria	o__Vicinamibacterales	f__UBA8438	SPL	Marine	92.59	66.81	3704349	4113	834.96	36	0	24	17	51	29.47	5.98	Aerobic	Scarcity
GCA_913043965	c__Vicinamibacteria	o__Vicinamibacterales	f__UBA823	SPL	Marine	92.90	54.26	2503777	2286	1016.60	21	0	8	15	37	32.05	6.12	Aerobic	Scarcity
GCA_913051555	c__Vicinamibacteria	o__Vicinamibacterales	f__UBA823	SPL	Marine	92.87	67.48	3703464	3319	1037.84	27	0	16	24	45	29.75	6.40	Aerobic	Scarcity
GCA_913052025	c__Vicinamibacteria	o__Vicinamibacterales	f__UBA8438	SPL	Marine	89.55	65.29	4921911	4775	922.88	34	3	46	15	58	28.25	5.79	Aerobic	Scarcity
GCA_913052135	c__Vicinamibacteria	o__Vicinamibacterales	f__UBA823	SPL	Marine	93.01	50.42	2392996	2205	1009.49	19	0	8	20	34	31.67	5.94	Aerobic	Scarcity
GCA_913052155	c__Vicinamibacteria	o__Vicinamibacterales	f__UBA823	SPL	Marine	92.39	59.74	2367942	2226	982.77	16	1	12	14	36	33.11	6.00	Aerobic	Scarcity
GCA_913063145	c__Vicinamibacteria	o__Vicinamibacterales	f__UBA8438	SPL	Marine	88.83	66.44	3905387	3830	904.35	25	0	24	15	61	30.33	6.32	Aerobic	Scarcity
GCA_934541105	c__Vicinamibacteria	o__Vicinamibacterales	f__Vicinamibacteraceae	SPL	Host	94.04	69.90	4033206	3457	1098.95	23	1	12	63	45	31.25	6.20	Aerobic	Scarcity
GCA_945866285	c__Vicinamibacteria	o__Vicinamibacterales	f__UBA2999	SPL	Freshwater	94.05	65.33	2801441	2777	949.51	30	0	12	18	49	28.84	6.04	Aerobic	Scarcity
GCA_945876205	c__Vicinamibacteria	o__Vicinamibacterales	f__UBA2999	SPL	Freshwater	93.28	65.45	4064614	4036	939.98	32	0	8	15	38	31.62	6.09	Aerobic	Scarcity
GCA_945897165	c__Vicinamibacteria	o__Vicinamibacterales	f__UBA2999	SPL	Freshwater	93.80	65.11	3150157	2997	987.79	33	0	17	15	45	28.7	6.60	Aerobic	Scarcity
GCA_945903905	c__Vicinamibacteria	o__Vicinamibacterales	f__SCN_69_37	SPL	Freshwater	93.87	64.33	3441543	3208	1008.86	22	2	8	24	41	28.06	6.21	Aerobic	Scarcity

Table S5. ANOVA results for the effect of Acidobacteriota class, the habitat from which the genome originated, and the interaction between the two on various genomic features. Results of Tukey post-hoc pairwise comparisons are shown for the significant factors.

**Genomic feature**		**Factor**	**Pairwise comparison**	**p-value[a]**	**% contribution[b]**
**General genomic features**	**Coding density**	Class		< 2e-16	10.21%
			c__Blastocatellia – c__Aminicenantia	7.24E-05	
			c__Holophagae – c__Blastocatellia	0	
			c__Terriglobia – c__Aminicenantia	0	
			c__Terriglobia – c__Holophagae	0	
			c__Thermoanaerobaculia – c__Aminicenantia	1.44E-05	
			c__Vicinamibacteria – c__Aminicenantia	0.001	
		Habitat		NS	NS
		Class:habitat		5.55E-06	1.49%
			c__Blastocatellia:Soil – c__Blastocatellia:Non-soil	0	
			c__Terriglobia:Soil – c__Terriglobia:Non-soil	0	
	**GC percentage**	Class		< 2e-16	41.18%
			c__Blastocatellia – c__Aminicenantia	3.90E-06	
			c__Holophagae – c__Blastocatellia	0	
			c__Terriglobia – c__Aminicenantia	0	
			c__Terriglobia – c__Holophagae	0	
			c__Thermoanaerobaculia – c__Aminicenantia	0	
			c__Thermoanaerobaculia – c__Holophagae	0	
			c__Vicinamibacteria – c__Aminicenantia	0	
			c__Vicinamibacteria – c__Holophagae	0.026	
		Habitat		4.21E-06	0.63%
		Class:habitat		2.59E-09	1.46%
			c__Aminicenantia:Soil – c__Aminicenantia:Non-soil	2.05E-03	
			c__Terriglobia:Soil – c__Terriglobia:Non-soil	4.00E-06	
	**Genome size**	Class		< 2e-16	10.16%
			c__Blastocatellia – c__Aminicenantia	0	
			c__Holophagae – c__Blastocatellia	0	
			c__Terriglobia – c__Aminicenantia	0	
			c__Terriglobia – c__Holophagae	0	
			c__Thermoanaerobaculia – c__Aminicenantia	0	
			c__Thermoanaerobaculia – c__Holophagae	0	
			c__Vicinamibacteria – c__Aminicenantia	1.00E-07	
			c__Vicinamibacteria – c__Holophagae	4.00E-07	
		Habitat		NS	NS
		Class:habitat		4.59E-04	
			c__Vicinamibacteria:Soil – c__Vicinamibacteria:Non-soil	1.71E-04	
	**Protein count**	Class		< 2e-16	11.06%
			c__Blastocatellia – c__Aminicenantia	0	
			c__Holophagae – c__Blastocatellia	0	
			c__Terriglobia – c__Aminicenantia	0	
			c__Terriglobia – c__Holophagae	0	
			c__Thermoanaerobaculia – c__Aminicenantia	0	
			c__Thermoanaerobaculia – c__Holophagae	0	
			c__Vicinamibacteria – c__Aminicenantia	0	
			c__Vicinamibacteria – c__Holophagae	0	
		Habitat		NS	NS
		Class:habitat		1.50E-04	
			c__Terriglobia:Soil – c__Terriglobia:Non-soil	0.016	
			c__Vicinamibacteria:Soil – c__Vicinamibacteria:Non-soil	1.00E-07	
	**Average gene length**	Class		3.12E-16	4.14%
			c__Terriglobia – c__Aminicenantia	3.00E-07	
			c__Terriglobia – c__Holophagae	0	
			c__Thermoanaerobaculia – c__Aminicenantia	8.76E-04	
			c__Thermoanaerobaculia – c__Holophagae	3.86E-05	
		Habitat		NS	NS
		Class:habitat		8.50E-04	
			c__Blastocatellia:Soil – c__Blastocatellia:Non-soil	0	
			c__Terriglobia:Soil – c__Terriglobia:Non-soil	0	
			c__Thermoanaerobaculia:Soil – c__Thermoanaerobaculia:Non-soil	1.00E-06	
			c__Vicinamibacteria:Soil – c__Vicinamibacteria:Non-soil	1.24E-04	

**Phage infection/immunity**	**CRISPRs**	Class		1.02E-11	3.08%
			c__Vicinamibacteria – c__Holophagae	4.59E-03	
		Habitat		NS	NS
		Class:habitat		3.89E-03	
	**Viral contigs**	Class		<2.2e-16	5.21%
			c__Blastocatellia – c__Aminicenantia	0.034	
			c__Holophagae – c__Blastocatellia	0.046	
			c__Terriglobia – c__Aminicenantia	3.00E-07	
			c__Terriglobia – c__Holophagae	0	
			c__Thermoanaerobaculia – c__Aminicenantia	0.026	
			c__Thermoanaerobaculia – c__Holophagae	0.038	
		Habitat		NS	NS
		Class:habitat		0.012	
			c__Terriglobia:Soil – c__Terriglobia:Non-soil	1.17E-03	
**Extracellular product arsenal**	**BGCs**	Class		7.38E-13	3.35%
			c__Blastocatellia – c__Aminicenantia	8.22E-04	
			c__Holophagae – c__Blastocatellia	0.037	
			c__Terriglobia – c__Aminicenantia	5.72E-04	
			c__Terriglobia – c__Holophagae	1.13E-05	
			c__Thermoanaerobaculia – c__Holophagae	3.00E-06	
			c__Vicinamibacteria – c__Aminicenantia	5.90E-05	
		Habitat		0.012	NS
		Class:habitat		NS	NS
	**CAZYmes**	Class		< 2e-16	28.63%
			c__Holophagae – c__Blastocatellia	0	
			c__Terriglobia – c__Aminicenantia	0	
			c__Terriglobia – c__Holophagae	0	
			c__Vicinamibacteria – c__Holophagae	2.61E-03	
		Habitat		0.006	NS
		Class:habitat		1.20E-04	
			c__Vicinamibacteria:Soil – c__Vicinamibacteria:Non-soil	0.018	
	**Peptidases**	Class		< 2e-16	12.98%
			c__Blastocatellia – c__Aminicenantia	0	
			c__Holophagae – c__Blastocatellia	4.99E-03	
			c__Terriglobia – c__Aminicenantia	0	
			c__Terriglobia – c__Holophagae	0	
			c__Thermoanaerobaculia – c__Aminicenantia	0	
			c__Vicinamibacteria – c__Aminicenantia	0	
		Habitat		NS	NS
		Class:habitat		3.35E-06	1.49%
			c__Terriglobia:Soil – c__Terriglobia:Non-soil	0	
			c__Vicinamibacteria:Soil – c__Vicinamibacteria:Non-soil	0	
**Predicted physiological optima**	**predicted OGT**	Class		< 2e-16	26.04%
			c__Blastocatellia – c__Aminicenantia	0	
			c__Terriglobia – c__Aminicenantia	0	
			c__Thermoanaerobaculia – c__Aminicenantia	0	
			c__Thermoanaerobaculia – c__Holophagae	0.011	
			c__Vicinamibacteria – c__Aminicenantia	0	
			c__Vicinamibacteria – c__Holophagae	1.72E-04	
		Habitat		5.00E-03	NS
		Class:habitat		3.41E-08	1.64%
			c__Terriglobia:Soil – c__Terriglobia:Non-soil	0	
	**Predicted pH**	Class		<2e-16	21.13%
			c__Blastocatellia – c__Aminicenantia	0	
			c__Holophagae – c__Blastocatellia	0	
			c__Terriglobia – c__Aminicenantia	0	
			c__Terriglobia – c__Holophagae	0	
			c__Vicinamibacteria – c__Aminicenantia	0.002	
			c__Vicinamibacteria – c__Holophagae	9.98E-05	
		Habitat		0.008	NS

		Class:habitat		<2e-16	4.89%
			c__Terriglobia:Soil – c__Terriglobia:Non-soil	0	
	**Predicted O2**	Class		<2e-16	36.09%
			c__Blastocatellia – c__Aminicenantia	0	
			c__Holophagae – c__Blastocatellia	0	
			c__Terriglobia – c__Aminicenantia	0	
			c__Terriglobia – c__Holophagae	0	
			c__Thermoanaerobaculia – c__Aminicenantia	0	
			c__Thermoanaerobaculia – c__Holophagae	0	
			c__Vicinamibacteria – c__Aminicenantia	0	
			c__Vicinamibacteria – c__Holophagae	0	
		Habitat		NS	NS
		Class:habitat		NS	
**Predicted life history strategy**	**Life history strategy**	Class		1.95E-07	2.01%
			c__Terriglobia – c__Aminicenantia	0	
			c__Terriglobia – c__Holophagae	0	
		Habitat		NS	NS
		Class:habitat		0.004	NS
			c__Terriglobia:Soil – c__Terriglobia:Non-soil	0	

a: F-test p-values < 1x10-5 were considered significant for each factor. However, a value of 0.05 was used for pairwise comparisons.

b: Percent contribution of class and habitat to the overall variance calculated using the sum of squares. Percent contribution is not shown for non-significant (NS) factors.

Table S6. Logistic regression results for the effect of Acidobacteria lineage (SPL versus NSPL) and the habitat from which the genome originated on various metabolic functions annotated in the genomes. The

Classification	KEGG Module/Gene[a]	KEGG module description/ Gene description[a]	Lineage-specific comparisons			Habitat-specific comparisons		
			p-value[b]	% contribution[c]	Verdict	p-value[b]	% contribution[c]	Verdict
Amino acid biosynthesis								
	M00031	Lysine biosynthesis, mediated by LysW, 2-aminoadipate => lysine	2.20E-16	25.48	Higher in SPL	3.01E-08	8.72	Higher in Genomes from Soil Sources
	M00016	Lysine biosynthesis, succinyl-DAP pathway, aspartate => lysine	2.00E-16	4.65	Higher in SPL	NS	NS	
	M00526	Lysine biosynthesis, DAP dehydrogenase pathway, aspartate => lysine	1.40E-11	2.44	Higher in SPL	NS	NS	
	M00028	Ornithine biosynthesis, glutamate => ornithine	2.00E-16	25.63	Higher in SPL	NS	NS	
	M00763	Ornithine biosynthesis, mediated by LysW, glutamate => ornithine	2.20E-16	25.48	Higher in SPL	3.01E-08	8.72	Higher in Genomes from Soil Sources
	M00844	Arginine biosynthesis, ornithine => arginine	5.33E-10	1.36	Higher in SPL	0.046	NS	
	M00029	Urea cycle	2.00E-16	16.51	Higher in SPL	NS	NS	
	M00018	Threonine biosynthesis, aspartate => homoserine => threonine	2.20E-16	5.63	Higher in SPL	2.89E-03	NS	
	M00019	Valine/isoleucine biosynthesis, pyruvate => valine / 2-oxobutanoate => isoleucine	7.76E-11	1.61	Higher in SPL	NS	NS	
	M00570	Isoleucine biosynthesis, threonine => 2-oxobutanoate => isoleucine	2.16E-09	1.36	Higher in SPL	NS	NS	
	M00026	Histidine biosynthesis, PRPP => histidine	2.12E-15	9.10	Higher in SPL	4.17E-07	0.99	
	M00034	Methionine salvage pathway	1.35E-09	5.16	Higher in SPL	NS	NS	
	M00022	Shikimate pathway, phosphoenolpyruvate + erythrose-4P => chorismate	2.00E-16	33.01	Higher in SPL	0.039	NS	
	M00023	Tryptophan biosynthesis, chorismate => tryptophan	2.20E-16	20.50	Higher in SPL	5.67E-06	0.25	Higher in Genomes from Soil Sources
	M00015	Proline biosynthesis, glutamate => proline	2.00E-16	9.53	Higher in NSPL	NS	NS	
Cofactor Biosynthesis								
	M00930	Menaquinone biosynthesis, futalosine pathway	2.20E-16	11.51	Higher in SPL	6.14E-04	NS	
	M00846	Siroheme biosynthesis, glutamyl-tRNA => siroheme	3.93E-13	1.56	Higher in SPL	6.69E-05	NS	
	M00926	Heme biosynthesis, bacteria, glutamyl-tRNA => coproporphyrin III => heme	2.20E-16	2.25	Higher in SPL	2.96E-07	3.62	Higher in Genomes from Soil Sources
	M00121	Heme biosynthesis, plants and bacteria, glutamate => heme	6.00E-14	1.67	Higher in SPL	1.35E-07	3.43	Higher in Genomes from Soil Sources
	M00916	Pyridoxal-P biosynthesis, R5P + glyceraldehyde-3P + glutamine => pyridoxal-P	2.00E-16	23.81	Higher in SPL	NS	NS	
	M00912	NAD biosynthesis, tryptophan => quinolinate => NAD	2.20E-16	9.56	Higher in SPL	0.008	NS	
	M00115	NAD biosynthesis, aspartate => quinolinate => NAD	1.65E-10	0.87	Higher in NSPL	2.20E-16	5.12	Higher in Genomes from NonSoil Sources
	M00913	Pantothenate biosynthesis, 2-oxoisovalerate/spermine => pantothenate	2.00E-16	1.91	Higher in SPL	2.00E-16	6.50	Higher in Genomes from Soil Sources
	M00119	Pantothenate biosynthesis, valine/L-aspartate => pantothenate	2.20E-16	1.82	Higher in SPL	8.14E-16	5.83	Higher in Genomes from Soil Sources
	M00126	Tetrahydrofolate biosynthesis, GTP => THF	2.00E-16	11.46	Higher in SPL	2.00E-16	6.00	Higher in Genomes from NonSoil Sources
	M00881	Lipoic acid biosynthesis, plants and bacteria, octanoyl-ACP => dihydrolipoyl-E2/H	1.57E-15	4.75	Higher in SPL	3.27E-05	NS	
	M00125	Riboflavin biosynthesis, plants and bacteria, GTP => riboflavin/FMN/FAD	2.20E-16	9.37	Higher in SPL	9.89E-06	0.31	
	M00880	Molybdenum cofactor biosynthesis, GTP => molybdenum cofactor	NS	NS		2.94E-08	2.83	Higher in Genomes from NonSoil Sources
	M00896	Thiamine biosynthesis, archaea, AIR (+ NAD+) => TMP/TPP	2.00E-16	18.41	Higher in NSPL	0.047	NS	
	M00899	Thiamine salvage pathway, HMP/HET => TMP	2.00E-16	15.53	Higher in NSPL	NS	NS	
	M00123	Biotin biosynthesis, pimeloyl-ACP/CoA => biotin	2.20E-16	7.39	Higher in NSPL	2.02E-04	NS	
	M00577	Biotin biosynthesis, BioW pathway, pimelate => pimeloyl-CoA => biotin	2.20E-16	7.39	Higher in NSPL	2.02E-04	NS	
	M00950	Biotin biosynthesis, BioU pathway, pimeloyl-ACP/CoA => biotin	2.20E-16	8.14	Higher in NSPL	5.56E-05	NS	
Biosynthesis – others								
	M00565	Trehalose biosynthesis, D-glucose 1P => trehalose	2.00E-16	7.22	Higher in SPL	0.035	NS	
	M00554	Nucleotide sugar biosynthesis, galactose => UDP-galactose	2.20E-16	7.69	Higher in SPL	1.29E-05	NS	
	M00793	dTDP-L-rhamnose biosynthesis	2.20E-16	2.56	Higher in SPL	7.17E-13	4.71	Higher in Genomes from Soil Sources
	M00909	UDP-N-acetyl-D-glucosamine biosynthesis, prokaryotes, glucose => UDP-GlcNAc	2.00E-16	28.60	Higher in SPL	0.019	NS	
	M00064	ADP-L-glycero-D-manno-heptose biosynthesis	2.00E-16	7.62	Higher in SPL	NS	NS	
	M00091	Phosphatidylcholine (PC) biosynthesis, PE => PC	2.20E-16	11.72	Higher in SPL	2.20E-16	2.80	Higher in Genomes from NonSoil Sources
	M00133	Polyamine biosynthesis, arginine => agmatine => putrescine => spermidine	2.20E-16	12.94	Higher in NSPL	3.59E-04	NS	
	M00923	UDP-L-FucNAm biosynthesis	2.69E-11	12.86	Higher in NSPL	0.010	NS	
	M00761	Undecaprenylphosphate alpha-L-Ara4N biosynthesis, UDP-GlcA => undecaprenyl phosphate alpha-L-Ara4N	2.20E-16	42.47	Higher in NSPL	2.49E-05	NS	
Biosynthesis – Nucleotides								
	M00938	Pyrimidine deoxyribonucleotide biosynthesis, UDP => dTTP	2.2E-16	7.67	Higher in NSPL	3.40E-08	5.36	Higher in Genomes from NonSoil Sources
Central C metabolism								
	M00004*	Pentose phosphate pathway (Pentose phosphate cycle)	2.20E-16	35.01	Higher in SPL	9.26E-05	NS	
	M00006*	Pentose phosphate pathway, oxidative phase, glucose 6P => ribulose 5P	2.20E-16	25.29	Higher in SPL	1.50E-08	8.66	Higher in Genomes from Soil Sources
	M00308	Semi-phosphorylative Entner-Doudoroff pathway, gluconate => glycerate-3P	2.20E-16	14.02	Higher in SPL	6.21E-04	NS	
	M00010	Citrate cycle, first carbon oxidation, oxaloacetate => 2-oxoglutarate	2.20E-16	19.78	Higher in SPL	5.87E-06	0.30	Higher in Genomes from Soil Sources
	M00087	beta-Oxidation	0.004	NS		6.62E-09	1.39	Higher in Genomes from NonSoil Sources
Other Sugar Degradation								
	M00632	Galactose degradation, Leloir pathway, galactose => alpha-D-glucose-1P	2.20E-16	11.51	Higher in SPL	1.14E-05	NS	
	M00061	D-Glucuronate degradation, D-glucuronate => pyruvate + D-glyceraldehyde 3P	2.00E-16	11.51	Higher in SPL	NS	NS	
	M00631	D-Galacturonate degradation (bacteria), D-galacturonate => pyruvate + D-glyceraldehyde 3P	1.06E-14	6.40	Higher in SPL	NS	NS	
Metabolism – Nucelotides								
	M00046	Pyrimidine degradation, uracil => beta-alanine, thymine => 3-aminoisobutanoate	2.00E-16	6.40	Higher in SPL	NS	NS	
	M00546	Purine degradation, xanthine => urea	1.03E-14	4.44	Higher in SPL	5.99E-06	5.02	Higher in Genomes from Soil Sources
Metabolism – Amino acids								
	M00970	Proline degradation, proline => glutamate	2.20E-16	9.31	Higher in SPL	6.61E-04	NS	
	M00948	Hydroxyproline degradation, trans-4-hydroxy-L-proline => 2-oxoglutarate	2.00E-16	7.03	Higher in SPL	NS	NS	
	M00038	Tryptophan metabolism, tryptophan => kynurenine => 2-aminomuconate	2.00E-16	8.09	Higher in SPL	NS	NS	
	M00035	Methionine degradation	9.37E-16	4.07	Higher in SPL	0.017	NS	
Fermentation/ Respiration								
	H2ase	Hydrogenases	2.2E-16	11.60	Higher in NSPL	0.004	NS	
	nife_group_3abd	3b: Directly couples oxidation of NADPH to fermentative evolution of H_2. Or, generates reductant for hydrogenotrophic carbon-fixation by directly coupling oxidation of H_2 to reduction of NAD.	1.53E-08	2.11	Higher in NSPL	NS	NS	
	nife_group_4a_g	4a: Directly couples formate oxidation to H_2 evolution. 4g: May form respiratory complex that couples ferredoxinred oxidation to proton reduction.	0.008	NS		8.60E-09	0.02	Higher in Genomes from Soil Sources
	fefe_group_b	hydrogenogenic fermentation.	7.62E-05	NS		0.002	NS	
	fefe_group_c1	May serve as H_2-sensors in regulatory cascades.	2.00E-16	31.85	Higher in NSPL	NS	NS	
	fefe_group_c3	May serve as H_2-sensors in regulatory cascades.	3.18E-05	NS		0.009	NS	

	nife_group_1	Hydrogenotrophic respiration	1.00E-04	NS		2.23E-11	0.01	Higher in Genomes from Soil Sources
	nife_group_3c	Bifurcates electrons from H2 to heterodisulfide and ferredoxin.	1.04E-06	2.95	Higher in NSPL	NS	NS	
	M00155	Cytochrome c oxidase, prokaryotes	2.00E-16	5.35	Higher in SPL	NS	NS	
	coxAB	Cytochrome oxidase aa3 (low affinity)	2.00E-16	17.01	Higher in SPL	NS	NS	
	ccoNOP	Cytochrome oxidase cbb3 (high affinity)	2.69E-04	NS		2.53E-09	0.86	Higher in Genomes from NonSoil Sources
	dsrABD_asrABC	Sulfite reduction	5.44E-12	6.07	Higher in SPL	NS	NS	
	phsA	Thiosulfate disproportionation	2.20E-16	14.61	Higher in NSPL	2.07E-04	NS	
	fccB_sqr	Sulfide oxidation	2.20E-16	3.43	Higher in NSPL	9.06E-12	8.96	Higher in Genomes from NonSoil Sources
	nxrAB	nitrite oxidation	8.19E-15	11.95	Higher in SPL	2.20E-16	16.42	Higher in Genomes from NonSoil Sources
	anfDKG_nifDK_vnfDKG_nifH	N_2 fixation	0.089	NS		2.61E-07	5.24	Higher in Genomes from Soil Sources
	nirKS_octR	Nitrite reduction	2.20E-16	16.44	Higher in NSPL	1.58E-07	9.95	Higher in Genomes from NonSoil Sources
	napAB_narGH	Nitrate reduction	2.00E-16	3.88	Higher in NSPL	2.00E-16	9.04	Higher in Genomes from NonSoil Sources
	nrfADH_nirBD	Nitrite reduction to ammonia	2.20E-16	2.90	Higher in NSPL	2.20E-16	8.21	Higher in Genomes from NonSoil Sources
	M00530	Dissimilatory nitrate reduction, nitrate => ammonia	2.20E-16	21.15	Higher in NSPL	4.21E-09	14.08	Higher in Genomes from NonSoil Sources
	M00579	Phosphate acetyltransferase-acetate kinase pathway, acetyl-CoA => acetate	2.00E-16	2.74	Higher in NSPL	0.013	NS	
	porA	pyruvate oxidation	1.95E-13	6.26	Higher in NSPL	2.37E-06	0.13	Higher in Genomes from Soil Sources
	Acetate => Acetaldehyde	Ethanol fermentation	5.35E-12	3.10	Higher in SPL	0.019	NS	
	Acetaldehyde => Ethanol	Ethanol fermentation	2.20E-16	9.00	Higher in SPL	0.017	NS	
	acs	Acetate to acetyl-CoA	2.20E-16	5.14	Higher in SPL	0.005	NS	
Antimicrobials/ Resiustance								
	M00777	Avermectin biosynthesis, 2-methylbutanoyl-CoA/isobutyryl-CoA => 6,8a-Seco-6,8a-deoxy-5-oxoavermectin 1a/1b aglycone => avermectin A1a/B1a/A1b/B1b	2.00E-16	9.40	Higher in SPL	NS	NS	
	M00627	beta-Lactam resistance, Bla system	2.20E-16	3.76	Higher in SPL	1.99E-05	NS	

a: Functions significantly higher in SPL classes are colored in green, while those significantly higher in NSPL classes are colored in magenta. Only two functions (highlighted by an *) were found to be significantly affected by the interaction between the two variables (lineage and habitat).

b: Chi-squared test p-values $< 1x10^{-5}$ were considered significant for each factor.

c: McFadden pseudo R2 was calculated for the full model and for the model when the variable (lineage or habitat) was removed to assess the variable contribution to the whole model. % contribution is not shown for non-significant (NS) factors.

Supplementary figures.

Figure S1. Habitat sources of the 2028 Acidobacteriota genomes in GTDB. Acidobacteriota classes are shown on the X-axis. The 6 classes compared in this study are highlighted with an *. These are the classes with at least 100 genomes and for which genomes were recovered from at least 5 of the 7 habitats.

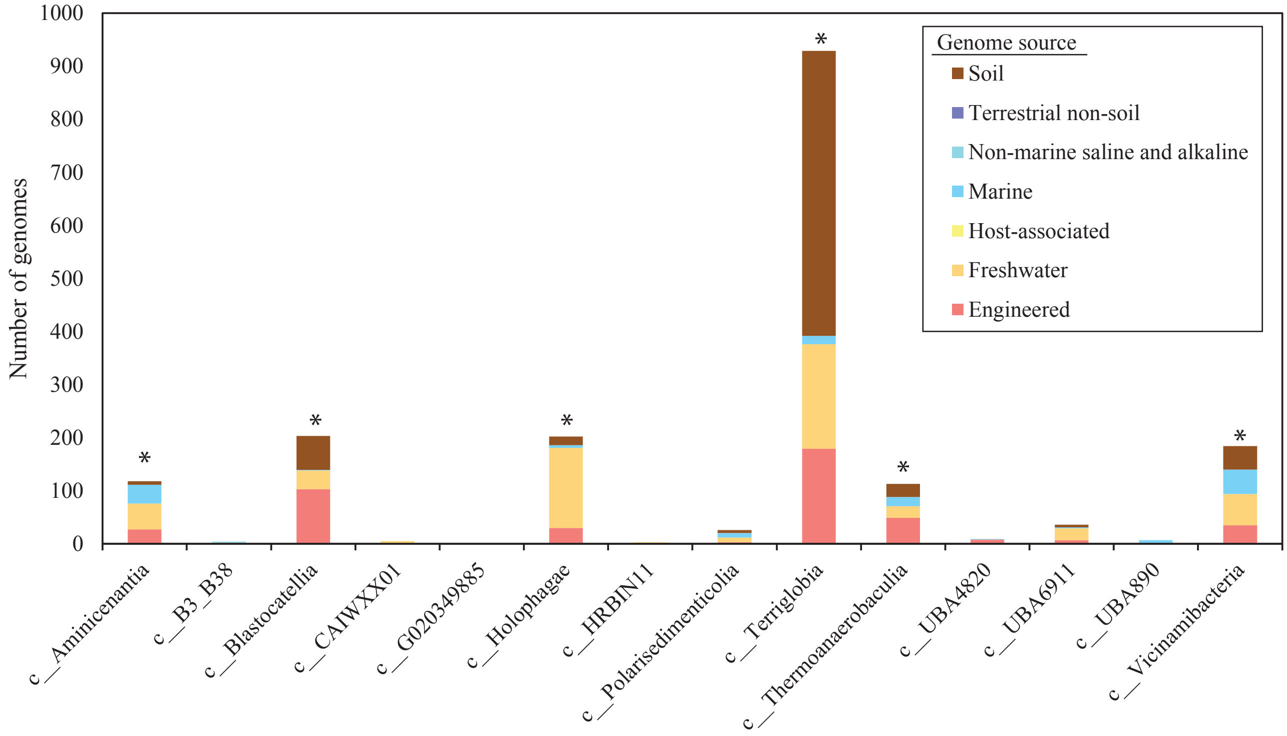

Number of genomes
1000
900
800
700
600
500
400
300
200
100
0
Genome source
Soil
Terrestrial non-soil
Non-marine saline and alkaline
Marine
Host-associated
Freshwater
Engineered
*
c__Aminicenantia
c__B3_B38
c__Blastocatellia
c__CAIWXX01
c__G020349885
c__Holophagae
c__HRBIN11
c__Polarisedimenticolia
c__Terriglobia
c__Thermoanaerobaculia
c__UBA4820
c__UBA6911
c__UBA890
c__Vicinamibacteria

Figure S2. Phylogenomic tree constructed using the GTDB concatenated alignment of 120 single copy marker gene. The tree was constructed in FastTree [3] and is wedged at the genus level. Wedges are color coded by class. The heatmap around the tree shows the number of genomes belonging to each genus. The bar charts show the distribution of the habitats from which the genomes were recovered. Genera with genomes obtained from more than one habitat were considered habitat-generalist as shown in Table S3.

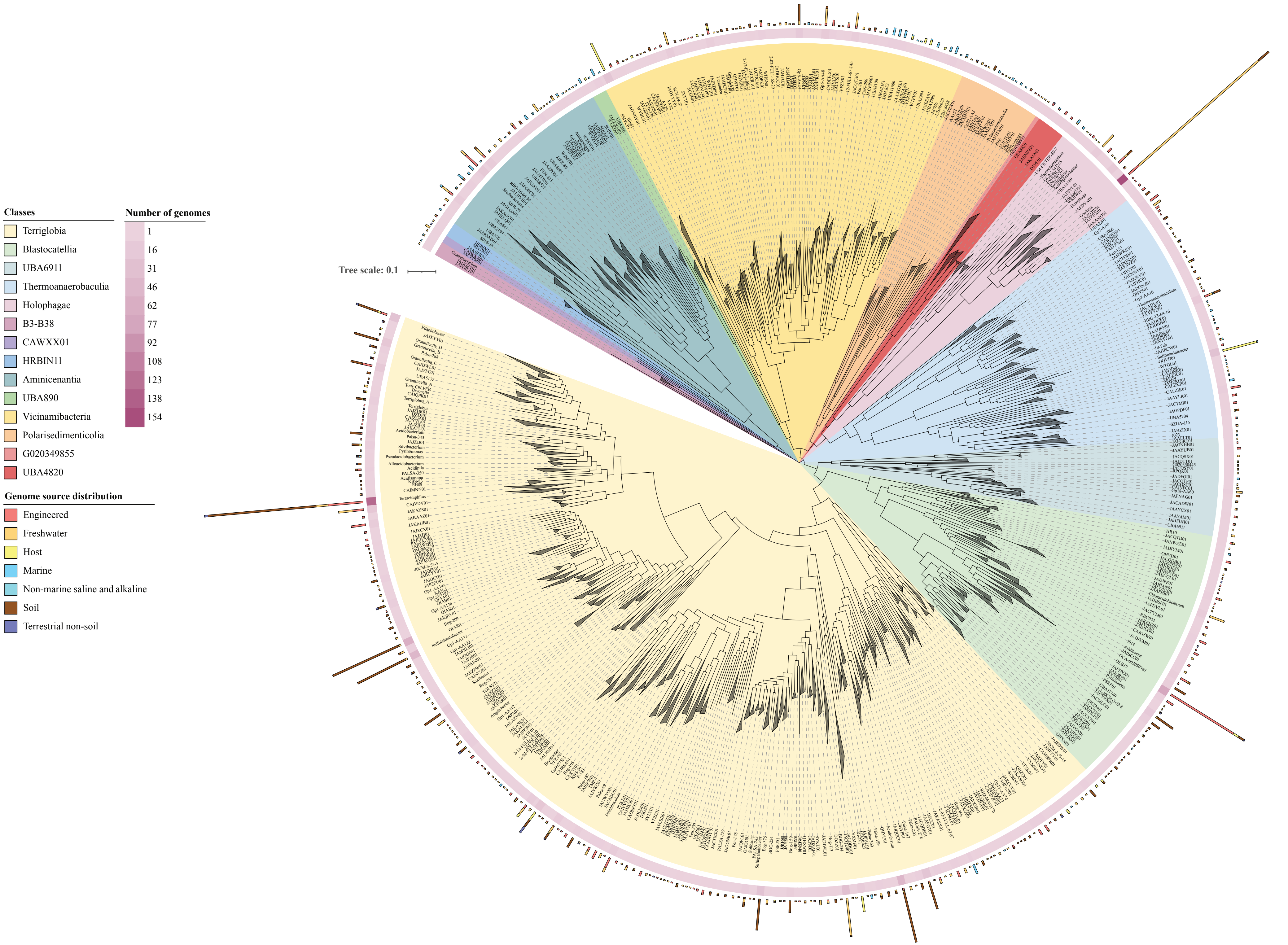
Classes
Terriglobia
Blastocatellia
UBA6911
Thermoanaerobaculia
Holophagae
B3-B38
CAWXX01
HRBIN11
Aminicenantia
UBA890
Vicinamibacteria
Polarisedimenticola
G020349855
UBA4820
Number of genomes
1
16
31
46
62
77
92
108
123
138
154
Genome source distribution
Engineered
Freshwater
Host
Marine
Non-marine saline and alkaline
Soil
Terrestrial non-soil
Tree scale: 0.1

References

1. Barns SM, Cain EC, Sommerville L *et al.* Acidobacteria phylum sequences in uranium-contaminated subsurface sediments greatly expand the known diversity within the phylum. *Appl Environ Microbiol* 2007;**73**:3113-6. https://doi.org/10.1128/aem.02012-06

2. Dedysh SN, Yilmaz P. Refining the taxonomic structure of the phylum Acidobacteria. *Int J Syst Evol Microbiol* 2018;**68**:3796-806. https://doi.org/10.1099/ijsem.0.003062

3. Price MN, Dehal PS, Arkin AP. FastTree 2 – Approximately Maximum-Likelihood Trees for Large Alignments. *PLOS ONE* 2010;**5**:e9490. https://doi.org/10.1371/journal.pone.0009490
